# From Simplicity to Complexity: Substituent‐Controlled *Z*′ Multiplicity as a Design Principle for Thermally Robust Yet Programmable Anisotropic Expansion

**DOI:** 10.1002/smll.75273

**Published:** 2026-08-18

**Authors:** Yuto Hino, Takumi Matsuo, Tomohiro Yamashita, Hiroko Yokota, Daiki Korenaga, Tomohiro Seki, Shotaro Hayashi

**Affiliations:** ^1^ School of Engineering Science Kochi University of Technology Kami Kochi Japan; ^2^ FOREST Center Research Institute Kochi University of Technology Kami Kochi Japan; ^3^ School of Materials and Chemical Technology Institute of Science Tokyo Yokohama Kanagawa Japan; ^4^ Department of Chemistry Faculty of Science Shizuoka University Shizuoka Japan

**Keywords:** anisotropic thermal expansion, anthracenes, dynamic crystals, molecular crystals, Z’

## Abstract

Anisotropic thermal expansion in molecular crystals has predominantly been reported for low‐*Z*′ systems (*Z*′ ≤ 2), in which only a few independent molecules occupy the asymmetric unit. In such crystals, thermal expansion is often interpreted using simplified models based on rigid molecular motion or averaged intermolecular interactions. However, these descriptions fail to capture the complex anisotropy, switchable responses, and multistep phase transitions frequently observed in molecular solids. Here, we report high‐*Z*′ molecular crystals that exhibit both thermal robustness and stepwise, rapid anisotropic thermal expansion upon temperature variation. These crystals were designed by combining substituent‐induced molecular asymmetry, competitive intermolecular interactions, and flexible intramolecular degrees of freedom. Crystals of 2,6‐bis(4‐*tert*‐butylphenyl)anthracene display an unusually high *Z*′ value of 5 over a wide temperature range (100–400 K), enabled by the noncovalent immobilization of coplanar *tert*‐butyl groups within a herringbone packing framework. Upon heating, progressive molecular motions induce dynamic averaging of crystallographically distinct local environments, culminating in a cooperative phase transition accompanied by symmetry recovery and reduction of *Z*' from 5 to 2.5 at 423 K. The resulting structural reorganization gives rise to rapid macroscopic anisotropic thermal expansion. These results establish high‐*Z*′ crystal engineering as a viable strategy for programmable and reversible thermal‐mechanical responses.

## Graphical abstract

Leveraging substituent‐controlled packing competition in 2,6‐bis(4‐*tert*‐butylphenyl)anthracene, we developed a high‐*Z*′ symmetry‐amplification strategy that controls crystallographic inequivalence. This strategy enables reversible, thermally robust phase transitions and programmable anisotropic thermal expansion within a single molecular crystal.

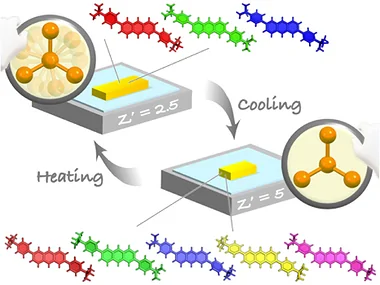

## 1 Introduction

Thermal robustness—the ability of a solid to nondestructively and reversibly accommodate changes in shape over a wide temperature range—has attracted considerable interest as a structural design guideline for phase‐change materials operating under extreme low‐ and high‐temperature conditions, thermal management materials for energy control, and crystal engineering in general [1, 2, 3, 4, 5, 6, 7, 8, 9, 10]. However, conventional thermal expansion, which originates from atomic thermal vibrations, is intrinsically limited in terms of anisotropy, accessible temperature range, magnitude, and responsiveness, making it difficult to optimize freely. Molecular crystals, owing to their versatile chemical structures, provide an ideal platform for precise control of thermal responses in solids, as subtle orientational changes and molecular motions can be activated by temperature stimuli. Since Mason et al. reported anisotropic thermal expansion in anthracene crystals in 1964, a wide variety of molecular crystals have been shown to exhibit diverse and highly anisotropic thermal expansion behaviors [11, 12, 13, 14, 15]. In contrast to continuous thermal expansion, phase‐transition molecular crystals—whose internal order dynamically switches with temperature—can markedly amplify thermal expansion through changes in molecular mobility and packing, thereby enabling anisotropic responses over a broader temperature range. A representative example is the thermosalient effect observed in palladium complexes, first reported by Etter and Siedle in 1983, for which Naumov et al. later systematized the underlying transition mechanisms responsible for anisotropic thermal expansion [16, 17, 18]. More recently, chemical strategies to control phase‐transition temperatures, pathways, and reversibility have been actively explored. Various design principles have been proposed, including stepwise expansion based on herringbone packing motifs and rapid phase transitions enabled by molecules bearing rotatable moieties [19, 20, 21]. In particular, the introduction of rotatable terminal groups into rigid molecular cores has been shown to induce reversible martensitic transitions in benzothieno[2‐*b*]benzothiophene derivatives, a concept that has subsequently been generalized to systems with modified core structures [22, 23, 24].

Despite these advances, a central challenge in this field remains unresolved: the structural factors that govern the initiation and propagation pathways of phase transitions are still poorly understood. Most phase‐transition molecular crystals reported to date are low‐Z′ systems, in which the number of independent molecules in the asymmetric unit is limited to Z′ ≤ 2. While such systems have provided valuable insights into thermally activated molecular motions—including well‐known examples such as pedal motion and temperature‐dependent disorder—structural simplicity inherently limits the diversity of distinct local molecular environments that can coexist within a single crystal. As a result, the design and systematic investigation of hierarchical, multi‐step switching processes involving inequivalent molecules remain largely unexplored [25, 26, 27, 28]. In contrast, high‐*Z*′ crystals (*Z*′ ≥ 4) provide a structurally rich platform in which multiple independent molecules experience distinct local environments, potentially enabling stepwise and selective molecular motions. However, the dynamics of multistep phase transitions in high‐*Z*′ molecular crystals induced by temperature changes have not yet been systematically elucidated. As a result, a substantial knowledge gap remains in understanding how molecular framework design, substituent architecture, and hierarchies of intermolecular interactions collectively dictate phase‐transition pathways—aside from a limited number of examples involving hydrogen‐bonded high‐*Z*′ crystals [29, 30]. In this study, we address this gap by reporting high‐*Z*′ anthracene crystals that combine thermal robustness with multistep, reversible phase transitions, using 2,6‐bis(*tert*‐butylphenyl)anthracene (**BBPA**) as a rationally designed molecular platform. This approach builds directly on our previous studies on crystal‐structure formation and phase‐transition control in anthracene crystals substituted at the 9,10‐ and 1,8‐positions [31, 32, 33, 34]. These studies revealed that subtle changes in substitution positions critically influence both packing symmetry and molecular mobility, suggesting that the longitudinal 2,6‐axis of the anthracene framework represents an unexplored handle for simultaneously programming packing anisotropy and dynamic degrees of freedom.

Based on this insight, we hypothesized that: (*i*) introduction of aryl substituents at the 2,6‐positions—corresponding to the longitudinal axis of the anthracene skeleton—induces effective C_2v_ symmetry and promotes herringbone packing; and (*ii*) incorporation of *tert*‐butyl‐substituted aryl groups, in which the sterically demanding and pseudo‐C_3_‐symmetric *tert*‐butyl units possess multiple low‐barrier methyl rotations and prevent complete coplanarization, generates dynamically accessible conformational diversity. This multiplicity of rotational and steric degrees of freedom creates distinct local molecular environments, thereby enabling the construction of high‐Z′ crystals and the modulation of anisotropic thermal expansion. To test this hypothesis, we designed and synthesized **BBPA**, which successfully crystallized into a high‐*Z*' crystal structure containing multiple crystallographically independent molecular environments. In these high‐*Z*′ crystals, the molecular degrees of freedom introduced by rational design are spatially differentiated and hierarchically accumulated within the crystal lattice, functioning as scaffolds for concerted and selective molecular motions. Consequently, **BBPA** crystals display a reversible symmetry‐increasing phase transition accompanied by a reduction of *Z*' from 5 to 2.5 over an exceptionally wide temperature range (100–473 K), accompanied by pronounced anisotropic thermal expansion. Furthermore, comparative analysis with 2,6‐diphenylanthracene (**DPA**) clarifies how the number of rotational sites governs the emergence of independent molecular environments and influences the resulting phase‐transition behavior. Collectively, these results provide insights into how molecular‐level rotational freedom can be translated into controllable crystal‐scale structural responses.

## 2 Results and Discussion

**BBPA** and **DPA** (Figure 1a) were synthesized via Suzuki–Miyaura cross‐coupling reactions (see Supporting Information for scheme and experimental details). Their chemical structures were unambiguously confirmed by ^1^H and ^13^C nuclear magnetic resonance (NMR) spectroscopy and high‐resolution mass spectrometric (HRMS) analysis (see Supporting Information). Single crystals suitable for single‐crystal x‐ray diffraction (SCXRD) were obtained by allowing supersaturated solutions of **BBPA** and **DPA** to stand under sealed conditions (see Supporting Information). The number of independent molecules (*Z*′) in the asymmetric units of the **BBPA** and **DPA** crystals was first determined by SCXRD. Measurements performed on a laboratory diffractometer (*λ* = 0.71073 Å, 103 K) revealed that **BBPA** crystallizes in the monoclinic space group *P*2_1_ with a remarkably high *Z*′ value of 5 (Figure 1b and Figure S2). The crystal structure features a herringbone arrangement of the anthracene cores stabilized by CH–π interactions (Figure 1b, top), while the *tert*‐butyl substituents assemble into a dense two‐dimensional brick‐wall‐like molecular layer (Figure 1b, bottom). In contrast, under comparable SCXRD conditions, **DPA** crystallizes in the monoclinic space group *P*2_1_/*c* with *Z*′ = 0.5, also adopting a herringbone arrangement of the anthracene cores (Figure 1c and Figure S3). The low *Z*′ values observed for **DPA** and for previously reported *tert*‐butyl‐containing organic crystals indicate that intramolecular rotational freedom alone is a necessary but insufficient condition for achieving high‐*Z*′ crystal structures [35]. In the case of **BBPA**, the presence of ten rotational degrees of freedom, combined with multipoint intermolecular interactions, induces asymmetric molecular conformations that stabilize multiple crystallographically independent molecules within the asymmetric unit. To further elucidate the structural dynamics, in situ variable‐temperature single‐crystal x‐ray diffraction (VT‐SCXRD) measurements were performed on **BBPA** crystals using synchrotron radiation (*λ* = 0.4133 Å). These measurements revealed a progressive increase in disorder of the *tert*‐butyl groups upon heating from 100 to 400 K (Figure 1d and Figure S4). Quantitative analysis based on the independent molecules in the asymmetric unit shows that the number of disordered *tert*‐butyl groups increases from 2 out of 10 at 100 K to 10 out of 10 at 400 K.

**FIGURE 1:**
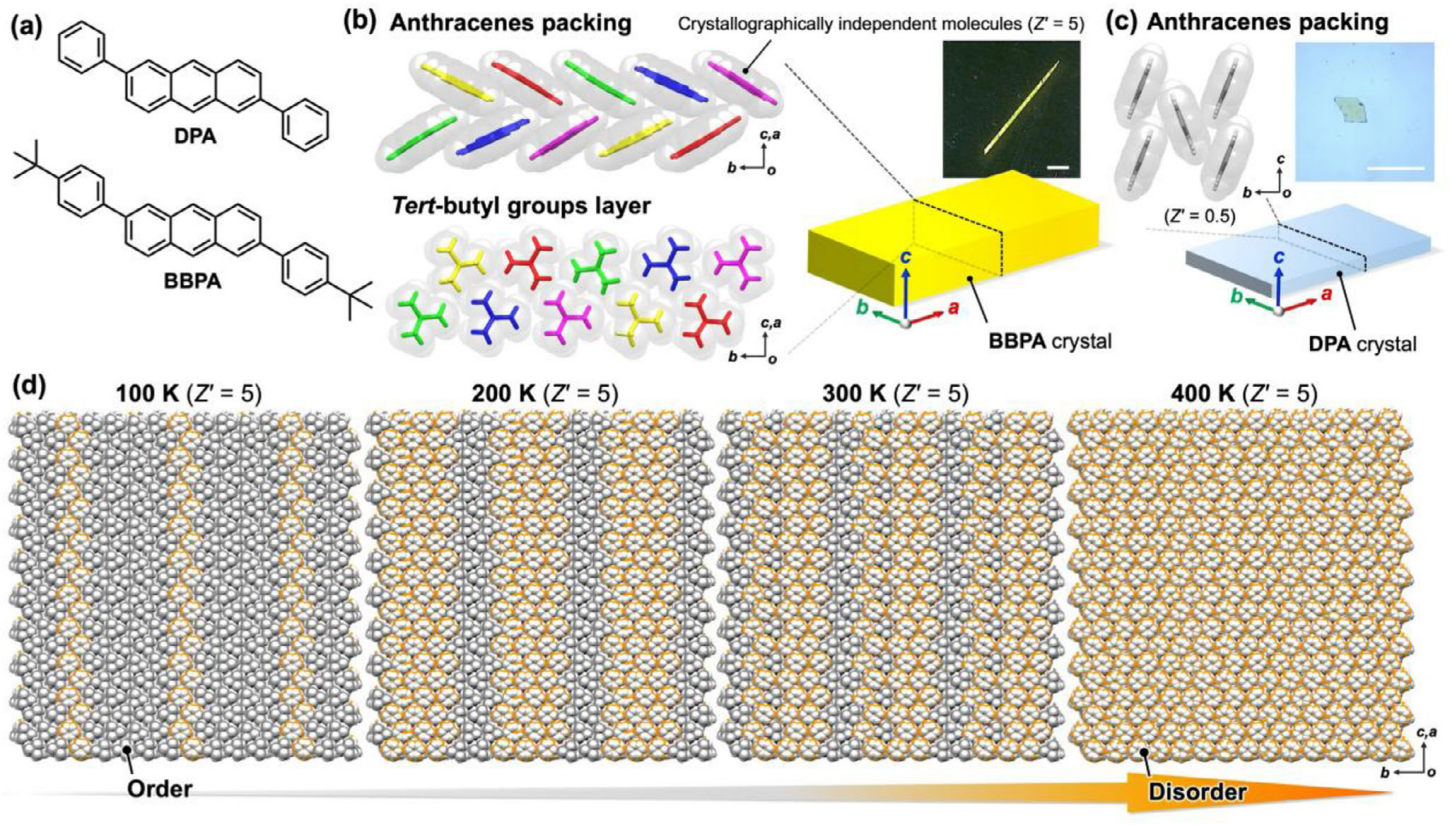
Substituent‐controlled *Z*′ multiplicity. (a) Chemical structures of **BBPA** and **DPA**. (b) Illustration of the **BBPA** crystal and its optical micrograph under ambient light (scale bar: 200 µm). Crystal structure of the anthracene packing and the *tert*‐butyl group layer in **BBPA** (*Z*′ = 5, 103 K). Crystallographically independent molecules are shown as capped‐sticks models (red, yellow, green, blue, and pink) and space‐filling models (gray). Disordered *tert*‐butyl groups are omitted for clarity. (c) Schematic illustration of the **DPA** crystal and its optical micrograph under ambient light (scale bar: 200 µm). Crystal structure of the anthracene packing in **DPA** (*Z*′ = 0.5, 103 K), in which the crystallographically independent molecule is shown using capped‐sticks and space‐filling models (gray). (d) Temperature‐dependent crystal structures of the *tert*‐butyl layer in **BBPA** (*Z*′ = 5, 100–400 K), represented using space‐filling models. Ordered *tert*‐butyl groups are shown in gray, and disordered *tert*‐butyl groups in orange.

VT‐SCXRD of **BBPA** crystals at 100–400 K revealed five crystallographically independent molecules, each adapting a distinct molecular conformation (Figure 2 and Figure S6). Upon heating to 423 K, **BBPA** crystals exhibited a decrease in the number of independent molecules from *Z*′ = 5 to *Z*′ = 2.5, accompanied by a structural phase transition and a change in space group. Concomitantly, the degree of *tert*‐butyl disorder increased progressively during the structural transformation, while the conformational differences among the crystallographically independent molecules became less pronounced. This process was accompanied by an increase in the anthracene centroid–centroid distances along the crystallographic *a*‐axis and a relative displacement between adjacent molecular layers (Figures S8 and S9). As a result, the distinctions among the independent molecular environments were progressively reduced, leading to symmetry enhancement and a reduction of Z′ from 5 to 2.5.

**FIGURE 2:**
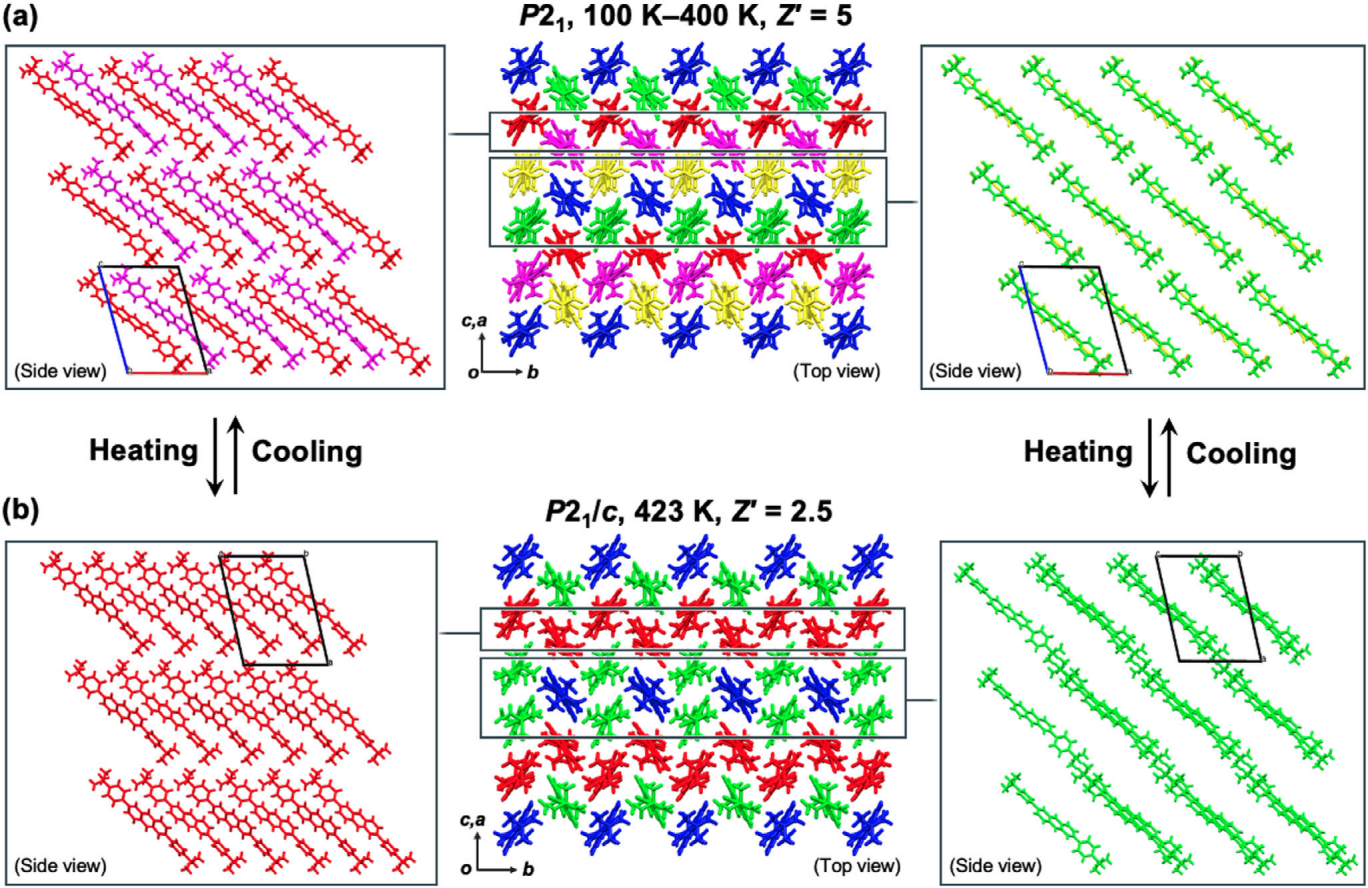
Thermally induced single‐crystal‐to‐single‐crystal phase transition accompanied by space‐group transformation and *Z*′ reduction. (a) Crystal structure of the low‐temperature phase of **BBPA** (*P*2_1_, 100–400 K, *Z*′ = 5). Crystallographically independent molecules are shown as capped‐stick models (red, yellow, green, blue, and purple). Disordered *tert*‐butyl groups are omitted for clarity. (b) Crystal structure of the high‐temperature phase of **BBPA** (*P*2_1_/*c*, 423 K, *Z*′ = 2.5). Crystallographically independent molecules are shown as capped‐stick models (red, green, and blue). Disordered *tert*‐butyl groups are omitted for clarity. The pink molecule merges into the red site, and the yellow molecule merges into the green site in the high‐temperature phase, consistent with the reduction of *Z*′ from 5 to 2.5.

To evaluate the energetic origin of the high‐Z′ structure, intermolecular interaction energies (Δ*E*_int_) were calculated for representative molecular dimers extracted from the crystal structures determined at 100 and 423 K (Figure S27). The low‐temperature phase (Z′ = 5) exhibits a broad distribution of interaction energies, indicating distinct local stabilization environments for the crystallographically independent molecules. This energetic diversity is consistent with the coexistence of multiple nonequivalent packing environments generated by the combined effects of CH–π interactions, steric constraints imposed by the tert‐butyl groups, and their associated rotational degrees of freedom. Upon heating, the interaction‐energy changes are highly non‐uniform: dimers 4 and 5 become substantially destabilized (Δ*E*_int_ = 15.4 and 16.5 kJ mol^−1^, respectively), whereas dimer 2 remains nearly unchanged (Δ*E*_int_ = −1.2 kJ mol^−1^). These results suggest that the phase transition is associated with selective relaxation of specific intermolecular interactions rather than uniform weakening of the interaction network. As the energetic differences among the independent molecular environments become progressively reduced, the crystal undergoes a symmetry increase accompanied by a reduction of Z′ from 5 to 2.5.

To visualize the molecular‐scale dynamics associated with this structural transition, we analyzed the anisotropic displacement parameters (ADPs) [36, 37, 38]. ADPs quantify the time‐averaged atomic displacements within a crystal lattice and thus provide a sensitive measure of thermally activated molecular motion. By mapping the magnitude of the ADPs using a color scale ranging from blue (small displacement) to red (large displacement), we spatially resolved the distribution of dynamic degrees of freedom within the crystal structure (Figure 3d). This analysis reveals that, upon increasing temperature, ADPs increase not only at the *tert*‐butyl moieties but also progressively at the anthracene core and the peripheral phenyl rings. These observations indicate that thermally activated degrees of freedom are not localized to the substituent termini but instead expand cooperatively across the entire molecular framework as the structural transition proceeds.

**FIGURE 3:**
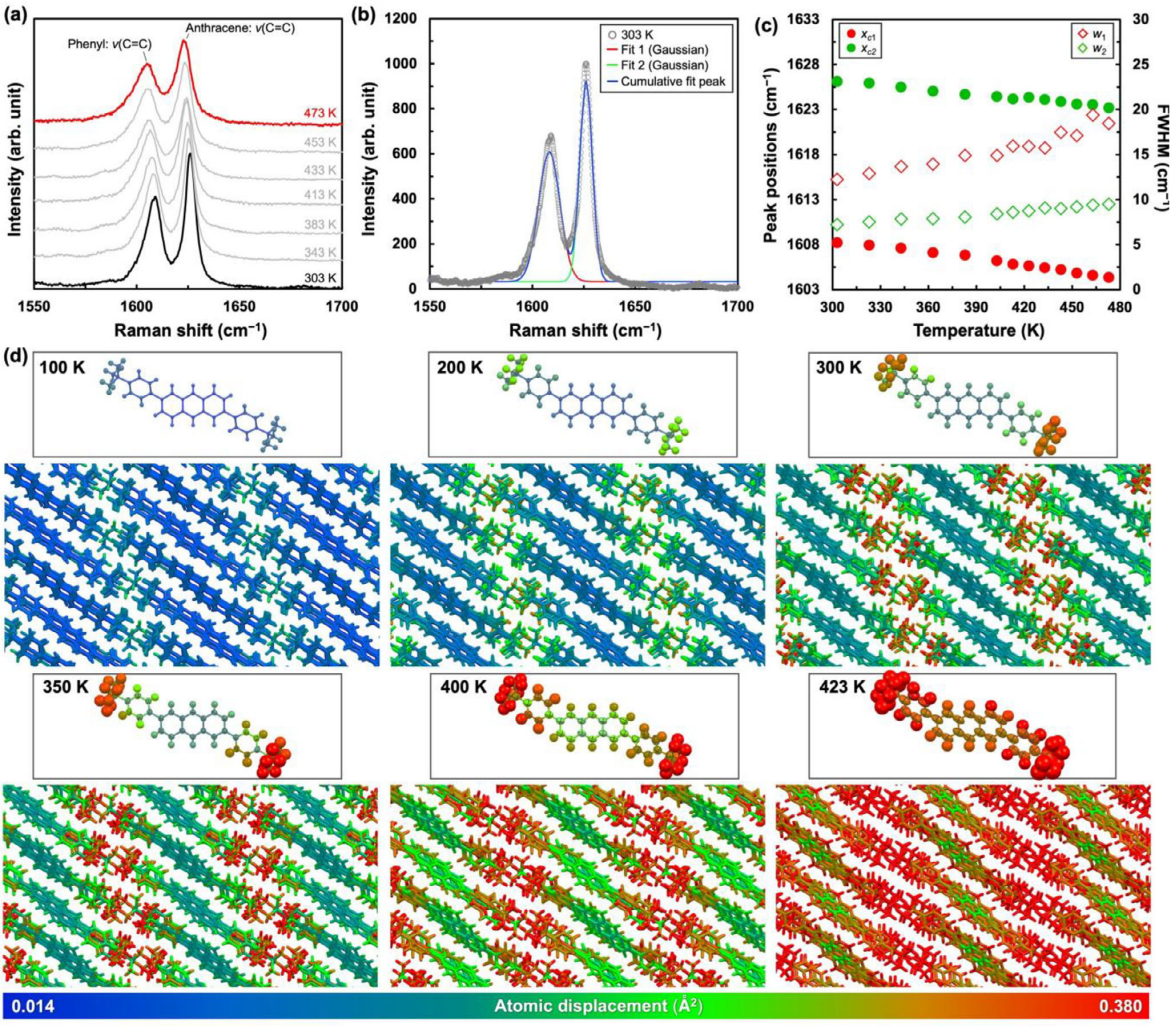
Visualization of thermally activated molecular mobility in **BBPA** crystals. (a) VT‐Raman spectra in the 1550–1700 cm^−1^ region recorded between 303 and 473 K. (b) Raman spectrum fitted with Gaussian functions. The experimental spectrum at 303 K is shown as white dots, with Gaussian components plotted as red (Fit 1) and green (Fit 2) lines, and the cumulative fit shown in blue. (c) Temperature dependence of the peak position, *x*_c1_ for Fit 1 (red circles) and *x*_c2_ for Fit 2 (green circles), and the corresponding FWHM values, *w*_1_ (red diamonds) and *w*_2_ (green diamonds), obtained from Raman spectral fitting. (d) Temperature‐dependent crystal structures visualized using anisotropic atomic displacement parameters (ADPs). Molecular structures are shown as thermal ellipsoid models drawn at the 50% probability level, whereas crystal packing structures are represented as capped‐stick models. Disordered *tert*‐butyl groups are omitted for clarity. Atoms are color‐coded according to the magnitude of their anisotropic displacement parameters (ADPs), with blue indicating smaller atomic displacements and red indicating larger atomic displacements. Atoms are color‐coded according to the magnitude of their ADPs, with blue indicating smaller atomic displacements and red indicating larger atomic displacements.

To capture the continuous modulation of the intramolecular vibrational environment accompanying the phase transition, variable‐temperature Raman (VT‐Raman) spectroscopy was performed on **BBPA** crystals over the range of 303–473 K. This analysis probes the temperature response of intramolecular vibrational modes originating from the anthracene core and the peripheral phenyl rings (Figure 3a and Figure S34). The Raman bands were fitted using Gaussian functions to enable quantitative analysis of their temperature‐dependent behavior (Figures S35–S47). To facilitate assignment of the observed Raman bands, Raman spectra were calculated for each of the five crystallographically independent molecules in the low‐temperature (Z′ = 5) crystal structure at 100 K (Figures S28–S32). The calculated spectra were subsequently averaged to generate a representative spectrum for comparison with the experimental data. The averaged spectrum reproduced the major features of the experimental Raman spectrum and enabled assignment of the observed bands (Figure S33). In particular, the Raman bands in the 1550–1700 cm^−1^ region were assigned to vibrational modes primarily associated with the anthracene core and peripheral phenyl rings. Based on these assignments, the progressive broadening of the corresponding Raman bands upon heating can be attributed to enhanced molecular motions and increasing dynamic disorder within the crystal lattice. This interpretation is consistent with the temperature‐dependent increase in *tert*‐butyl disorder and atomic displacement parameters observed by VT‐SCXRD, indicating that molecular mobility is activated well before the crystallographic phase transition. These observations support the view that dynamic averaging of crystallographically distinct local environments precedes the symmetry increase and reduction of Z′ associated with the phase transition. With increasing temperature, a systematic shift of the Raman peak positions (*x_c_*) toward lower wavenumbers, together with a concomitant increase in the full width at half maximum (FWHM, *w*), was observed in the 1550–1700 cm^−1^ region (Figure 3b,c). These spectral changes reflect a progressive modulation of the local vibrational environment associated with increasing molecular mobility upon heating, consistent with the progressive increase in *tert*‐butyl disorder observed by VT‐SCXRD and indicating that molecular motions become increasingly active as the phase‐transition temperature is approached.

Variable‐temperature powder x‐ray diffraction (VT‐PXRD) measurements of **BBPA** single crystals revealed rapid and stepwise changes in the diffraction patterns upon heating (Figure 4a and Figures S19–S23). In comparison, VT‐PXRD measurements of **DPA** crystals exhibited stepwise changes in diffraction patterns over the temperature range of 303–473 K, albeit without pronounced anisotropic lattice responses (Figure 4b and Figures S24–S26). To directly quantify the lattice strain and thermal expansion behavior of **BBPA** crystals, VT‐SCXRD measurements were performed over the range of 100–453 K (Figure 4c). The lattice parameters were obtained from current‐cell refinements of the VT‐SCXRD datasets, enabling continuous tracking of the lattice evolution up to 453 K, whereas complete crystal structure refinements were performed up to 423 K. The lattice strain develops anisotropically along the crystallographic *a*‐ and *c*‐axes between 100 and 400 K, reflecting the thermal response of the crystal lattice. Upon further heating from 400 to 423 K, the lattice strain increases sharply along the *a*‐axis while decreasing along the *b*‐ and *c*‐axes. This abrupt and highly directional lattice response accompanies the structural transition from the low‐temperature (*Z*′ = 5) phase to the high‐temperature (*Z*′ = 2.5) phase and reflects substantial reorganization of the crystal packing. Consistent with this behavior, the lattice constants of **BBPA** crystals exhibit rapid yet continuous changes across the transition region, accompanied by a sharp increase in the unit‐cell volume near the phase‐transition temperature (Figure 4d). These results indicate that the phase transition is dominated by anisotropic lattice rearrangement rather than uniform lattice expansion. Notably, the crystallographic *a*‐axis—corresponding to the longitudinal direction of the platelet‐shaped crystals—undergoes a significant expansion during the transition from the low‐temperature phase (*Z*′ = 5) to the high‐temperature phase (*Z*′ = 2.5) (Figure 4c), underscoring the highly anisotropic nature of the thermally induced lattice transformation.

**FIGURE 4:**
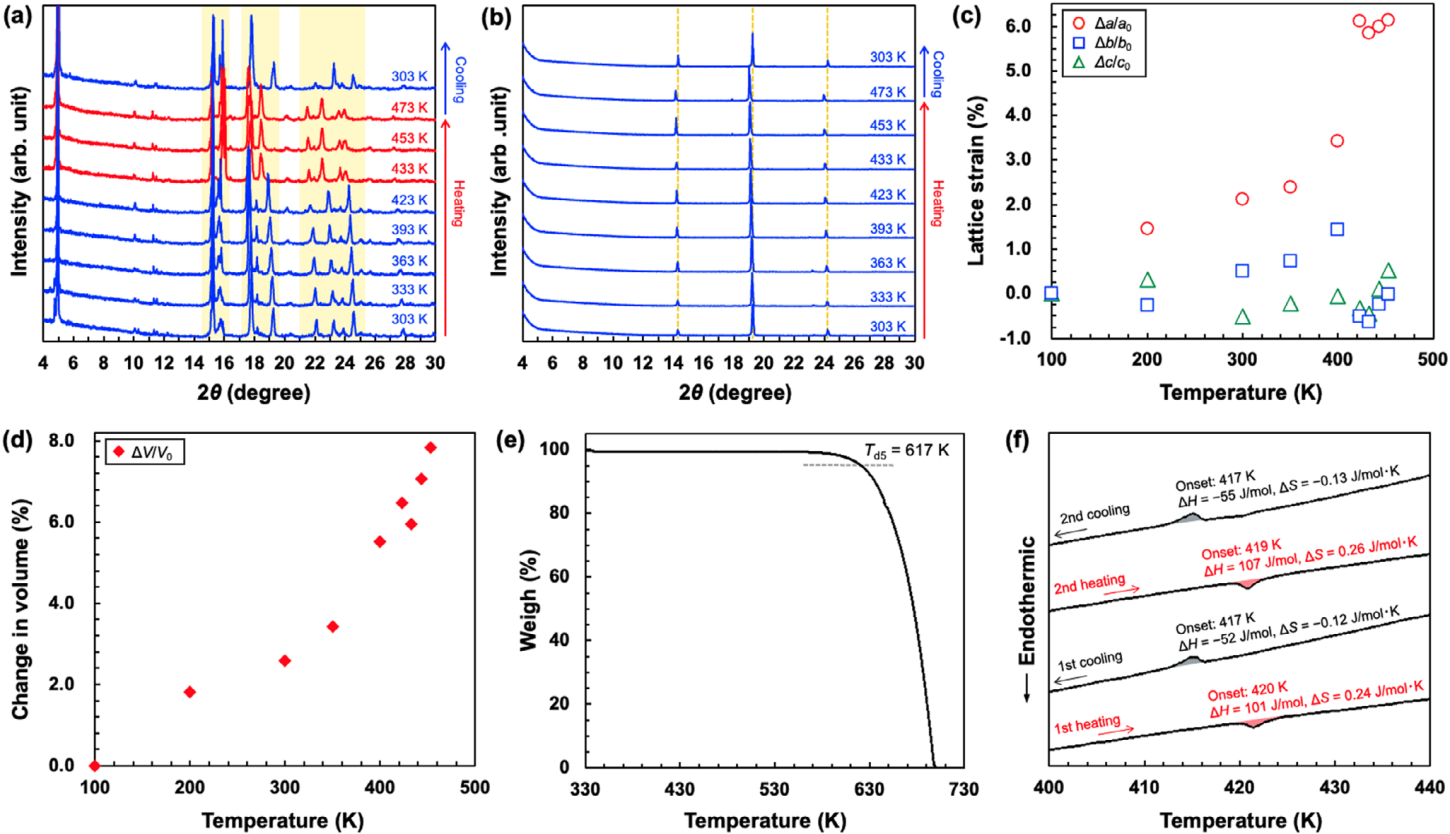
Reversible structural phase transition in single‐crystal samples. VT‐PXRD patterns of (a) **BBPA** and (b) **DPA** crystals, illustrating the structural evolution upon heating and cooling. Temperature dependence of the (c) lattice strain and (d) change in lattice volume of **BBPA** crystals calculated from unit‐cell parameters obtained by current‐cell refinement of VT‐SCXRD data. (e) TGA trace of **BBPA** crystals, with the 5% mass‐loss temperature (*T*_5d_) marked by a dashed line. (f) DSC profiles of **BBPA** crystals over two heating–cooling cycles, highlighting the reversible thermal phase transition.

To establish a direct structural relationship between the anisotropic lattice expansion and the phase transition, centroid‐to‐centroid distances of representative molecular pairs were compared between the 100 and 423 K crystal structures (Figures S8 and S9). The analysis revealed increased intermolecular separations along the directions corresponding to the crystallographic *a*‐ and *c*‐axes, consistent with the pronounced lattice expansion observed in Figure 4c. These distance changes reflect relative displacement between neighboring molecular layers and reorganization of the packing arrangement during the phase transition. As a consequence, the crystallographically distinct molecular environments become increasingly similar, ultimately leading to the reduction of *Z*' from 5 to 2.5.

Importantly, thermogravimetric analysis (TGA) confirmed the high thermal stability of **BBPA** crystals, with a 5% weight loss temperature (*T*_d5_) of 617 K (Figure 4e) [39, 40]. This result demonstrates that the thermally induced structural transitions occur well below the decomposition temperature. Differential scanning calorimetry (DSC) measurements further revealed reproducible endothermic and exothermic peaks during the heating and cooling cycles of **BBPA** single crystals, respectively (Figure 4f and Figure S58). As commonly observed for solid‐state phase transitions, a thermal hysteresis in the onset temperature was detected [41, 42, 43, 44]. Specifically, the transition temperatures were observed at 419–420 K upon heating and at 417 K upon cooling. Notably, these endothermic and exothermic features disappeared when the **BBPA** crystals were mechanically crushed prior to DSC measurements (Figures S50 and S59). The disappearance of the DSC anomaly after grinding suggests a significant reduction in the cooperativity of the phase transition (Figures S56 and S57). Consistent with this interpretation, Scherrer analysis revealed a substantial decrease in crystallite size following mechanical crushing, indicating partial disruption of long‐range crystalline order (Tables S8 and S9). Nevertheless, VT‐PXRD measurements confirmed that the structural transformation persists in the ground sample (Figures S53–S55). These results demonstrate that mechanical crushing weakens long‐range cooperative interactions and attenuates the thermal signature of the transition, while leaving the underlying structural transformation intact. The persistence of the structural transformation after grinding further suggests that the temperature‐responsive behavior is not restricted to high‐quality single crystals, although the macroscopic thermal response is strongly amplified by long‐range crystalline order. Taken together, these findings indicate that the phase transition in **BBPA** is enantiotropic in nature and originates from cooperative molecular rearrangements whose effects are amplified by long‐range structural order.

DSC measurements provide important thermodynamic insight into the phase transition in **BBPA** single crystals. At the transition temperature, where the Gibbs free energies of the low‐ and high‐temperature phases are equal (Δ*G* = 0), the transformation proceeds endothermically upon heating (Δ*H* > 0), reflecting the energetic cost associated with rearranging the crystal packing from the low‐temperature (*Z*' = 5) phase to the high‐temperature (*Z*' = 2.5) phase [45]. Despite this substantial crystallographic reorganization, the associated entropy gain is exceptionally small (Δ*S* = 0.24–0.26 J mol^−1^ K^−1^). According to Boltzmann's relation, Δ*S* = *R*ln(*W*_HT_/*W*_LT_), where *R* is the molar gas constant, and *W*_HT_ and *W*_LT_ denote the effective numbers of accessible microstates in the high‐ and low‐temperature phases, respectively. Using the experimentally determined entropy change, the effective microstate ratio was estimated to be only *W*_HT_/ *W*_LT_ = 1.029–1.032 (Table S10). In other words, the number of accessible microstates increases by less than 3% across the transition. Such a minor change in the number of accessible microstates is unexpected given the substantial crystallographic reorganization accompanying the reduction of *Z*' from 5 to 2.5. In conventional order–disorder or cooperative rotator transitions, a large entropy gain is typically associated with the emergence of additional configurational states [22]. By contrast, the near‐unity microstate ratio observed for **BBPA** suggests that the phase transition is not driven by the creation of substantial configurational disorder. Instead, the small entropy change indicates that the reduction of Z′ is primarily associated with dynamic averaging of pre‐existing local molecular environments rather than the generation of new configurational states, consistent with a cooperative symmetry‐recovery process.

This interpretation is further supported by variable‐temperature second‐harmonic generation (VT‐SHG) measurements, which directly probe the evolution of crystal symmetry (Figure S60). A clear SHG response is observed in the low‐temperature phase, independently confirming its non‐centrosymmetric nature. Upon heating, the second‐harmonic (SH) intensity decreases monotonically and reaches the background level above 423 K. Upon cooling back to 298 K, the SH signal reappears, confirming the reversible recovery of the non‐centrosymmetric phase. These results provide independent experimental evidence for a reversible phase transition between a non‐centrosymmetric low‐temperature phase and a centrosymmetric high‐temperature phase, consistent with the symmetry change determined by VT‐SCXRD. Notably, the gradual decrease in SH intensity upon heating indicates that the evolution of crystal symmetry is not confined to the transition temperature itself but develops progressively as the system approaches the phase transition.

This behavior is consistent with the structural and spectroscopic observations. VT‐SCXRD reveals a progressive increase in *tert*‐butyl disorder with temperature together with a substantial increase in atomic displacement parameters (ADPs) near the phase‐transition temperature (Figure S4), reflecting enhanced molecular mobility within the crystal lattice. Consistent with these crystallographic observations, VT‐Raman measurements show gradual broadening of vibrational bands upon heating (Figure 3c), further supporting the activation of molecular motions. Together, these crystallographic and spectroscopic results indicate that molecular motions become increasingly active well before the crystallographic phase transition occurs. Importantly, the increase in disorder and ADPs is interpreted as a physically meaningful manifestation of enhanced molecular mobility rather than a deterioration of the structural model. Rather than generating a large number of new configurational states at the phase‐transition temperature, these motions progressively average crystallographically distinct local environments. As this dynamic averaging becomes sufficiently extensive, the distinctions among independent molecular environments diminish, ultimately leading to restoration of higher crystallographic symmetry and a reduction of Z′ from 5 to 2.5 (Figures S6 and S7). This interpretation is further supported by the exceptionally small entropy change and near‐unity microstate ratio derived from DSC measurements. Taken together, these results suggest that the phase transition in **BBPA** is more appropriately described as a cooperative symmetry‐recovery process with only a minor entropic contribution, rather than a conventional order–disorder transition.

The progressive symmetry recovery described above is accompanied not only by crystallographic and spectroscopic changes but also by a pronounced macroscopic mechanical response. Notably, optical microscopy observations on a temperature‐controlled stage revealed that **BBPA** single crystals shift and jump from their original positions upon heating and cooling, respectively (Figure 5a,b and Movie S1). These macroscopic motions indicate that lattice strain accumulates within the crystals and is abruptly released near the transition temperature. Quantitative analysis further shows that the crystal length along the crystallographic *a*‐axis undergoes rapid anisotropic expansion upon heating in a narrow temperature window (419.3–419.6 K) (Figure 5d). Upon cooling, the thermally expanded crystals contract sharply near the transition temperature (411.6–411.9 K), reproducing the behavior observed by VT‐SCXRD and DSC measurements.

**FIGURE 5:**
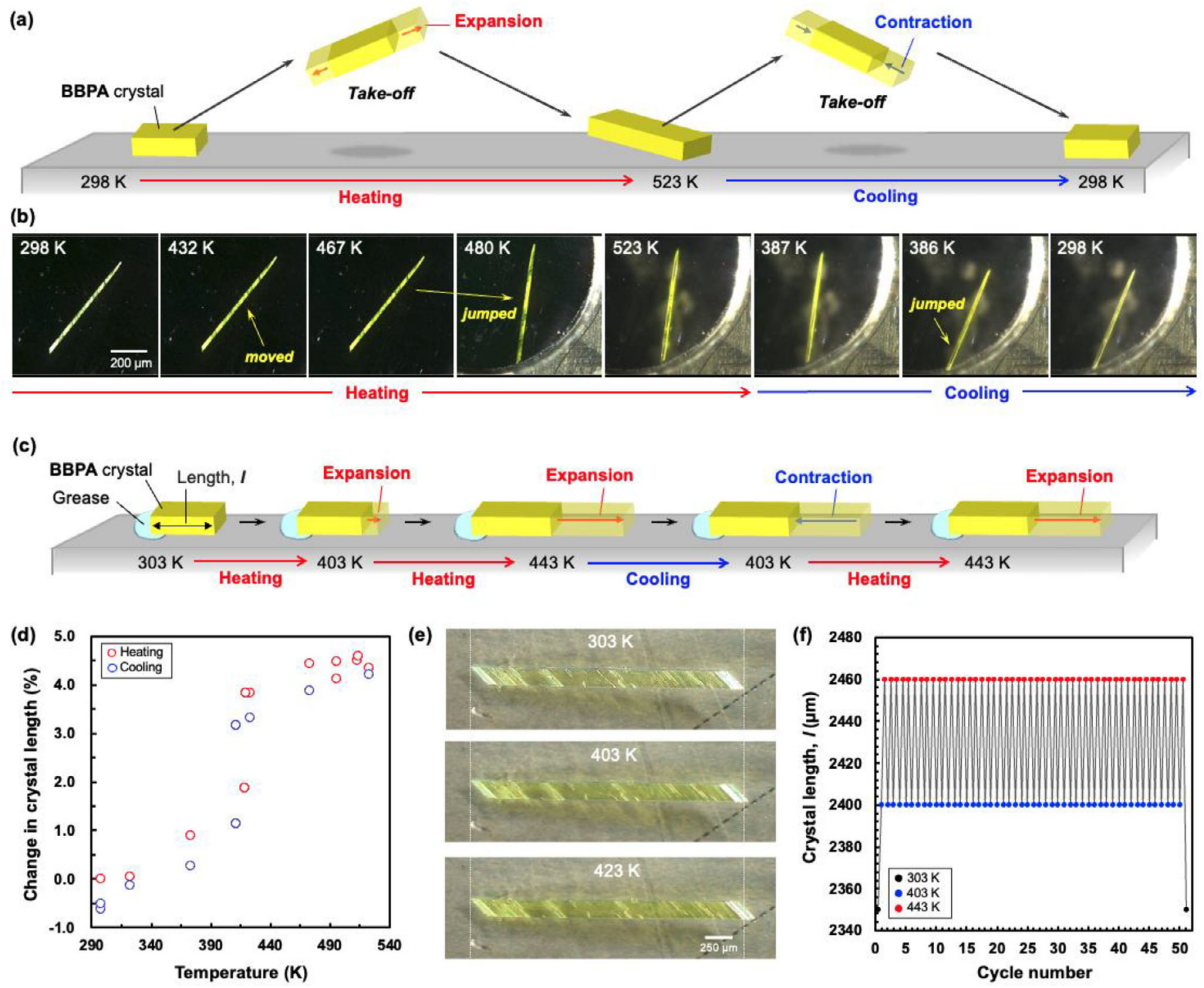
Thermally robust yet programmable macroscopic anisotropic expansion of **BBPA** single crystals. (a) Schematic illustrations and (b) optical micrographs of thermally driven crystal movement and jumping. (c) Schematic illustrations of thermal expansion and contraction of the crystal, showing elongation upon heating and shrinkage upon cooling. (d) Temperature‐dependent change in crystal length over successive heating–cooling cycles. (e) Optical micrographs showing the crystal length at 303, 403, and 423 K. (f) Reversible crystal‐length changes over multiple thermal cycles with no detectable degradation and complete recovery after each cycle.

**BBPA** crystals operate stably not only on rigid aluminum and glass substrates but also on compliant grease substrates (Figure 5c). As shown in Figure 5e, **BBPA** crystals fixed on grease exhibit both gradual and rapid anisotropic thermal expansion, while the abrupt temperature jump associated with the phase transition is effectively suppressed. Furthermore, the structural transitions of **BBPA** crystals proceed cooperatively during both heating (403–423 K) and cooling (423–403 K), as directly visualized by optical microscopy (Movies S2 and S3). Together with the thermal hysteresis (Figure 4f) and abrupt anisotropic lattice deformation (Figure 4c), the propagation of a distinct phase boundary indicates that the phase transition is martensitic in nature. This reversible martensitic‐type transition enables programmable anisotropic thermal expansion, which was reproducibly demonstrated over 50 thermal cycles between 403 and 443 K without detectable degradation (Figure 5f). Moreover, the crystals retained their macroscopic integrity after subsequent cooling to 303 K, demonstrating robust operation over the temperature range of 303–443 K.

Although the present study focuses on **BBPA** as a representative model system, we further examined whether the proposed molecular design principle can be extended beyond a single molecular example. Based on this design principle, namely the introduction of sterically demanding tert‐butyl‐substituted aryl groups to increase the number of accessible local molecular environments, we designed a structurally related anthracene derivative. As shown in Figure S62, this compound, despite possessing a different molecular framework, also crystallizes as a high‐Z′ structure (Z′ = 4 at 103–303 K). This result demonstrates that the formation of high‐Z′ crystal structures is not unique to BBPA and supports the broader applicability of the proposed molecular design principle to structurally related molecular systems.

## 3 Conclusion

We have further advanced our aryl‐substituted anthracene design strategy to create high‐*Z*′ anthracene crystals that undergo rapid and reversible structural phase transitions accompanied by temperature‐induced changes in *Z*′, based on a herringbone packing framework. The introduction of *tert*‐butyl‐substituted aryl groups at both ends of the molecular long axis in 2,6‐bis(4‐*tert*‐butylphenyl)anthracene generates asymmetric molecular conformations through multipoint intermolecular interactions, giving rise to five crystallographically independent molecules within a single asymmetric unit. The resulting high‐*Z*′ anthracene crystals exhibit cooperative and selective molecular motions upon temperature variation, leading to a cooperative symmetry‐recovery phase transition accompanied by a reduction of *Z*' from 5 to 2.5. Importantly, the rotational degrees of freedom introduced at the molecular level are spatially differentiated and hierarchically accumulated within the crystal lattice, thereby defining the initiation sites and propagation pathways of the phase transition. This hierarchical amplification of molecular‐scale structural freedom enables pronounced and rapid anisotropic thermal expansion over an exceptionally wide temperature range while simultaneously promoting dynamic averaging of crystallographically distinct local environments. These findings provide a molecular design principle in which high‐*Z*′ crystals emerge from the concerted interplay between rigid aromatic cores and localized peripheral rotational degrees of freedom, independent of a specific molecular scaffold. The concept of phase‐transition control based on high‐*Z*′ crystal design presented here provides not only a molecular design principle for developing thermally robust phase‐transition molecular crystals but also a foundation for exploring emergent solid‐state properties associated with phase transitions, including shape‐memory materials and intelligent organic semiconductors.

## Conflicts of Interest

The authors declare no conflicts of interest.

## Supporting information




**Supporting File 1**: smll75273‐sup‐0001‐SuppMat.pdf.

**Supporting File 2**: smll75273‐sup‐0002‐MovieS1.mp4.

**Supporting File 3**: smll75273‐sup‐0003‐MovieS2.mp4.

**Supporting File 4**: smll75273‐sup‐0004‐MovieS3.mp4.

## Data Availability

The data that support the findings of this study are available from the corresponding author upon reasonable request.
