# Supplementary material for: From Simplicity to Complexity: Substituent‐Controlled Z′ Multiplicity as a Design Principle for Thermally Robust Yet Programmable Anisotropic Expansion
Source: Small. 2026 Aug 18;22(59):e75273. doi: 10.1002/smll.75273 (PMC13690609; doi:10.1002/smll.75273)
Supplement: Supplementary file 1 [file SMLL-22-e75273-s004.pdf]

Supporting Information

From Simplicity to Complexity: Substituent-Controlled *Z′* Multiplicity as a Design Principle for Thermally Robust yet Programmable Anisotropic Expansion

Yuto Hino,[a] Takumi Matsuo,[a,b] Tomohiro Yamashita,[c] Hiroko Yokota,[c] Daiki Korenaga,[d] Tomohiro Seki,*[d] Shotaro Hayashi*[a,b]

[a] School of Engineering Science, Kochi University of Technology, 185 Miyanokuchi, Tosayamada, Kami, Kochi 782-8502, Japan.

[b] FOREST Center, Research Institute, Kochi University of Technology, 185 Miyanokuchi, Tosayamada, Kami, Kochi 782-8502, Japan.

[c] School of Materials and Chemical Technology, Institute of Science Tokyo, 4259 Nagatsuta-cho, Midori-ku, Yokohama, Kanagawa 226-8501, Japan

[d] Department of Chemistry, Faculty of Science, Shizuoka University, Shizuoka City, Shizuoka 422-8017, Japan

*To whom correspondence should be addressed. E-mail: seki.tomohiro@shizuoka.ac.jp (T. Seki); hayashi.shotaro@kochi-tech.ac.jp (S. Hayashi)

Contents

1. Experimental procedures 2
2. X-ray crystallographic observation 7
2.1 Crystallization 7
2.2 Substituent-controlled *Z*′ multiplicity in crystal structures 8
2.3 Visualizing temperature-dependent *tert*-butyl disorder in crystals 14
2.4 Symmetry-reorganized crystal structures after phase transition 24
2.5 Single-unit-cell reciprocal-space reconstruction 30
2.6 Variable-temperature X-ray diffraction reveals structural evolution 39
3. Intermolecular interaction analysis 46
4. Raman spectroscopic observation 48
4.1 Computational Raman analysis 48
4.2 Temperature-dependent Raman spectra 54
4.3 Peak shifts and FWHM analysis 55
5. Effect of grinding on the thermal phase transition 62
5.1 Mechanical grinding procedure 62
5.2 Evaluation of crystallinity and crystallite size 63
5.3 Variable-temperature X-ray diffraction reveals structural evolution 67
5.4 Effect of mechanical grinding on the DSC behavior 71
6. Temperature-dependent SHG response 73
7. Microscopic observation of thermal stability limits 74
8. Generality of the high-*Z*′ crystal-engineering strategy across anthracene scaffolds .75
9. Reported thermal phase transitions and *Z*′ values in single-component crystals 80
References 86
NMR spectra 88
High-resolution mass spectra 91
checkCIF/PLATON reports 92
PLATON/ADDSYM 146
Appendix 149

1. Experimental procedures

Materials: Solvents and reagents were purchased from Tokyo Chemical Industry Co., Sigma-Aldrich Co., LLC, Kanto Chemical Co., Inc., and FUJIFILM Wako Pure Chemical. Aluminum crimp and open pans ware purchased from Shimadzu Co. Purified water was obtained using an ion exchange method (Pureline WL200, Yamato Scientific). The ion exchange resin cartridge (CPC-P) had a capacity of 2 L and contained activated carbon. The water sample had a conductivity of 0.04 × 102 mol L^{-4} S/m.

Nuclear magnetic resonance (NMR): NMR spectra were recorded on a JEOL JNM-ECZ400S spectrometer operating at 400 MHz for ^{1}H and 100 MHz for ^{13}C at 333 K. Chemical shifts were referenced to the residual solvent signal of $CDCl_3$ (δ = 7.26 ppm for ^{1}H and δ = 77.16 ppm for ^{13}C). ^{1}H NMR data are reported as chemical shifts (δ, ppm), coupling constants (*J*, Hz), multiplicities (s = singlet, d = doublet, m = multiplet), and integration values. Spectra were processed using Delta software (JEOL).

High-resolution mass spectrometric (HRMS) analysis: High-resolution mass spectra were obtained on a Bruker compact QTOF instrument and processed using DataAnalysis 5.1 software. APCI-MS spectra were recorded via a direct insertion probe (Bruker). External calibration was carried out with Tuning Mix (Agilent Technologies) over the *m*/*z* range 100–3000.

Raman spectroscopy: Raman spectroscopy was performed using a HORIBA Jobin-Yvon LabRAM HR800 spectrometer with an Nd:YAG laser (λ = 532 nm) as the excitation source. The sample was irradiated over an area of approximately 30 μm through a D2 filter using a 50× objective lens. The sample temperature was controlled using a microscope-compatible heating/cooling stage (Linkam model 10002L) under a nitrogen atmosphere.

The experimental data were fitted using a Gaussian function:

$$y = y_0 + \frac{A\,\exp[-4\ln(2)\,(x - x_c)^2/w^2}{w\sqrt{\pi/4\ln(2)}}$$

where y_0 is the baseline offset, A is the integrated peak area, x_c is the peak center position, and w is the full width at half maximum (FWHM) of the peak. The goodness of the Gaussian fitting was evaluated using R-squared (R-Squ.) and adjusted R-squared (Adj. R-Squ.), with the latter accounting for the number of fitting parameters.

Differential scanning calorimetry (DSC): DSC measurements were performed using a Shimadzu DSC-60A Plus instrument. Samples (ca. 3 mg) were sealed in aluminum pans and heated and cooled at a rate of 10 K min^{-1} under a nitrogen atmosphere.

The enthalpy change (ΔH) associated with the phase transition was determined from the integrated area of the corresponding DSC peak using the instrument software. Assuming an enantiotropic phase transition, the Gibbs free energies of the low- and high-temperature phases are equal at the transition temperature (T_{onset}). Accordingly, the entropy change (ΔS) was calculated as

$$\Delta S = \frac{\Delta H}{T_{\mathrm{onset}}}$$

The Gibbs free energy difference (ΔG) between the low- and high-temperature phases was calculated according to

$$\Delta G(T) = \Delta H - T\Delta S$$

where T is the absolute temperature. The enantiotropic transition temperature was defined as the temperature at which the Gibbs free energy difference becomes zero ($\Delta G = 0$).[1]

For the heating process, Boltzmann's relation was employed:

$$S = R\ln W$$

where R is the molar gas constant and W is the effective number of accessible microstates. Accordingly, the entropy difference between the high-temperature (HT) and low-temperature (LT) phases can be expressed as

$$\Delta S = R\ln\left(\frac{W_{\mathrm{HT}}}{W_{\mathrm{LT}}}\right)$$

where W_{HT} and W_{LT} denote the effective numbers of accessible microstates in the HT and LT phases, respectively.

The effective microstate ratio ($W_{\mathrm{HT}}/W_{\mathrm{LT}}$) was therefore calculated using

$$\frac{W_{\mathrm{HT}}}{W_{\mathrm{LT}}} = \exp\left(\frac{\Delta S}{R}\right)$$

For the cooling process, the corresponding microstate ratio was calculated as

$$\frac{W_{\mathrm{LT}}}{W_{\mathrm{HT}}} = \exp\left(\frac{\Delta S}{R}\right)$$

Simultaneous thermogravimetry/Differential thermal analysis (TG-DTA): TG–DTA measurements were performed using an STA7200RV instrument (Hitachi High-Tech Co.). A few milligrams of **BBPA** crystals were placed in a platinum pan. Nitrogen was used as the purge gas at a flow rate of 100 mL min^{-1}, and the sample was heated at a rate of 10 K min^{-1} over a temperature range of 303 K–873 K. The thermal stability was evaluated by the temperature at 5% weight loss (T_{d5}).

Optical microscopy: Optical microscopy images and movies were acquired using a SHIMADZU STZ-171-TLED microscope equipped with a Moticam 1080BMH camera, as well as a KEYENCE VHX-S8000 digital microscope. A microscope-compatible heating/cooling stage (Linkam model 10002L) was used under a nitrogen atmosphere.

Powder X-ray diffraction (PXRD): PXRD measurements were performed using a Rigaku SmartLab SE diffractometer equipped with nickel-filtered CuKα radiation (1.5418 Å) operated at 40 kV and 40 mA. Diffraction data were collected in Bragg–Brentano geometry using a D/teX Ultra 250 detector over a 2θ range of 5º–35º with a step size of 0.01º and a scan rate of 5º min^{-1}. The incident slit was fixed at 1/2º, and a 2.5º Soller slit was employed. *In situ* variable-temperature PXRD (VT-PXRD) profiles were

also measured using the same instrument equipped with a heating unit and a platinum holder.

Crystallite-size analysis: Crystallite sizes were estimated from PXRD patterns using Rigaku SmartLab Studio II software. All resolved diffraction peaks were fitted using split pseudo-Voigt functions to determine the FWHM. Crystallite sizes were calculated according to the Scherrer equation. The reported crystallite size corresponds to the average value obtained from all resolved diffraction peaks and is used for comparative purposes between the single-crystal sample and the ground sample.

Second harmonic generation (SHG): To probe changes in crystal symmetry and polarity, SHG measurements were performed using a continuous-wave laser with a fundamental wavelength of 1050 nm. Temperature-dependent measurements were conducted using a Linkam heating/cooling stage, enabling controlled variation of the sample temperature.

Single crystal X-ray diffraction (SCXRD): SCXRD experiments for **BBPA** and **DPA** crystals were performed using a Rigaku XtaLAB Synergy-S/Mo system (λ = 0.71073 Å) equipped with a HyPix-600HE photon-counting detector at Kochi University of Technology. Each single crystal was mounted on the tip of a polyimide loop using silicone grease and submitted for measurement. The reduction and correction of diffraction data proceeded with the CrysAlisPro program. The diffraction experiments were processed using the Olex2 software package.[2] The structure was solved with the ShelXT structure solution program using Intrinsic Phasing.[3] The structure was refined with the ShelXL refinement package using least-squares minimization.[4] Any alerts in the CIF should be carefully addressed with the guidance of a crystallographer when necessary. The occupancies of the molecules were refined based on electron densities using the free variables. The crystallographic refinement process includes the handling of disordered molecules. All non-hydrogen atoms were refined with anisotropic displacement parameters. Hydrogen atoms were placed in idealized positions and refined as rigid atoms with the relative isotropic displacement parameters. Their omission does not significantly impact the final data. The number of crystallographically independent molecules in the asymmetric unit (Z') was determined and analyzed based on structure solution and refinement results. Some Alerts A and B notifications were found in the validation program CheckCIF. Those alerts are mainly derived from short intermolecular contacts and unusual temperature factors involving atoms on disordered molecules. Comments regarding these alerts are included in the CIF and Supporting Information using the validation response form (vrf).

In situ variable-temperature single-crystal X-ray diffraction (VT-SCXRD) experiments for **BBPA** crystals were performed at the BL02B1 beamline of SPring-8 using a quarter-circle diffractometer (Rigaku Co., Japan), with diffraction data collected using a two-dimensional semiconductor detector, PILATUS3 X CdTe (DECTRIS Ltd., Switzerland).[5] The diffraction experiments were synchrotron radiation (λ = 0.4133 Å). **BBPA** crystal was maintained at temperatures between 100 K and 453 K during data collection. The intensities of the Bragg reflections were collected using the CrysAlisPro software. In measurements performed with high-energy synchrotron radiation, X-ray

absorption by organic materials is intrinsically minimal. Accordingly, absorption effects are negligible without consideration of crystal size, and no absorption correction was applied.

Raman spectral calculations: Raman spectral calculations were performed using the Gaussian 16 program package.[6,7] Raman activities were calculated at the ωB97X-D/6-31G(d,p) level of theory without symmetry constraints (NoSymm), using the fixed molecular geometries extracted directly from the crystallographic structures. Harmonic vibrational frequency calculations, including Raman activities, were carried out using the Freq=Raman keyword.

Molecular geometries used for Raman spectral simulations were extracted from the single-crystal X-ray structure (CCDC 2532253) by isolating each crystallographically independent molecule from the asymmetric unit. In the presence of crystallographic disorder, a representative ordered conformation was selected by removing minor disordered components. For the Z' = 5 crystal, all five crystallographically independent molecules were treated separately.

The Raman spectra calculated for the five independent molecules were averaged point-by-point on a common Raman-shift grid with a step size of 1 cm^{-1} to obtain a representative spectrum for the Z' = 5 crystal (Figure S32). Normal-mode displacement vectors were visualized using GaussView based on the fixed molecular geometries. Displacement vectors are scaled for clarity. The simulated Raman spectra were used to assist vibrational-mode assignments and to enable qualitative comparison with experimental spectra rather than quantitative reproduction of absolute Raman intensities.

Dimer interaction-energy calculations: Interaction-energy calculations were performed using the Condor computational chemistry package (Rowan Scientific, version 8bd1193).[32] Single-point electronic energies were calculated with the GPU4PySCF engine at the ωB97M-D3BJ/vDZP level of theory.[33,34,35] Representative molecular dimers were extracted directly from the crystallographic structures determined at 100 K and 423 K. No geometry optimization was performed, and the crystallographic geometries were retained for all calculations. The interaction energy (E_{int}) was defined as

$$E_{\mathrm{int}} = E_{\mathrm{dimer}} - \sum E_{\mathrm{isolated}}$$

where E_{dimer} is the electronic energy of the dimer and $\Sigma E_{\mathrm{isolated}}$ is the sum of the electronic energies of the two corresponding isolated molecules calculated at the same level of theory. The calculated interaction energies were used to compare the relative stabilization of representative intermolecular contacts in the low- and high-temperature phases.

Mechanical grinding: Mechanical grinding was performed using a SHAKE MAN 3 (Bio Medical Science Inc.). Single-crystal samples of **BBPA** were placed in a 2 mL tube containing stainless-steel balls (5 mm diameter) and ground at 4000 rpm for 180 s. The resulting ground sample was subsequently used for VT-PXRD and DSC measurements.

2,6-Bis(4-*tert*-butylphenyl)anthracene (BBPA): 2,6-Dibromoanthracene (100 mg, 0.30 mmol), $Pd(PPh_3)_4$ (34 mg, 30 μmol), and 4-*tert*-butylphenylboronic acid (213 mg, 1.2

mmol) were added to a two-necked flask with a magnetic stirrer bar. Then, the flask was filled with argon. Toluene (5 mL) and K_2CO_3 aq. (2 mol L^{-1}, 2 mL) were added, and the resulting mixture was stirred at 110 ºC for 48 h. The reaction mixture was quenched with brine, allowing the organic layer to separate. The aqueous layer was extracted with chloroform. The combined organic solution was washed with brine, dried over $MgSO_4$, filtered, and concentrated by rotary evaporation. The crude product was dissolved in chloroform recrystallized to obtain the **BBPA** as pale-yellow powder (78 mg, 59%).

Physical data; ^{1}H NMR (400 MHz, $CDCl_3$, ppm) δ=8.64 (s, 2H), 8.20 (s, 2H), 8.07 (d, 2H, J = 8.4 Hz), 7.77 (d, 2H, J = 8.8 Hz), 7.73 (d, 4H, J = 8.4 Hz), 7.54 (d, 4H, J = 8.4 Hz), 1.42 (s, 18H); ^{13}C NMR (100 MHz, $CDCl_3$, ppm) δ=150.8 (*C*), 138.4 (*C*), 137.9 (*C*), 132.4 (*C*), 131.6 (*C*), 128.8 (*C*H), 127.2 (*C*H), 126.4 (*C*H), 126.0 (*C*H), 125.9 (*C*H), 125.7 (*C*H), 34.80 (*C*), 31.59 (*C*H_3); HRMS (APCI/TOF) *m*/*z* calcd for $C_{34}H_{34}+H^+$: 443.2733 $[M+H]^+$; found: 443.2752.

2,6-Diphenylanthracene (DPA): 2,6-Dibromoanthracene (100 mg, 0.30 mmol), $Pd(PPh_3)_4$ (17 mg, 15 μmol), and phenylboronic acid (146 mg, 1.2 mmol) were added to a two-necked flask with a magnetic stirrer bar. Then, the flask was filled with argon. Toluene (5 mL) and K_2CO_3 aq. (2 mol L^{-1}, 2 mL) were added, and the resulting mixture was stirred at 110 ºC for 48 h. The reaction mixture was quenched with brine, allowing the organic layer to separate. The aqueous layer was extracted with ethyl acetate. The combined organic solution was washed with brine, dried over $MgSO_4$, filtered, and concentrated by rotary evaporation. The solid was purified by soxhlet extraction (toluene). The crude product was dissolved in chloroform recrystallized to obtain the **DPA** as yellow powder (50 mg, 50%).

Physical data; ^{1}H NMR (400 MHz, $CDCl_3$, ppm) δ=8.49 (s, 2H), 8.15 (d, 2H), 8.09 (d, 2H, J = 8.4 Hz), 7.80–7.76 (m, 6H), 7.53–7.49 (m, 4H), 7.42–7.37 (m, 2H). The ^{1}H NMR spectrum is in accordance with the literature.[8]

2,6-Bis(4-*tert*-butylethynyl)anthracene (BBEA): 2,6-Dibromoanthracene (50 mg, 0.15 mmol), $Pd(PPh_3)_2Cl_2$ (11 mg, 15 μmol), and CuI (3 mg, 15 μmol) were added to a two-necked flask with a magnetic stirrer bar. Then, the flask was filled with argon. 3,3-Dimethyl-1-butyne (246 mg, 3.0 mmol) and triethylamine (3 mL) were added, and the resulting mixture was stirred at 80 ºC for 66 h. The reaction mixture was purified by column chromatography on silica gel (chloroform). The crude product was dissolved in chloroform recrystallized to obtain the **BBEA** as colorless powder (38 mg, 75%).

Physical data; ^{1}H NMR (400 MHz, $CDCl_3$, ppm) δ=8.25 (s, 2H), 8.20 (s, 2H), 7.87 (d, 2H, J = 8.8 Hz), 7.40 (dd, 2H, J = 8.8 Hz, 2.0 Hz), 1.38 (s, 18H); ^{13}C NMR (100 MHz, $CDCl_3$, ppm) δ=131.6 (*C*), 131.3 (*C*H), 130.9 (*C*), 128.6 (*C*H), 128.1 (*C*H), 125.9 (*C*H), 121.2 (*C*), 99.90 (*C*), 79.75 (*C*), 31.21 (*C*H_3), 28.25 (*C*).

2. X-ray crystallographic observation

2.1 Crystallization

BBPA crystals were prepared by a slow cooling method. A solution of **BBPA** (2 mg) in chloroform (2 mL) was placed in a screw-cap test tube and allowed to cool slowly to room temperature. Pale-yellow plate-like single crystals of **BBPA** formed on the inner walls of the test tube. **DPA** crystals were prepared in the same manner. A solution of **DPA** (1 mg) in chloroform (2 mL) was placed in a screw-cap test tube and allowed to cool slowly to room temperature. Yellow plate-like single crystals of **DPA** formed on the inner walls of the test tube. One of the crystals was removed from the solution, mounted on a single crystal X-ray diffractometer using a nylon loop, and used for diffraction data collection.

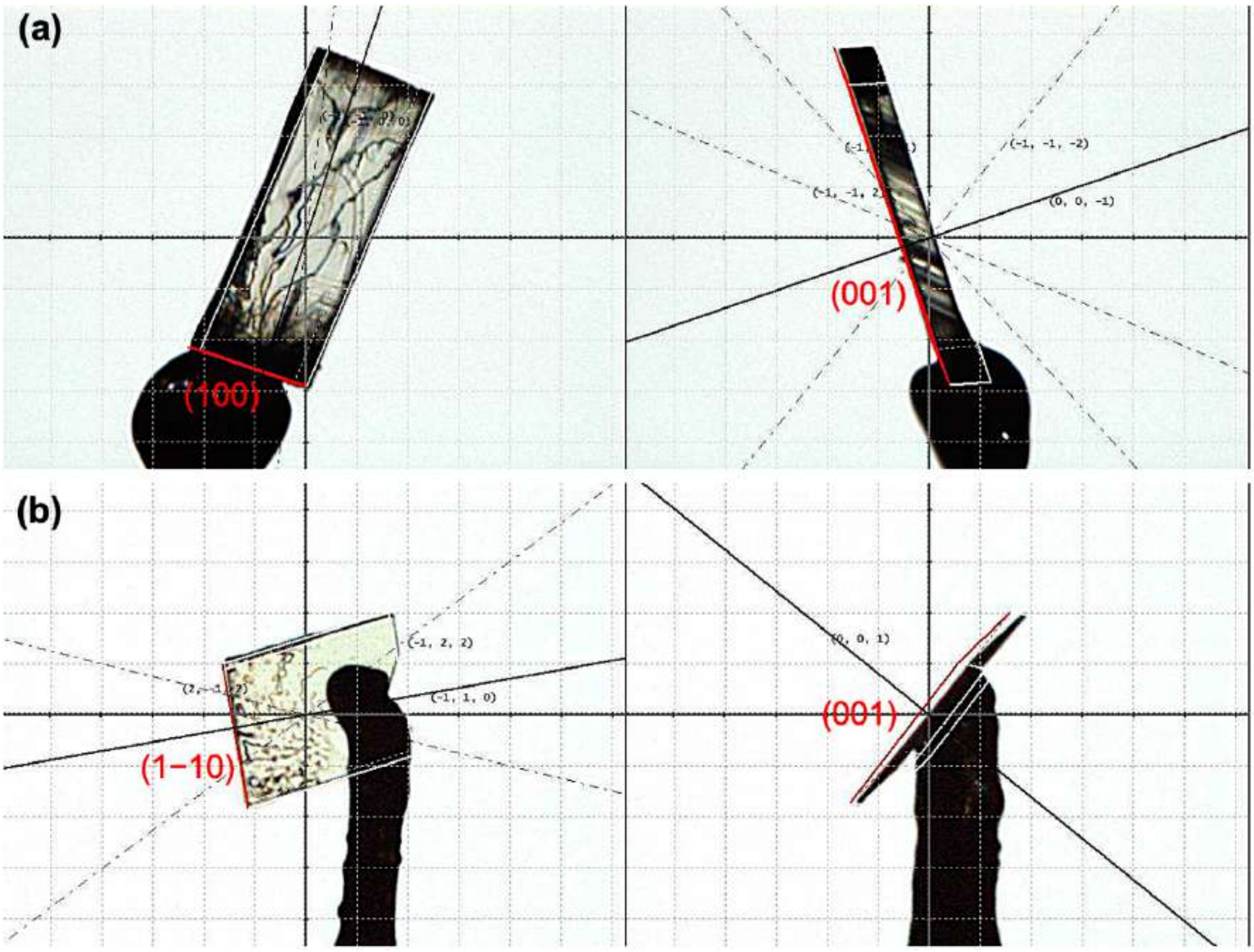

Figure S1. Indexing of SCXRD images and the relationship between crystal morphology and lattice planes for (a) **BBPA** and (b) **DPA** crystals. The red boxes and red labels indicate the indexed crystallographic planes.

2.2 Substituent-controlled *Z′* multiplicity in crystal structures

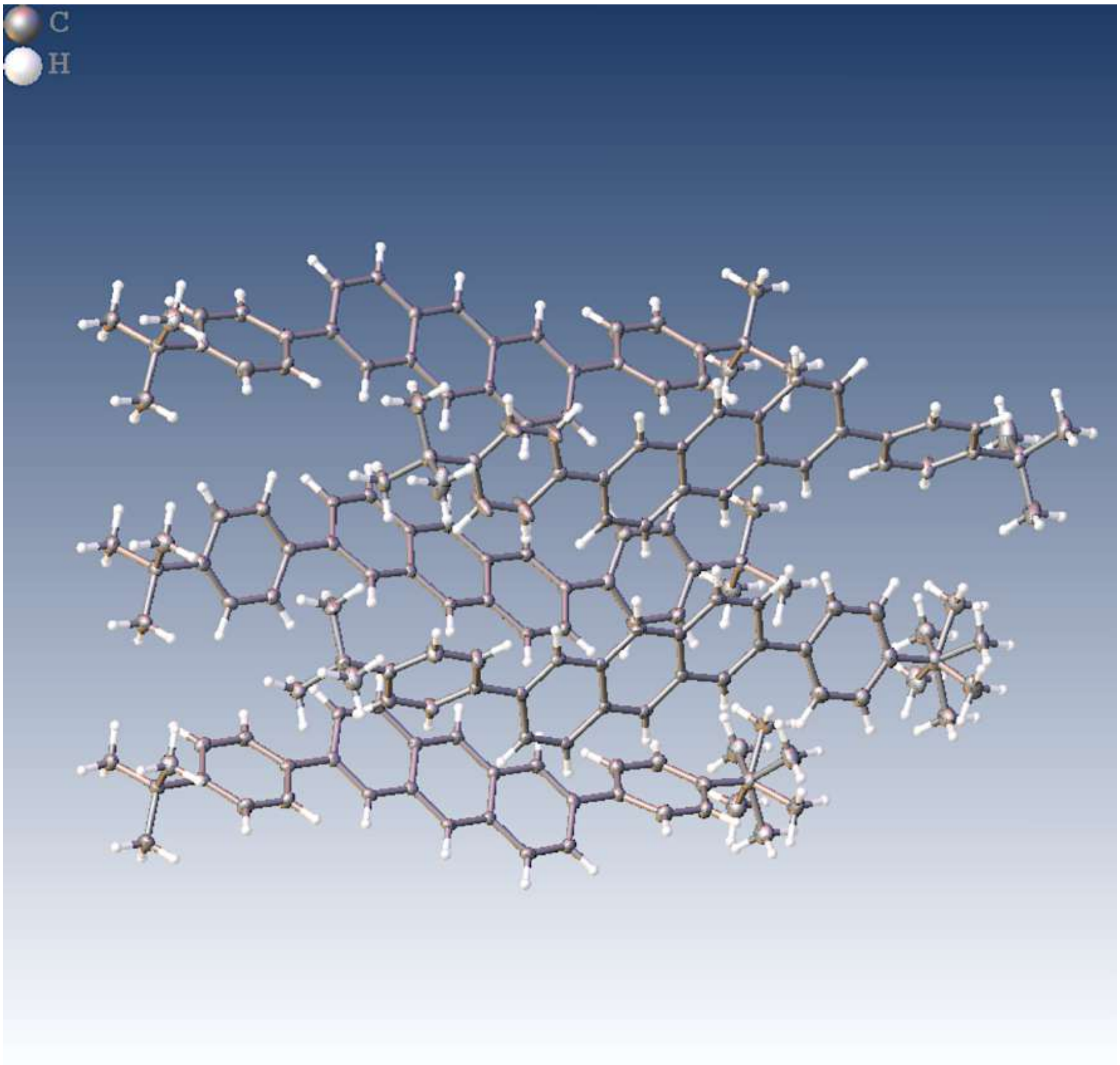

Figure S2. Crystal structure of **BBPA** determined at 103 K (CCDC 2465106). ORTEP drawing shown with 50% probability anisotropic displacement parameters. *Z′* = 5.

Table S1. Crystal Data and Structure Refinement Parameters for the **BBPA** at 103 K

Identification code	exp_256	
CCDC deposit number	2465106	
Empirical formula	$C_{34}H_{34}$	
Formula weight	442.61	
Wavelength	0.71073 Å	
Crystal system	monoclinic	
Space group	$P2_1$	
Unit cell dimensions	a = 12.6713(3) Å	α = 90º
	b = 28.9509(5) Å	β = 105.554(3)º
	c = 17.6785(5) Å	γ = 90º
Volume	6247.8(3) Å^3	
Z	10	
Density (calculated)	1.176 g/cm^3	
Absorption coefficient	0.066 mm^{-1}	
F(000)	2380.0	
Crystal size	0.982 × 0.223 × 0.171 mm^3	
2θ range for data collection	3.546 to 52.742º	
Index ranges	-15≦h≦13, -36≦k≦36, -22≦l≦18	
Reflections collected	47054	
Independent reflections	23997 [R(int) = 0.0268]	
Data / restraints / parameters	23997 / 7 / 1623	
Goodness-of-fit on F^2	1.023	
Final R indices [I>2σ(I)]	R_1 = 0.0495, wR_2 = 0.1175	
R indices (all data)	R_1 = 0.0704, wR_2 = 0.1302	
Largest diff. peak and hole	0.30/-0.21 e.Å^{-3}	
Flack parameter	0.6(10)	

A- and B-level alerts in the Checkcif file:

(There is no A-level alerts)

B-Level alerts

"Reflection # Likely Affected by the Beamstop ... 2 Check"

This alert arises because a small number of reflections occur at very low diffraction angles, close to the direct beam, and may therefore be partially shadowed by the beamstop. These reflections are few in number and do not affect the refined structural parameters or the reliability of the overall structural model.

"Number of (*I*obs-*I*calc)/Sigma(W) > 10 Outliers .. 2 Check"

This alert arises because a very small number of very low-angle reflections close to the direct beam show unusually large differences between observed and calculated intensities. These outliers are difficult to measure accurately due to partial shadowing by the beamstop and experimental limitations in the lowest-angle region, and they do not affect the refined structural parameters or the reliability of the structure.

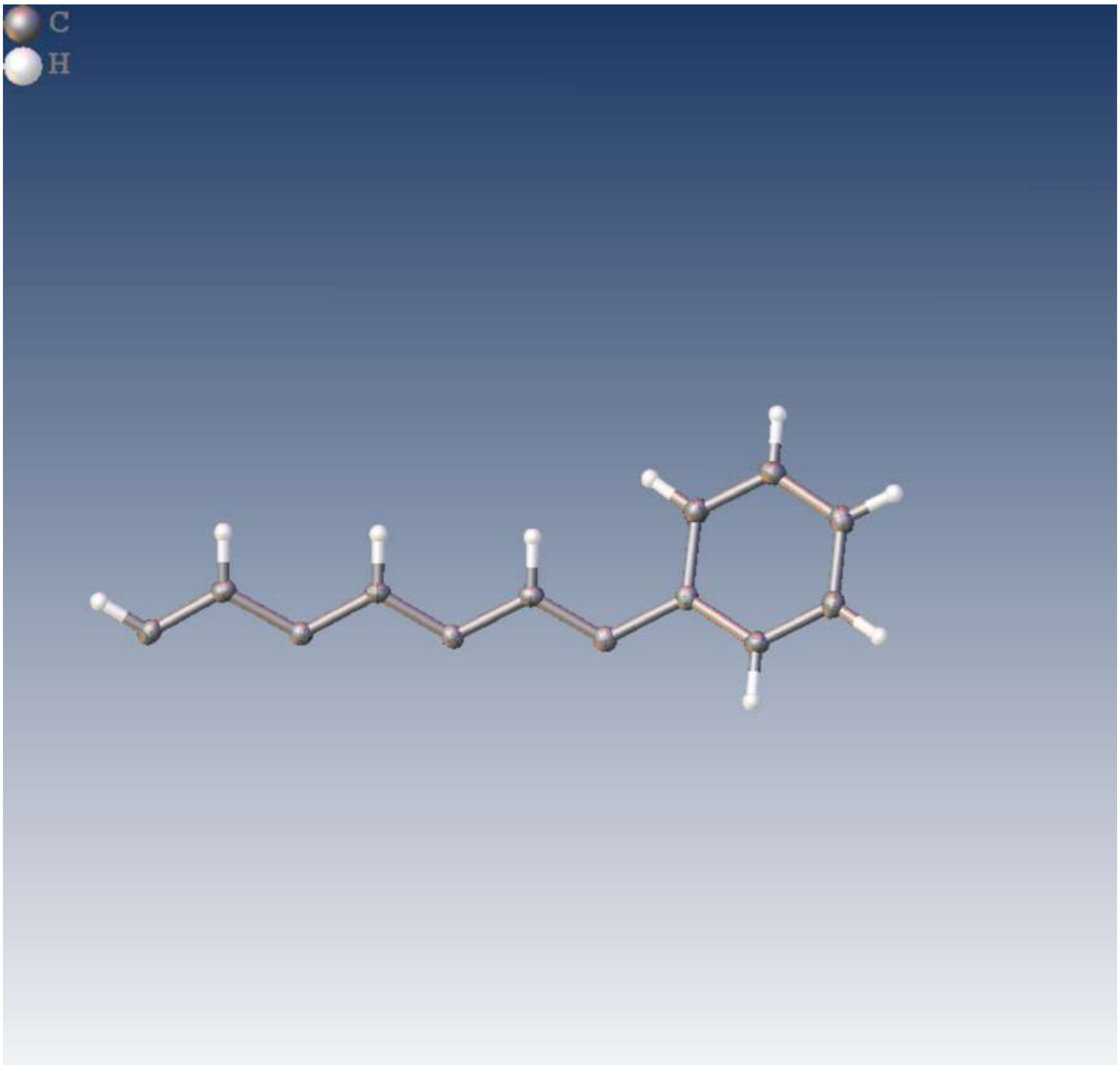

Figure S3. Crystal structure of **DPA** determined at 103 K (CCDC 2465111). ORTEP drawing shown with 50% probability anisotropic displacement parameters. *Z*′ = 0.5.

Table S2. Crystal Data and Structure Refinement Parameters for the **DPA** at 103 K

Identification code	exp_1015	
CCDC deposit number	2465111	
Empirical formula	$C_{26}H_{18}$	
Formula weight	330.40	
Wavelength	0.71073 Å	
Crystal system	monoclinic	
Space group	$P2_1/c$	
Unit cell dimensions	a = 17.9884(12) Å	α = 90º
	b = 7.3638(3) Å	β = 90.590(7)º
	c = 6.2625(4) Å	γ = 90º
Volume	829.51(8) $Å^3$	
Z	2	
Density (calculated)	1.323 g/cm^3	
Absorption coefficient	0.075 mm^{-1}	
F(000)	348.0	
Crystal size	0.44 × 0.338 × 0.052 mm^3	
2θ range for data collection	4.528 to 60.078º	
Index ranges	$-22 \leqq h \leqq 17$, $-10 \leqq k \leqq 8$, $-8 \leqq l \leqq 7$	
Reflections collected	3548	
Independent reflections	1581 [R(int) = 0.0221]	
Data / restraints / parameters	1581 / 0 / 118	
Goodness-of-fit on F^2	1.062	
Final R indices [I>2σ(I)]	R_1 = 0.0409, wR_2 = 0.1013	
R indices (all data)	R_1 = 0.0592, wR_2 = 0.1116	
Largest diff. peak and hole	0.19/–0.29 e.$Å^{-3}$	

A- and B-level alerts in the Checkcif file:

A-Level alerts

"Diffrn_measured_fraction_theta_full value Low. 0.841 Why?"

This alert arises because a fraction of high-angle reflections could not be reliably measured due to the limited diffracting volume of the thin crystal (0.052 mm thickness), resulting in a reduced θ_full completeness (0.841). These missing reflections do not affect the refined structural parameters or the conclusions.

B-Level alerts

"Missing FCF Refl Between Thmin & STh/L= 0.600"

This alert arises because some reflections between θ_min and STh/L = 0.600 could not be reliably integrated due to weak intensities at higher diffraction angles, arising from the limited diffracting volume of the thin crystal. These missing reflections do not influence the refined structure or the conclusions.

2.3 Visualizing temperature-dependent *tert*-butyl disorder in crystals

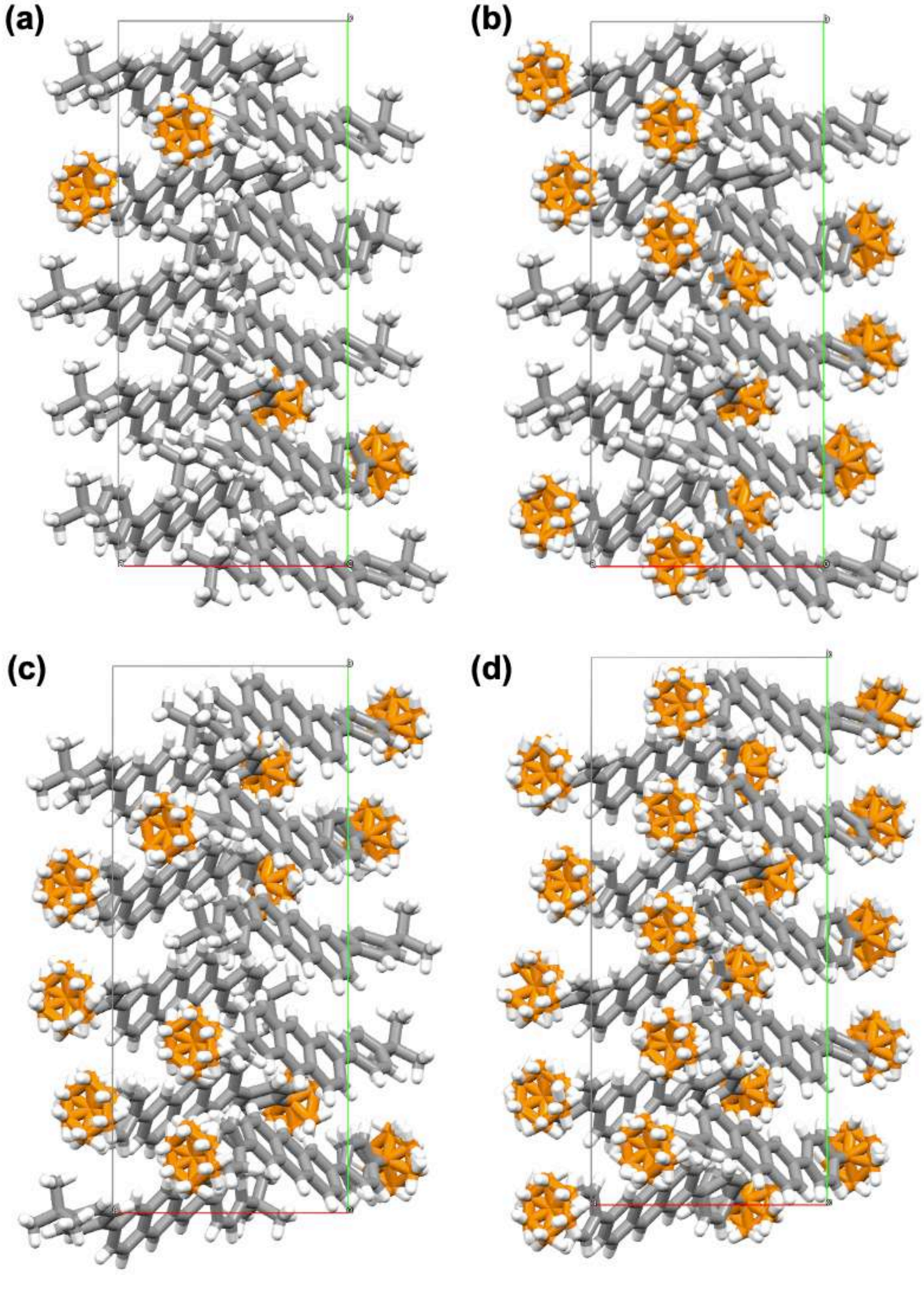

Figure S4. Temperature-controlled disorder ratios (DRs) of *tert*-butyl groups in crystal **1**: (a) 100 K (CCDC 2532253), DR = 2/10; (b) 200 K (CCDC 2532254), DR = 6/10; (c) 300 K (CCDC 2465110), DR = 6/10; and (d) 400 K (CCDC 2532310), DR = 10/10. The molecules in the unit cell are shown using capped-stick models. Ordered carbon atoms are shown in gray, disordered carbon atoms in orange, and hydrogen atoms in white.

Table S3. Crystal Data and Structure Refinement Parameters for the **BBPA** at 100 K

Identification code	exp_100	
CCDC deposit number	2532253	
Empirical formula	$C_{34}H_{34}$	
Formula weight	442.61	
Wavelength	0.4133 Å	
Crystal system	monoclinic	
Space group	$P2_1$	
Unit cell dimensions	a = 12.72020(10) Å	α = 90º
	b = 29.0778(2) Å	β=105.5870(10)º
	c = 17.70500(10) Å	γ = 90º
Volume	6307.81(8) Å^3	
Z	10	
Density (calculated)	1.165 g/cm^3	
Absorption coefficient	0.031 mm^{-1}	
F(000)	2380.0	
2θ range for data collection	2.926 to 43.546º	
Index ranges	$-22 \leqq h \leqq 22$, $-51 \leqq k \leqq 51$, $-31 \leqq l \leqq 31$	
Reflections collected	335839	
Independent reflections	64810 [R(int) = 0.0544]	
Data / restraints / parameters	64810 / 7 / 1623	
Goodness-of-fit on F^2	1.039	
Final R indices [I>2σ(I)]	R_1 = 0.0404, wR_2 = 0.1134	
R indices (all data)	R_1 = 0.0440, wR_2 = 0.1157	
Largest diff. peak and hole	0.44/-0.63 e.Å^{-3}	
Flack parameter	0.6(10)	

A- and B-level alerts in the Checkcif file:

(There is no A-level alerts)

B-Level alerts

"The minimum difference density is < −0.1*ZMAX*1.00"

This alert arises because the minimum residual electron density slightly exceeds the test threshold but is small in magnitude and does not indicate any deficiencies in the structural model.

"Large Reported Min. (Negative) Residual Density −0.63 eA^{-3}"

This alert arises because the minimum negative residual electron density marginally exceeds the guideline threshold. Its magnitude is small and does not indicate any deficiencies in the structural model.

"Missing FCF Reflection(s) Below Theta(Min)[Deg]= 1.46 Note"

This alert arises because some very low-angle reflections below $\theta = 1.46^{o}$ were excluded due to limited reliability and do not influence the refined structure.

Table S4. Crystal Data and Structure Refinement Parameters for the **BBPA** at 200 K

Identification code	exp_200	
CCDC deposit number	2532254	
Empirical formula	$C_{34}H_{34}$	
Formula weight	442.61	
Wavelength	0.4133 Å	
Crystal system	monoclinic	
Space group	$P2_1$	
Unit cell dimensions	a = 12.86790(10) Å	α = 90º
	b = 29.0348(2) Å	β=104.9670(10)º
	c = 17.76910(10) Å	γ = 90º
Volume	6413.61(8) Å^3	
Z	10	
Density (calculated)	1.146 g/cm^3	
Absorption coefficient	0.030 mm^{-1}	
F(000)	2380.0	
2θ range for data collection	2.92 to 43.51º	
Index ranges	$-22 \leqq h \leqq 22$, $-51 \leqq k \leqq 51$, $-31 \leqq l \leqq 31$	
Reflections collected	342394	
Independent reflections	66005 [R(int) = 0.0942]	
Data / restraints / parameters	66005 / 19 / 1747	
Goodness-of-fit on F^2	1.024	
Final R indices [$I>2\sigma(I)$]	R_1 = 0.0560, wR_2 = 0.1559	
R indices (all data)	R_1 = 0.0714, wR_2 = 0.1670	
Largest diff. peak and hole	0.51/-0.42 e.Å^{-3}	
Flack parameter	1.3(10)	

A- and B-level alerts in the Checkcif file:

(There is no A-level alerts)

B-Level alerts
"Missing FCF Reflection(s) Below Theta(Min)[Deg]= 1.46 Note"
This alert arises because some very low-angle reflections below $\theta = 1.46^{o}$ were excluded due to limited reliability and do not influence the refined structure.

"Number of (Iobs-Icalc)/Sigma(W) > 10 Outliers .. 4 Check"
This alert arises because a small number of intensity outliers are present and do not influence the refined structure or the conclusions.

Table S5. Crystal Data and Structure Refinement Parameters for the **BBPA** at 300 K

Identification code	exp_300	
CCDC deposit number	2465110	
Empirical formula	$C_{34}H_{34}$	
Formula weight	442.61	
Wavelength	0.4133 Å	
Crystal system	monoclinic	
Space group	$P2_1$	
Unit cell dimensions	a = 12.9889(2) Å	α = 90º
	b = 29.2286(3) Å	β=104.4890(10)º
	c = 17.7849(2) Å	γ = 90º
Volume	6537.25(14) Å^3	
Z	10	
Density (calculated)	1.124 g/cm^3	
Absorption coefficient	0.030 mm^{-1}	
F(000)	2380.0	
2θ range for data collection	3.03 to 43.574º	
Index ranges	-23≦h≦23, -51≦k≦51, -31≦l≦31	
Reflections collected	347898	
Independent reflections	67239 [R(int) = 0.0706]	
Data / restraints / parameters	67239 / 49 / 1858	
Goodness-of-fit on F^2	0.987	
Final R indices [I>2σ(I)]	R_1 = 0.0649, wR_2 = 0.1856	
R indices (all data)	R_1 = 0.1151, wR_2 = 0.2170	
Largest diff. peak and hole	0.32/–0.21 e.Å^{-3}	
Flack parameter	6.8(10)	

A- and B-level alerts in the Checkcif file:

(There is no A-level alerts)

B-Level alerts
"Atom C20 has ADP max/min Ratio.....4.1 prolat"
This alert arises because atom C20 exhibits a strongly anisotropic displacement ellipsoid (oblate) with a relatively large ADP max/min ratio (4.1). This behavior is attributed to anisotropic thermal motion and local flexibility of the molecular fragment containing C20 under the measurement conditions, which leads to a preferred direction of atomic displacement. The displacement parameters remain physically reasonable and do not indicate any misassignment or missing disorder model; the refinement is stable and the structural model is reliable.

"Large Hirshfeld Difference C02T --C9 . 0.28 Ang."
This alert arises because the Hirshfeld rigid-bond test indicates a slightly larger difference for the C02T --C9 bond at 300 K. This bond involves atoms with relatively large atomic displacement parameters, and the observed difference is attributed to enhanced thermal motion at room temperature. The deviation is modest in magnitude and does not indicate any disorder, misassignment, or deficiencies in the structural model.

"Low 'MainMol' Ueq as Compared to Neighbors of C02T Check"
This alert arises because atom C02T exhibits a slightly lower Ueq than its neighbors due to local rigidity and anisotropic thermal motion. This does not affect the validity of the refined structure.

"Missing FCF Reflection(s) Below Theta(Min)[Deg]= 1.51 Note"
This alert arises because some very low-angle reflections below $\theta = 1.51^{o}$ were excluded due to limited reliability and do not influence the refined structure.

Table S6. Crystal Data and Structure Refinement Parameters for the **BBPA** at 400 K

Identification code	exp_400	
CCDC deposit number	2532310	
Empirical formula	$C_{34}H_{34}$	
Formula weight	442.61	
Wavelength	0.4133 Å	
Crystal system	monoclinic	
Space group	$P2_1$	
Unit cell dimensions	a = 13.1496(3) Å	α = 90º
	b = 29.4925(5) Å	β = 103.947(2)º
	c = 17.7763(4) Å	γ = 90º
Volume	6690.7(2) $Å^3$	
Z	10	
Density (calculated)	1.099 g/cm^3	
Absorption coefficient	0.029 mm^{-1}	
F(000)	2380.0	
2θ range for data collection	3.022 to 43.576º	
Index ranges	$-23 \leqq h \leqq 23$, $-51 \leqq k \leqq 52$, $-31 \leqq l \leqq 31$	
Reflections collected	354081	
Independent reflections	68620 [R(int) = 0.0980]	
Data / restraints / parameters	68620 / 42 / 1908	
Goodness-of-fit on F^2	0.920	
Final R indices [I>2σ(I)]	R_1 = 0.0747, wR_2 = 0.2032	
R indices (all data)	R_1 = 0.2122, wR_2 = 0.2783	
Largest diff. peak and hole	0.21/-0.16 e.$Å^{-3}$	
Flack parameter	–0.6(10)	

A- and B-level alerts in the Checkcif file:

A-Level alerts

"The absolute value of parameter shift to su ratio > 0.20"

This alert arises because the final refinement cycle shows a relatively large shift-to-standard-uncertainty ratio, which is attributed to the high measurement temperature (400 K) and the presence of large atomic displacement parameters. The refinement is otherwise stable, with reasonable displacement parameters and no chemically unreasonable features. Additional refinement cycles do not lead to significant changes in the structural model, and the reported structure is considered reliable.

"Maximum Shift/Error .... 1.78 Why ?"

This alert arises because the final refinement cycle shows a maximum shift/error of 1.78 due to enhanced thermal motion at 400 K. The structural model is stable and reliable, and additional cycles do not change the results.

"Large Hirshfeld Difference C037 --C20 . 0.50 Ang."

"Large Hirshfeld Difference C03Q --C6A . 0.37 Ang."

This alert arises because the Hirshfeld rigid-bond test shows relatively large differences for the C037 --C20 and C03Q --C6A bonds. These bonds involve atoms with comparatively large and anisotropic displacement parameters, and the observed deviations are attributed to enhanced thermal motion and local flexibility of the molecular framework. The differences do not indicate any disorder, misassignment, or deficiencies in the structural model, and the displacement parameters are physically reasonable.

B-Level alerts

"Ratio Observed / Unique Reflections (too) Low .. 32%"

This alert arises because many reflections are weak and fall below the $I > 2\sigma(I)$ threshold, resulting in a low observed/unique ratio. The refinement remains stable and the structural model is reliable.

"Atom C20 has ADP max/min Ratio ..... 4.5 oblate"

This alert arises because atom C20 exhibits a strongly anisotropic displacement ellipsoid (oblate) with a relatively large ADP max/min ratio (4.5). This behavior is attributed to anisotropic thermal motion and local flexibility of the molecular fragment containing C20 under the measurement conditions, which leads to a preferred direction of atomic displacement. The displacement parameters remain physically reasonable and do not indicate any misassignment or missing disorder model; the refinement is stable and the structural model is reliable.

"Large Hirshfeld Difference C034 --C2A .. 0.26 Ang."

"Large Hirshfeld Difference C03L --C45A.. 0.28 Ang."

This alert arises because the Hirshfeld rigid-bond test shows relatively large differences for the C034 --C2A and C03L –C45A bonds. These bonds involve atoms with

comparatively large and anisotropic displacement parameters, and the observed deviations are attributed to enhanced thermal motion and local flexibility of the molecular framework. The differences do not indicate any disorder, misassignment, or deficiencies in the structural model, and the displacement parameters are physically reasonable.

"Low 'MainMol' Ueq as Compared to Neighbors of C02V"
"Low 'MainMol' Ueq as Compared to Neighbors of C034"
"Low 'MainMol' Ueq as Compared to Neighbors of C037"
"Low 'MainMol' Ueq as Compared to Neighbors of C03Q"
"Low 'MainMol' Ueq as Compared to Neighbors of C03L"
"Low 'MainMol' Ueq as Compared to Neighbors of C03Y"
This alert arises because some atoms in the main molecule show slightly lower Ueq values than their neighbors due to local rigidity and anisotropic thermal motion. These features do not affect the validity of the refined structure.

"Missing FCF Reflection(s) Below Theta(Min)[Deg]= 1.51 Note"
This alert arises because some very low-angle reflections below $\theta = 1.51°$ were excluded due to limited reliability and do not influence the refined structure.

2.4 Symmetry-reorganized crystal structures after phase transition

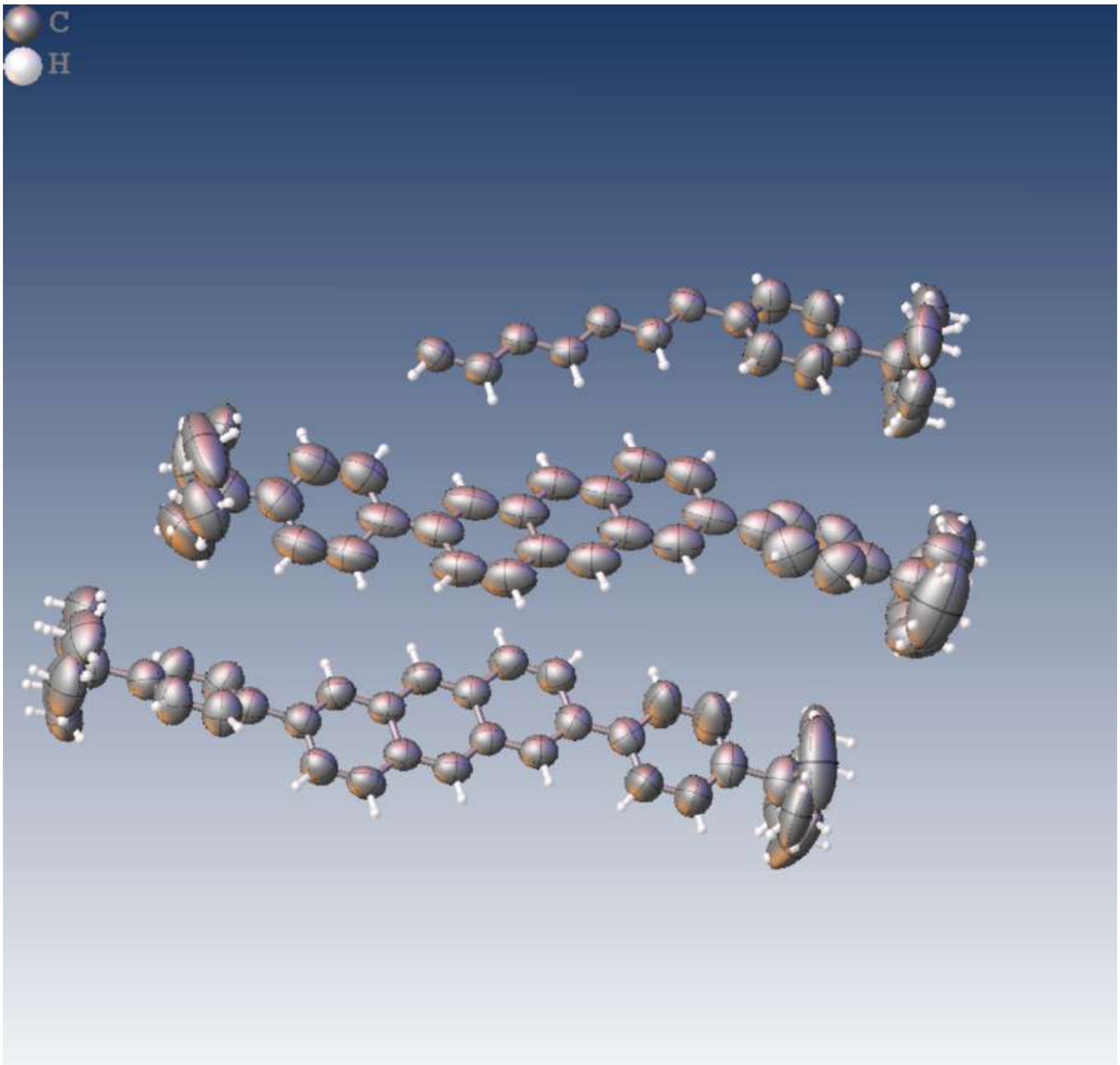

Figure S5. Crystal structure of **BBPA** determined at 423 K (CCDC 2532311). ORTEP drawing shown with 50% probability anisotropic displacement parameters. *Z*′ = 2.5.

Table S7. Crystal Data and Structure Refinement Parameters for the **BBPA** at 423 K

Identification code	exp_423	
CCDC deposit number	2532311	
Empirical formula	$C_{34}H_{34}$	
Formula weight	442.61	
Wavelength	0.4133 Å	
Crystal system	monoclinic	
Space group	$P2_1/c$	
Unit cell dimensions	a = 17.7442(7) Å	α = 90º
	b = 28.9193(7) Å	β = 102.709(4)º
	c = 13.4932(5) Å	γ = 90º
Volume	6754.4(4) Å^3	
Z	10	
Density (calculated)	1.088 g/cm^3	
Absorption coefficient	0.029 mm^{-1}	
F(000)	2380.0	
2θ range for data collection	2.98 to 43.166º	
Index ranges	-31≦h≦31, -51≦k≦50, -24≦l≦23	
Reflections collected	350571	
Independent reflections	35529 [R(int) = 0.1396]	
Data / restraints / parameters	35529 / 15 / 936	
Goodness-of-fit on F^2	0.931	
Final R indices [I>2σ(I)]	R_1 = 0.1141, wR_2 = 0.3350	
R indices (all data)	R_1 = 0.3139, wR_2 = 0.4754	
Largest diff. peak and hole	0.27/-0.15e.Å^{-3}	

A- and B-level alerts in the Checkcif file:

A-Level alerts

"The absolute value of parameter shift to su ratio > 0.20"

This alert arises because the final refinement cycle shows a relatively large shift-to-standard-uncertainty ratio, which is attributed to the high measurement temperature (423 K), leading to enhanced thermal motion and large, highly correlated atomic displacement parameters. The refinement is otherwise stable, with reasonable displacement parameters and no chemically unreasonable features. Additional refinement cycles do not lead to significant changes in the structural model, and the reported structure is considered reliable.

"Ratio Observed / Unique Reflections (too) Low .. 22%"

This alert arises because the ratio of observed to unique reflections is low (22%), as a large number of reflections do not satisfy the criterion $I > 2\sigma(I)$, particularly at higher diffraction angles. This behavior is consistent with weak scattering and enhanced thermal motion under the measurement conditions. Nevertheless, the refinement converged stably with physically reasonable displacement parameters and no significant residual electron density features, supporting the reliability of the refined structural model.

"Maximum Shift/Error .... 0.40 Why ?"

This alert arises because the final refinement cycle shows a maximum shift/error of 0.40 due to enhanced thermal motion at 423 K, leading to increased correlations among refined parameters. The structural model is stable and reliable, and additional refinement cycles do not change the results.

"High wR2 Value (i.e. > 0.25) .... 0.48"

This alert arises because the wR2 is relatively high due to the presence of many weak reflections, particularly at higher diffraction angles, which are strongly affected by enhanced thermal motion under the measurement conditions. As a result, the weighting scheme amplifies the contribution of these weak reflections to wR2. Nevertheless, the refinement converged stably with chemically reasonable geometry, physically meaningful displacement parameters, and no significant residual electron density features, supporting the reliability of the structural model.

B-Level alerts

"Hirshfeld Test Diff for C005 --C00D.. 8.3 s.u."
"Hirshfeld Test Diff for C00J --C00V.. 10.0 s.u."
"Hirshfeld Test Diff for C00M --C014.. 7.7 s.u."
"Hirshfeld Test Diff for C00Q --C00S.. 16.3 s.u."
"Hirshfeld Test Diff for C012 --C01D.. 11.3 s.u."
"Hirshfeld Test Diff for C01F --C01S.. 7.7 s.u."
"Hirshfeld Test Diff for C01M --C01Y.. 9.2 s.u."
"Hirshfeld Test Diff for C01O --C01X.. 9.5 s.u."

This alert arises because the Hirshfeld rigid bond test indicates elevated differences (in s.u.) for a small number of C-C bonds (C005-C00D, C00J-C00V, C00M-C014, C00Q-C00S, C012-C01D, C01F-C01S, C01M-C01Y, and C01O-C01X). These bonds involve atoms located in structurally flexible regions, including tert-butyl substituents and other moieties with significant rotational freedom. At the elevated measurement temperature, enhanced thermal motion and local libration and rotational motion lead to anisotropic and partially correlated displacement parameters, giving rise to larger Hirshfeld test differences. The displacement parameters are physically reasonable and these features do not indicate disorder, misassignment, or deficiencies in the structural model.

“Low ’MainMol’ Ueq as Compared to Neighbors of C01U”
“Low ’MainMol’ Ueq as Compared to Neighbors of C01V”
“Low ’MainMol’ Ueq as Compared to Neighbors of C01P”
“Low ’MainMol’ Ueq as Compared to Neighbors of C01X”
“Low ’MainMol’ Ueq as Compared to Neighbors of C01Y”
This alert arises because several atoms in the main molecule (C01U, C01V, C01P, C01X, and C01Y) show slightly lower equivalent isotropic displacement parameters (Ueq) compared to their neighboring atoms. These atoms are located in locally rigid parts of the molecular framework or in regions undergoing anisotropic thermal motion, while adjacent atoms belong to more flexible groups with significant rotational freedom (e.g. tert-butyl substituents). At the elevated measurement temperature, such differences in local motion lead to non-uniform but physically reasonable displacement parameters. These features do not indicate disorder, misassignment, or deficiencies in the structural model.

“Missing FCF Reflection(s) Below Theta(Min)[Deg]= 1.49 Note”
This alert arises because some very low-angle reflections below $\theta = 1.49^{\circ}$ were excluded due to limited reliability and do not influence the refined structure.

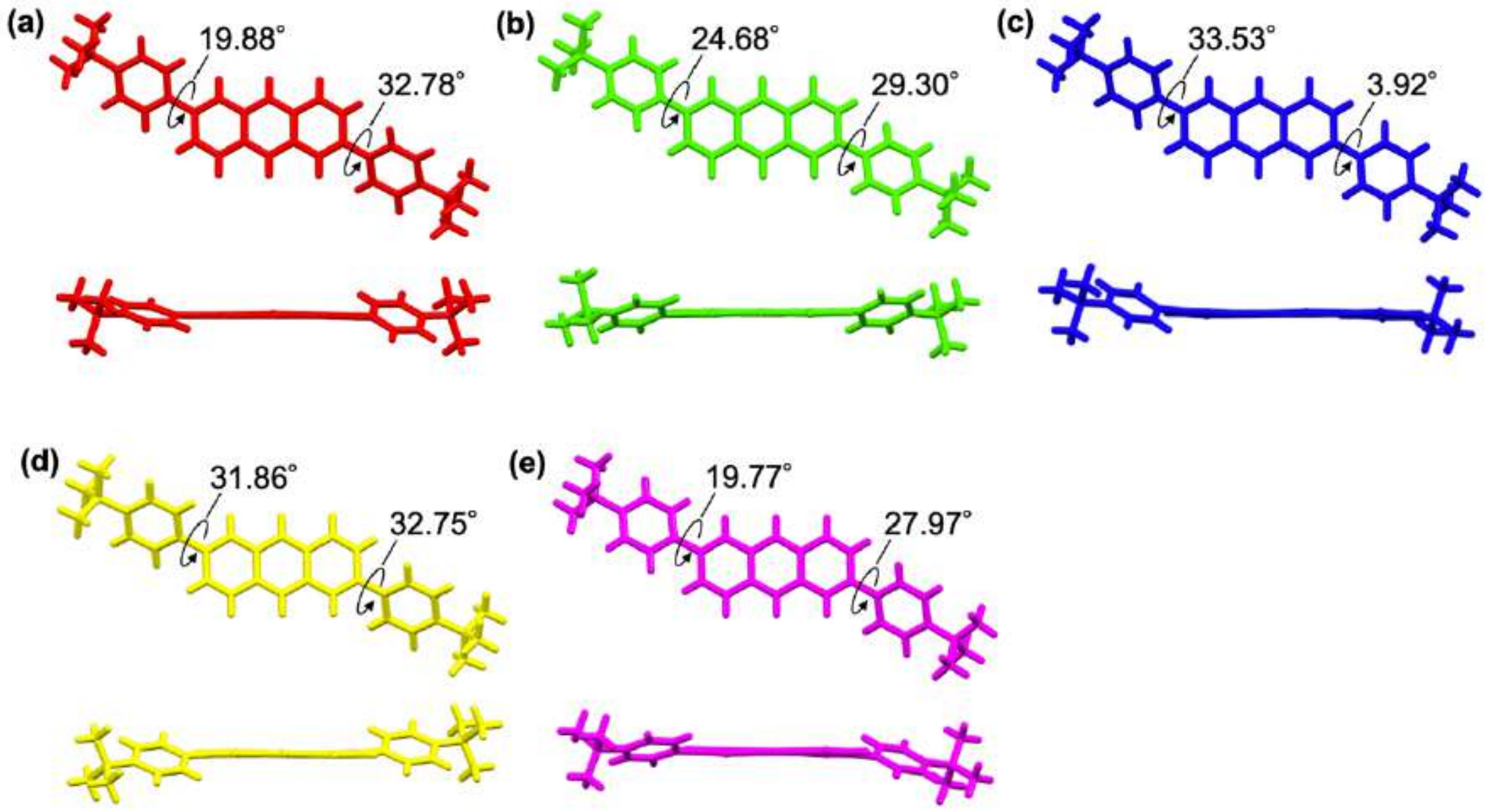

Figure S6. Crystal structures of **BBPA** determined at 100 K (CCDC 2532253). The five crystallographically independent conformers are shown in (a) red, (b) green, (c) blue, (d) yellow, and (e) pink. The dihedral angles between the anthracene and phenyl moieties are indicated. Disordered atoms are omitted for clarity.

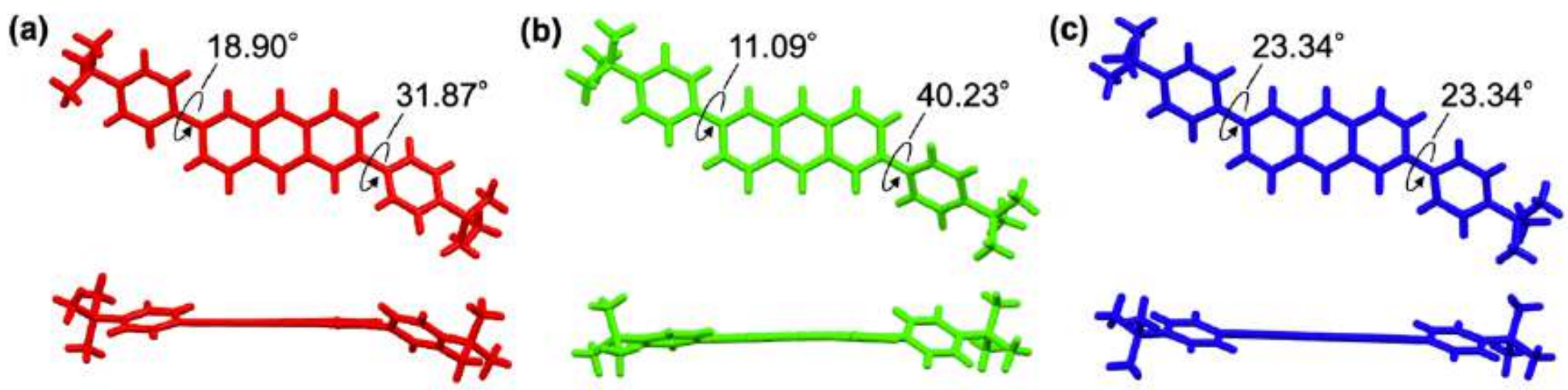

Figure S7. Crystal structures of **BBPA** determined at 423 K (CCDC 2532311). The three crystallographically independent conformers are shown in (a) red, (b) green, and (c) blue. The dihedral angles between the anthracene and phenyl moieties are indicated. Disordered atoms are omitted for clarity.

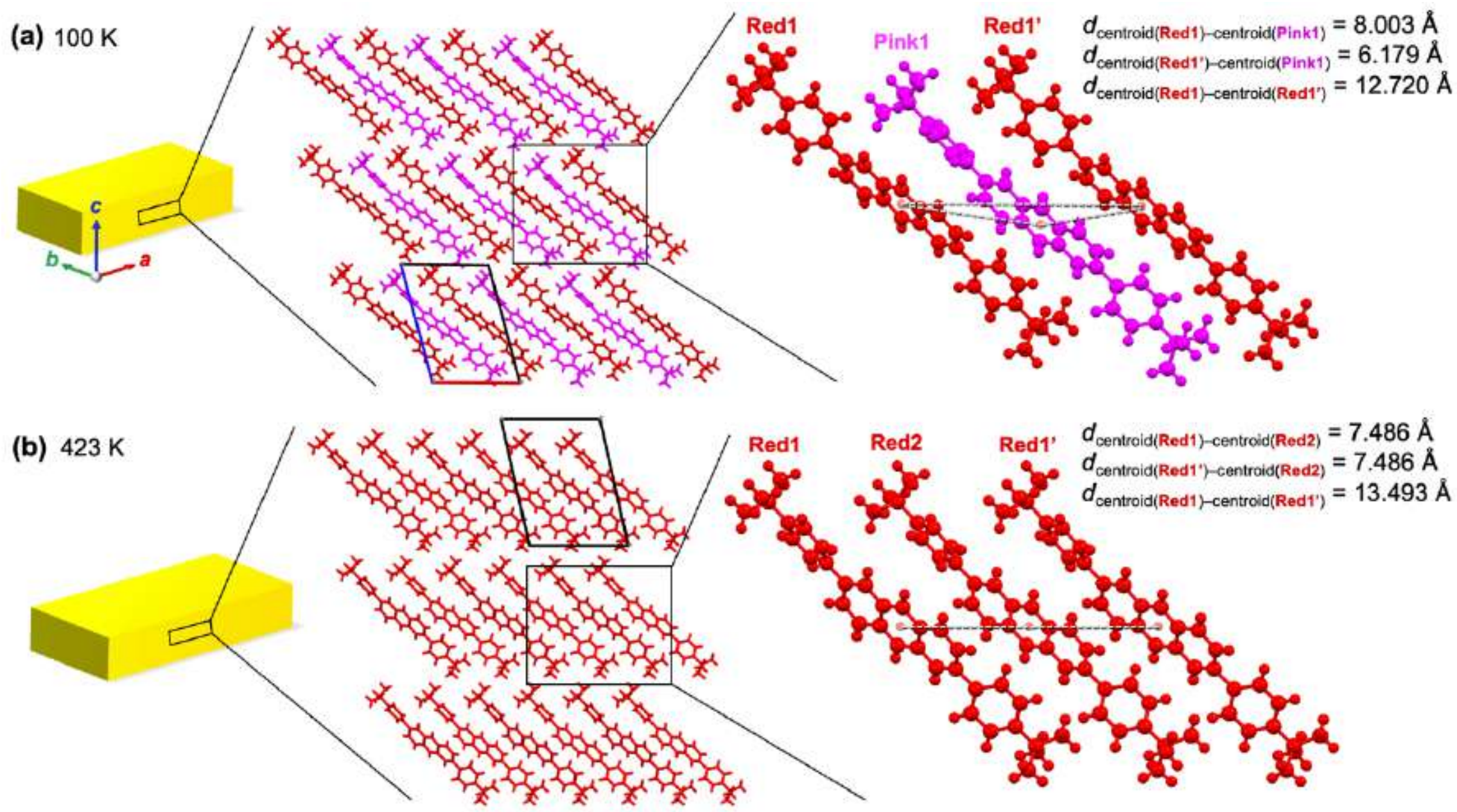

Figure S8. Crystal packing structures of **BBPA** viewed along the *b*-axis at (a) 100 K and (b) 423 K. The two crystallographically independent conformers are shown in red and pink. The reduction in *Z*′ is accompanied by modulation of the anthracene centroid–centroid distances ($d_{\text{centroid–centroid}}$) along the *a*-axis.

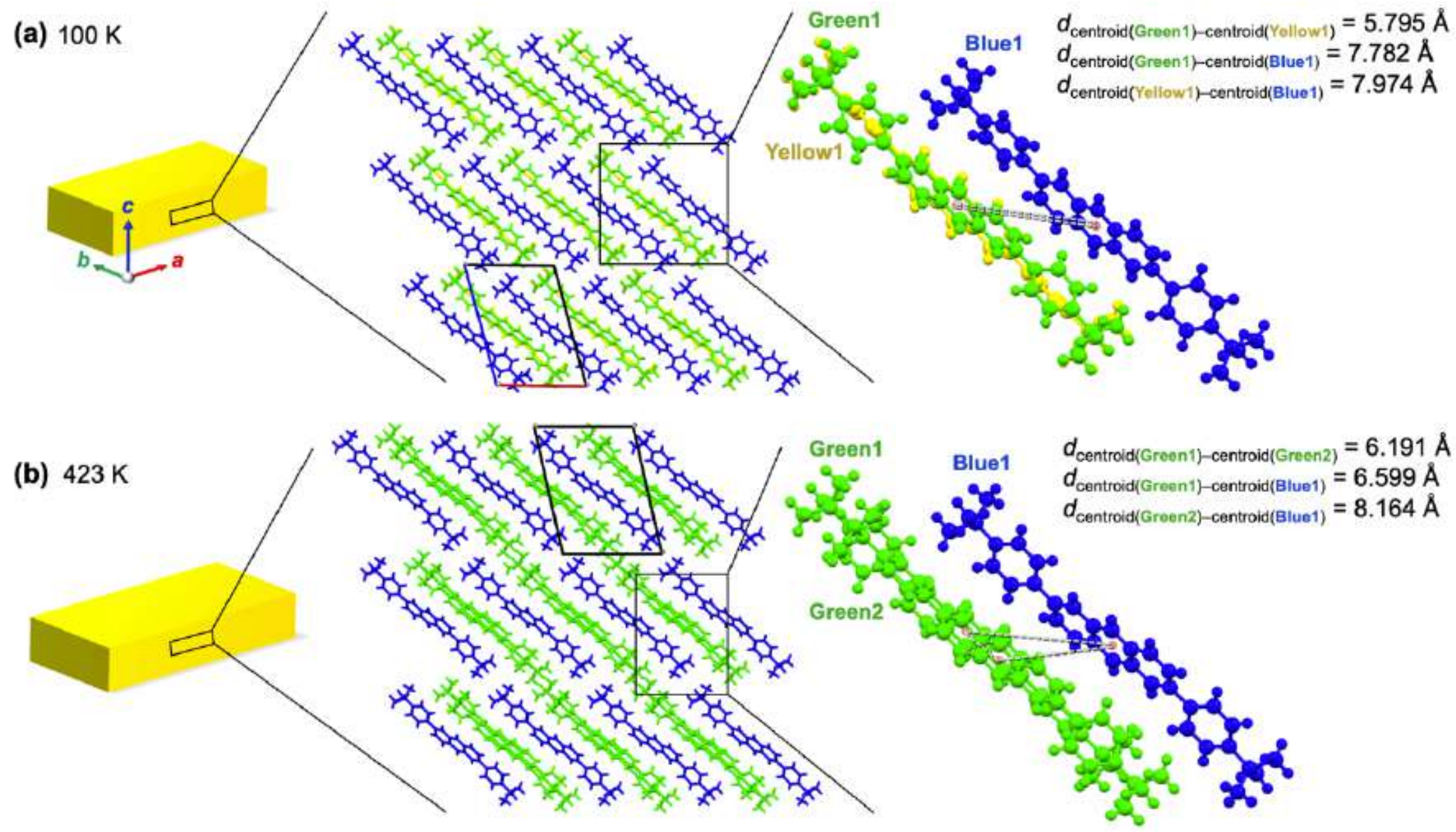

Figure S9. Crystal packing structures of **BBPA** viewed along the *b*-axis at (a) 100 K and (b) 423 K. The three crystallographically independent conformers are shown in blue, green, and yellow. The reduction in *Z*′ is accompanied by modulation of the anthracene centroid–centroid distances ($d_{\text{centroid–centroid}}$) along the *a*-axis.

2.5 Single-unit-cell reciprocal-space reconstruction

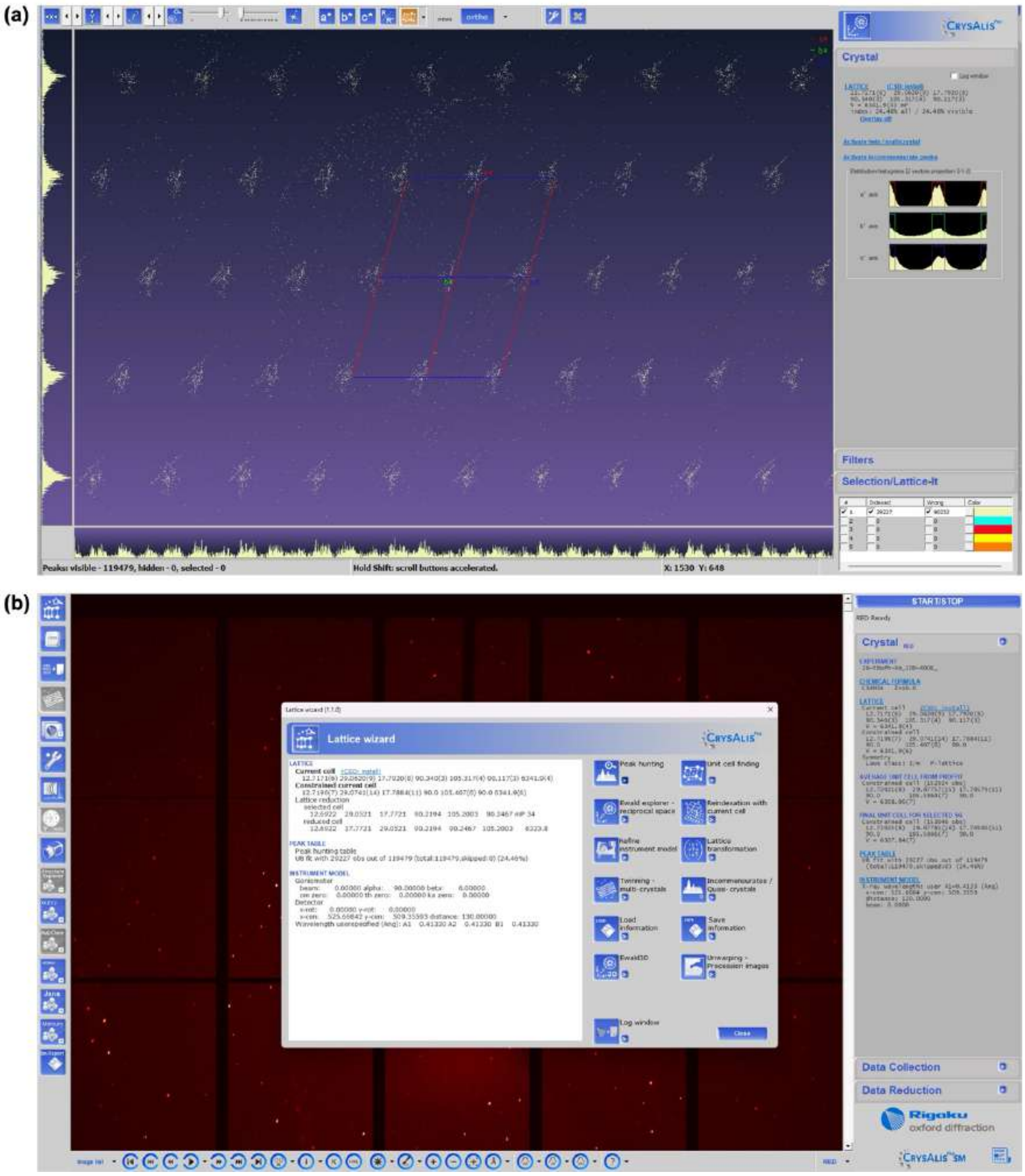

Figure S10. Representative reciprocal-space reconstruction and diffraction frames of **BBPA** crystal collected at 100 K using CrysAlisPro. All observed reflections can be indexed by a single unit cell, with no evidence of superstructure reflections or twinning. (a) Reconstructed reciprocal lattice plane and (b) unit cell determination. The unit cell was observed in the monoclinic crystal system with lattice parameters a = 12.7176(6) Å, b = 29.0741(14) Å, c = 17.7884(11) Å, and β = 105.407(6)º.

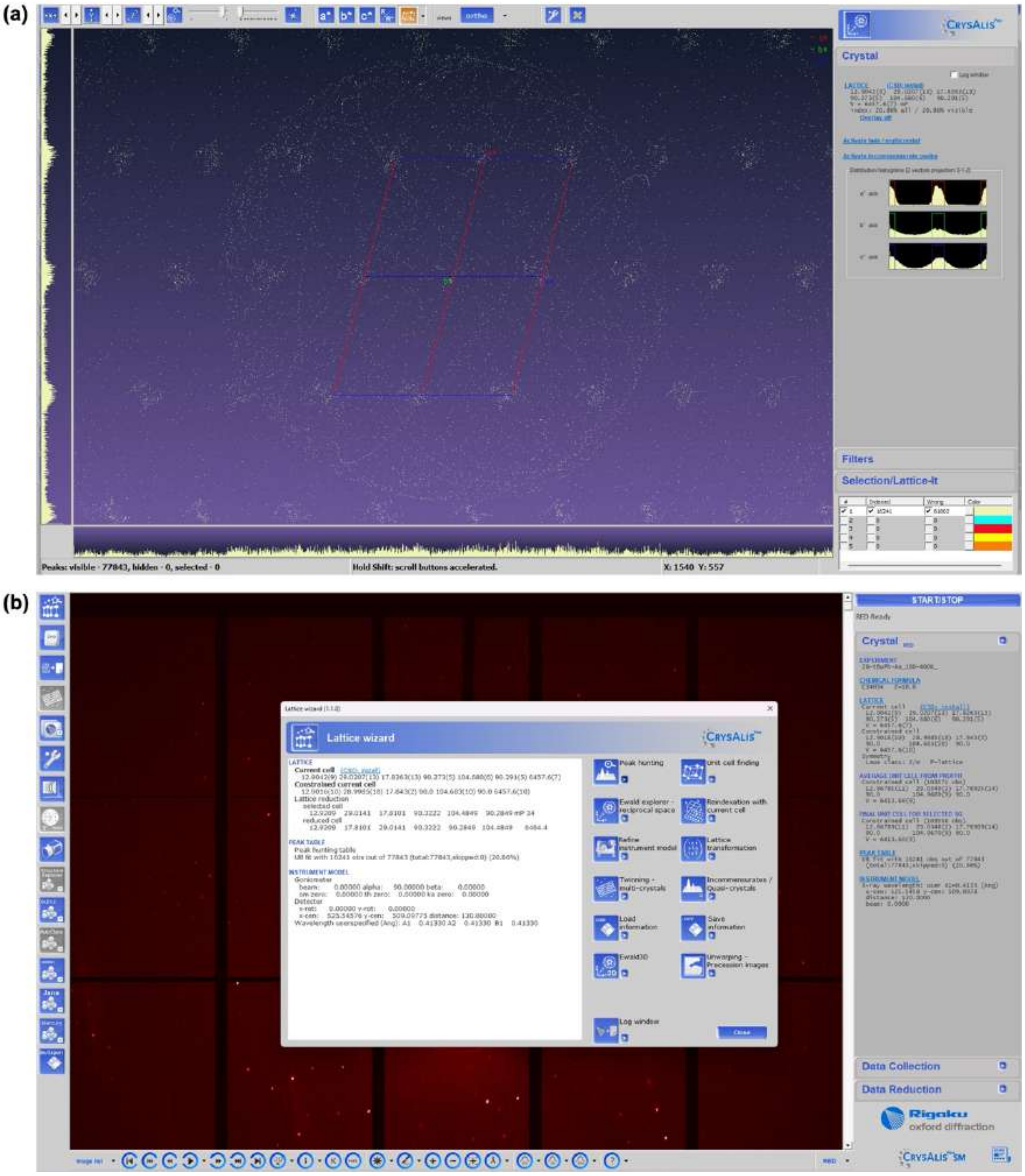

Figure S11. Representative reciprocal-space reconstruction and diffraction frames of **BBPA** crystal collected at 200 K using CrysAlisPro. All observed reflections can be indexed by a single unit cell, with no evidence of superstructure reflections or twinning. (a) Reconstructed reciprocal lattice plane and (b) unit cell determination. The unit cell was observed in the monoclinic crystal system with lattice parameters a = 12.9016(10) Å, b = 28.9985(18) Å, c = 17.843(2) Å, and β = 104.683(10)º.

(a) (b)

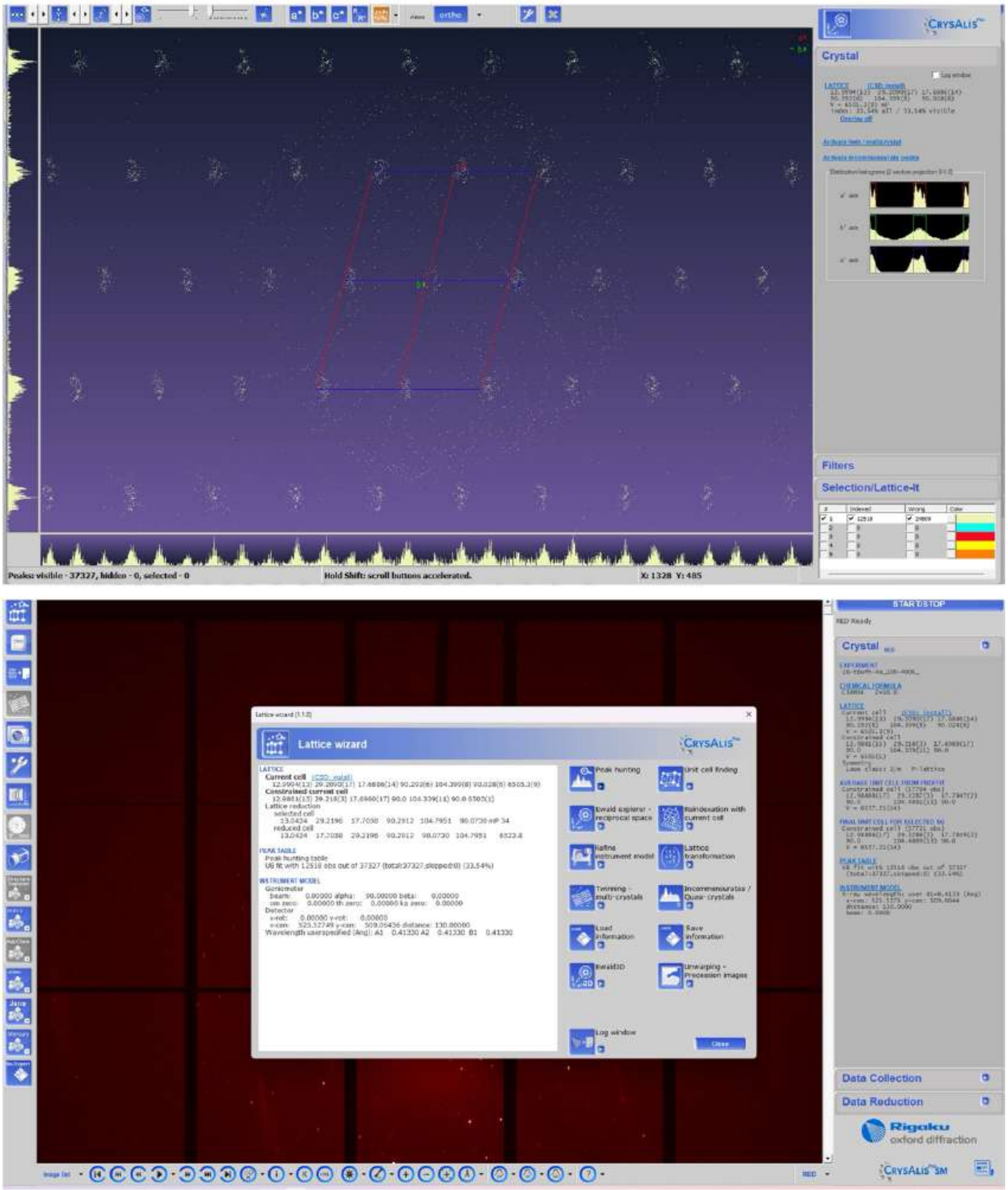

Figure S12. Representative reciprocal-space reconstruction and diffraction frames of **BBPA** crystal collected at 300 K using CrysAlisPro. All observed reflections can be indexed by a single unit cell, with no evidence of superstructure reflections or twinning. (a) Reconstructed reciprocal lattice plane and (b) unit cell determination. The unit cell was observed in the monoclinic crystal system with lattice parameters a = 12.9861(15) Å, b = 29.218(3) Å, c = 17.6960(17) Å, and β = 104.339(11)º.

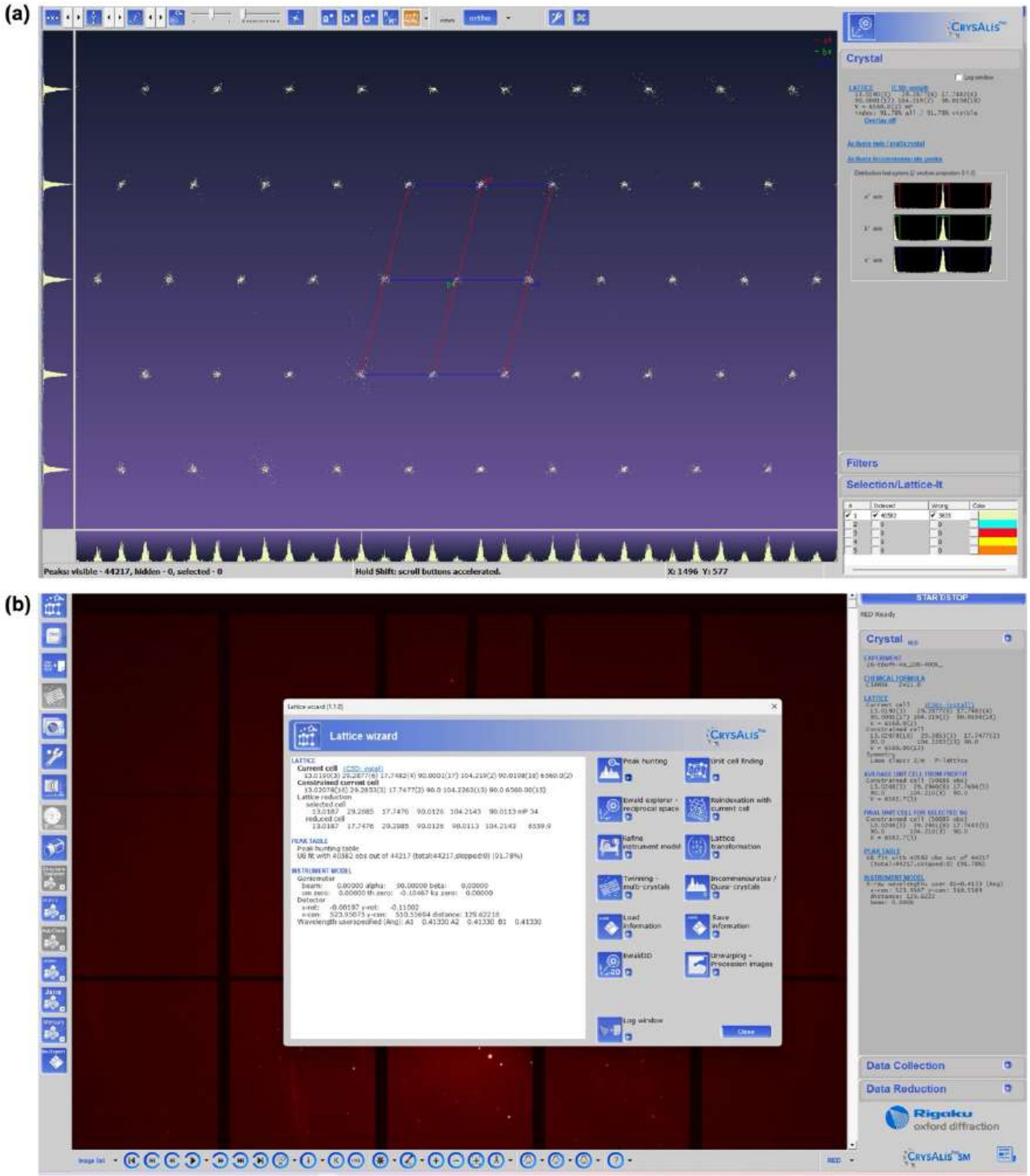

Figure S13. Representative reciprocal-space reconstruction and diffraction frames of **BBPA** crystal collected at 350 K using CrysAlisPro. All observed reflections can be indexed by a single unit cell, with no evidence of superstructure reflections or twinning. (a) Reconstructed reciprocal lattice plane and (b) unit cell determination. The unit cell was observed in the monoclinic crystal system with lattice parameters a = 13.02078(16) Å, b = 29.2853(3) Å, c = 17.7477(2) Å, and β = 104.2263(13)º.

(a)

(b)

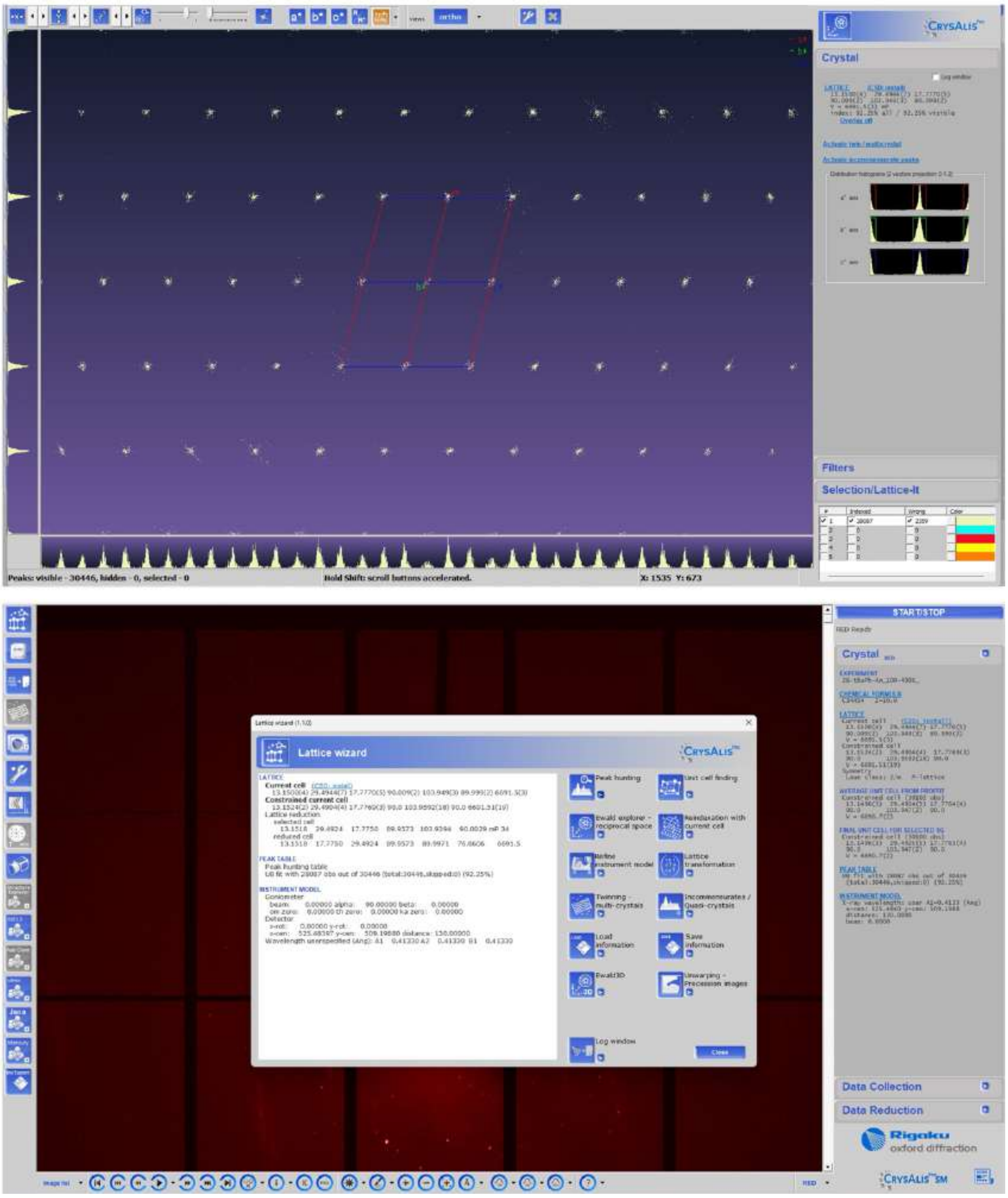

Figure S14. Representative reciprocal-space reconstruction and diffraction frames of **BBPA** crystal collected at 400 K using CrysAlisPro. All observed reflections can be indexed by a single unit cell, with no evidence of superstructure reflections or twinning. (a) Reconstructed reciprocal lattice plane and (b) unit cell determination. The unit cell was observed in the monoclinic crystal system with lattice parameters *a* = 13.1524(2) Å, *b* = 29.4904(4) Å, *c* = 17.7769(3) Å, and *β* = 103.9592(18)º.

(a)

(b)

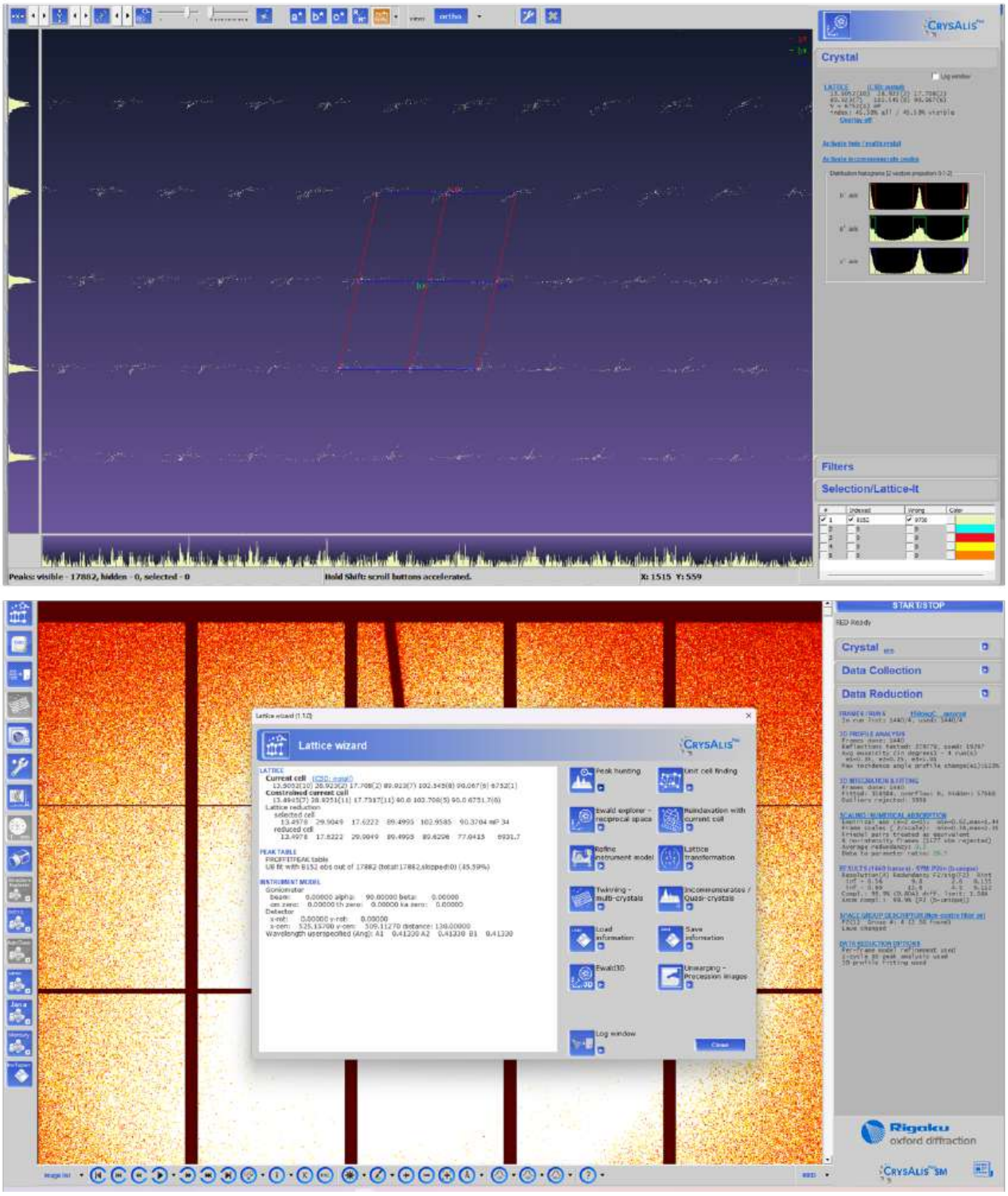

Figure S15. Representative reciprocal-space reconstruction and diffraction frames of **BBPA** crystal collected at 423 K using CrysAlisPro. All observed reflections can be indexed by a single unit cell, with no evidence of superstructure reflections or twinning. (a) Reconstructed reciprocal lattice plane and (b) unit cell determination. The unit cell was observed in the monoclinic crystal system with lattice parameters a = 13.4945(7) Å, b = 28.9251(11) Å, c = 17.7317(11) Å, and β = 102.708(5)º.

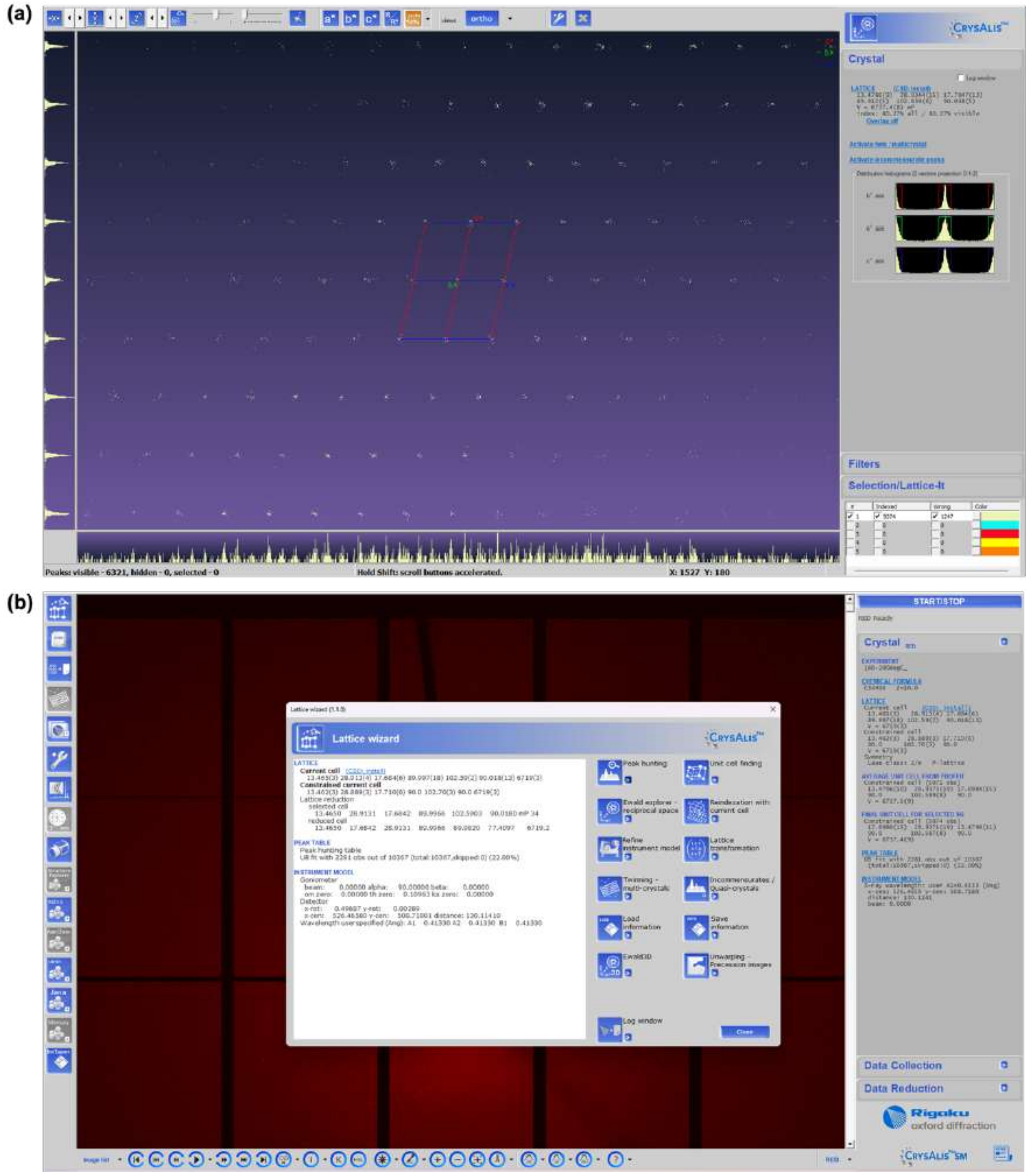

Figure S16. Representative reciprocal-space reconstruction and diffraction frames of **BBPA** crystal collected at 433 K using CrysAlisPro. All observed reflections can be indexed by a single unit cell, with no evidence of superstructure reflections or twinning. (a) Reconstructed reciprocal lattice plane and (b) unit cell determination. The unit cell was observed in the monoclinic crystal system with lattice parameters a = 13.462(3) Å, b = 28.889(3) Å, c = 17.710(6) Å, and β = 102.70(3)º.

(a)

(b)

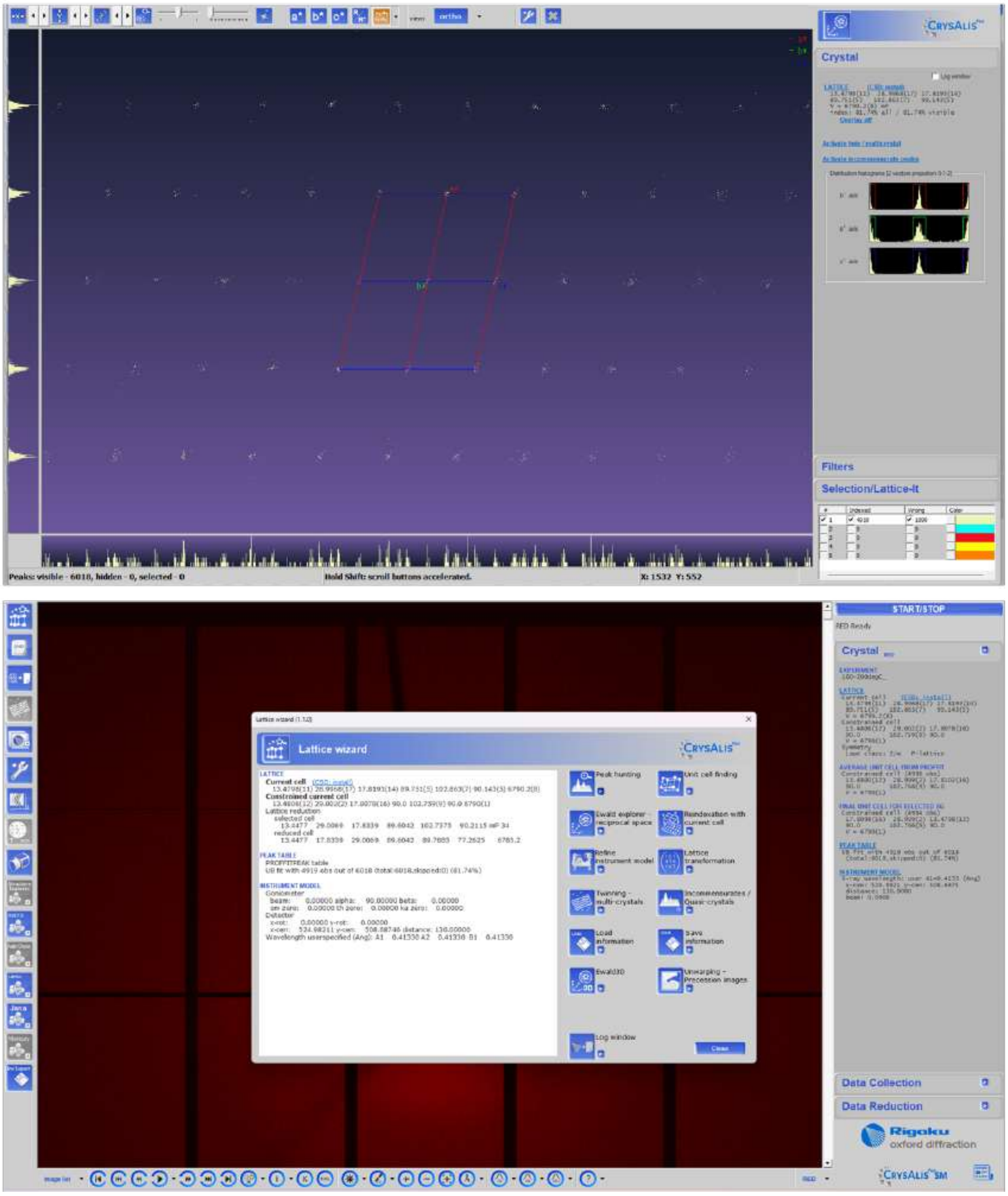

Figure S17. Representative reciprocal-space reconstruction and diffraction frames of **BBPA** crystal collected at 443 K using CrysAlisPro. All observed reflections can be indexed by a single unit cell, with no evidence of superstructure reflections or twinning. (a) Reconstructed reciprocal lattice plane and (b) unit cell determination. The unit cell was observed in the monoclinic crystal system with lattice parameters a = 13.4806(12) Å, b = 29.002(2) Å, c = 17.8078(16) Å, and β = 102.759(9)º.

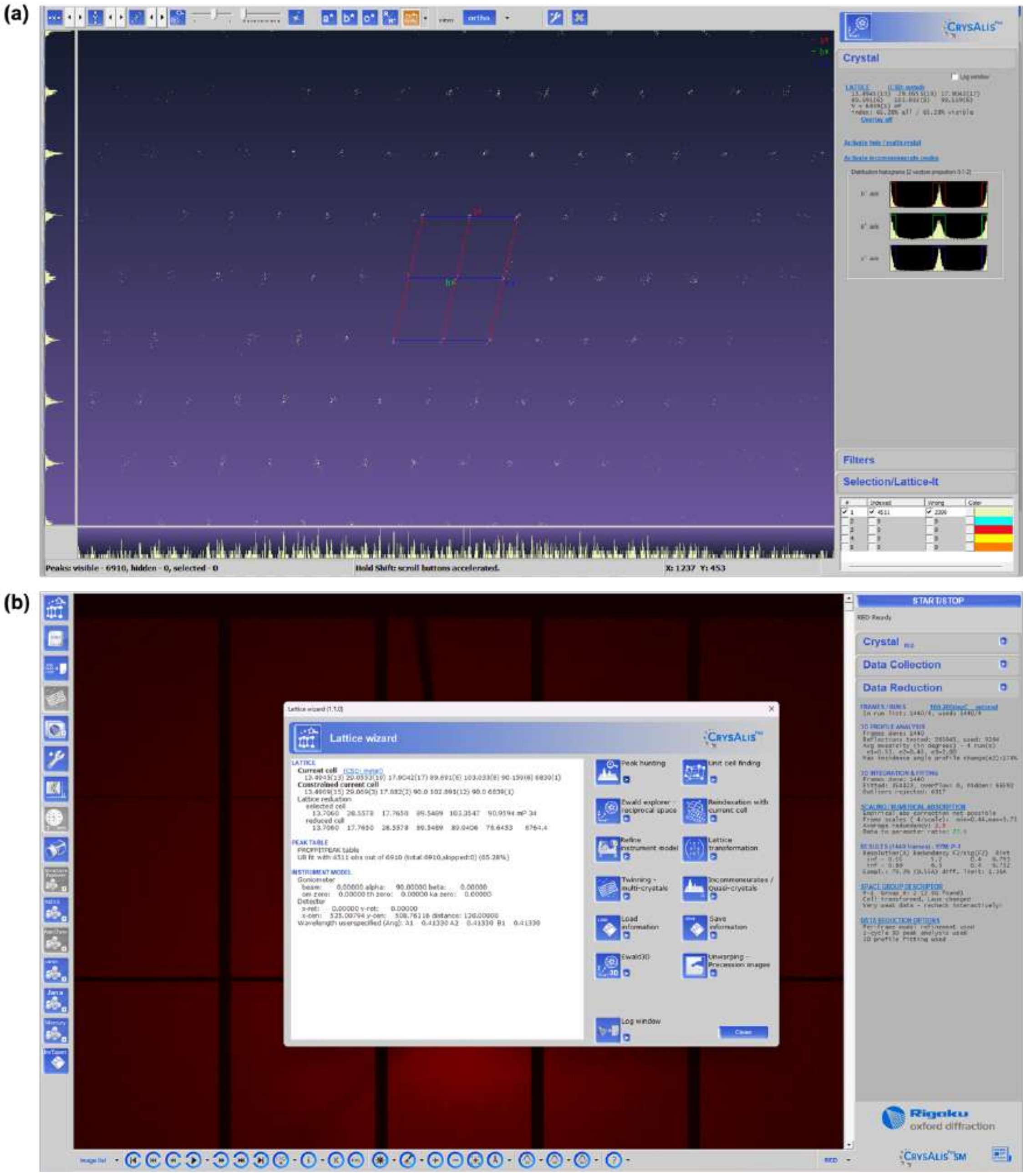

Figure S18. Representative reciprocal-space reconstruction and diffraction frames of **BBPA** crystal collected at 453 K using CrysAlisPro. All observed reflections can be indexed by a single unit cell, with no evidence of superstructure reflections or twinning. (a) Reconstructed reciprocal lattice plane and (b) unit cell determination. The unit cell was observed in the monoclinic crystal system with lattice parameters a = 13.4969(15) Å, b = 29.069(3) Å, c = 17.882(2) Å, and β = 102.891(12)º.

2.6 Variable-temperature X-ray diffraction reveals structural evolution

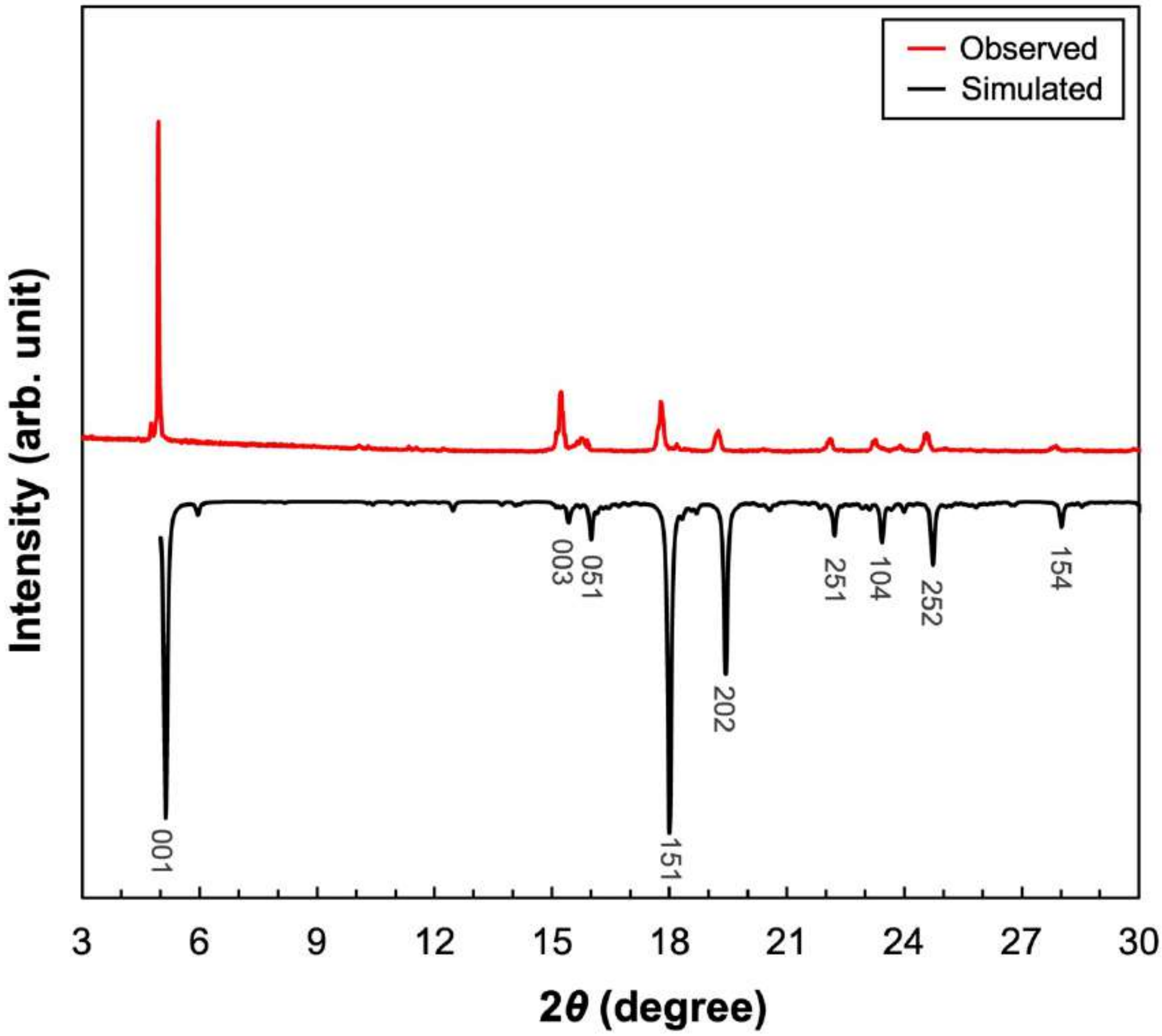

Figure S19. Simulated PXRD patterns (black line) calculated from the crystal structure of **BBPA** at 300 K (CCDC 2465110), compared with the experimental PXRD patterns (red line) measured at 303 K for the pristine single-crystal sample before heating through the phase transition. Indexed reflections are indicated.

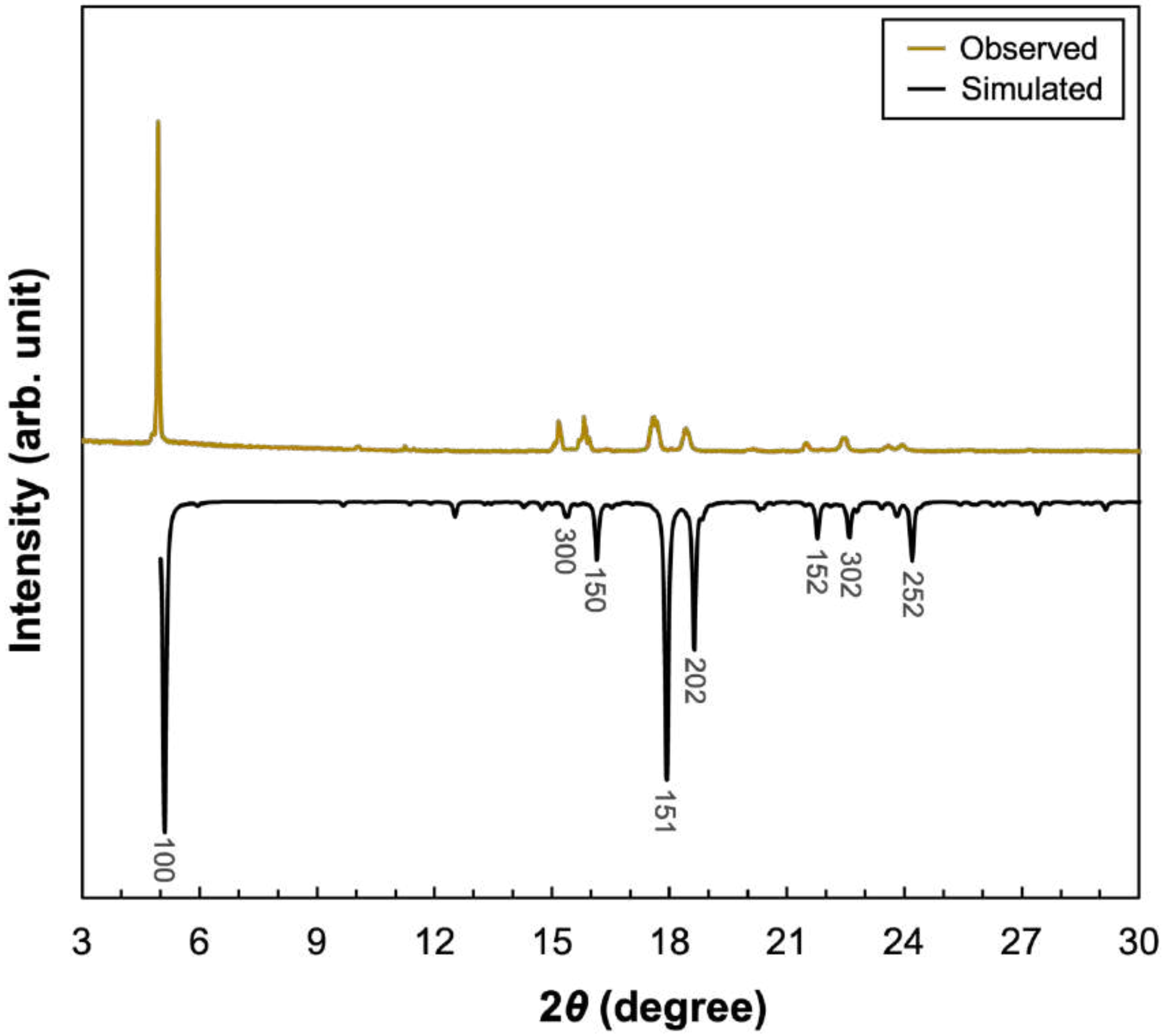

Figure S20. Simulated PXRD patterns (black line) calculated from the crystal structure of **BBPA** at 423 K (CCDC 2532311), compared with the experimental PXRD patterns (yellow line) measured for the single-crystal sample at 473 K. Indexed reflections are indicated.

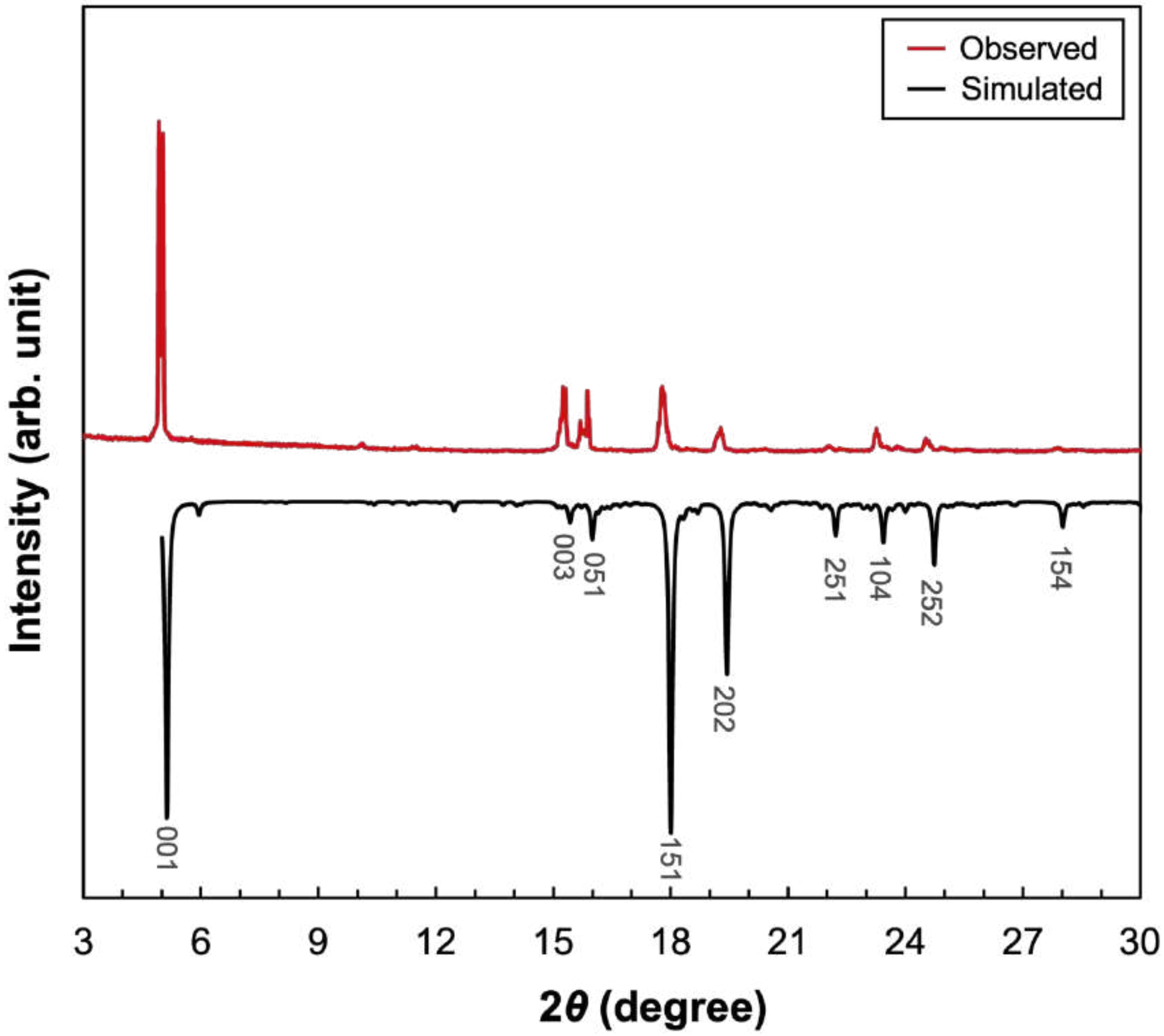

Figure S21. Simulated PXRD patterns (black line) calculated from the crystal structure of **BBPA** at 300 K (CCDC 2465110), compared with the experimental PXRD patterns (red line) measured at 303 K after cooling the single-crystal sample from the high-temperature phase. Indexed reflections are indicated.

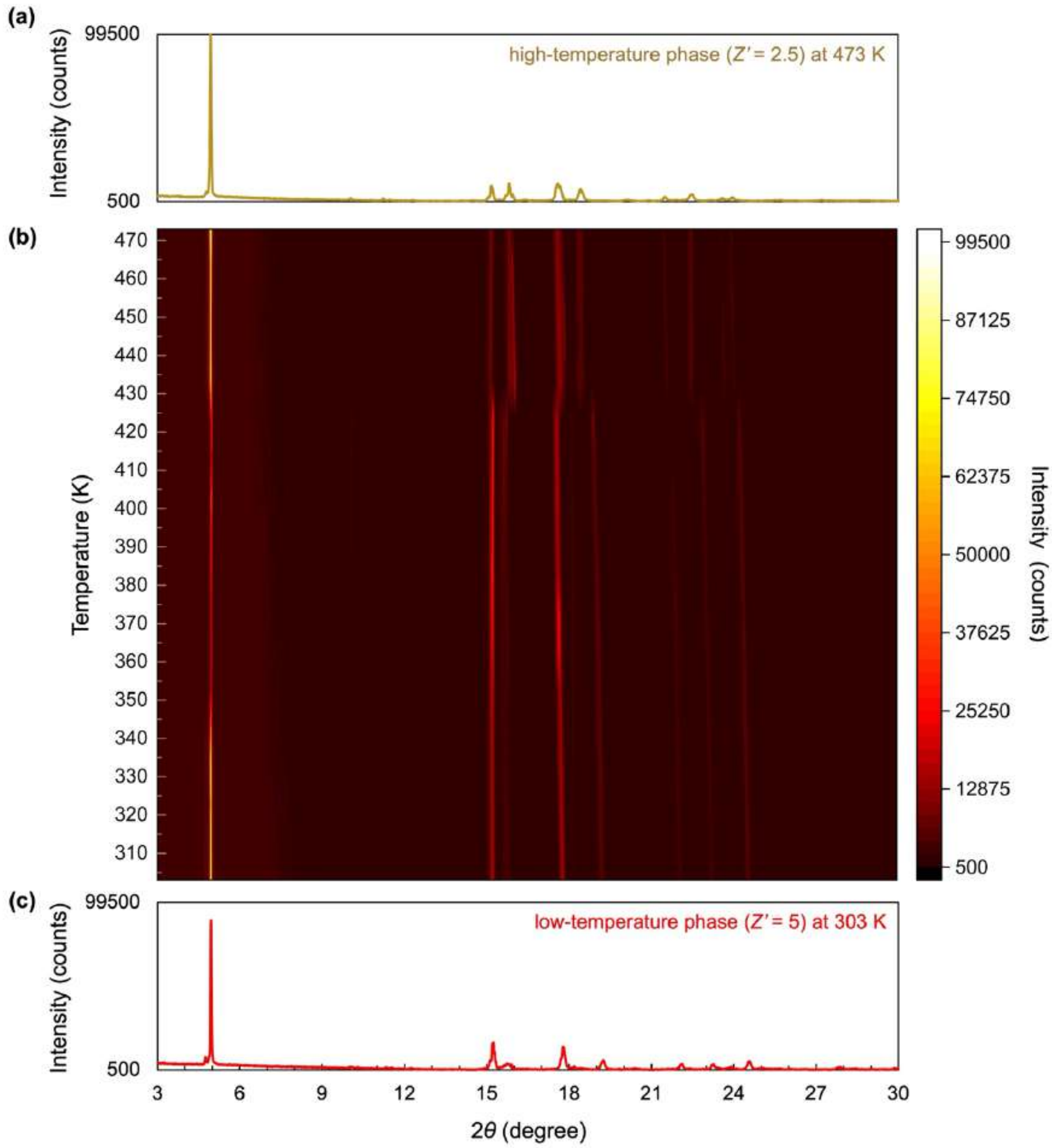

Figure S22. VT-PXRD patterns of **BBPA** collected for the single-crystal sample during heating from 303 K to 473 K. (a) PXRD patterns in the high-temperature phase at 473 K (Z' = 2.5). (b) Colormap representation of the VT-PXRD patterns. (c) PXRD patterns in the low-temperature phase at 303 K (Z' = 5).

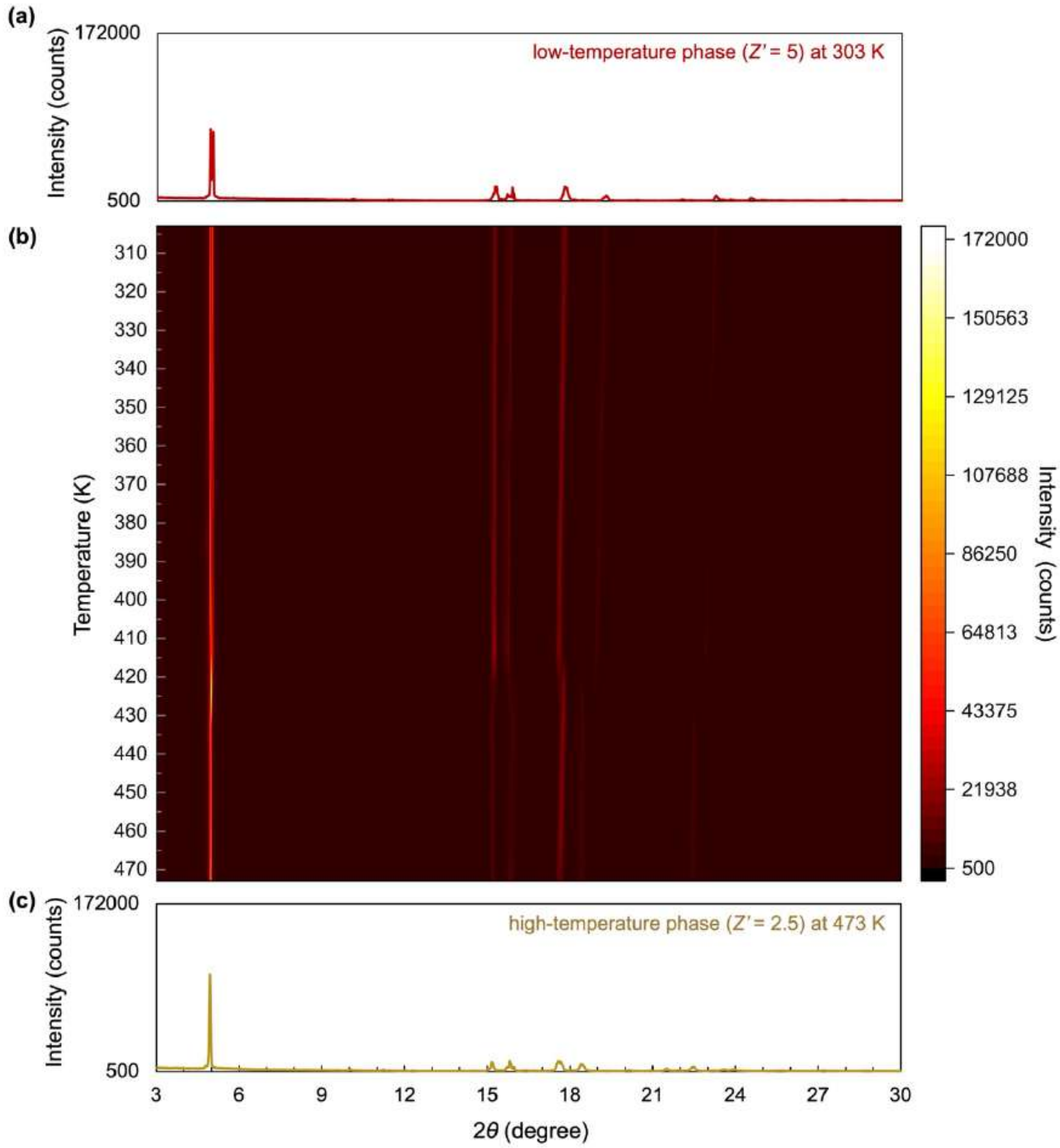

Figure S23. VT-PXRD patterns of **BBPA** collected for the single-crystal sample during cooling from 473 K to 303 K. (a) PXRD patterns in the low-temperature phase at 303 K ($Z' = 5$). (b) Colormap representation of the VT-PXRD patterns. (c) PXRD patterns in the high-temperature phase at 473 K ($Z' = 2.5$).

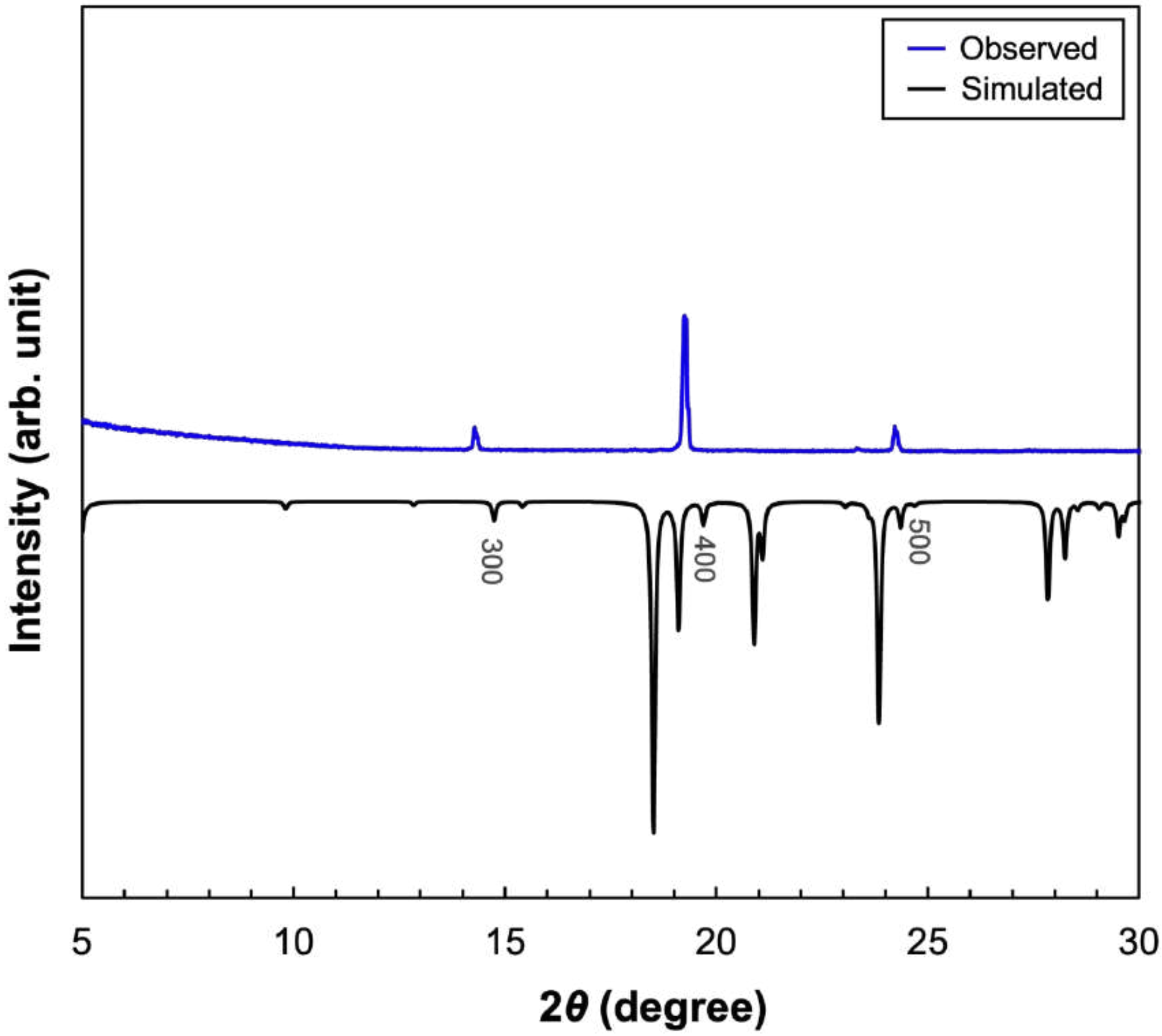

Figure S24. Simulated PXRD patterns (black line) calculated from the crystal structure of **DPA** at 103 K (CCDC 2465111), compared with the experimental PXRD patterns (blue line) measured at 303 K. Indexed reflections are indicated. The experimentally observed diffraction data exhibit sharp and intense peaks indicative of high crystalline quality, and indexing based on the single-crystal structure reveals that the dominant (*h*00) reflections arise from preferential alignment of the (*h*00) planes parallel to the substrate surface.[9]

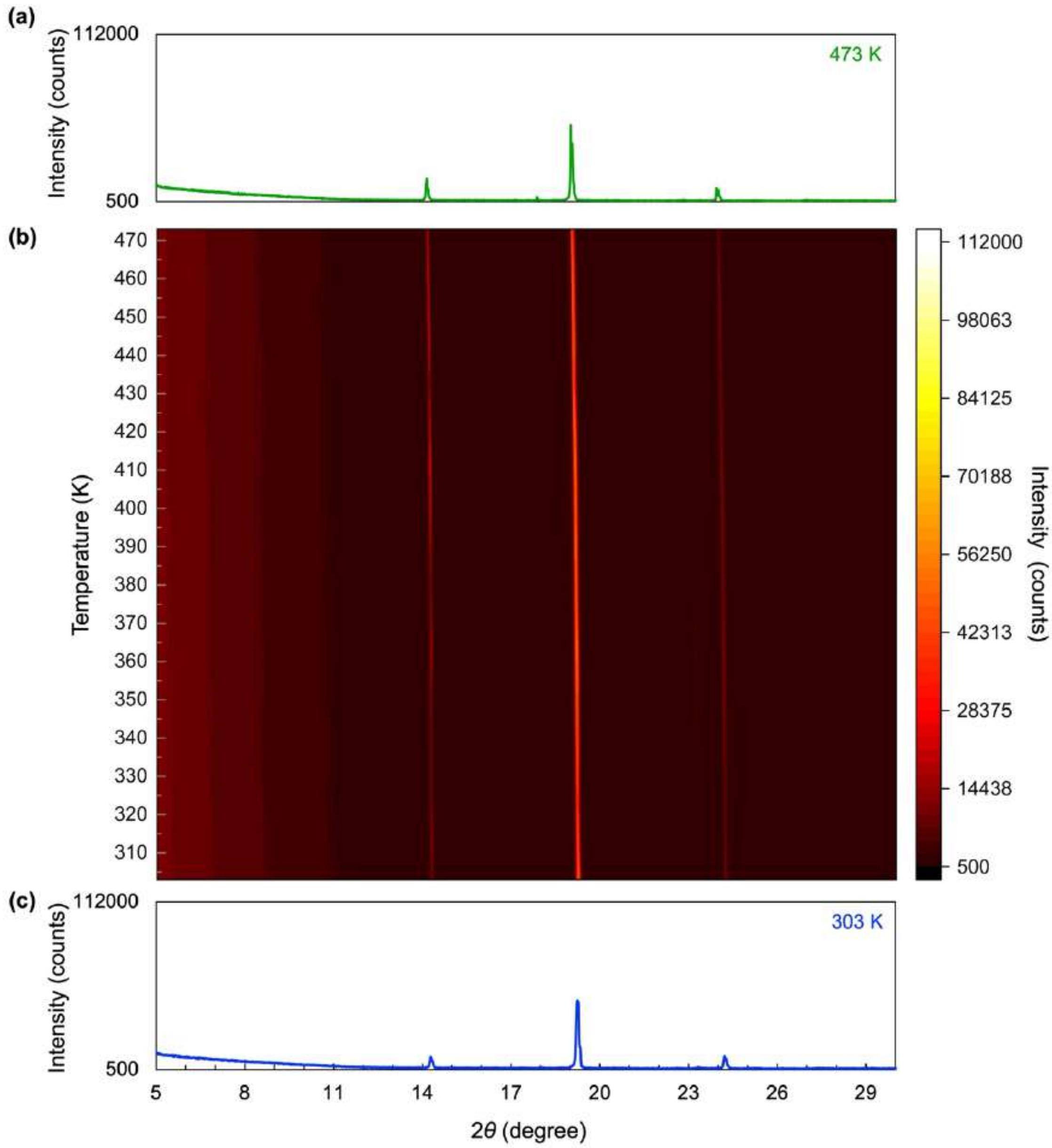

Figure S25. VT-PXRD patterns of **DPA** collected for the single-crystal sample during heating from 303 K to 473 K. (a) PXRD patterns at 473 K. (b) Colormap representation of the VT-PXRD patterns. (c) PXRD patterns at 303 K.

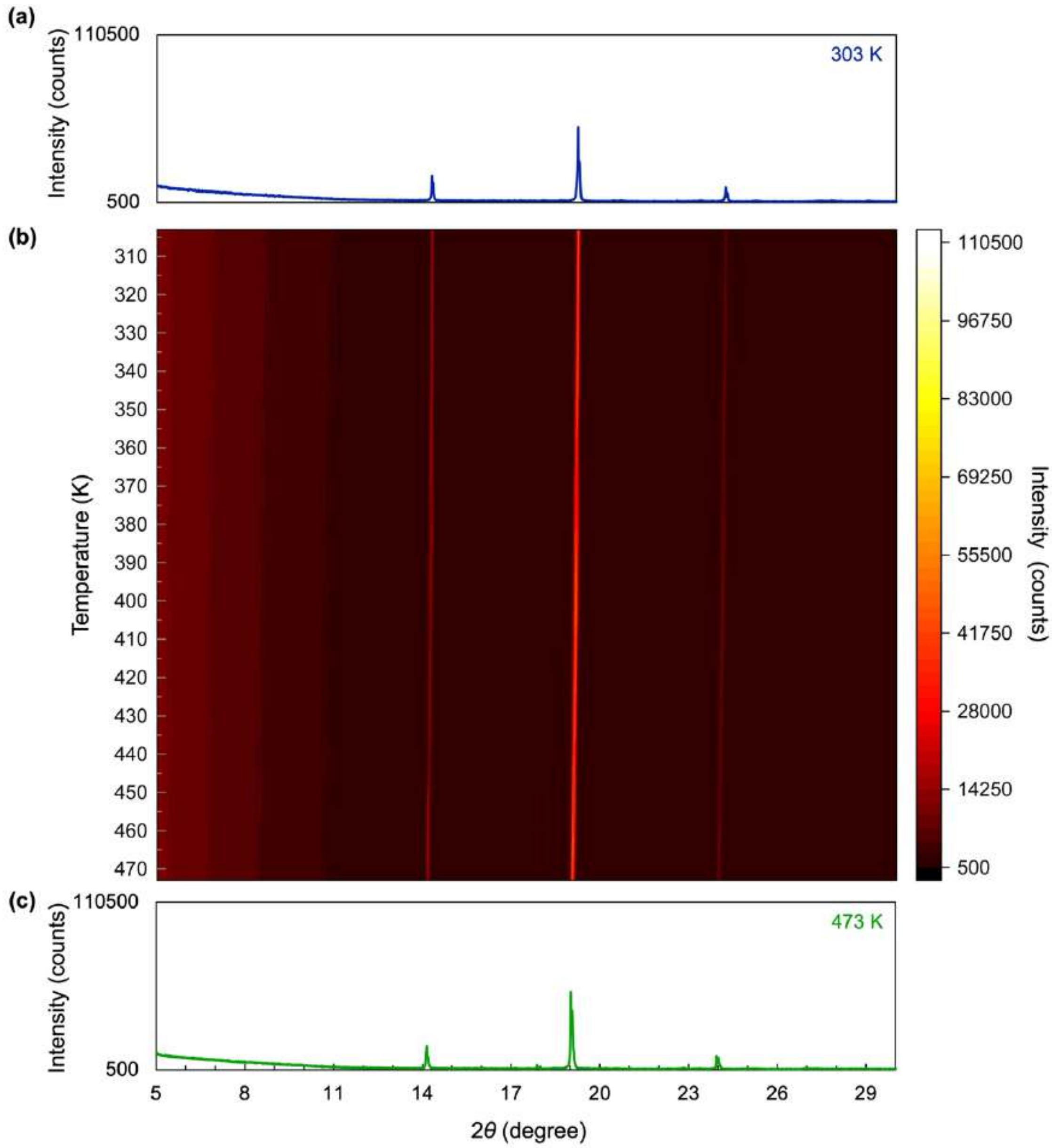

Figure S26. VT-PXRD patterns of **DPA** collected for the single-crystal sample during cooling from 473 K to 303 K. (a) PXRD patterns at 303 K. (b) Colormap representation of the VT-PXRD patterns. (c) PXRD patterns at 473 K.

3. Intermolecular interaction analysis

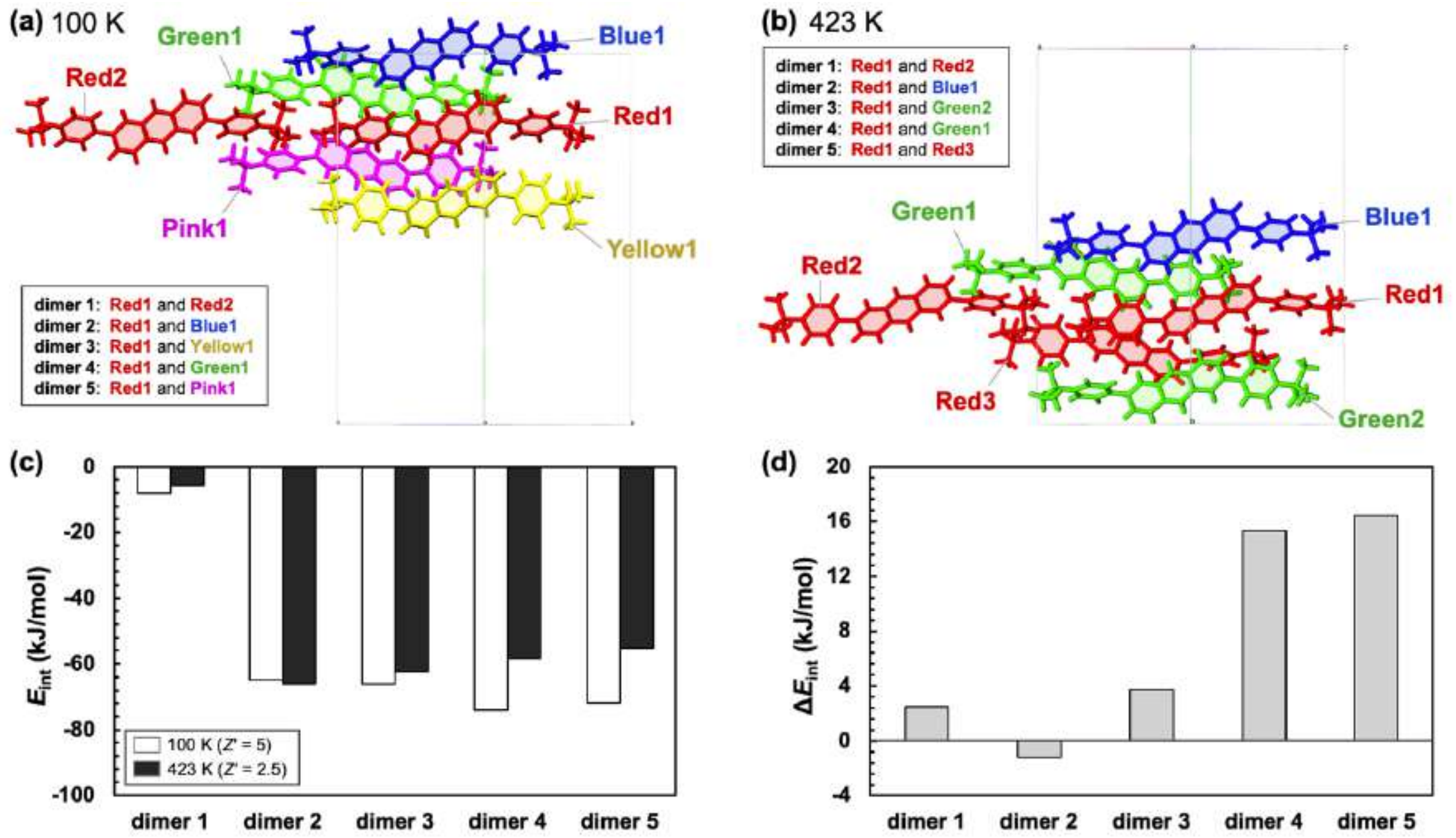

Figure S27. Crystal structures of **BBPA** showing crystallographically independent molecules in different colors for the (a) 100 K ($Z' = 5$) and (b) 423 K ($Z' = 2.5$) phases. (c) Interaction energies (E_{int}) calculated for representative molecular dimers extracted from the crystal structures at 100 K (white bars) and 423 K (black bars) using the ωB97M-D3BJ/vDZP level of theory. (d) Corresponding interaction-energy differences ($\Delta E_{\text{int}} = E_{\text{int}}$ (423 K) − E_{int} (100 K)). The broad distribution of interaction energies is consistent with the existence of multiple crystallographically independent molecular environments.

4. Raman spectroscopic observation

4.1 Computational Raman analysis

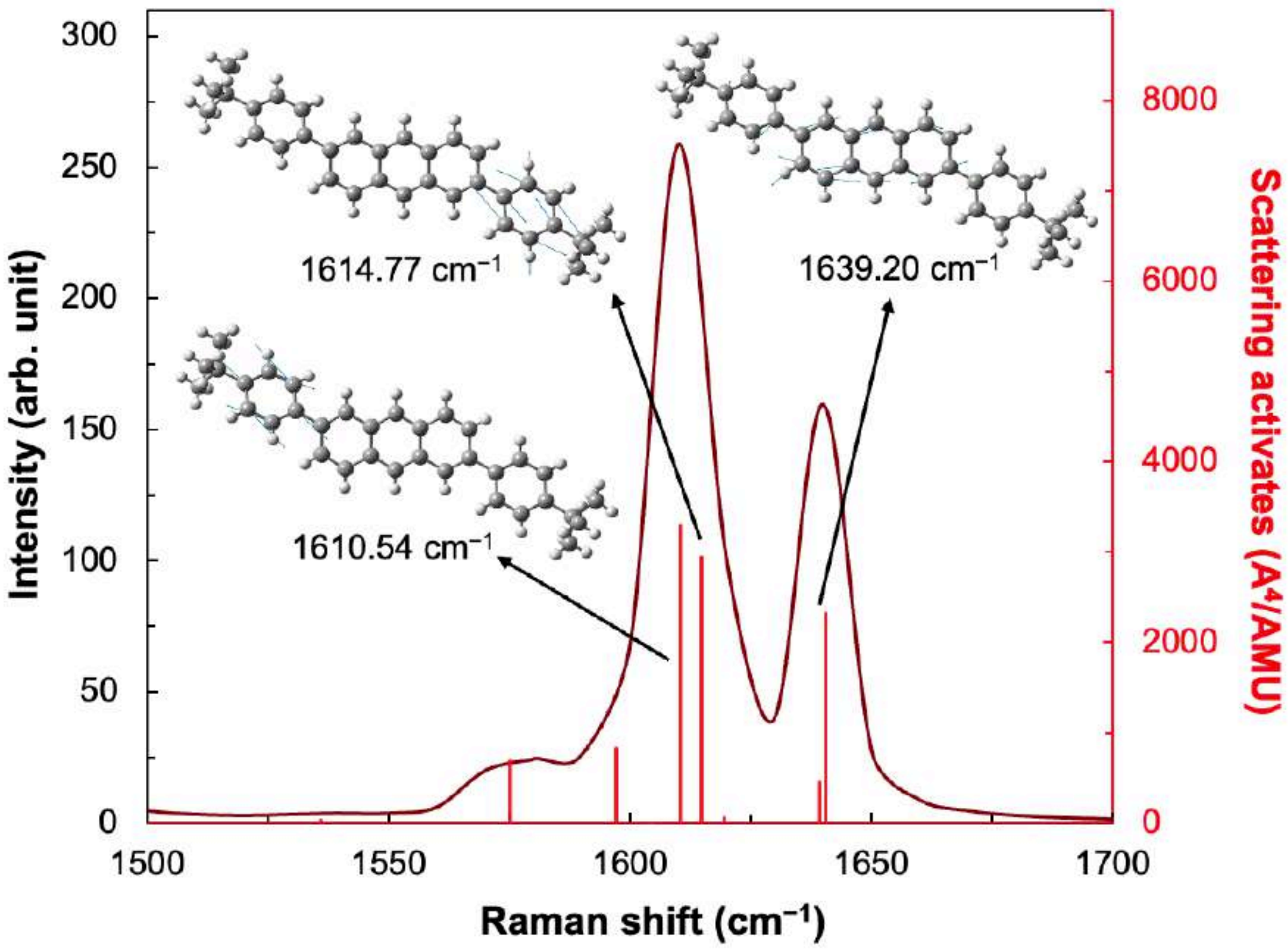

Figure S28. Calculated Raman spectrum of the red conformer of **BBPA** (CCDC 2532253). The dark red line shows the calculated Raman spectrum, while the light-red line represents the corresponding Raman activities. The molecular structure is overlaid with representative vibrational modes, indicated by blue displacement vectors.

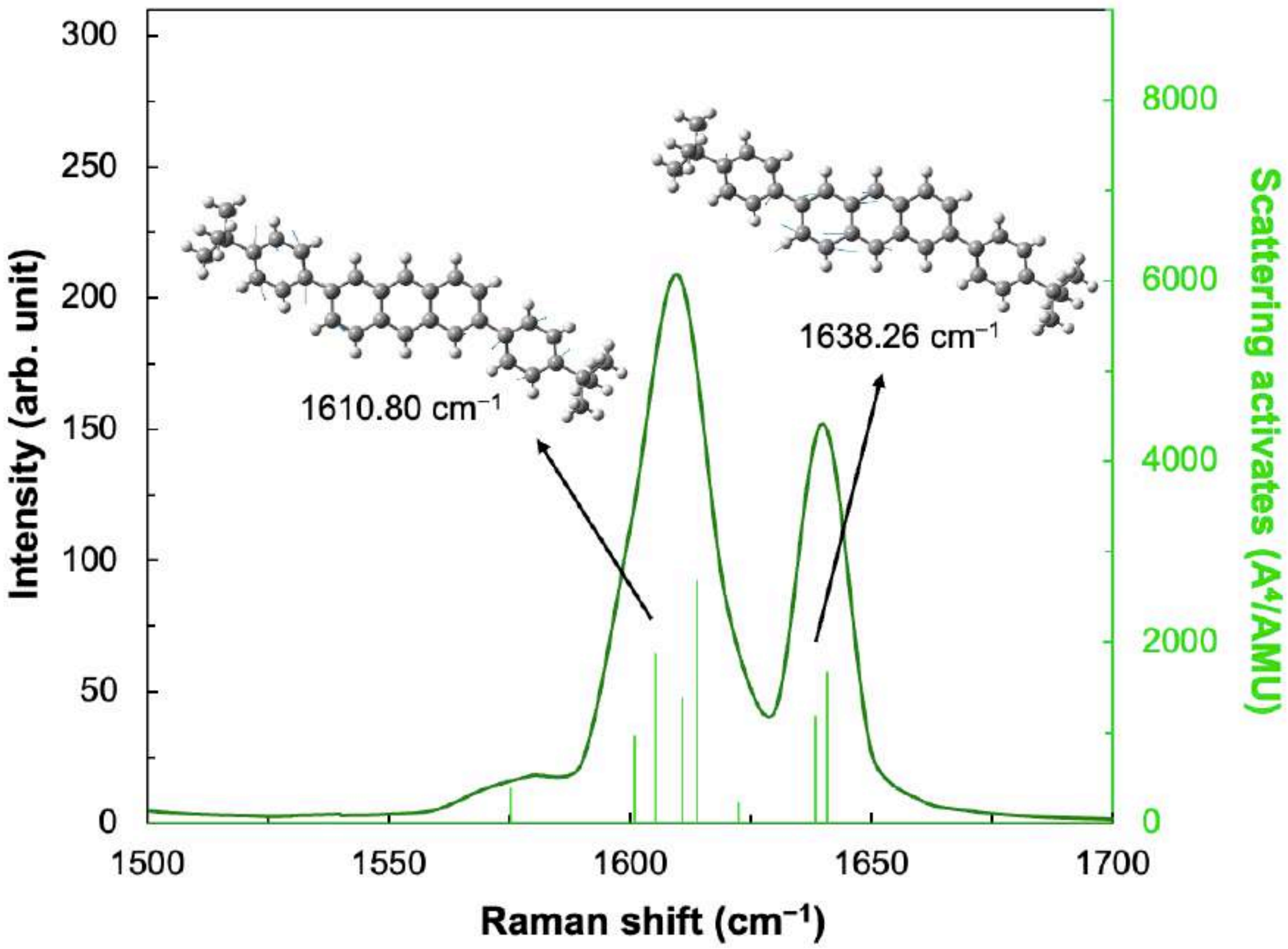

Figure S29. Calculated Raman spectrum of the green conformer of **BBPA** (CCDC 2532253). The dark green line shows the calculated Raman spectrum, while the light-green line represents the corresponding Raman activities. The molecular structure is overlaid with representative vibrational modes, indicated by blue displacement vectors.

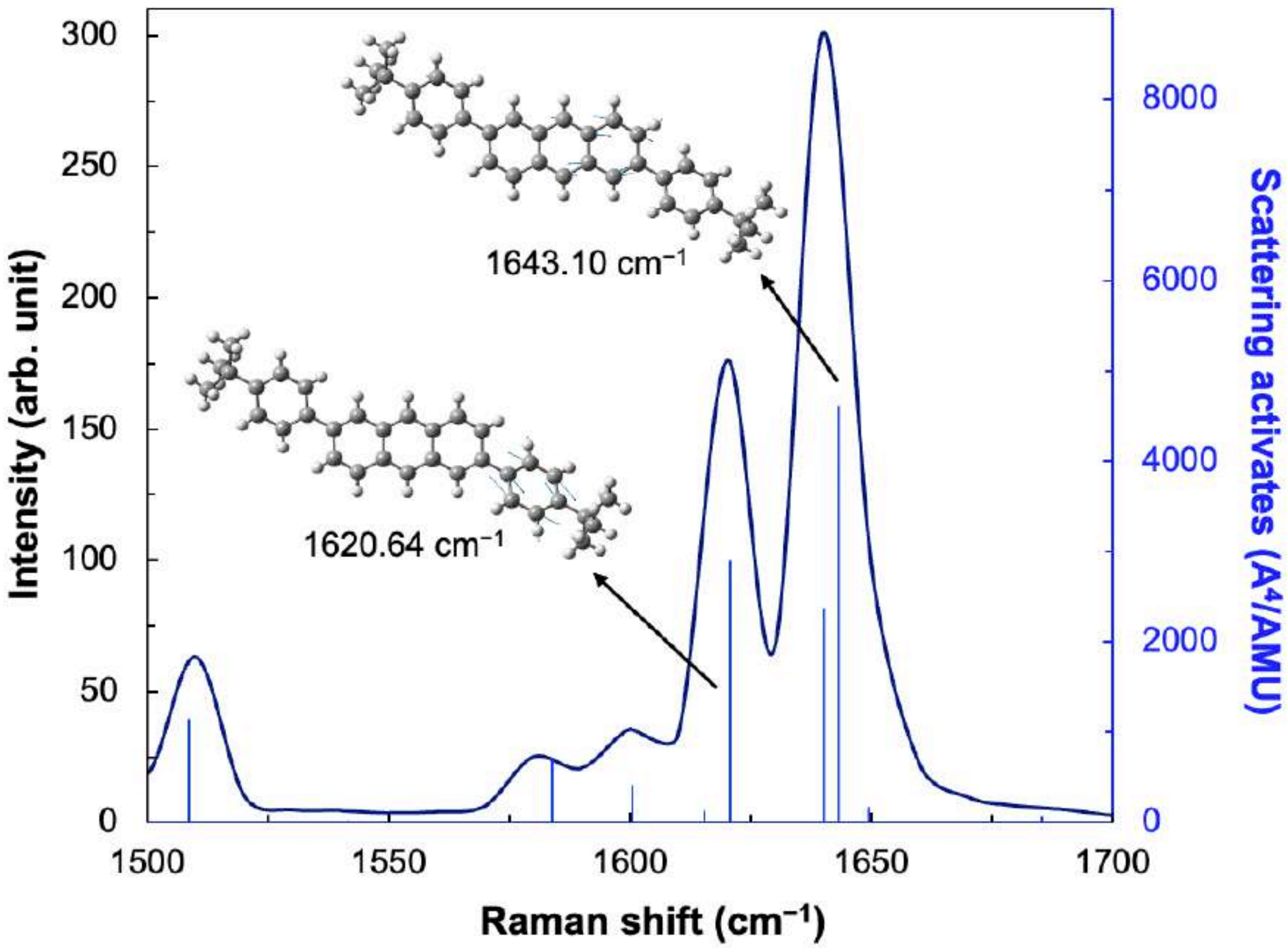

Figure S30. Calculated Raman spectrum of the blue conformer of **BBPA** (CCDC 2532253). The dark blue line shows the calculated Raman spectrum, while the light-blue line represents the corresponding Raman activities. The molecular structure is overlaid with representative vibrational modes, indicated by blue displacement vectors.

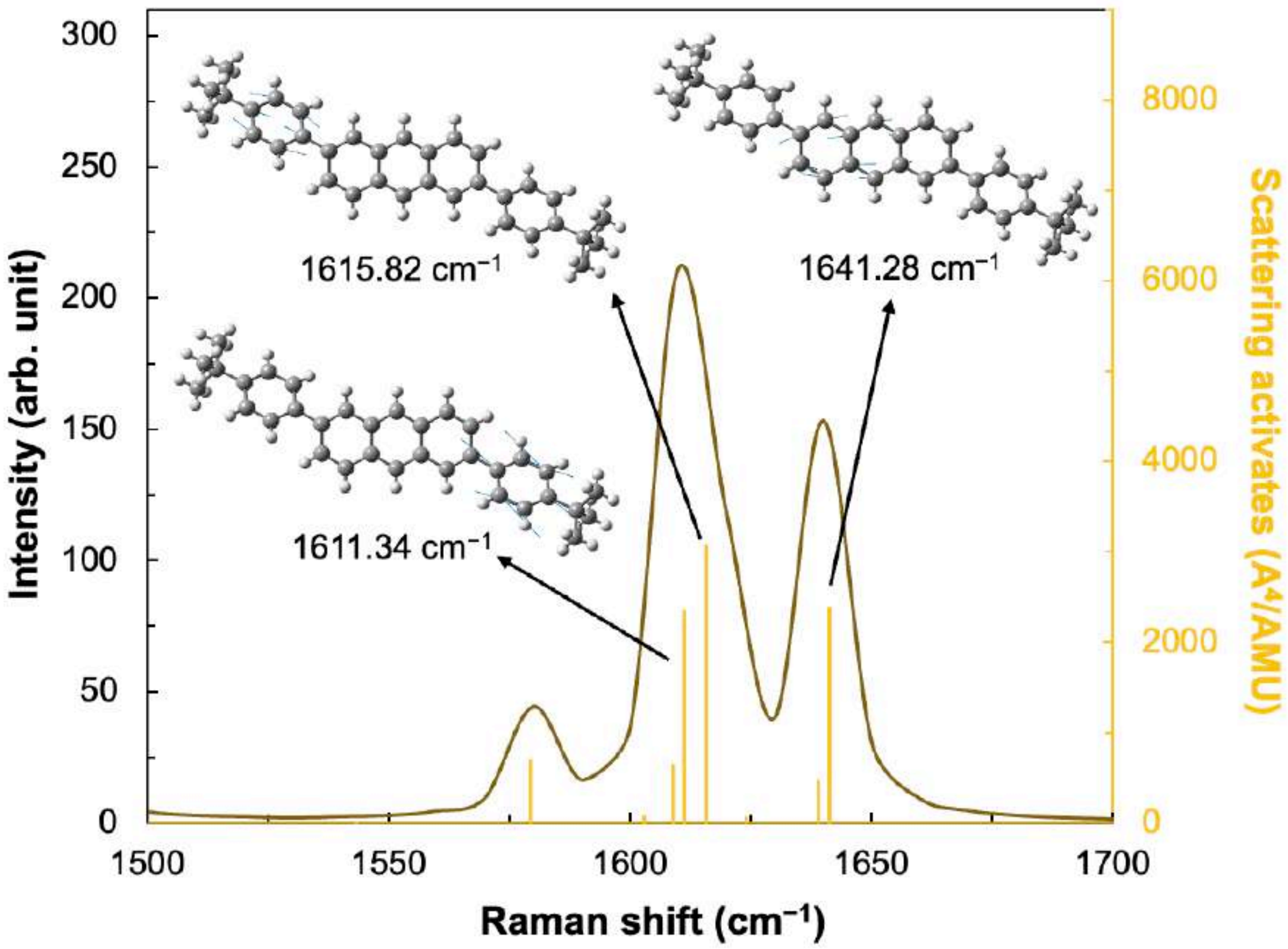

Figure S31. Calculated Raman spectrum of the yellow conformer of **BBPA** (CCDC 2532253). The dark yellow line shows the calculated Raman spectrum, while the light-yellow line represents the corresponding Raman activities. The molecular structure is overlaid with representative vibrational modes, indicated by blue displacement vectors.

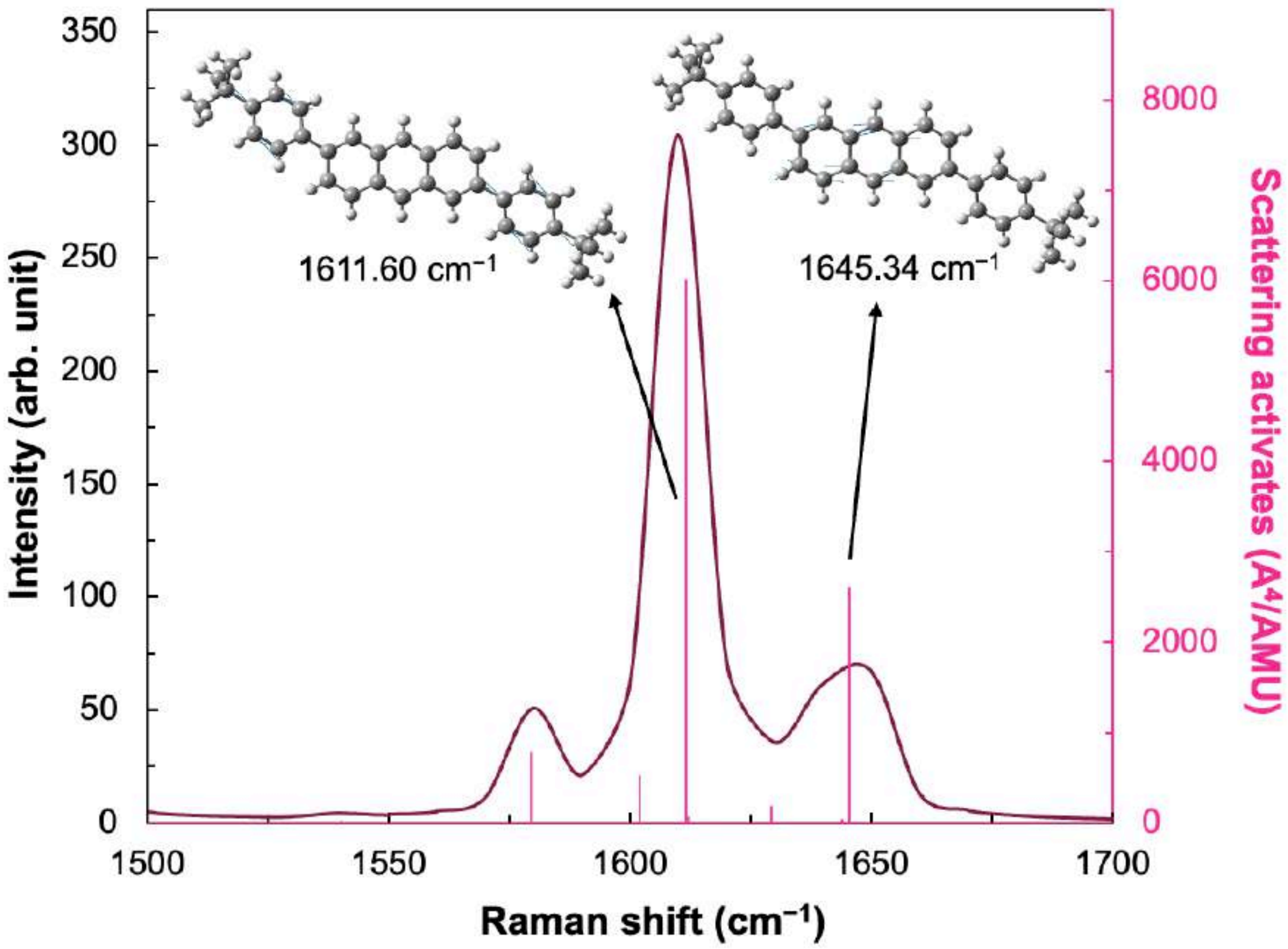

Figure S32. Calculated Raman spectrum of the pink conformer of **BBPA** (CCDC 2532253). The dark pink line shows the calculated Raman spectrum, while the light-pink line represents the corresponding Raman activities. The molecular structure is overlaid with representative vibrational modes, indicated by blue displacement vectors.

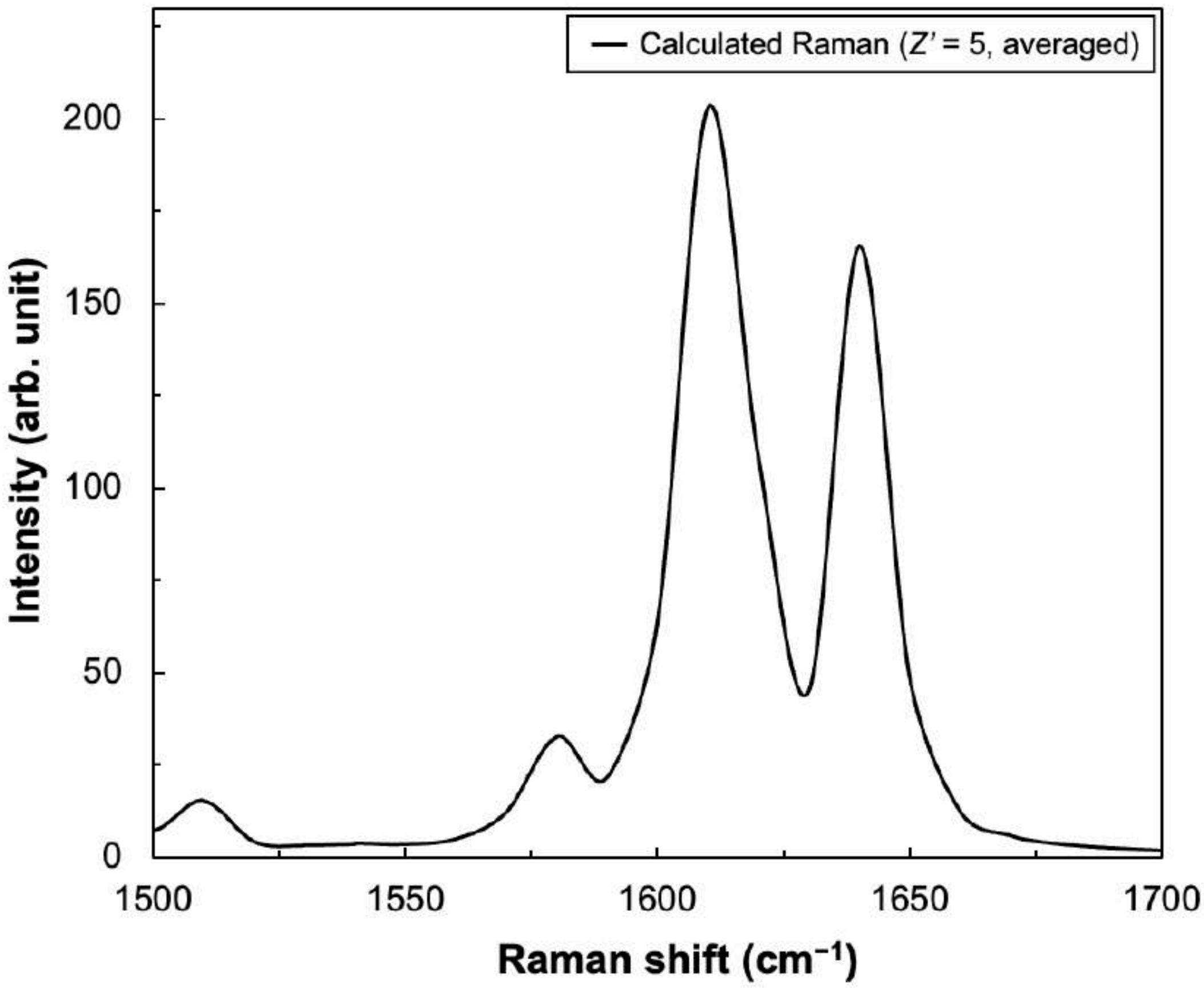

Figure S33. The calculated Raman spectrum of **BBPA** corresponds to the average of spectra independently calculated for the five crystallographically independent molecules.

4.2 Temperature-dependent Raman spectra

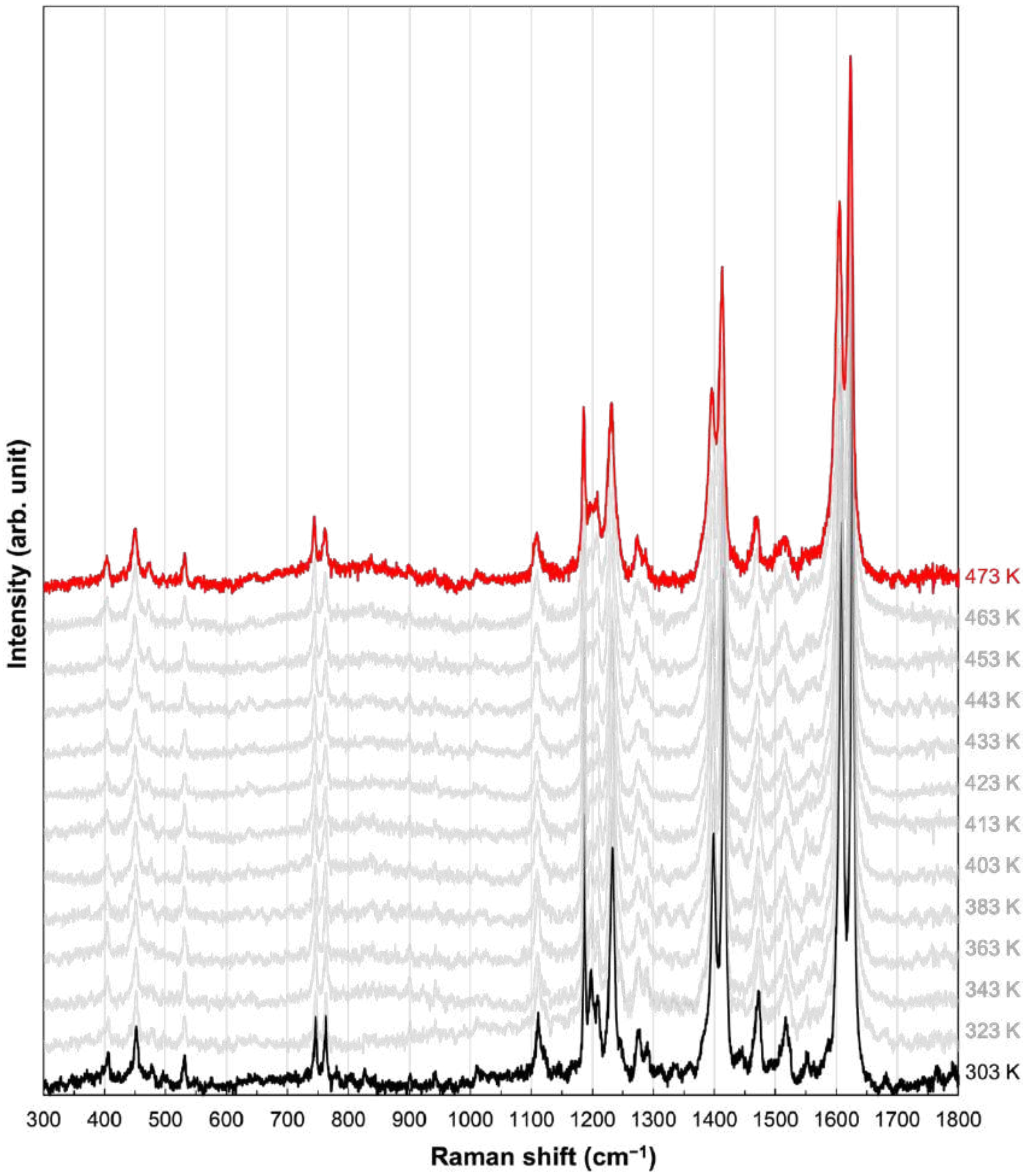

Figure S34. VT-Raman spectra of **BBPA** in the 1550 cm^{-1}–1700 cm^{-1} region recorded for the single-crystal sample during heating from 303 K to 473 K.

4.3 Peak shifts and FWHM analysis

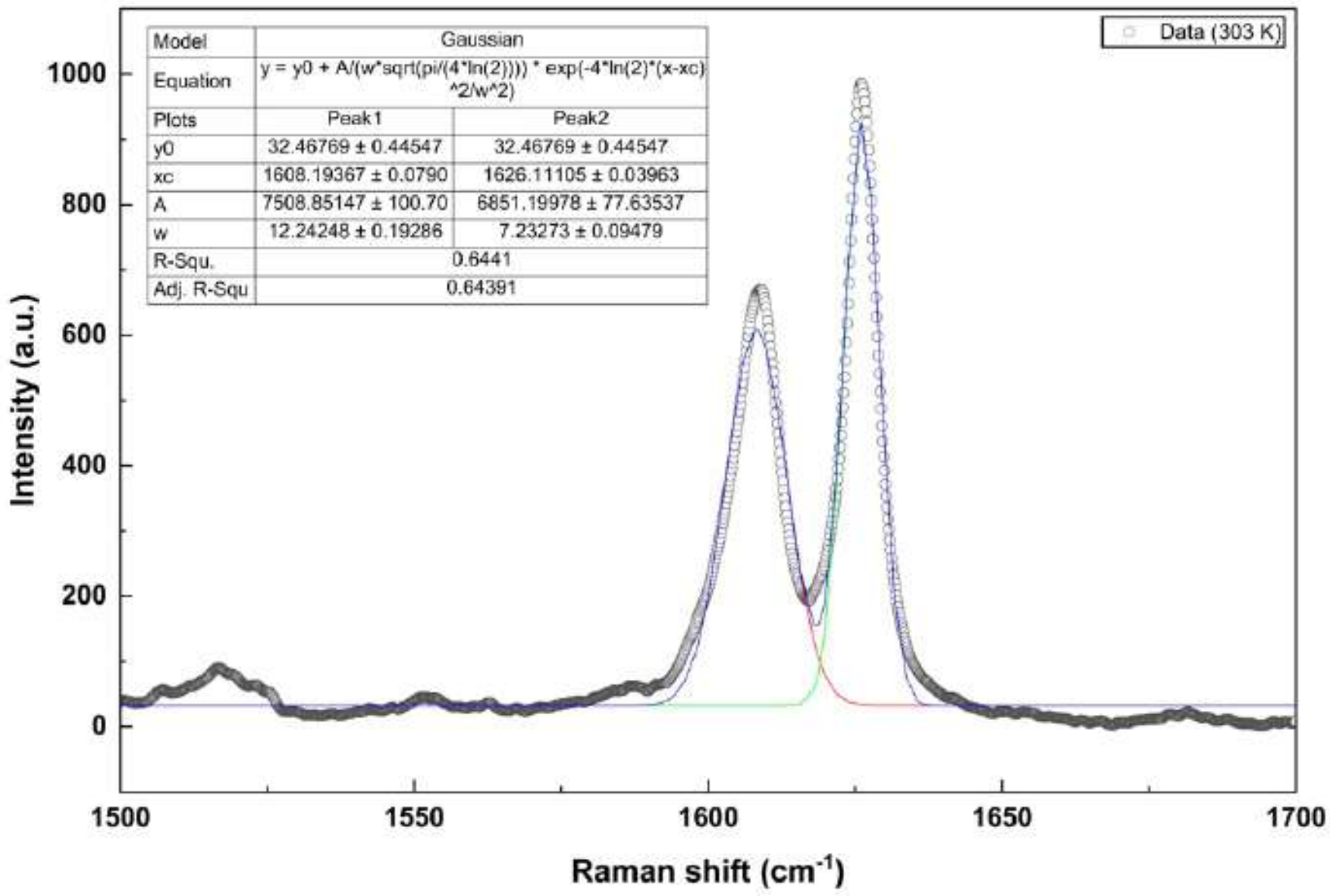

Figure S35. Gaussian fitting of the Raman spectrum of a **BBPA** crystal in the 1500 cm^{-1}–1700 cm^{-1} region at 303 K. Open circles represent the experimental spectrum, while the red and green curves correspond to the individual Gaussian components (Peaks 1 and 2). The blue curve represents the overall fitted profile.

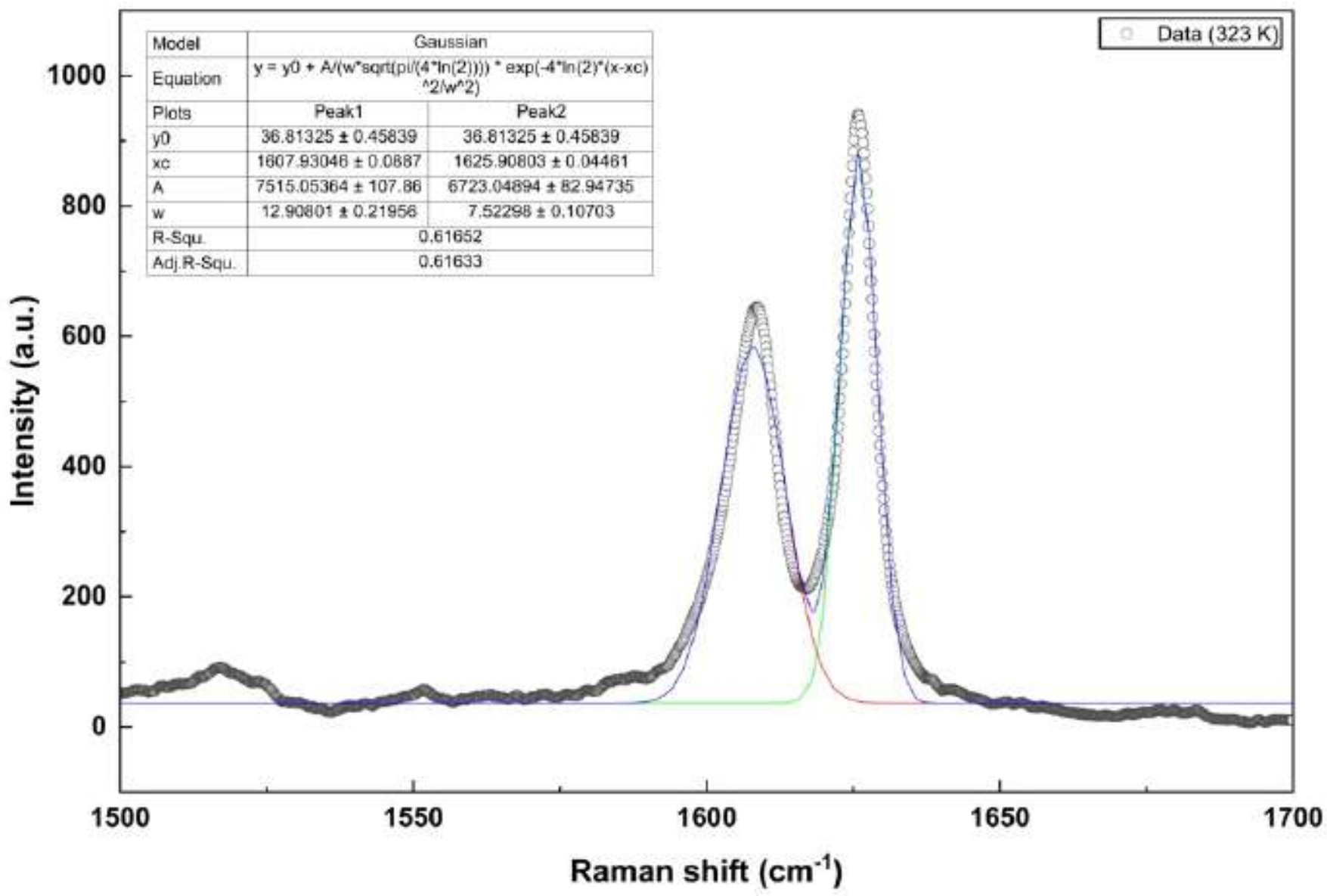

Figure S36. Gaussian fitting of the Raman spectrum of a **BBPA** crystal in the 1500 cm^{-1}–1700 cm^{-1} region at 323 K. Open circles represent the experimental spectrum, while the red and green curves correspond to the individual Gaussian components (Peaks 1 and 2). The blue curve represents the overall fitted profile.

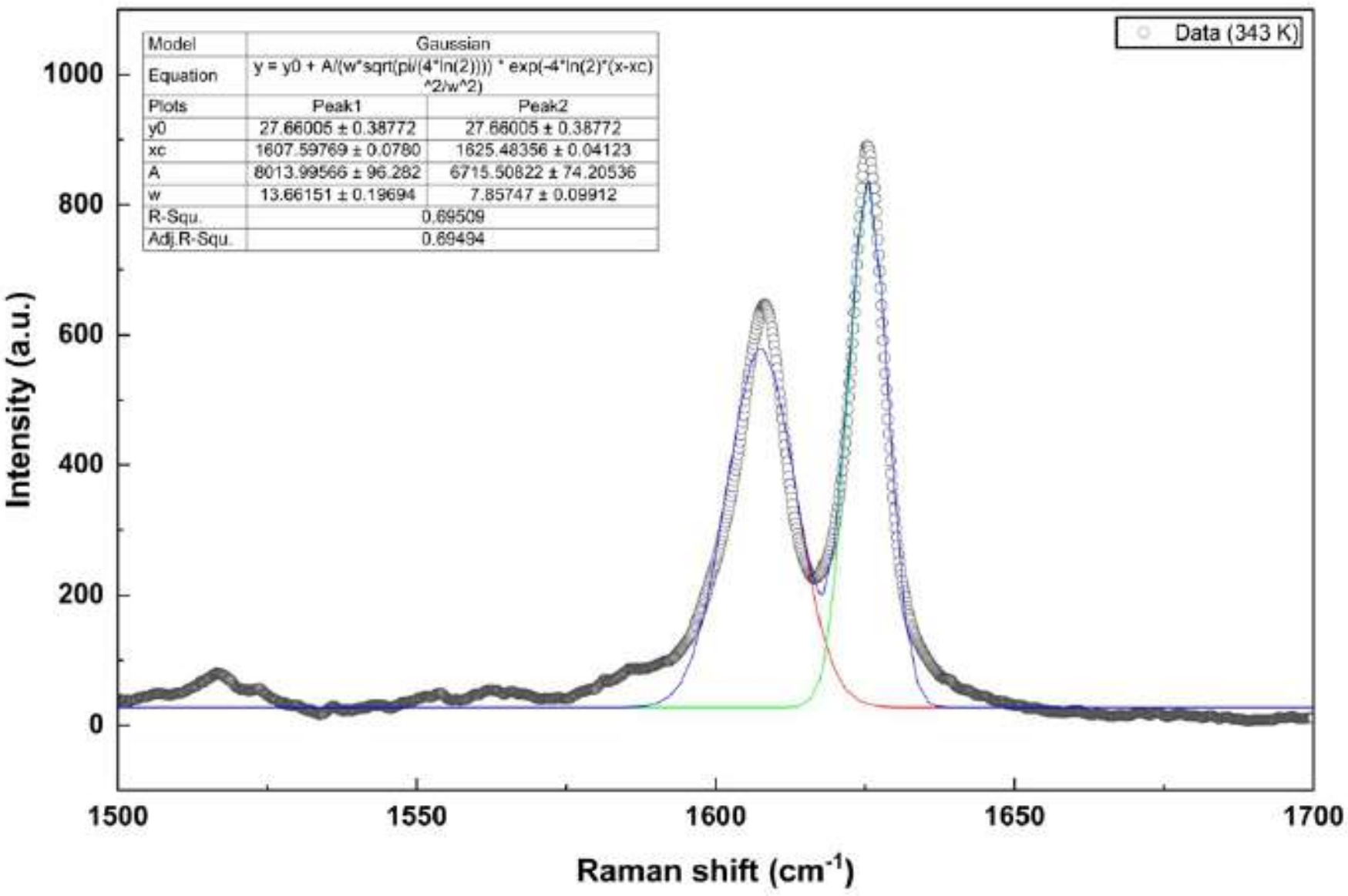

Figure S37. Gaussian fitting of the Raman spectrum of a **BBPA** crystal in the 1500 cm^{-1}–1700 cm^{-1} region at 343 K. Open circles represent the experimental spectrum, while the red and green curves correspond to the individual Gaussian components (Peaks 1 and 2). The blue curve represents the overall fitted profile.

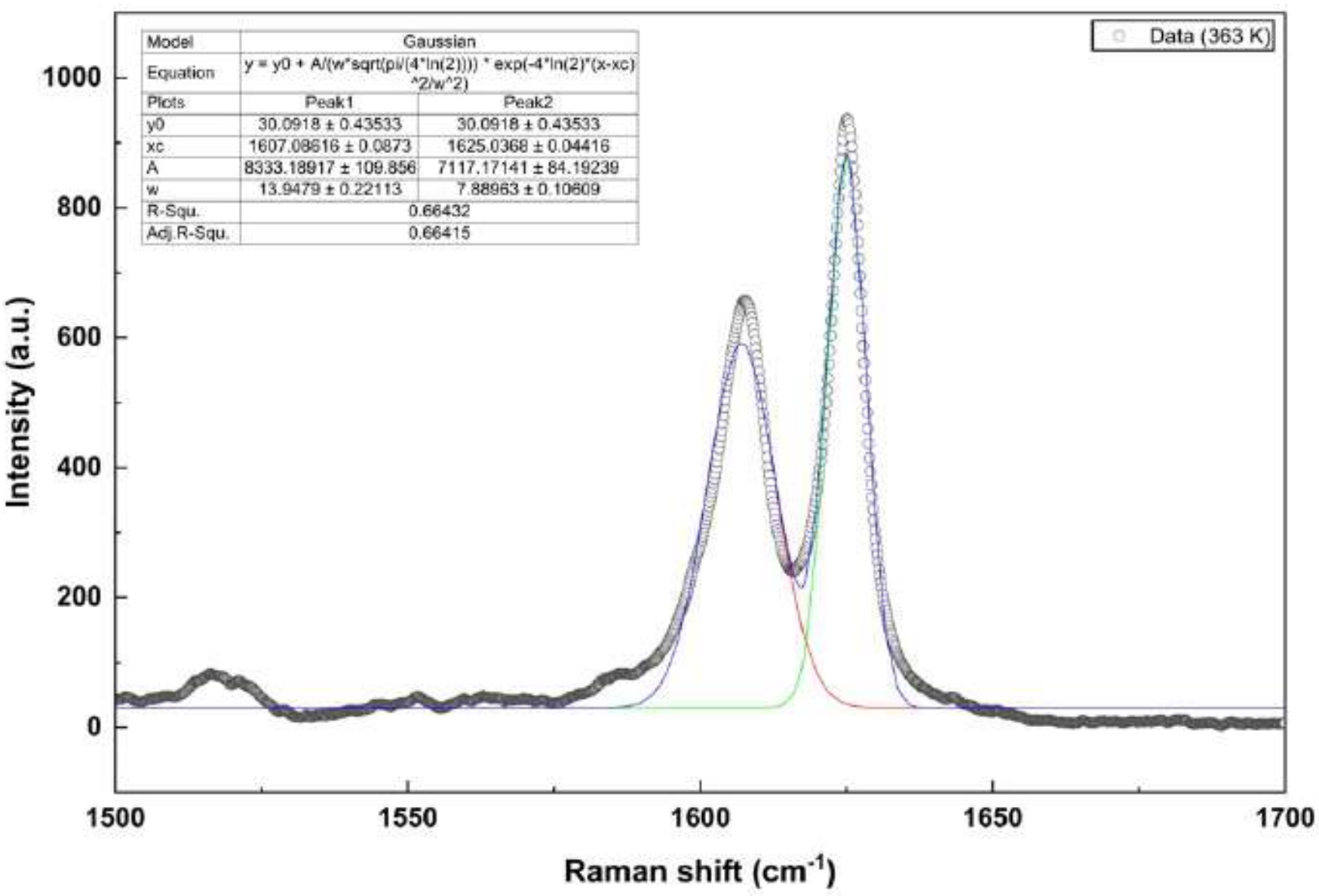

Figure S38. Gaussian fitting of the Raman spectrum of a **BBPA** crystal in the 1500 cm^{-1}–1700 cm^{-1} region at 363 K. Open circles represent the experimental spectrum, while the red and green curves correspond to the individual Gaussian components (Peaks 1 and 2). The blue curve represents the overall fitted profile.

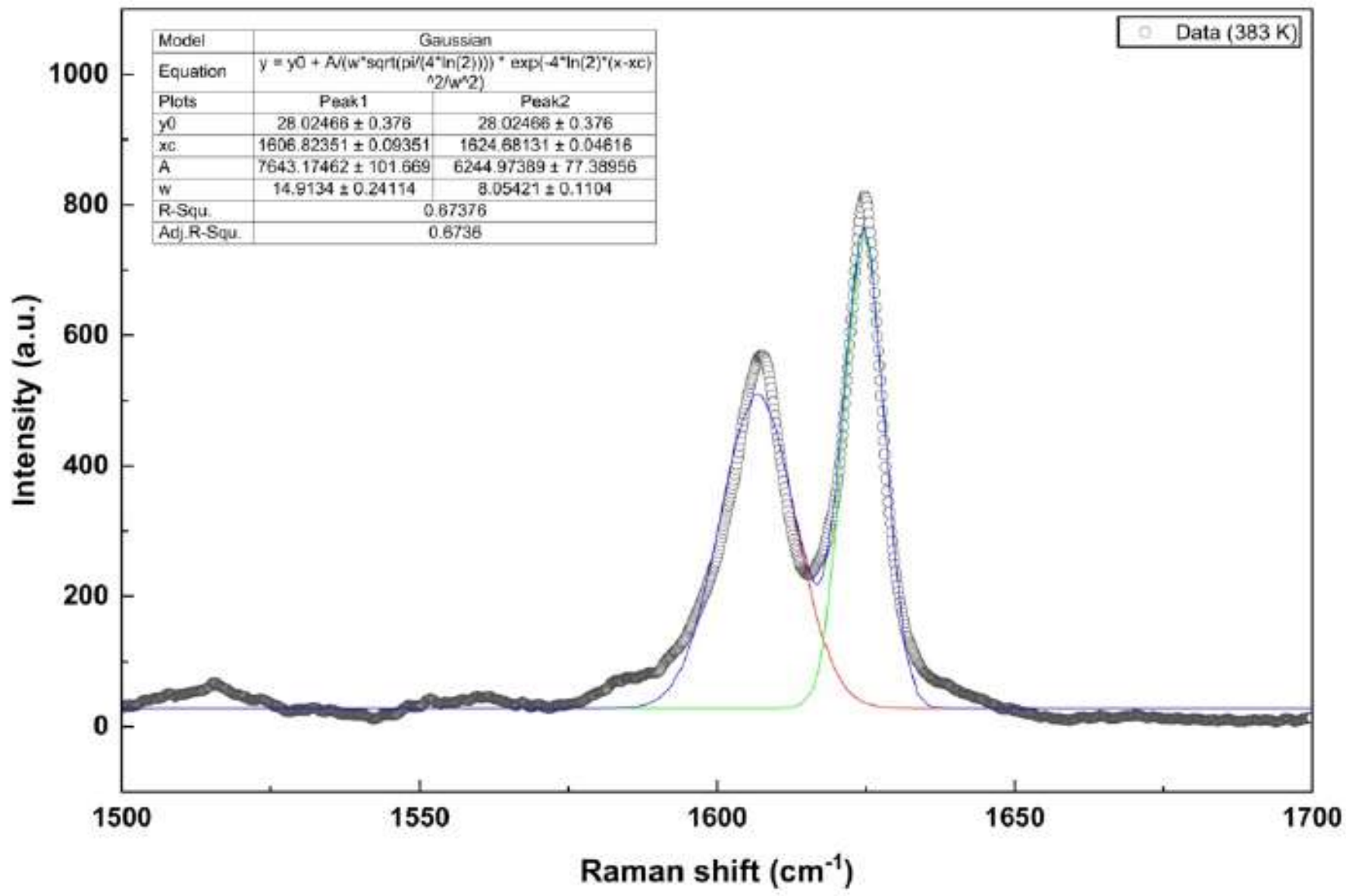

Figure S39. Gaussian fitting of the Raman spectrum of a **BBPA** crystal in the 1500 cm^{-1}–1700 cm^{-1} region at 383 K. Open circles represent the experimental spectrum, while the red and green curves correspond to the individual Gaussian components (Peaks 1 and 2). The blue curve represents the overall fitted profile.

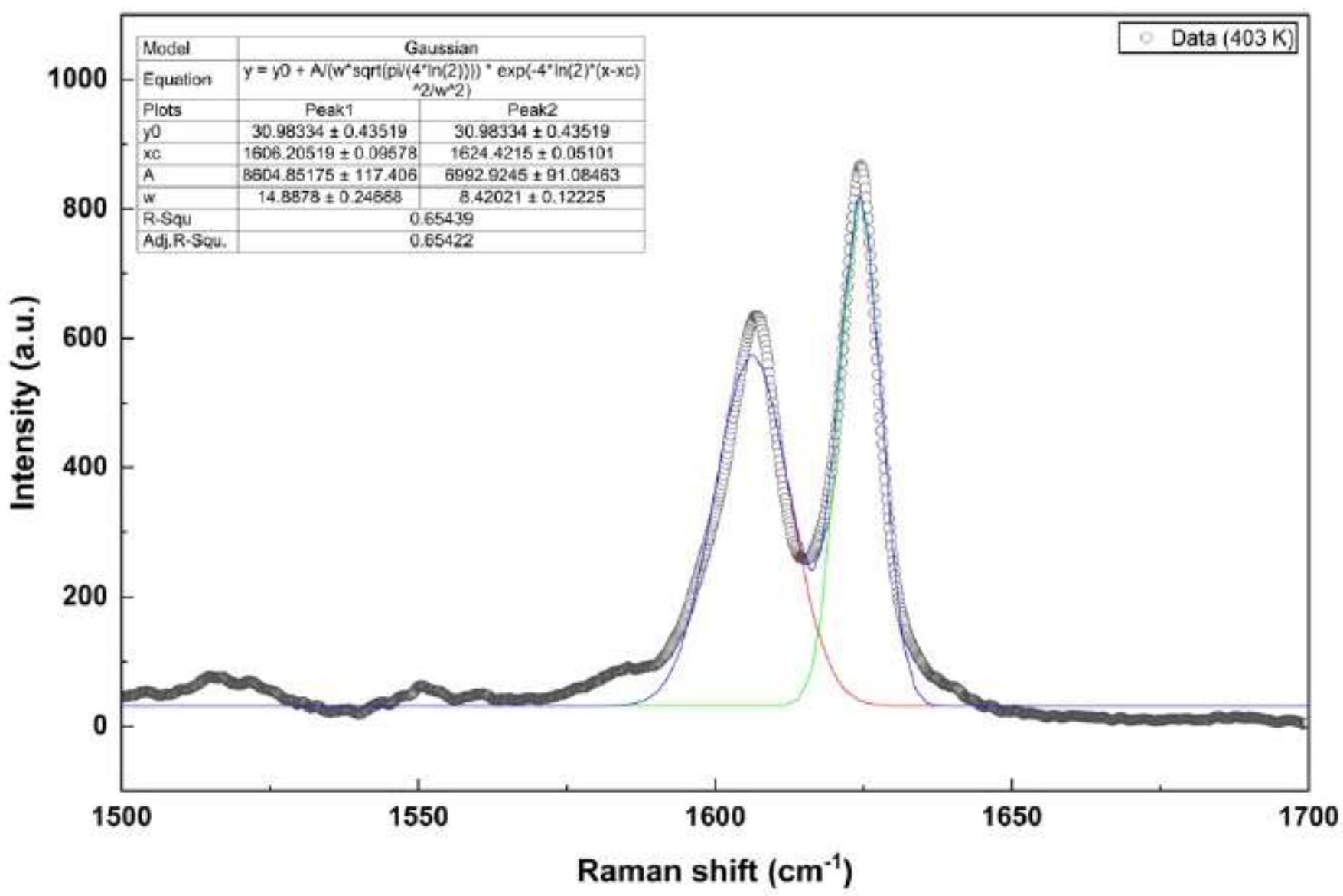

Figure S40. Gaussian fitting of the Raman spectrum of a **BBPA** crystal in the 1500 cm^{-1}–1700 cm^{-1} region at 403 K. Open circles represent the experimental spectrum, while the red and green curves correspond to the individual Gaussian components (Peaks 1 and 2). The blue curve represents the overall fitted profile.

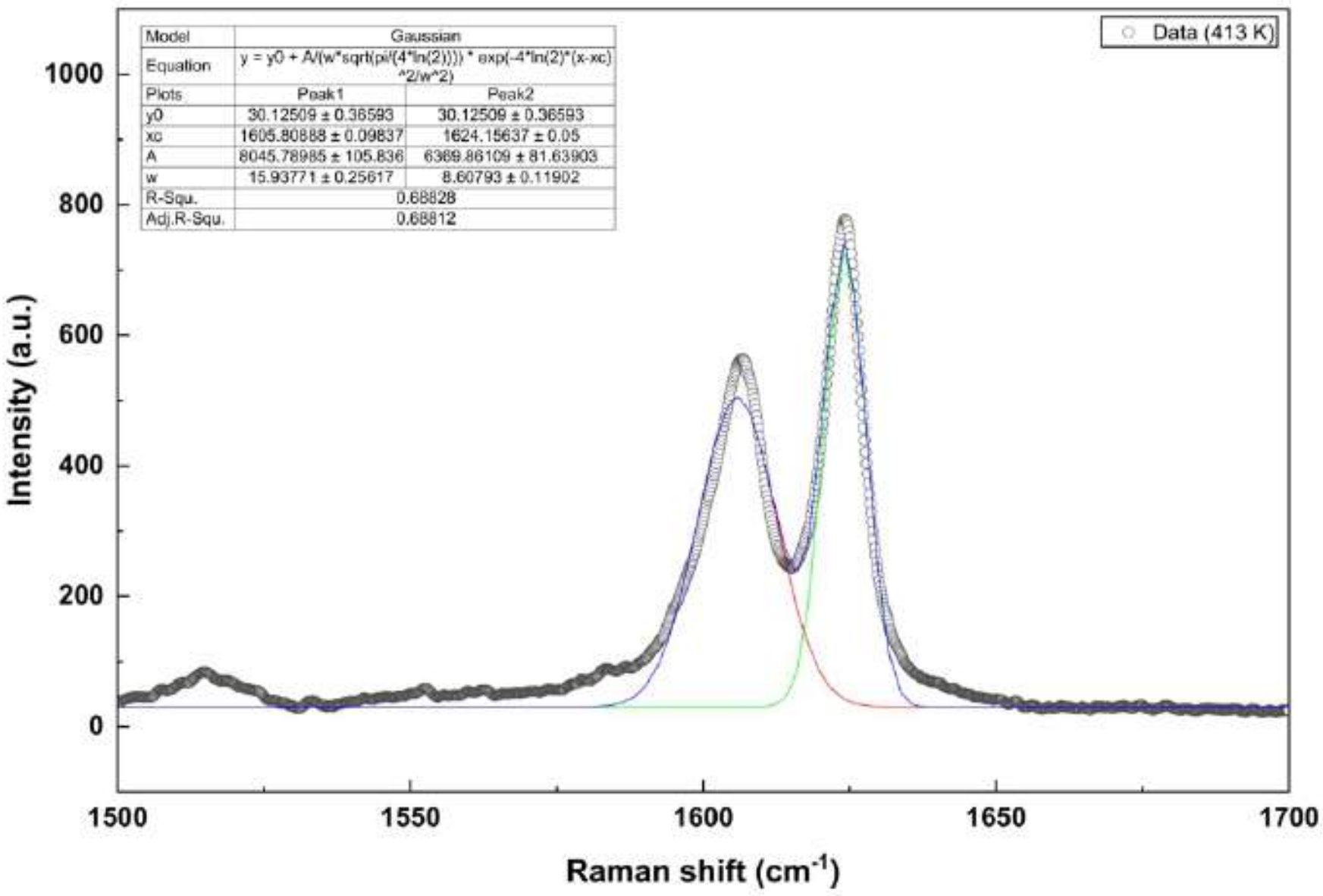

Figure S41. Gaussian fitting of the Raman spectrum of a **BBPA** crystal in the 1500 cm^{-1}–1700 cm^{-1} region at 413 K. Open circles represent the experimental spectrum, while the red and green curves correspond to the individual Gaussian components (Peaks 1 and 2). The blue curve represents the overall fitted profile.

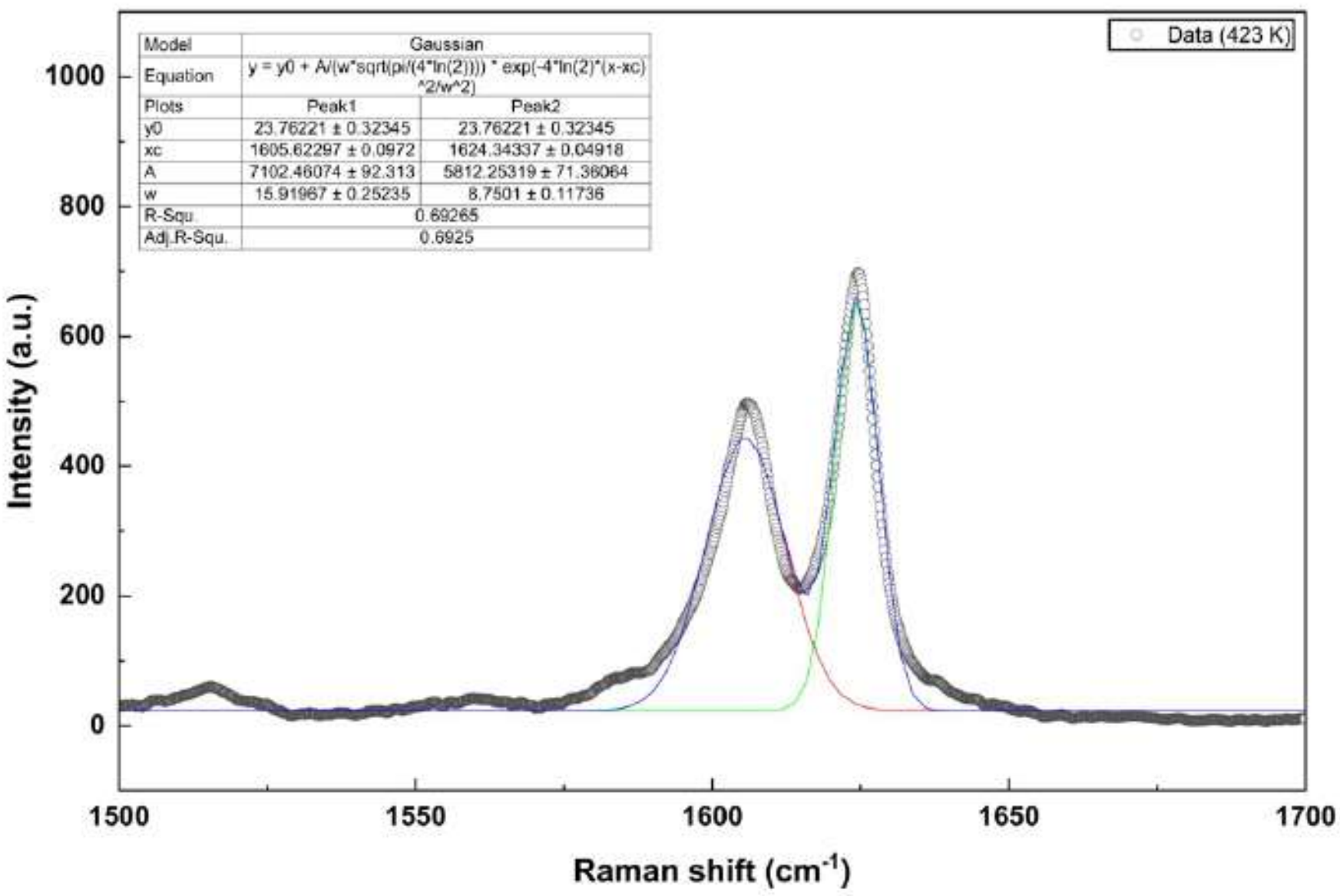

Figure S42. Gaussian fitting of the Raman spectrum of a **BBPA** crystal in the 1500 cm^{-1}–1700 cm^{-1} region at 423 K. Open circles represent the experimental spectrum, while the red and green curves correspond to the individual Gaussian components (Peaks 1 and 2). The blue curve represents the overall fitted profile.

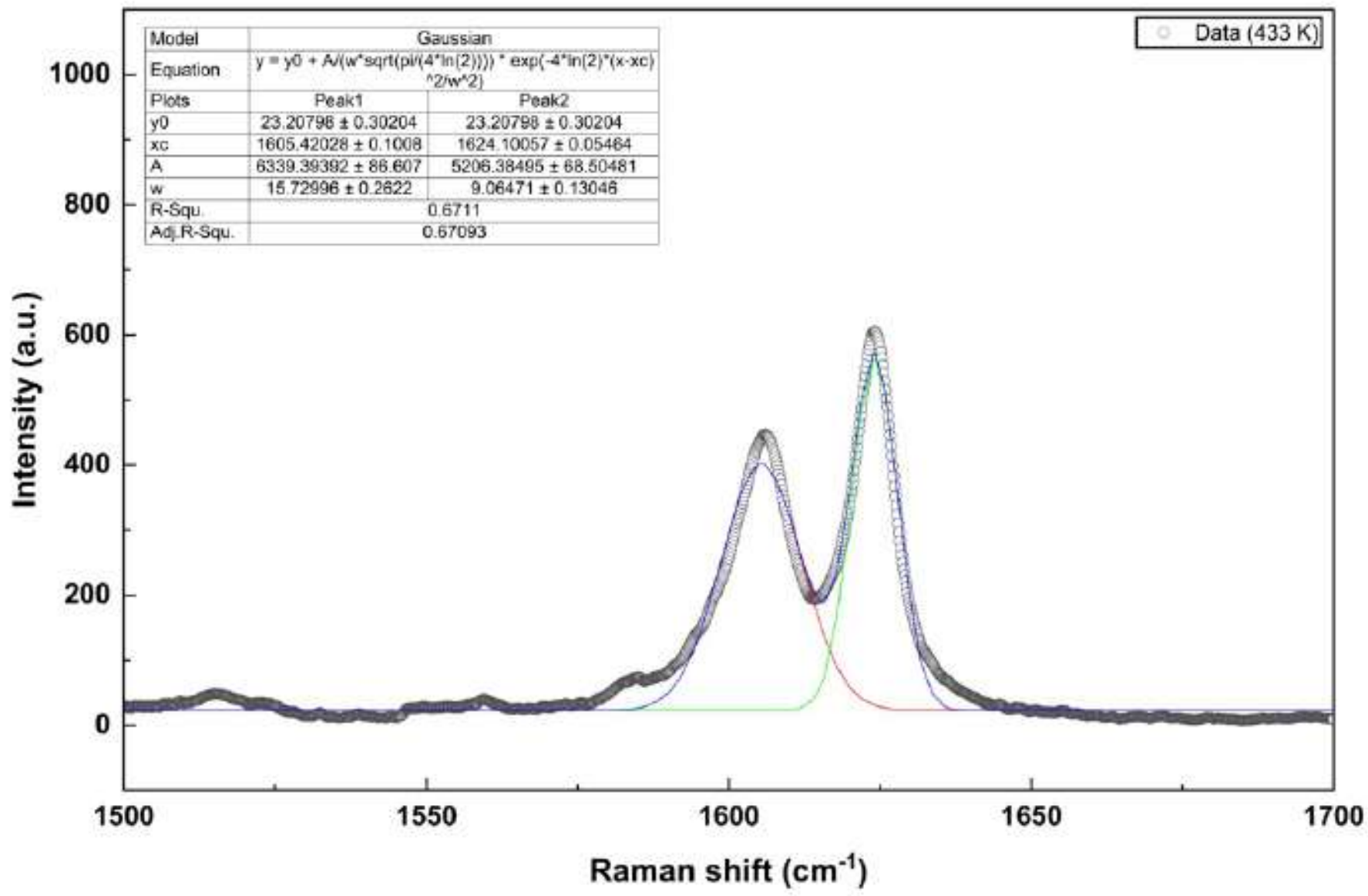

Figure S43. Gaussian fitting of the Raman spectrum of a **BBPA** crystal in the 1500 cm^{-1}–1700 cm^{-1} region at 433 K. Open circles represent the experimental spectrum, while the red and green curves correspond to the individual Gaussian components (Peaks 1 and 2). The blue curve represents the overall fitted profile.

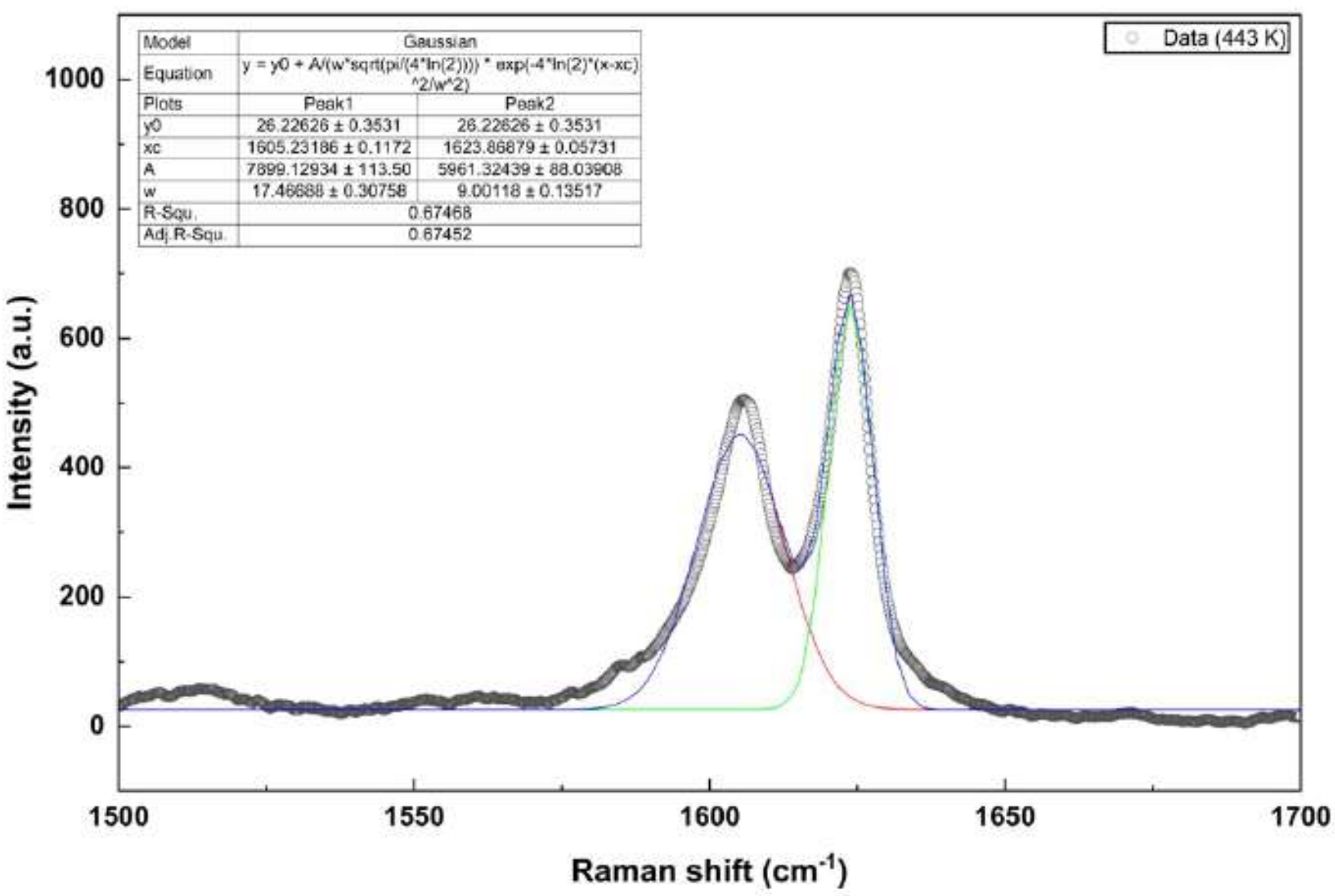

Figure S44. Gaussian fitting of the Raman spectrum of a **BBPA** crystal in the 1500 cm^{-1}–1700 cm^{-1} region at 443 K. Open circles represent the experimental spectrum, while the red and green curves correspond to the individual Gaussian components (Peaks 1 and 2). The blue curve represents the overall fitted profile.

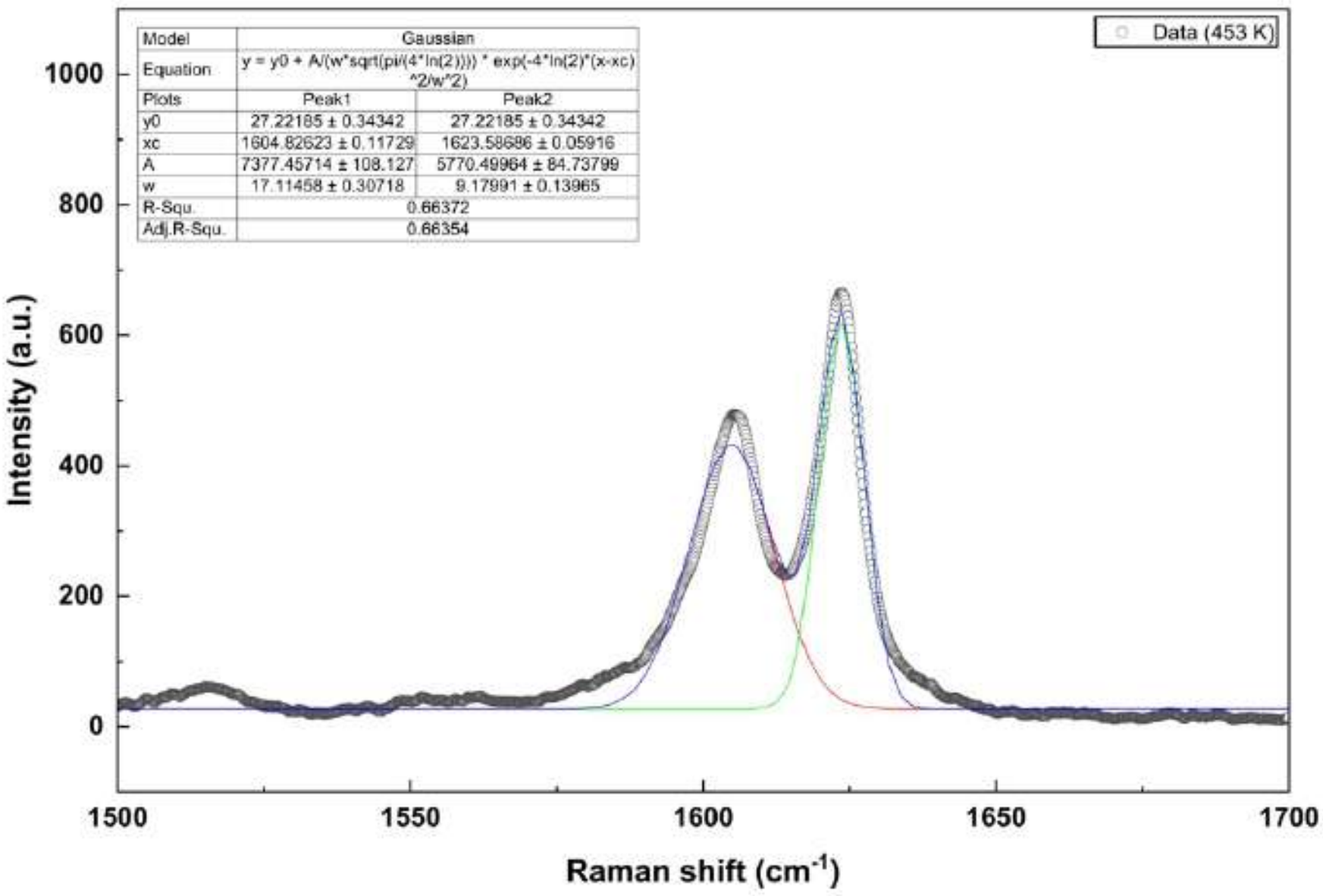

Figure S45. Gaussian fitting of the Raman spectrum of a **BBPA** crystal in the 1500 cm^{-1}–1700 cm^{-1} region at 453 K. Open circles represent the experimental spectrum, while the red and green curves correspond to the individual Gaussian components (Peaks 1 and 2). The blue curve represents the overall fitted profile.

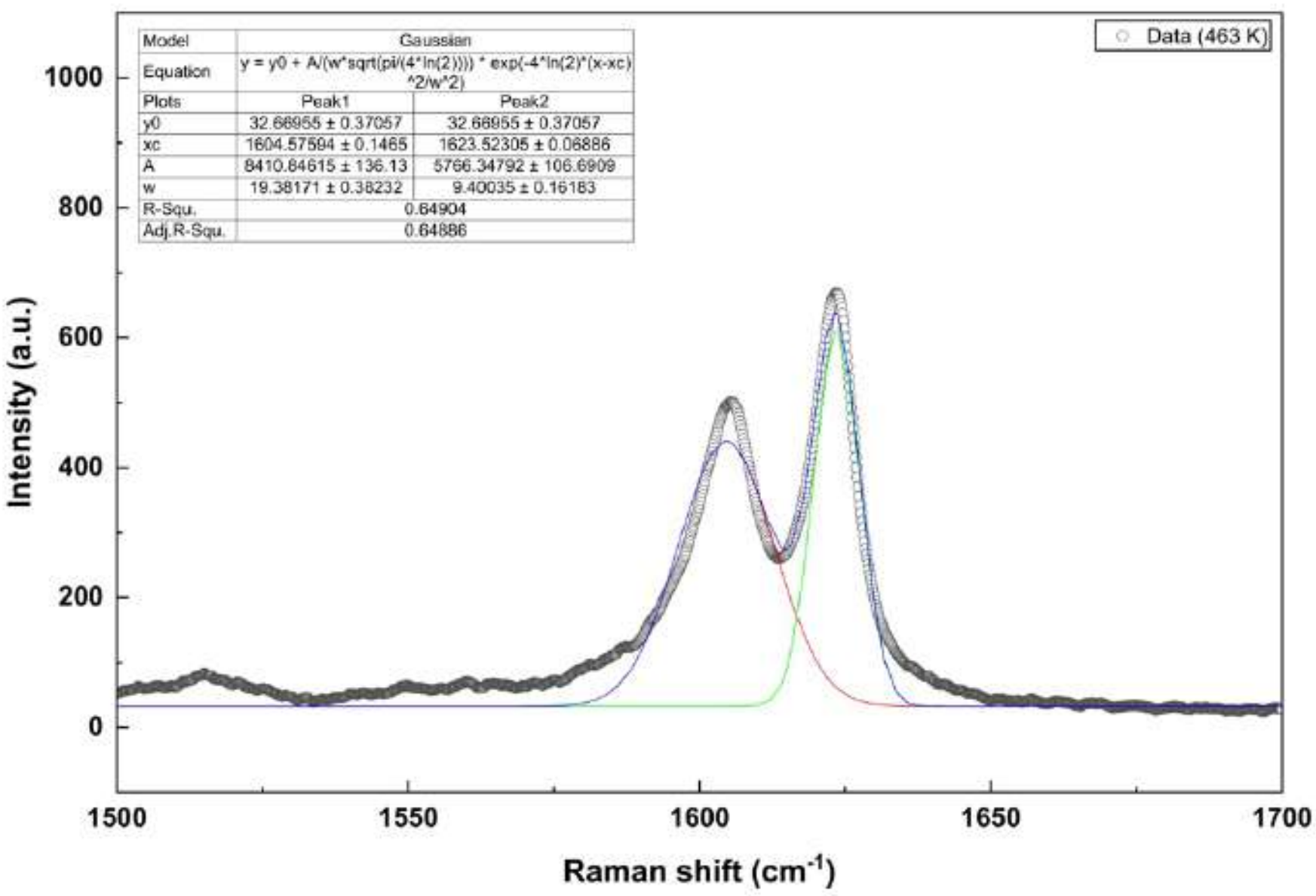

Figure S46. Gaussian fitting of the Raman spectrum of a **BBPA** crystal in the 1500 cm^{-1}–1700 cm^{-1} region at 463 K. Open circles represent the experimental spectrum, while the red and green curves correspond to the individual Gaussian components (Peaks 1 and 2). The blue curve represents the overall fitted profile.

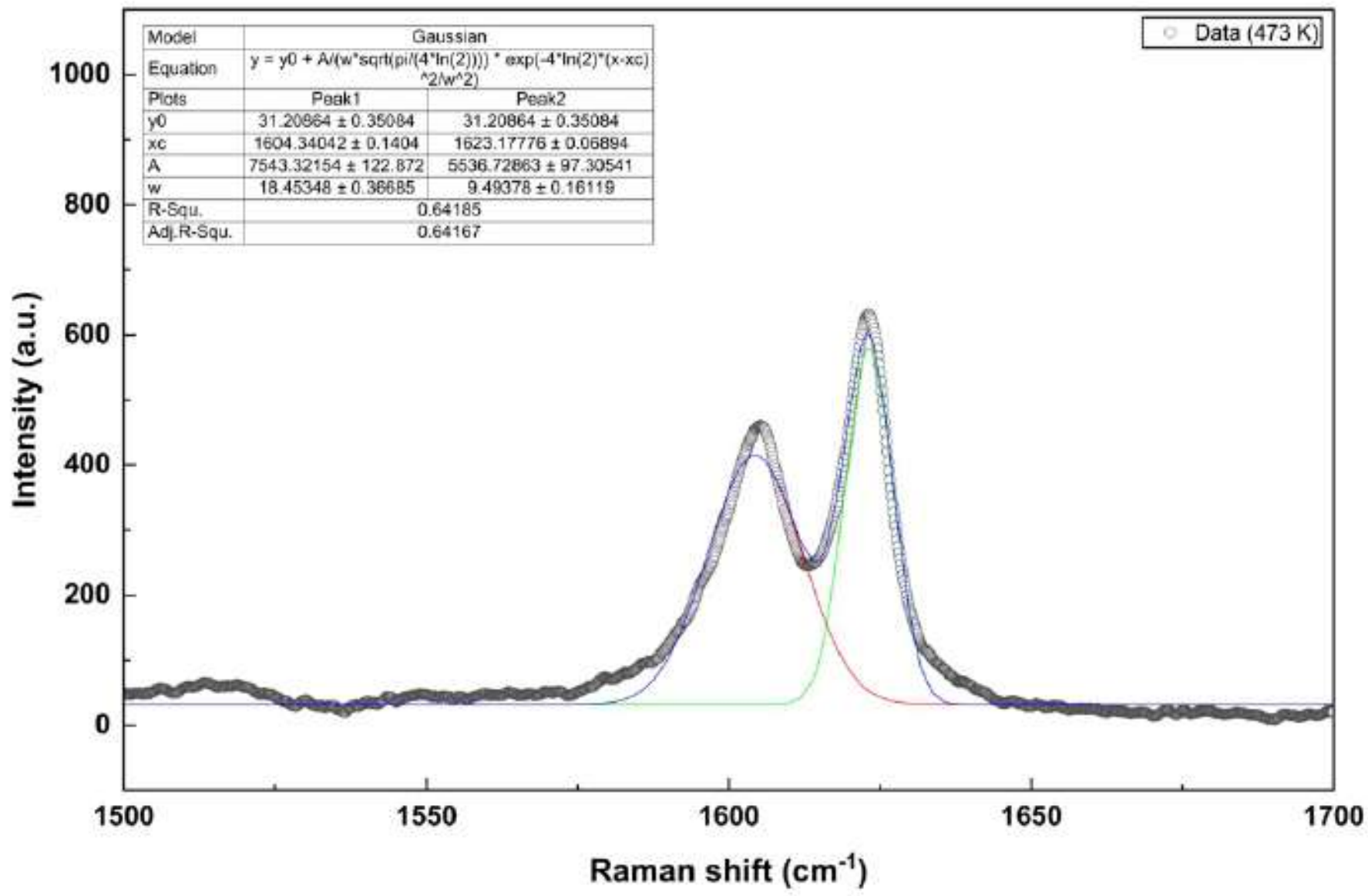

Figure S47. Gaussian fitting of the Raman spectrum of a **BBPA** crystal in the 1500 cm^{-1}–1700 cm^{-1} region at 473 K. Open circles represent the experimental spectrum, while the red and green curves correspond to the individual Gaussian components (Peaks 1 and 2). The blue curve represents the overall fitted profile.

5. Effect of grinding on the thermal phase transition

5.1 Mechanical grinding procedure

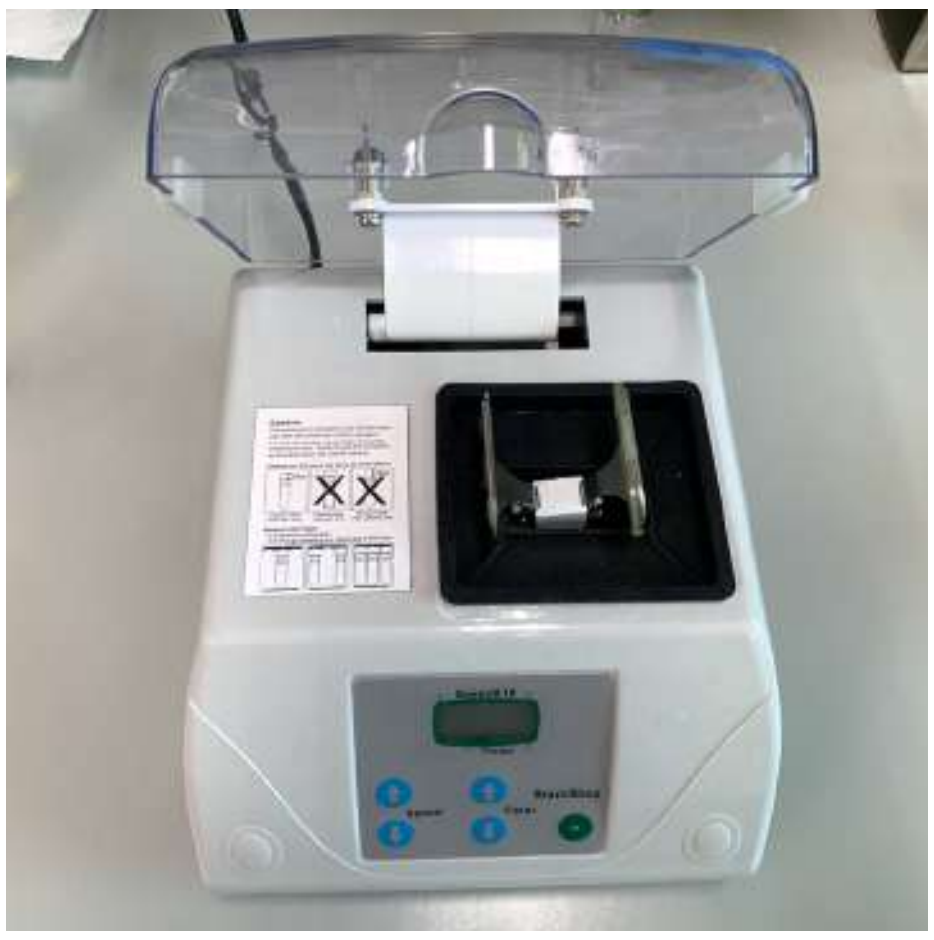

Figure S48. SHAKE MAN 3 used in this study.

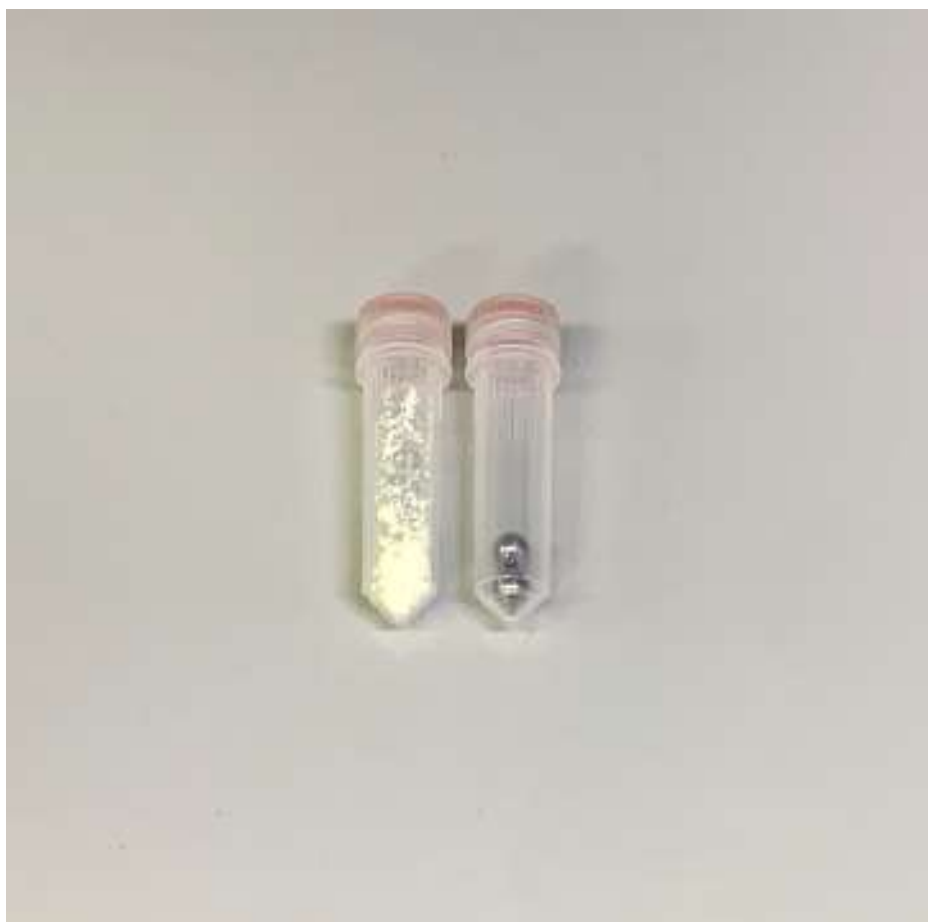

Figure S49. Tube and stainless-steel ball used in this study.

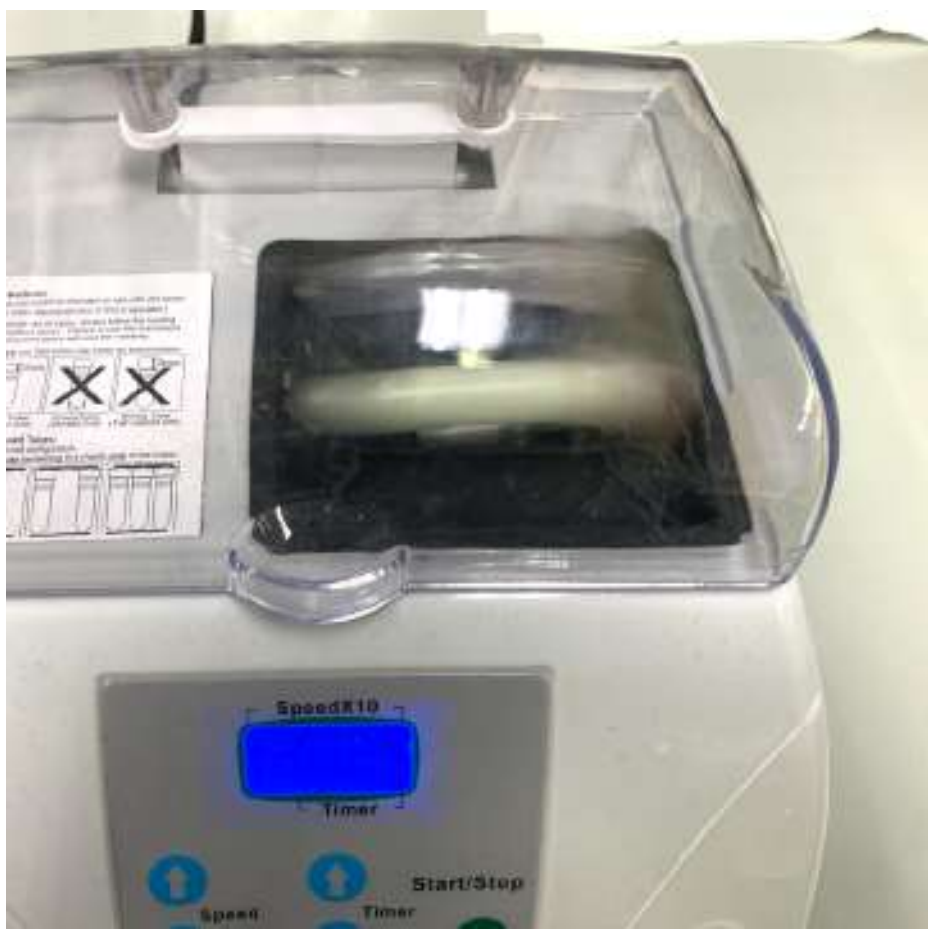

Figure S50. Mechanical grinding process.

5.2 Evaluation of crystallinity and crystallite size

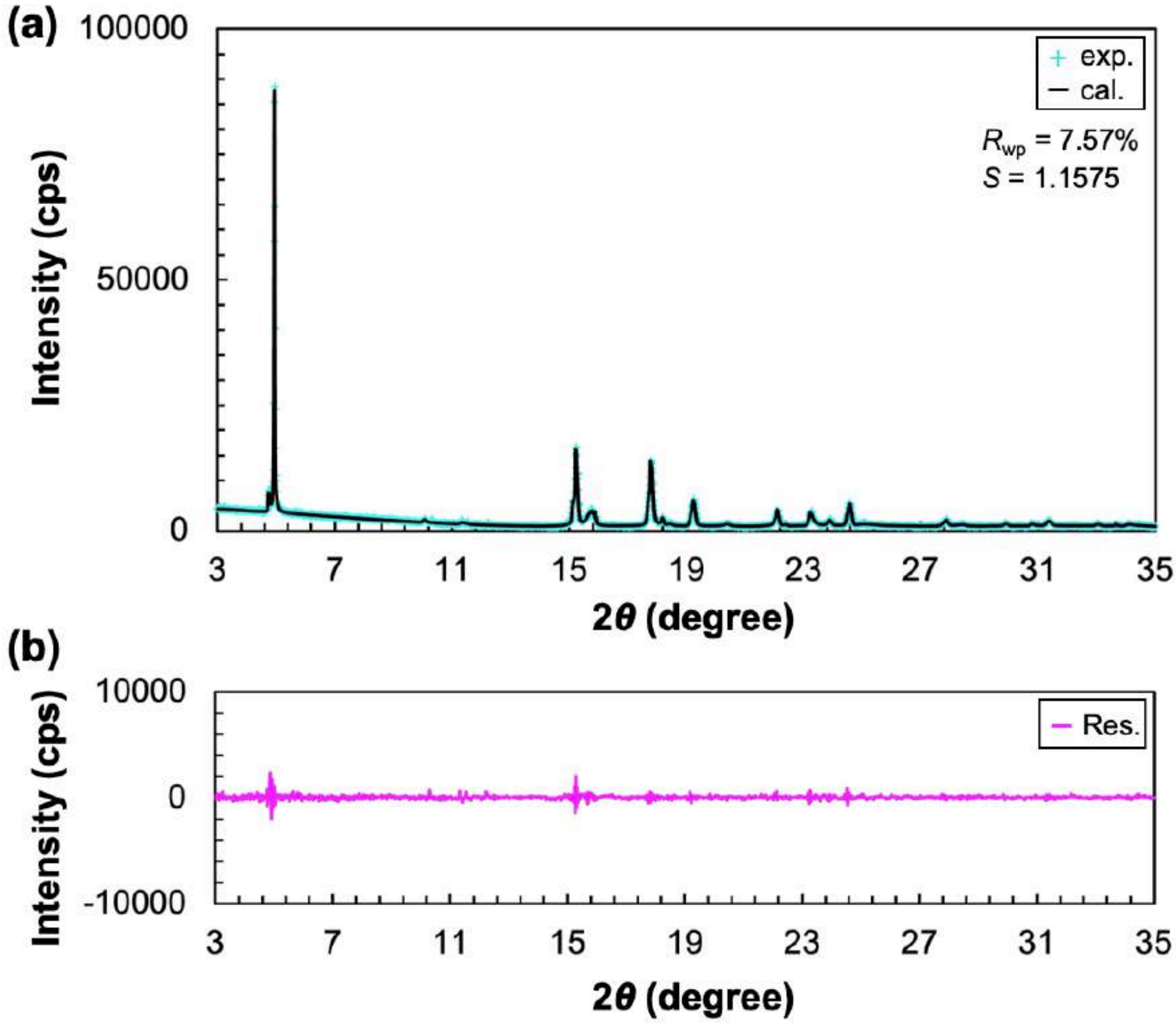

Figure S51. (a) PXRD patterns of **BBPA** measured at 303 K for crystallite-size analysis of the single-crystal sample. Experimental data are shown as cyan cross markers, and the split pseudo-Voigt fitting profile is shown as a black line. The weighted-profile reliability factor (R_{wp}) and goodness-of-fit parameter (S) are indicated as measures of the fitting quality. (b) Residual profile.

Table S8. PXRD Peak Parameters and Crystallite Sizes of the **BBPA** Single-Crystal Sample at 303 K

2θ (º)	FWHM (º)	Crystallite size (Å)
4.743(3)	0.050(10)	1657(322)
4.939(2)	0.0347(17)	2390(115)
10.07(3)	0.13(4)	646(177)
11.353(15)	0.22(5)	373(85)
15.116(2)	0.042(5)	1983(236)
15.2059(6)	0.021(2)	4026(466)
15.251(3)	0.107(6)	779(43)
15.763(5)	0.280(11)	300(12)
15.9044(10)	0.012(4)	6918(2001)
17.700(4)	0.104(16)	808(121)
17.7830(14)	0.076(3)	1104(41)
18.189(5)	0.058(10)	1455(247)
18.42(2)	0.21(4)	400(81)
19.238(4)	0.148(3)	568(13)
20.398(19)	0.17(5)	484(138)
22.104(6)	0.114(8)	739(52)
22.382(10)	0.09(3)	965(371)
23.225(3)	0.125(11)	678(58)
23.857(4)	0.128(12)	664(64)
24.570(7)	0.140(5)	606(20)
25.05(3)	0.77(8)	110(11)
27.882(4)	0.173(9)	494(27)
28.458(14)	0.42(7)	206(35)
29.883(7)	0.16(2)	538(74)
30.755(10)	0.076(15)	1140(230)
31.342(19)	0.183(14)	472(35)

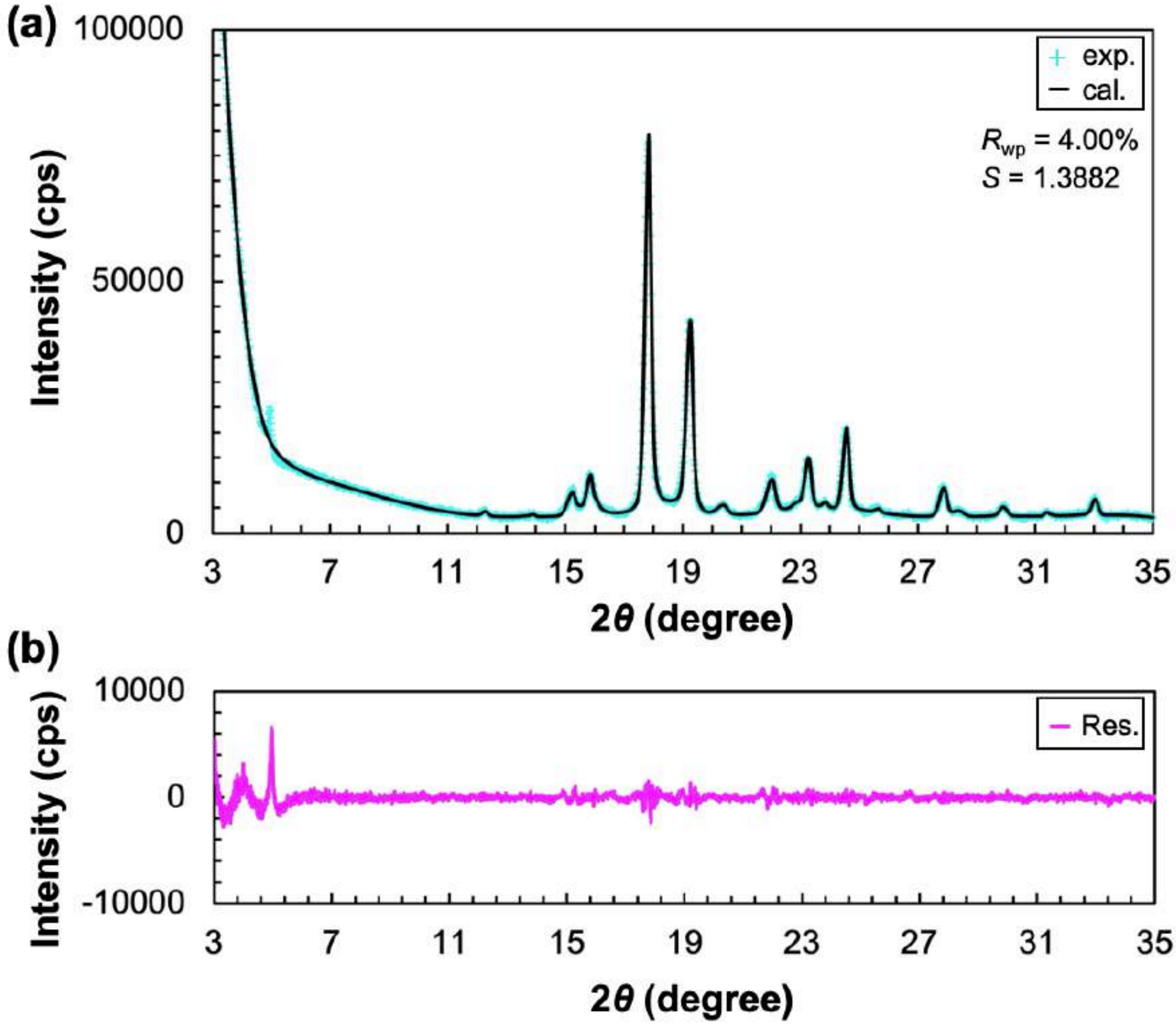

Figure S52. (a) PXRD patterns of **BBPA** measured at 303 K for crystallite-size analysis of the ground sample. Experimental data are shown as cyan cross markers, and the split pseudo-Voigt fitting profile is shown as a black line. The weighted-profile reliability factor (R_{wp}) and goodness-of-fit parameter (S) are indicated as measures of the fitting quality. An increase in the background intensity around 2θ = 3–4º is likely due to scattering from the Pt sample holder. (b) Residual profile.

Table S9. PXRD Peak Parameters and Crystallite Sizes of the **BBPA** Ground Sample at 303 K

2θ (º)	FWHM (º)	Crystallite size (Å)
12.26(3)	0.23(3)	355(45)
13.89(5)	0.25(8)	339(104)
15.250(6)	0.362(12)	231(8)
15.858(4)	0.257(8)	326(10)
17.8365(13)	0.2364(13)	355.3(19)
18.71(4)	1.27(14)	66(7)
19.2441(15)	0.258(2)	326(3)
20.317(7)	0.45(2)	188(8)
22.025(5)	0.339(6)	250(4)
22.869(16)	0.30(3)	285(37)
23.272(3)	0.257(5)	329(7)
23.847(10)	0.19(3)	442(61)
24.566(3)	0.241(3)	352(4)
25.682(14)	0.21(3)	414(63)
27.858(9)	0.284(7)	301(8)
28.35(3)	0.48(8)	180(31)
29.88(2)	0.289(19)	297(19)
31.342(12)	0.20(4)	421(89)
33.001(17)	0.231(14)	374(23)

5.3 Variable-temperature X-ray diffraction reveals structural evolution

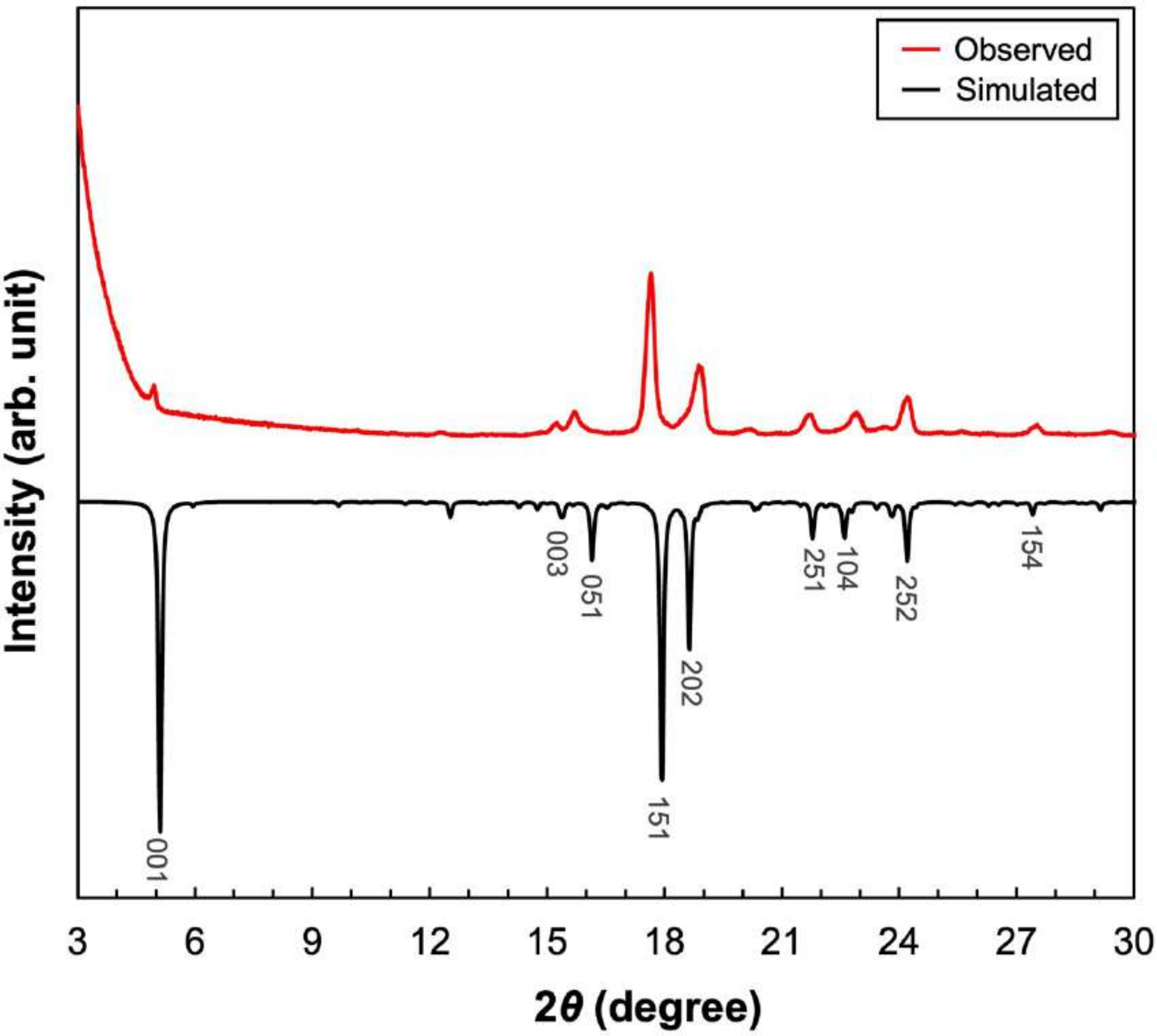

Figure S53. Simulated PXRD patterns (black line) calculated from the crystal structure of **BBPA** at 300 K (CCDC 2465110), compared with the experimental PXRD patterns (red line) measured for the ground sample at 303 K. Indexed reflections are indicated.

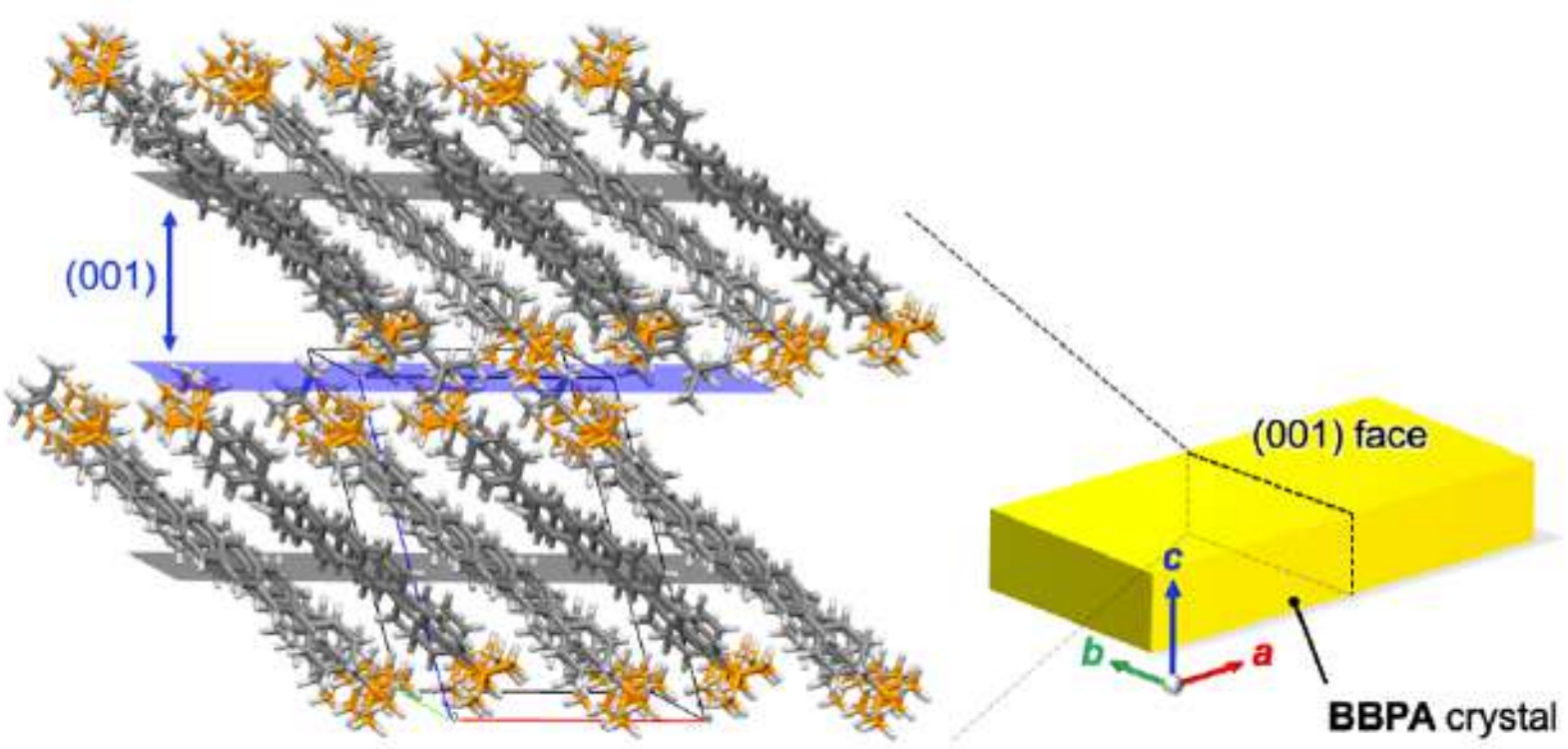

Figure S54. Crystal packing of **BBPA** at 303 K and the (001) face orientation. C of the anthracene and phenyl moieties, gray; C of the *tert*-butyl moieties, orange; H, white.

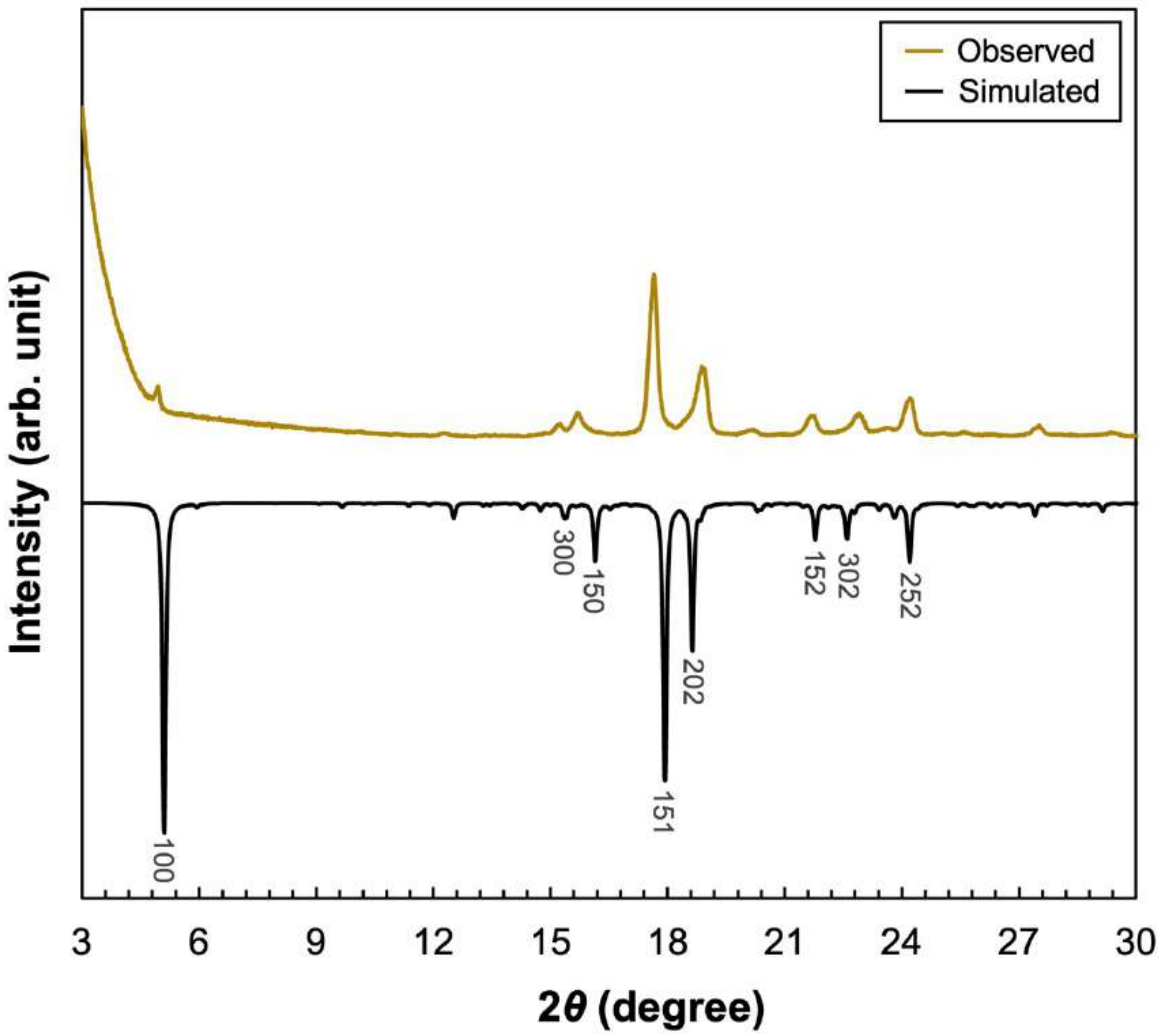

Figure S55. Simulated PXRD patterns (black line) calculated from the crystal structure of **BBPA** at 423 K (CCDC 2532311), compared with the experimental PXRD patterns (yellow line) measured for the ground sample at 473 K. Indexed reflections are indicated.

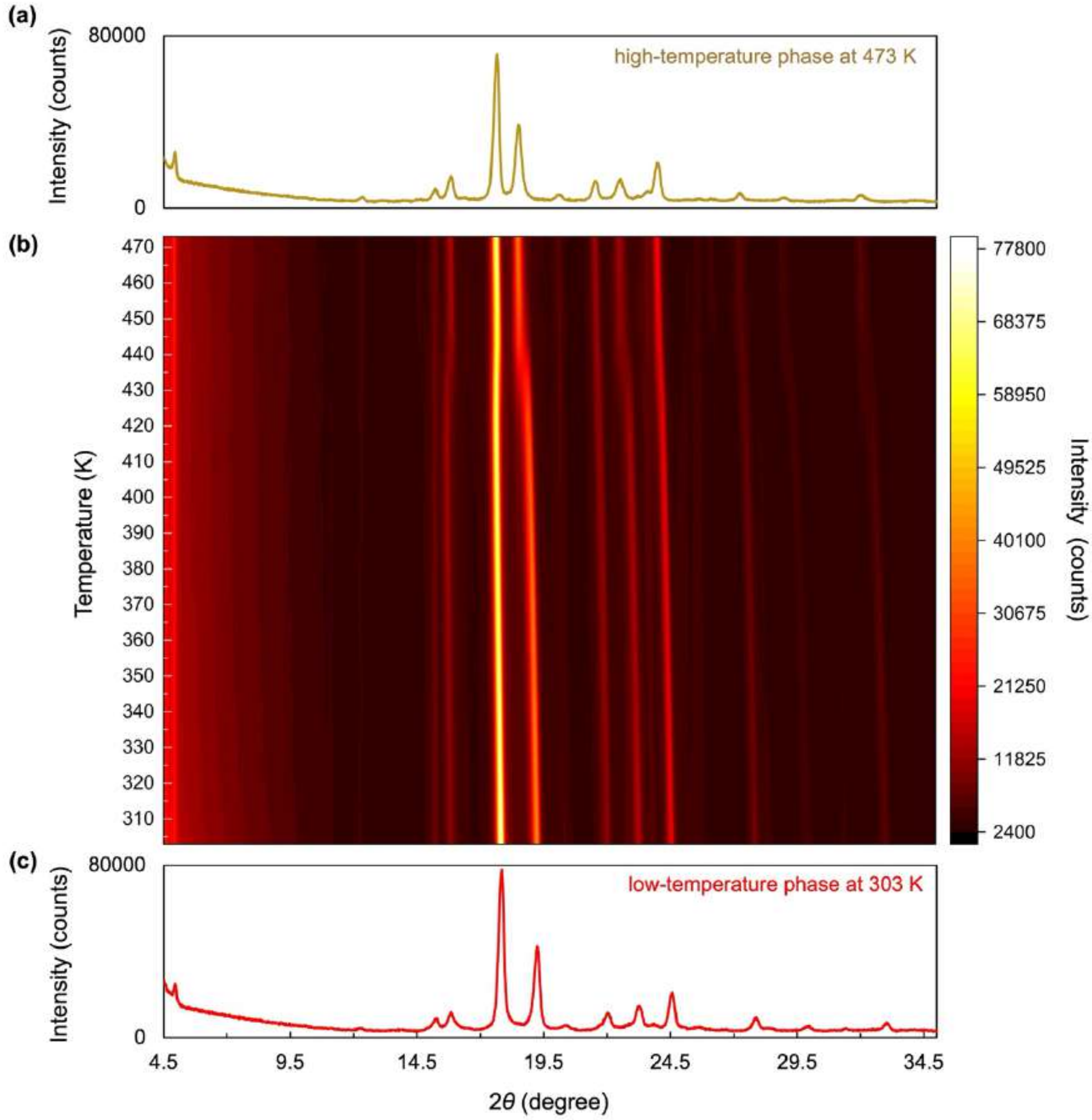

Figure S56. VT-PXRD patterns of **BBPA** collected for the ground sample during heating from 303 K to 473 K. (a) PXRD patterns in the high-temperature phase at 473 K. (b) Colormap representation of the VT-PXRD patterns. (c) PXRD patterns in the low-temperature phase at 303 K.

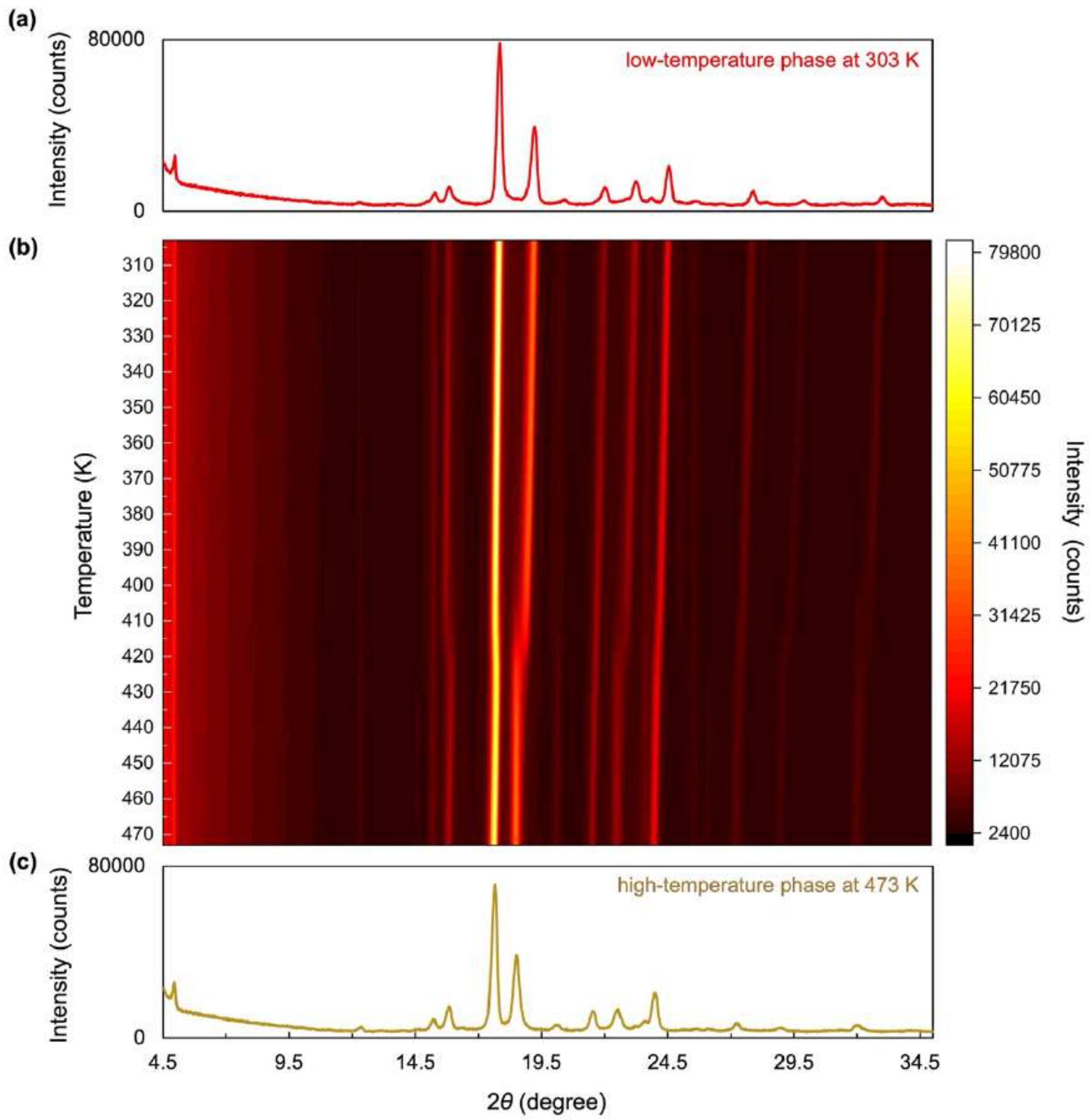

Figure S57. VT-PXRD patterns of **BBPA** collected for the ground sample during cooling from 473 K to 303 K. (a) PXRD patterns in the low-temperature phase at 303 K. (b) Colormap representation of the VT-PXRD patterns. (c) PXRD patterns in the high-temperature phase at 473 K.

5.4 Effect of mechanical grinding on the DSC behavior

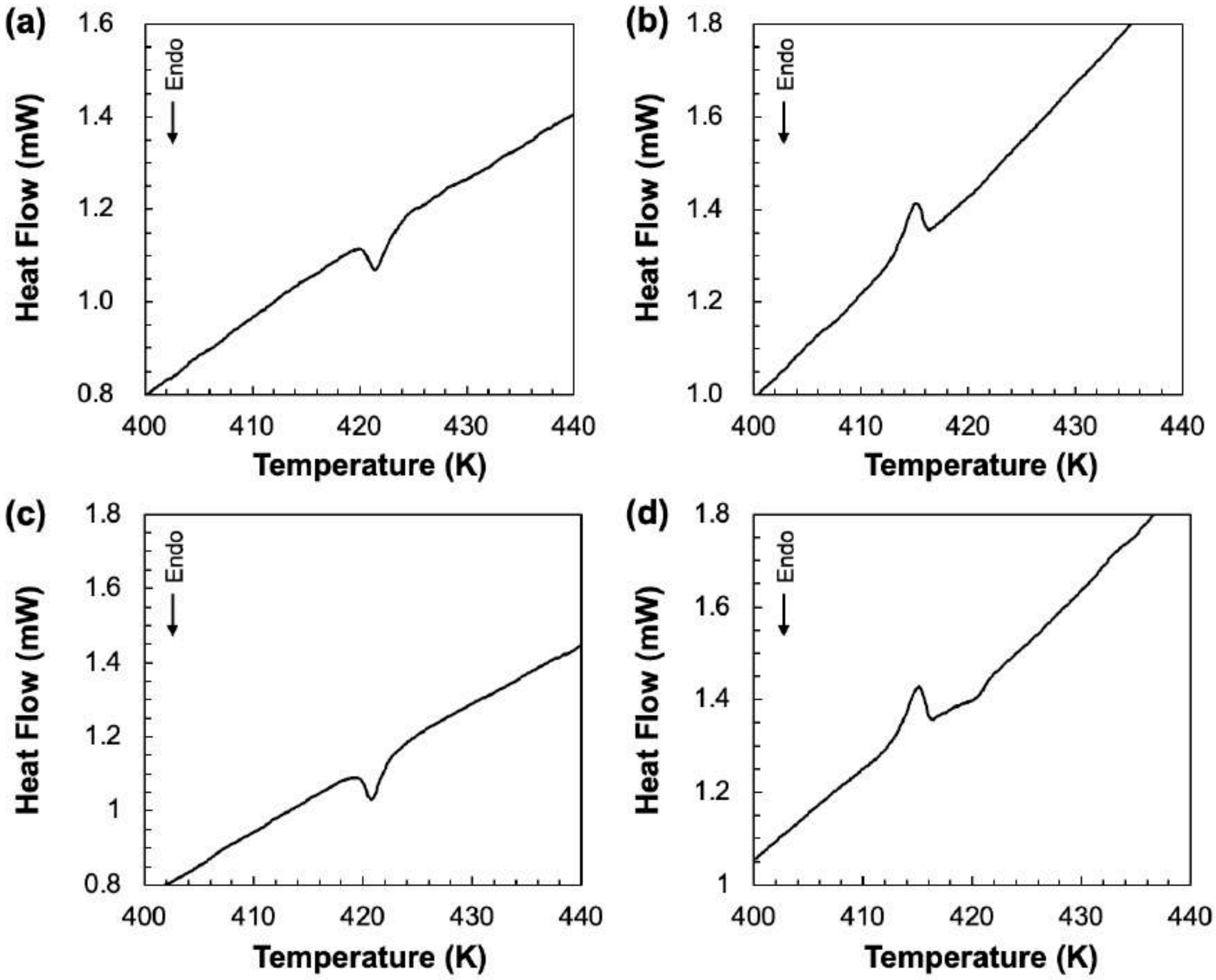

Figure S58. DSC curves of the **BBPA** single-crystal sample (3.24 mg): (a) first heating, (b) first cooling, (c) second heating, and (d) second cooling scans.

Table S10. Entropy Changes and Effective Microstate Ratios

Cycle	Process	ΔH (J/mol)	T_{onset} (K)	ΔS (J/mol K)	Effective microstate ratio
1st	Heating	101	420	0.24	W_{HT}/W_{LT} = 1.029
1st	Cooling	−52	417	−0.12	W_{LT}/W_{HT} = 0.986
2nd	Heating	107	419	0.26	W_{HT}/W_{LT} = 1.032
2nd	Cooling	−55	417	−0.13	W_{LT}/W_{HT} = 0.985

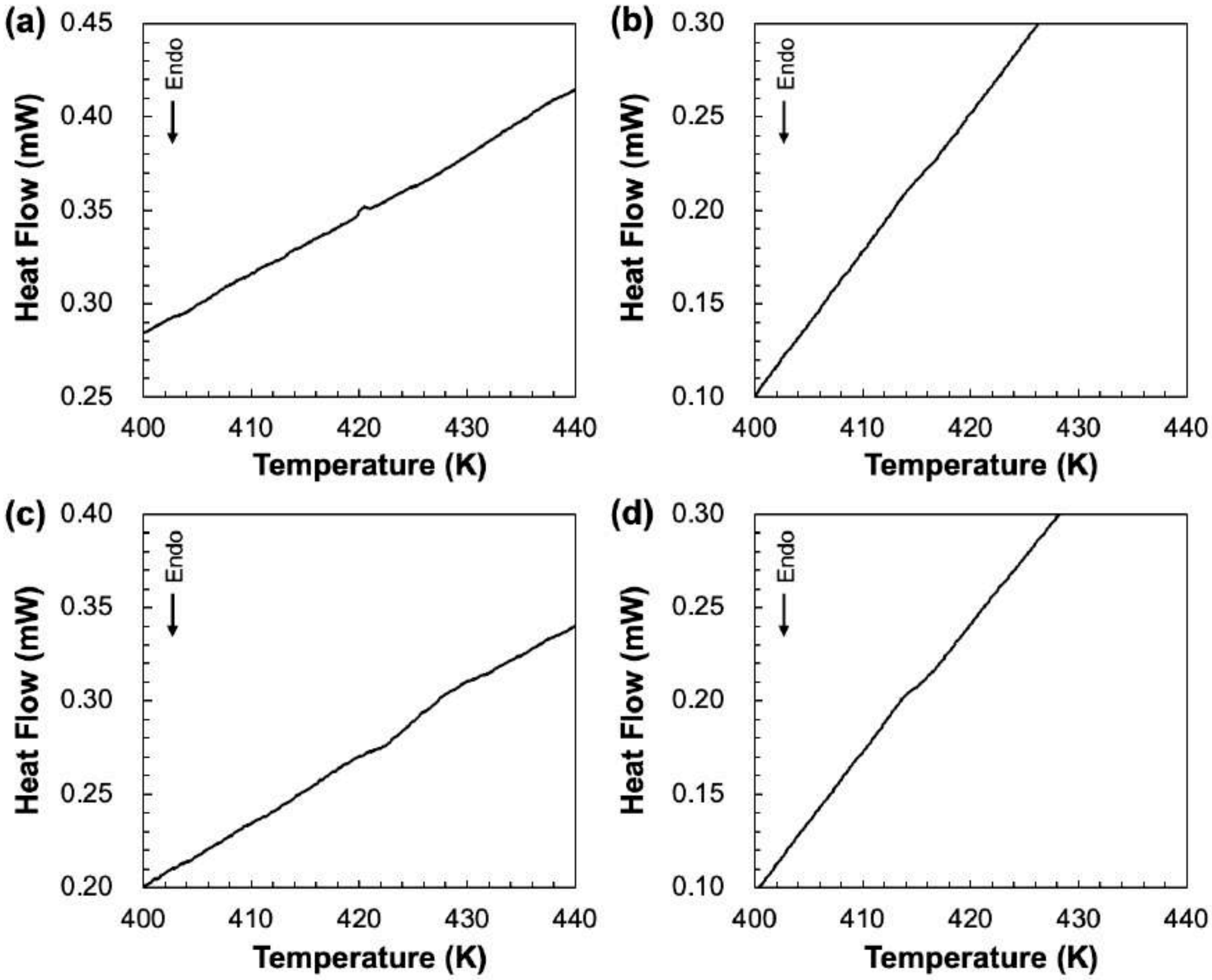

Figure S59. DSC curves of the **BBPA** ground sample (3.33 mg): (a) first heating, (b) first cooling, (c) second heating, and (d) second cooling scans.

6. Temperature-dependent SHG response

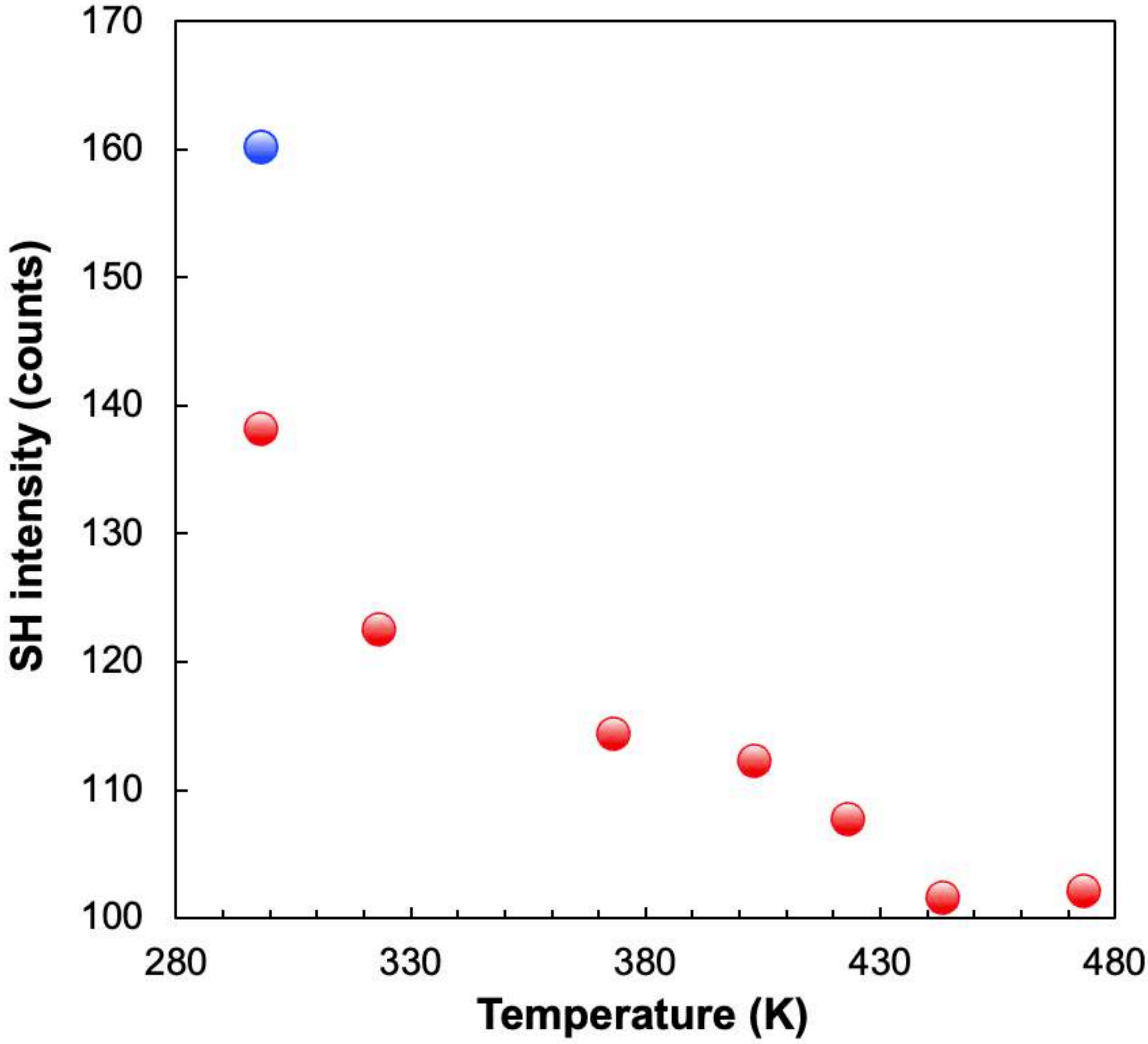

Figure S60. Temperature-dependent second harmonic (SH) intensity of a **BBPA** single crystal measured during heating from 298 K to 473 K (red circles). The blue circle corresponds to the SH intensity measured after cooling back to 298 K. The decrease in SH intensity upon heating and its recovery upon cooling are consistent with the reversible symmetry change associated with the crystallographically observed phase transition between the noncentrosymmetric $P2_1$ phase and the centrosymmetric $P2_1/c$ phase.

7. Microscopic observation of thermal stability limits

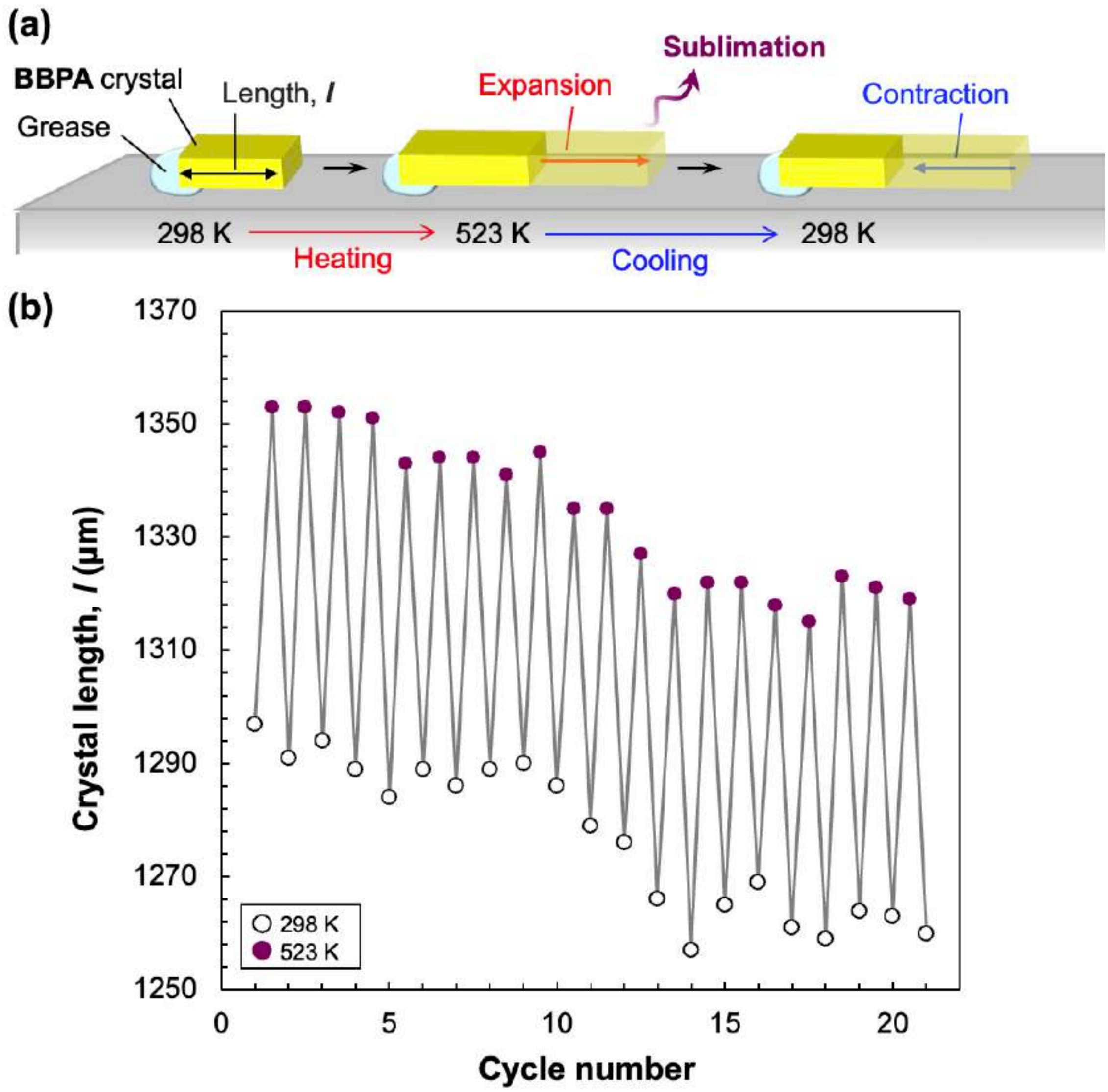

Figure S61. Applicable temperature window for anisotropic thermal expansion.[31] (a) Schematic illustration of thermal expansion and contraction in **BBPA** single-crystal sample, with elongation accompanied by sublimation upon heating (above 523 K) and shrinkage upon cooling. (b) Irreversible crystal-length changes upon thermal cycling up to 523 K.

8. Generality of the high-Z' crystal-engineering strategy across anthracene scaffolds

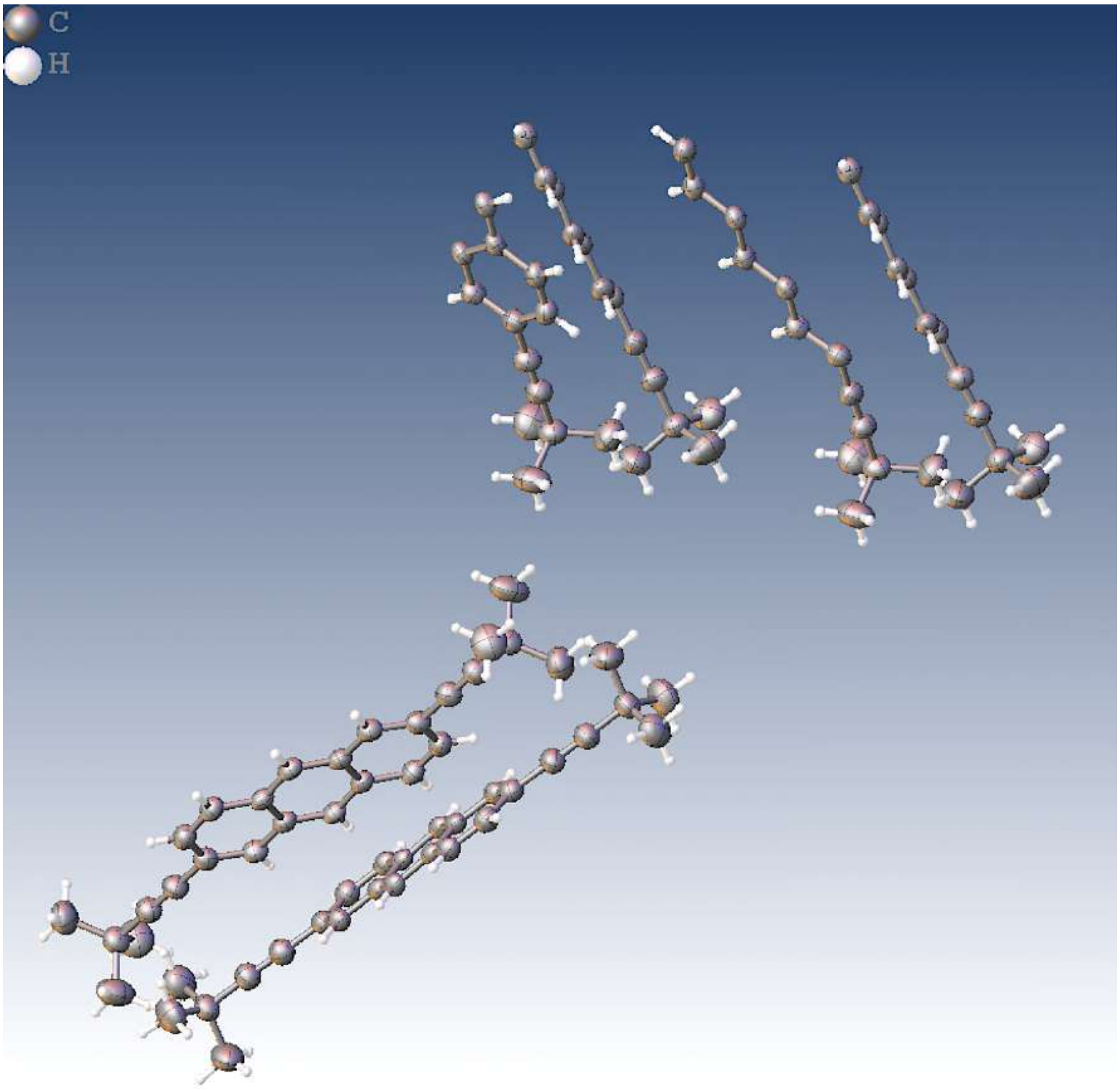

Figure S62. Crystal structure of **BBEA** determined at 303 K (CCDC 2578744). ORTEP drawing shown with 50% probability anisotropic displacement parameters. $Z' = 4$.

Table S11. Crystal Data and Structure Refinement Parameters for the **BBEA** at 103 K

Identification code	exp_799	
CCDC deposit number	2558815	
Empirical formula	$C_{22}H_{22}$	
Formula weight	338.47	
Wavelength	0.71073 Å	
Crystal system	triclinic	
Space group	$P\bar{1}$	
Unit cell dimensions	a = 11.8442(2) Å	α = 91.1550(10)°
	b = 11.9402(2) Å	β = 95.0950(10)°
	c = 33.2880(5) Å	γ = 119.725(2)°
Volume	4060.82(13) $Å^3$	
Z	8	
Density (calculated)	1.107 g/cm^3	
Absorption coefficient	0.062 mm^{-1}	
F(000)	1456.0	
Crystal size	0.396 × 0.273 × 0.105 mm^3	
2θ range for data collection	3.696 to 60.316°	
Index ranges	$-14 \leqq h \leqq 16$, $-16 \leqq k \leqq 16$, $-45 \leqq l \leqq 44$	
Reflections collected	57121	
Independent reflections	19849 [R(int) = 0.0257]	
Data / restraints / parameters	19849 / 0 / 961	
Goodness-of-fit on F^2	1.050	
Final R indices [I>2σ(I)]	R_1 = 0.0554, wR_2 = 0.1619	
R indices (all data)	R_1 = 0.0872, wR_2 = 0.1839	
Largest diff. peak and hole	0.68/-0.23 e.$Å^{-3}$	

A- and B-level alerts in the Checkcif file:

(There is no A-level alerts)

B-Level alerts
"Large Reported Max. (Positive) Residual Density 0.68 eA-3"
This is due to residual electron density located in the vicinity of carbon atoms and does not indicate any chemically meaningful missing atoms or unresolved disorder.

Table S12. Crystal Data and Structure Refinement Parameters for the **BBEA** at 303 K

Identification code	exp_804	
CCDC deposit number	2578744	
Empirical formula	$C_{22}H_{22}$	
Formula weight	338.47	
Wavelength	0.71073 Å	
Crystal system	triclinic	
Space group	$P\bar{1}$	
Unit cell dimensions	a = 11.9558(3) Å	α = 83.349(2)º
	b = 12.0778(3) Å	β = 84.934(2)º
	c = 33.7088(5) Å	γ = 60.376(2)º
Volume	4200.10(18) $Å^3$	
Z	8	
Density (calculated)	1.071 g/cm^3	
Absorption coefficient	0.060 mm^{-1}	
F(000)	1456.0	
Crystal size	0.388 × 0.253 × 0.111 mm^3	
2θ range for data collection	3.652 to 60.408º	
Index ranges	$-16 \leqq h \leqq 14$, $-17 \leqq k \leqq 17$, $-46 \leqq l \leqq 45$	
Reflections collected	56901	
Independent reflections	20459 [R(int) = 0.0253]	
Data / restraints / parameters	20459 / 0 / 961	
Goodness-of-fit on F^2	1.035	
Final R indices [I>2σ(I)]	R_1 = 0.0627, wR_2 = 0.1770	
R indices (all data)	R_1 = 0.01183, wR_2 = 0.2093	
Largest diff. peak and hole	0.41/-0.17 e.$Å^{-3}$	

A- and B-level alerts in the Checkcif file:

(There are no A- and B-level alerts)

9. Reported thermal phase transitions and *Z′* values in single-component crystals

Table S13. Selected Anthracene Derivatives Highlighting Their *Z′* Values

**Compounds**	***Z′***	**Measurement temperatures**	**CCDC Deposition Numbers**	**References**
anthracene	0.5	295 K	1103062	10
2,6-di-*tert*-butylanthracene	0.5	173 K	887757	11
2,6-diphenylanthracene	0.5	113 K	1044209	8
2,6-bis(naphthalen-1-yl)anthracene	0.5	293 K	2017537	12
2,6-bis(naphthalen-2-yl)anthracene	1	150 K	1901585	12
2,6-bis(4-methylphenyl)anthracene	0.5	296 K	1982542	13
2,6-bis(4-propylphenyl)anthracene	1	150 K	1982543	13
2,6-bis(4-ethylphenyl)anthracene	0.5	293 K	1442079	14
2,6-bis(4-methoxyphenyl)anthracene	0.5	293 K	1442078	14
2,6-bis(4-(methylsulfanyl)phenyl)anthracene	2	293 K	1442077	14
2,6-bis(4-*tert*-butylphenyl)anthracene	5	103 K	2465106	This work
2,6-bis(4-(trifluoromethyl)phenyl)anthracene	0.5	296 K	252391	15
2,6-bis(thien-2-yl)anthracene	0.5	293 K	265820	16
2,6-bis(5-*n*-hexylthien-2-yl)anthracene	0.5	293 K	265821	16
2,6-bis(6-*t*-butylnaphthalen-2-yl)anthracene	0.5	294 K	2160624	17
4-(6-(pyridin-4-yl)-2-anthryl)pyridine	0.5	173 K	1439496	18
7,7'-(anthracene-2,6-diyl)diquinoline	0.5	170 K	2383915	19

Note: This table supports the anthracene-based high-*Z′* crystal strategy, which decouples structural order from molecular flexibility by spatially confining torsional degrees of freedom to the molecular periphery. It further suggests a general route to high-*Z′* differentiation in molecular crystals.

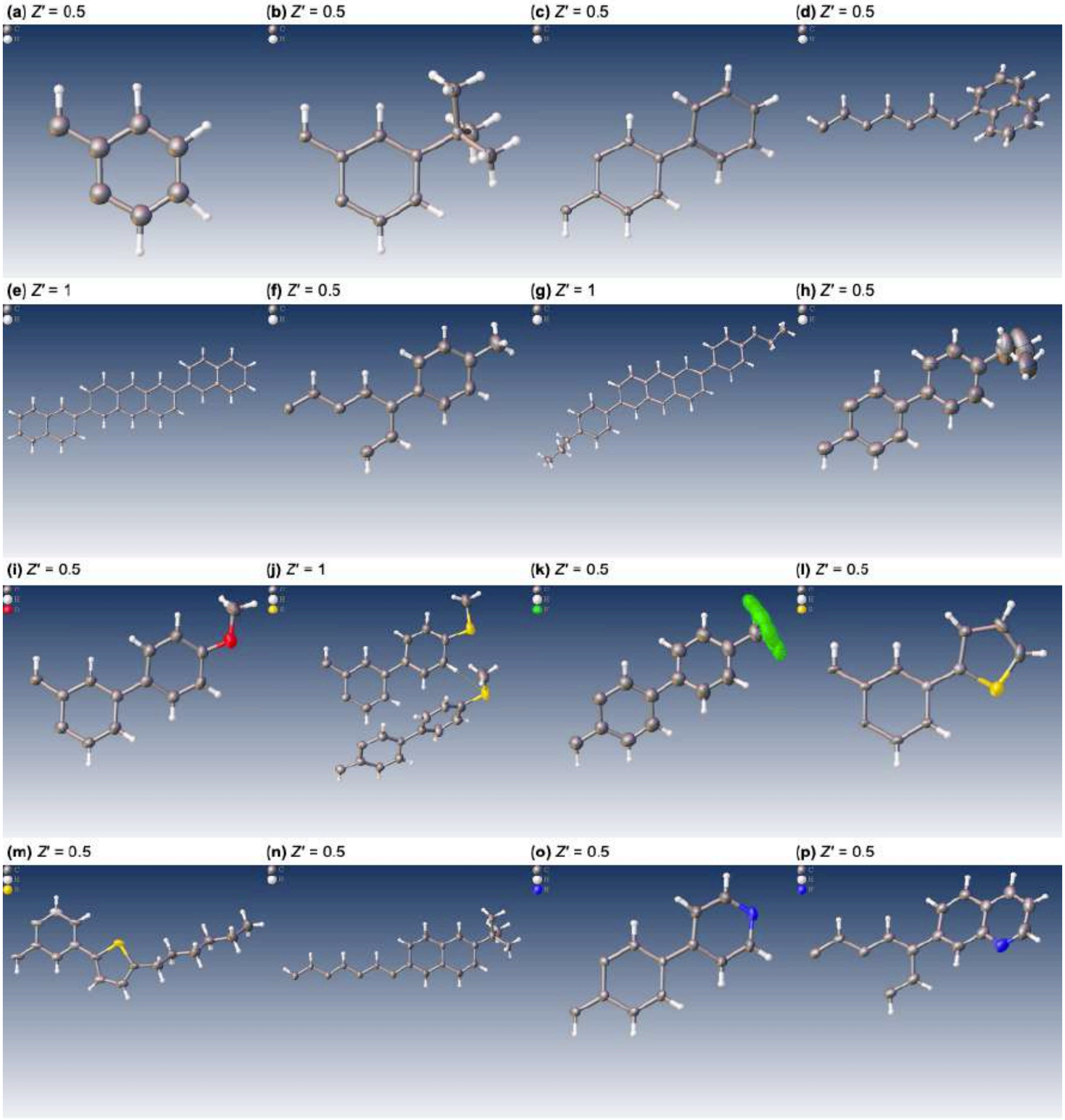

Figure S63. Selected anthracene derivatives and their Z' values. CCDC deposition numbers of (a) 1103062, (b) 887757, (c) 1044209, (d) 2017537, (e) 1901585, (f) 1982542, (g) 1982543, (h) 1442079, (i) 1442078, (j) 1442077, (k) 252391, (l) 265820, (m) 265821, (n) 2160624, (o) 1439496, and (p) 2383915.

Table S14. Selected Thermal Solid-State Phase Transitions Highlighting Their Z' Values

Compounds	Z'	Measurement temperatures	Reversibility	CCDC Deposition Numbers	References
(*S*,*S*)-octa-3,5-diyn-2,7-diol	1	225 K	reversible	723906	20
		330 K		723913	
(2(phenyldiazenyl) phenyl)(1,1,1,5,5,5-hexafluoroacetyl acetonato)palladium(ii)	2	90 K	reversible	1013656	21
	2	149 K		1013645	
	1	358 K		1013657	
	2	100 K	reversible	1013654	
	1	298 K		1013655	
2,7-bis[4(decyloxy)phenyl]benzo[lmn][3,8]phenanthroline-1,3,6,8(2H,7H)-tetrone	0.5	298 K	reversible	1992521	22
	1	338 K		1992522	
5,6-dimethoxy-4,7-bis[5-(4-nonylphenyl)thiophen-2-yl]-2,1,3-benzothiadiazole	2	296 K	reversible	2313851	23
	1	353 K		2313852	
methyl *N*-(*t*-butoxycarbonyl)methionylglycinate	8	100 K	reversible	2113339	24
	4	166 K		2113337	
(*E*)-*N'*-(diphenyl methylene)-4-hydroxybenzohydrazide	2	173 K	reversible	2348689	25
	6	293 K		2348688	
	1	383 K		2348687	
2,7-di-*t*-butyl[1] benzothieno[3,2-b][1]benzothiophene	0.5	298 K	reversible	1570908	26
		370 K		1570909	
(pentacene-6,13-diylbis(ethyne-2,1-diyl))bis(tri isopropylsilane)	0.5	300 K	reversible	1570910	26
		400 K		1570911	
2,7-bis(propan-2-yl)[1]benzothieno[3,2-b][1]benzo thiophene	0.5	90 K	irreversible	1008120	27
		100 K		1959510	28

([1]benzothieno[3,2-b][1]benzothiene-2,7-diyl)bis(trimethylsilane)	1	123 K	irreversible	1008576	27
	0.5	100 K		1959511	28
2,6-bis(4-*tert*-butylphenyl)anthracene	5	100 K	reversible	2532253	This work
	2.5	423 K		2532311	
2,8-di-*t*-butyl thieno[3',2':5,6][1]benzothieno[3,2-b]thieno[3,2-f][1]benzothiophene	1	100 K	reversible	2345569	29
		255 K		2345570	
3,7-bis([1,1'-biphenyl]-4-yl)-5H-dibenzo[b,d]thiophene-5,5-dione3,7-bis([1,1'-biphenyl]-4-yl)-5H-5-dibenzo[b,d]thiophene-5,5-dione	1	193 K	reversible	2286600	30
	0.5	470 K		2286599	

Note: This table summarizes reported thermal solid-state phase transitions in single-component molecular crystals. To date, high-*Z′* crystals have predominantly been observed in hydrogen-bonded organic systems. Although many thermal phase transitions have been induced by the motional freedom of side chains appended to π-conjugated cores, the corresponding *Z′* values are typically ≤ 2, and cooperative, correlated molecular motions have rarely been directly crystallographically resolved.

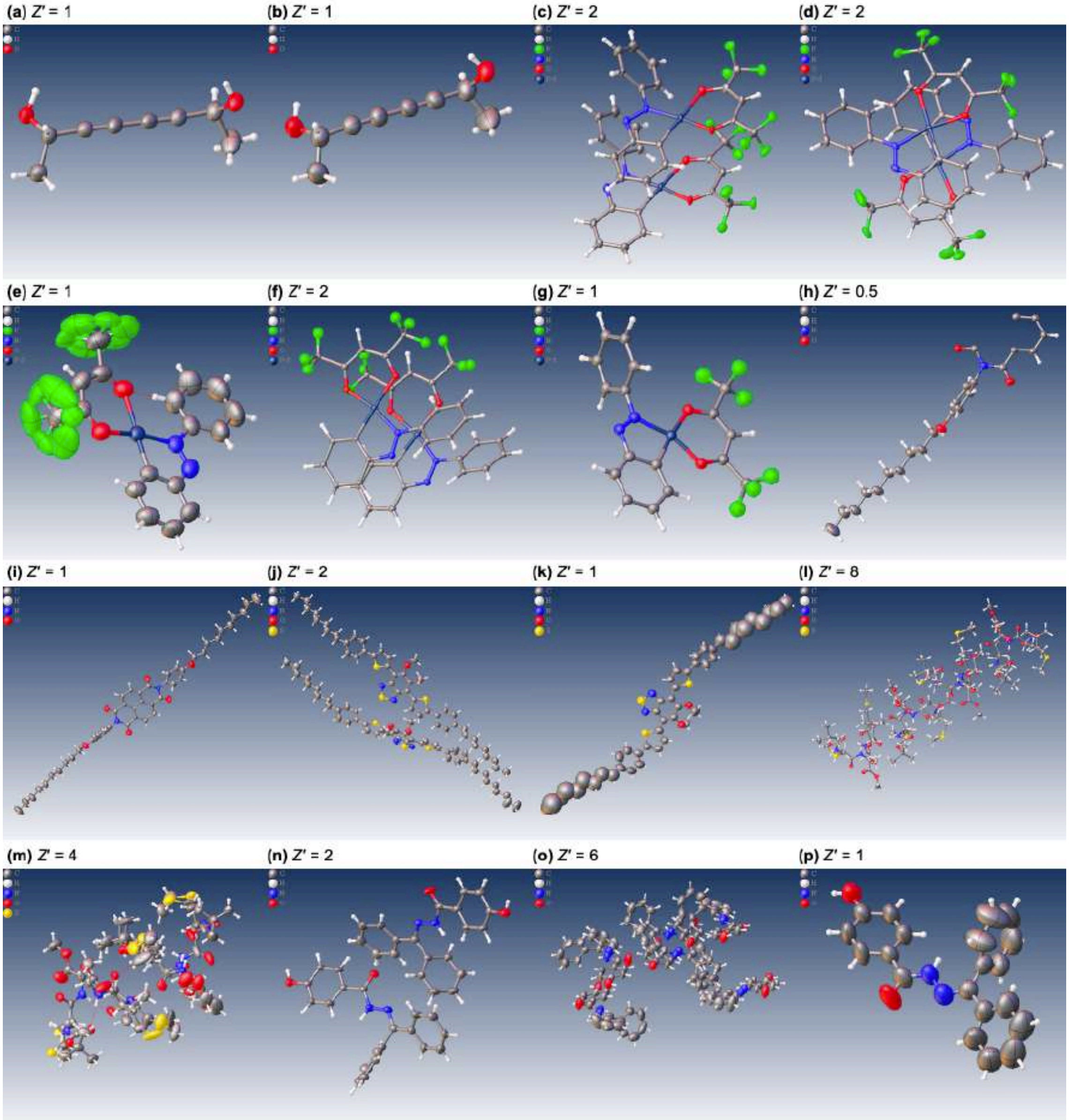

Figure S64. Selected compounds and their Z' values. CCDC deposition numbers of (a) 723906, (b) 723913, (c) 1013656, (d) 1013645, (e) 1013657, (f) 1013654, (g) 1013655, (h) 1992521, (i) 1992522, (j) 2313851, (k) 2313852, (l) 2113339, (m) 2113337, (n) 2348689, (o) 2348688, and (p) 2348687.

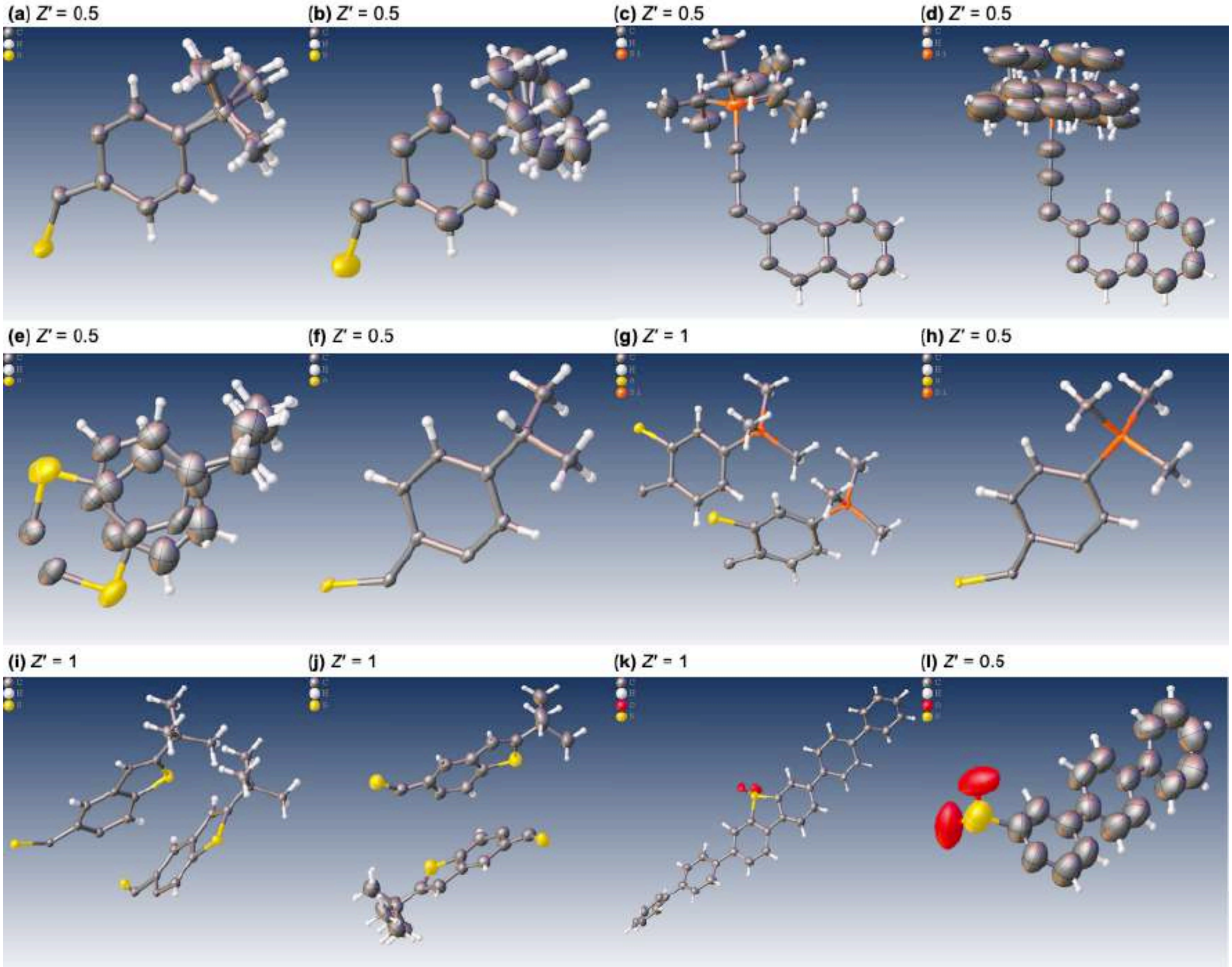

Figure S65. Selected compounds and their *Z*′ values. CCDC deposition numbers of (a) 1570908, (b) 1570909, (c) 1570910, (d) 1570911, (e) 1008120, (f) 1959510, (g) 1008576, (h) 1959511, (i) 2345569, (j) 2345570, (k) 2286600, and (l) 2286599.

References

[1] M. M. H. Smets, E. Kalkman, A. Krieger, et al, *IUCrJ* **2020**, *7*, 331–341, https://doi.org/10.1107/S2052252520001335.

[2] O. V. Dolomanov, L. J. Bourhis, R. J. Gildea, J. A. K. Howard, H. Puschmann, *J. Appl. Cryst.* **2009**, *42*, 339–341, https://doi.org/10.1107/S0021889808042726.

[3] G. M. Sheldrick, *Acta Cryst. A* **2015**, *71*, 3–8, https://doi.org/10.1107/S2053273314026370.

[4] G. M. Sheldrick, *Acta Cryst. C* **2015**, *71*, 3–8, https://doi.org/10.1107/S2053229614024218.

[5] K. Sugimoto, H. Ohsumi, S. Aoyagi, et al., *AIP Conf. Proc.* **2010**, *1234*, 887–890, https://doi.org/10.1063/1.3463359.

[6] A. D. Becke, *J. Chem. Phys.* **1993**, *98*, 5648–5652, https://doi.org/10.1063/1.464913.

[7] M. J. Frisch, G. W. Trucks, H. B. Schlegel, et al., Gaussian 16, Revision B.01, Gaussian, Inc., Wallingford, CT, 2016.

[8] J. Liu, H. Zhang, H. Dong, et al., *Nat. Commun.* **2015**, *6*, 10032, https://doi.org/10.1038/ncomms10032.

[9] J. Liu, H. Dong, Z. Wang, et al., *Chem. Commun.* **2015**, *51*, 11777–11779, https://doi.org/10.1039/C4CC10348C.

[10] R.Mason, *Acta Cryst.* 1964, **17**, 547, https://doi.org/10.1107/S0365110X64001281.

[11] C. Hoffend, F. Schodel, M. Bolte, H. -W. Lerner, M. Wagner, *Chem. Eur. J* **2012**, *18*, 15394, https://doi.org/10.1002/chem.201202280.

[12] J. Li, D. Ji, Y. Hu, et al., *J. Mater. Chem. C* **2020**, *8*, 15597, https://doi.org/10.1039/D0TC03426F.

[13] D. Zhang, S. Yokomori, R. Kameyama, et al., *ACS Appl. Mater. Interfaces* **2021**, *13*, 989, https://doi.org/10.1021/acsami.0c16144.

[14] L. Yan, Y. Zhao, H. Yu, et al., *J. Mater. Chem. C* **2016**, *4*, 3517, https://doi.org/10.1039/C6TC01088A.

[15] S. Ando, J. Nishida, E. Fujiwara, et al., *Chem. Mater.* **2005**, *17*, 1261, https://doi.org/10.1021/cm0478632.

[16] H. Meng, F. Sun, M. B. Goldfinger, et al., *J. Am. Chem. Soc.* **2005**, *127*, 2406, https://doi.org/10.1021/ja043189d.

[17] J. Li, Z. Qin, Y. Sun, et al., *Angew. Chem. Int. Ed.* **2022**, *61*, e202206825, https://doi.org/10.1002/anie.202206825.

[18] J. Liu, W. Zhu, K. Zhou, et al., *J. Mater. Chem. C* **2016**, *4*, 3621, https://doi.org/10.1039/C6TC00499G.

[19] P. Wang, C. Gao, Z. Ni, et al., *Angew. Chem. Int. Ed.* **2025**, *64*, e202419213, https://doi.org/10.1002/anie.202419213.

[20] D. Das, T. Jacobs, L. J. Barbour, *Nat. Mater.* **2010**, *9*, 36, https://doi.org/10.1038/nmat2583.

[21] M. K. Panda, T. Runčevski, S. C. Sahoo, et al., *Nat. Commun.* **2014**, *5*, 4811, https://doi.org/10.1038/ncomms5811.

[22] M. Dharmarwardana, S. Pakhira, R. P. Welch, et al., *J. Am. Chem. Soc.* **2021**, *143*, 5951, https://doi.org/10.1021/jacs.1c01549.

[23] M. A. Uddin, R. Martín, S. Gámez-Valenzuela, et al., *J. Am. Chem. Soc.* **2024**, *146*, 27690, https://doi.org/10.1021/jacs.4c09222.

[24] R. Oketani, H. Takahashi, S. Clevers, et al., *Cryst. Growth Des.* **2022**, *22*, 2230, https://doi.org/10.1021/acs.cgd.1c01330.

[25] E. Parisi, E. Santagata, E. Simone, F. Borbone, R. Centore, *J. Am. Chem. Soc.* **2024**, *146*, 19405, https://doi.org/10.1021/jacs.4c05559.

[26] H. Chung, D. Dudenko, F. Zhang, et al., *Nat. Commun.* **2018**, *9*, 278, https://doi.org/10.1038/s41467-017-02607-9.

[27] G. Schweicher, V. Lemaur, C. Niebel, et al., *Adv. Mater.* **2015**, *27*, 3066, https://doi.org/10.1002/adma.201500322.

[28] H. Chung, S. Chen, B. Patel, et al., *Cryst. Growth Des.* **2020**, *20*, 1646, https://doi.org/10.1021/acs.cgd.9b01372.

[29] Lu. Catalano, R. Sharma, D. P. Karothu, et al., J. Am. Chem. Soc. **2024**, *146*, 31911, https://doi.org/10.1021/jacs.4c11289.

[30] Z. Wang, R. Shi, I. Tahir, et al., *J. Am. Chem. Soc.* **2025**, *147*, 7749, https://doi.org/10.1021/jacs.4c17448.

[31] Y. Hino, T. Matsuo, D. Korenaga, et al., *Zenodo*, **2026**, https://doi.org/10.5281/zenodo.18844678.

[32] *Rowan Scientific.* https://www.rowansci.com (accessed 2026-05-23).

[33] R. Li, Q. Sun, X. Zhang, et al., *J. Phys. Chem. A* **2025**, *129*, 1459–1468, https://doi.org/10.1021/acs.jpca.4c05876.

[34] A. Najibi, L. Goerigk, *J. Chem. Theory Comput.* **2018**, *14*, 5725–5738, https://doi.org/10.1021/acs.jctc.8b00842.

[35] S. Lehtola, C. Steigemann, M. J. T. Oliveira, et al., *Software X* **2018**, *7*, 1–5, https://doi.org/10.1016/j.softx.2017.11.002.

NMR spectra

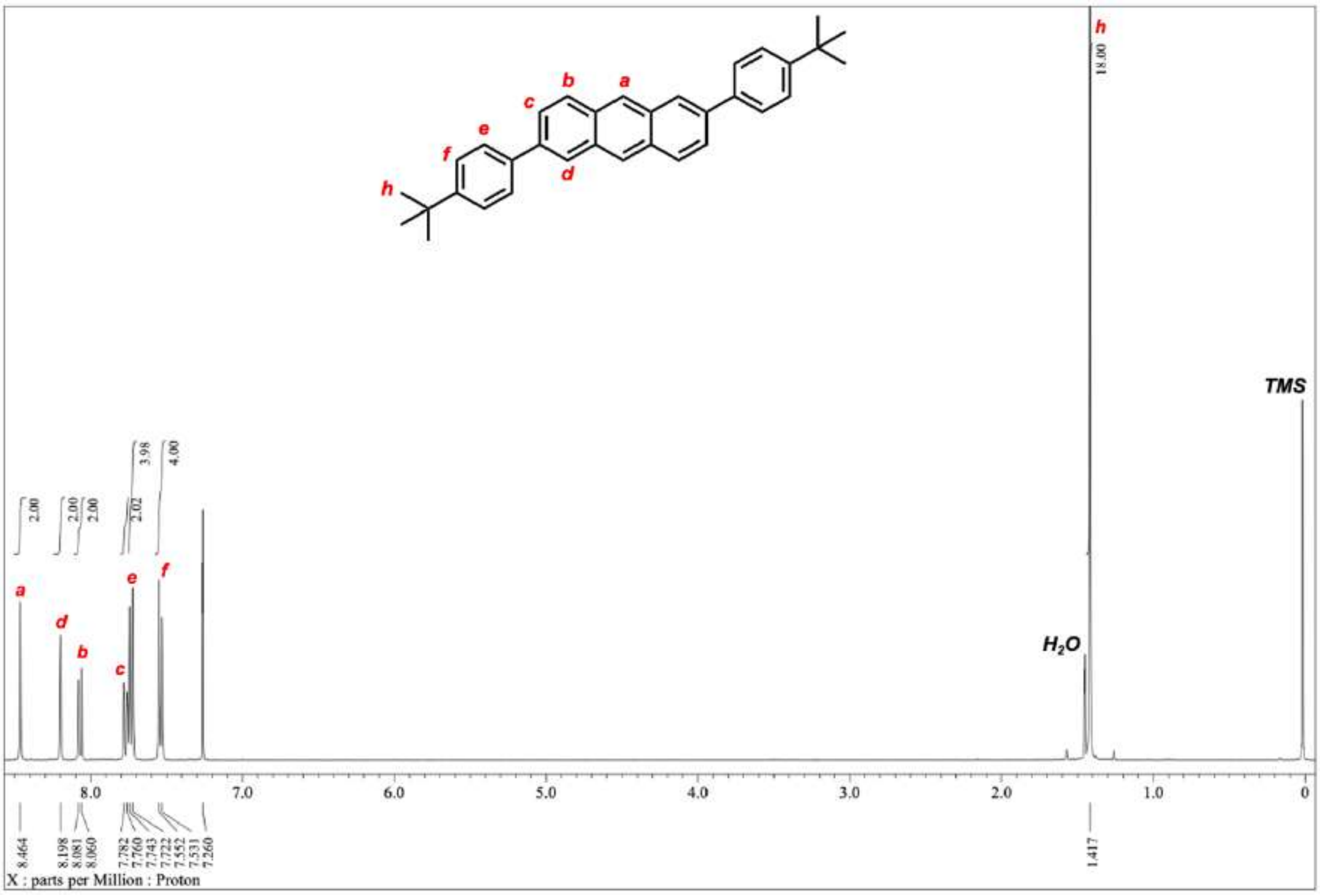

Figure S66. ^{1}H NMR spectrum of **BBPA**.

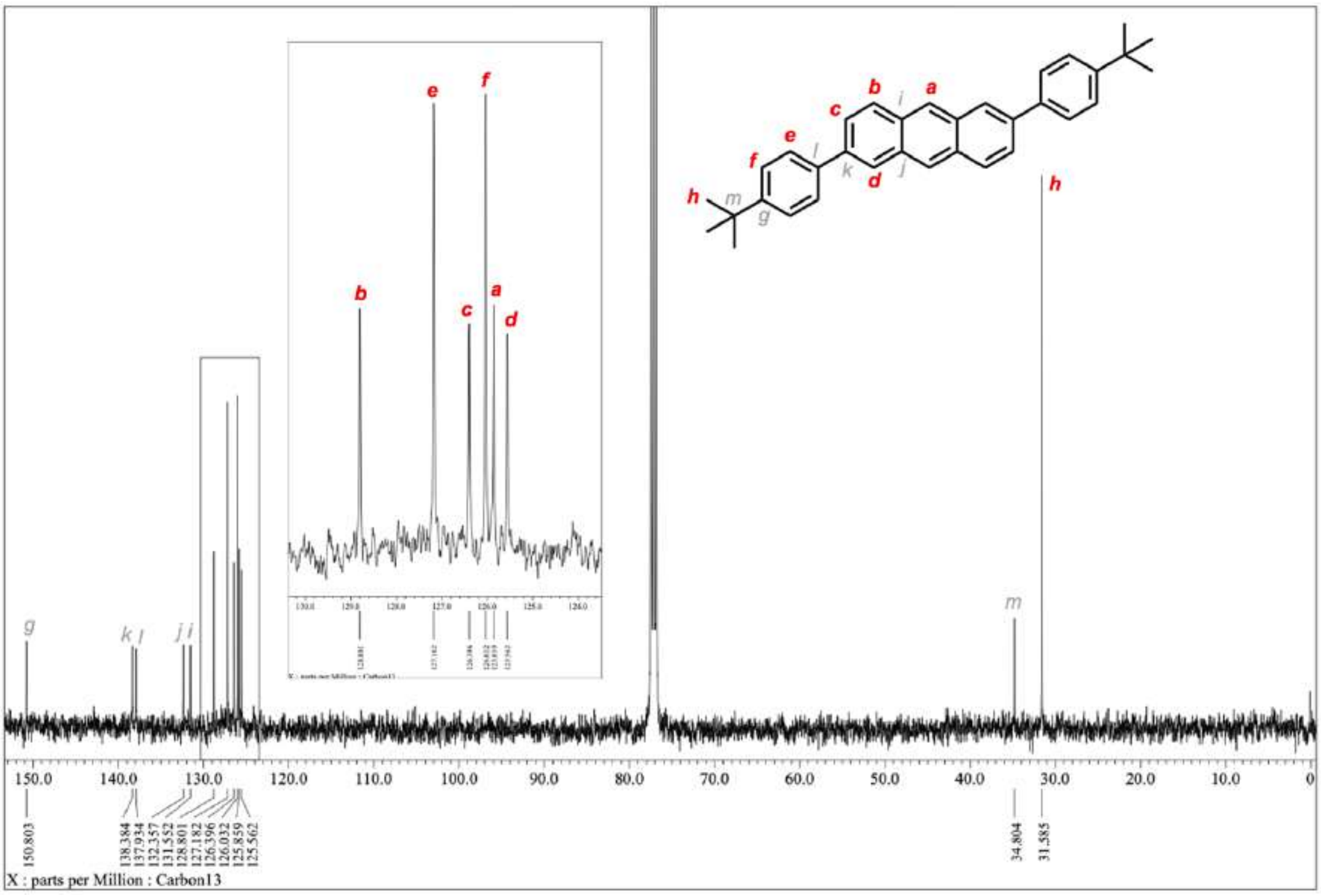

Figure S67. ^{13}C NMR spectra of **BBPA**.

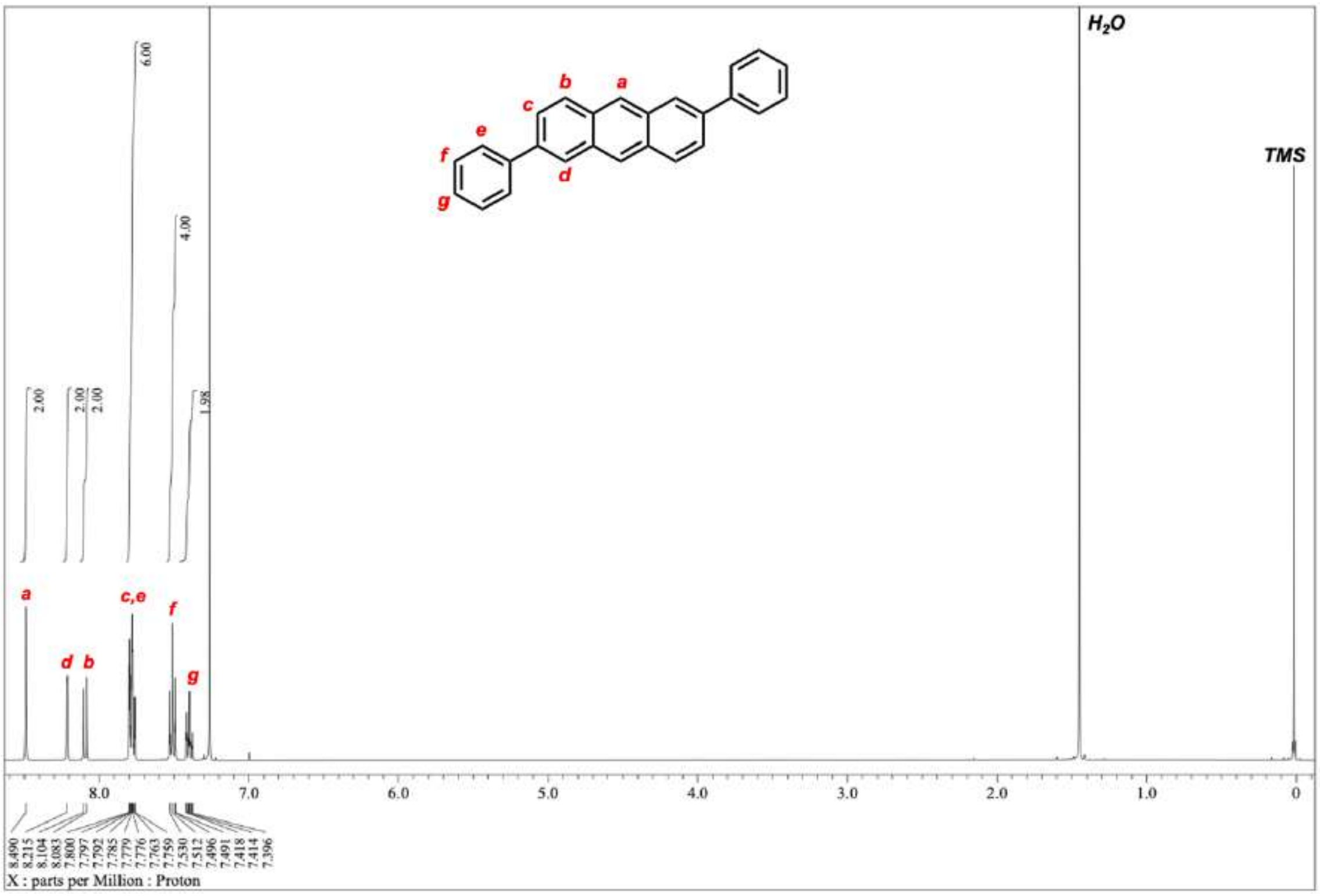

Figure S68. ^{1}H NMR spectrum of **DPA**.

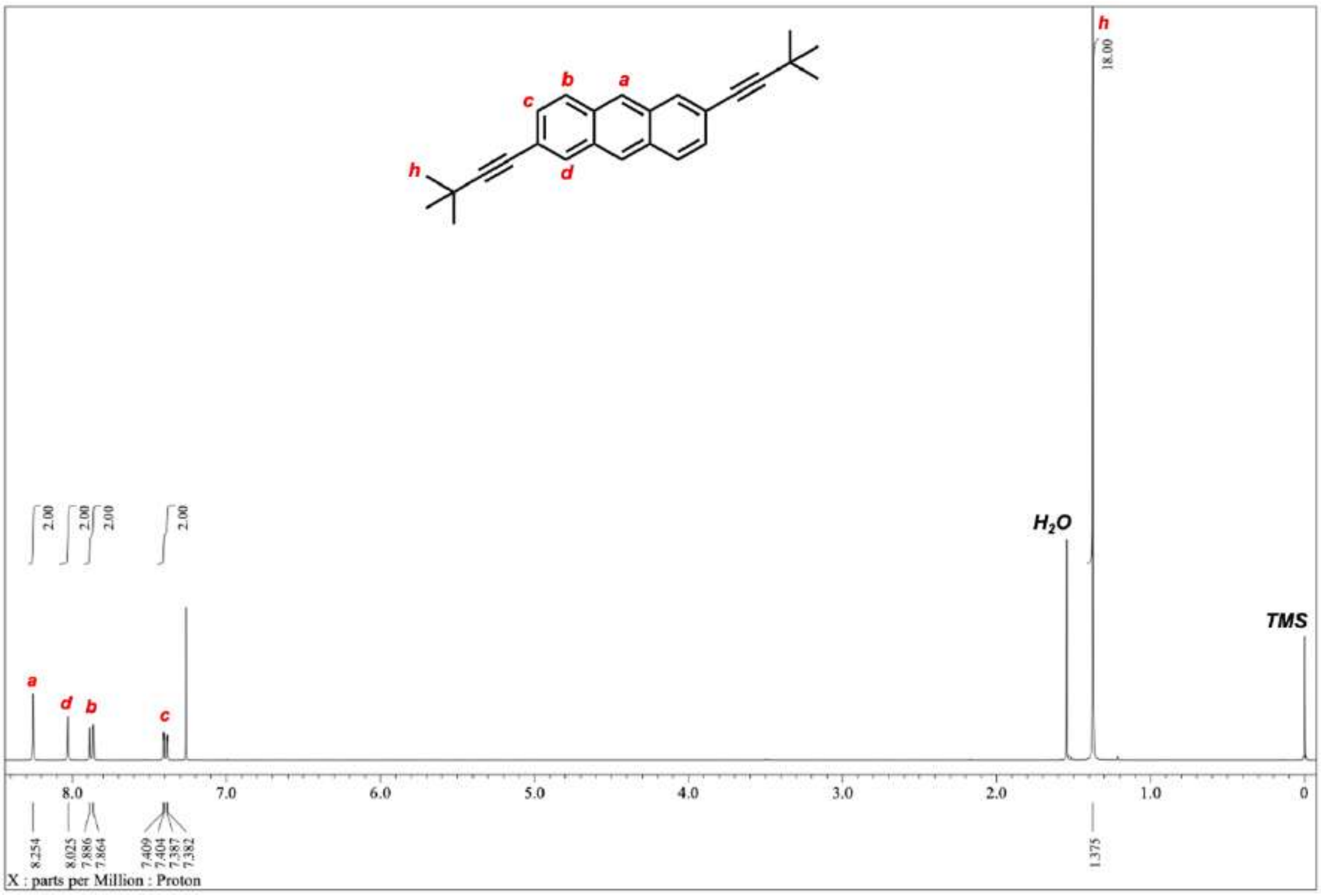

Figure S69. ^{1}H NMR spectrum of **BBEA**.

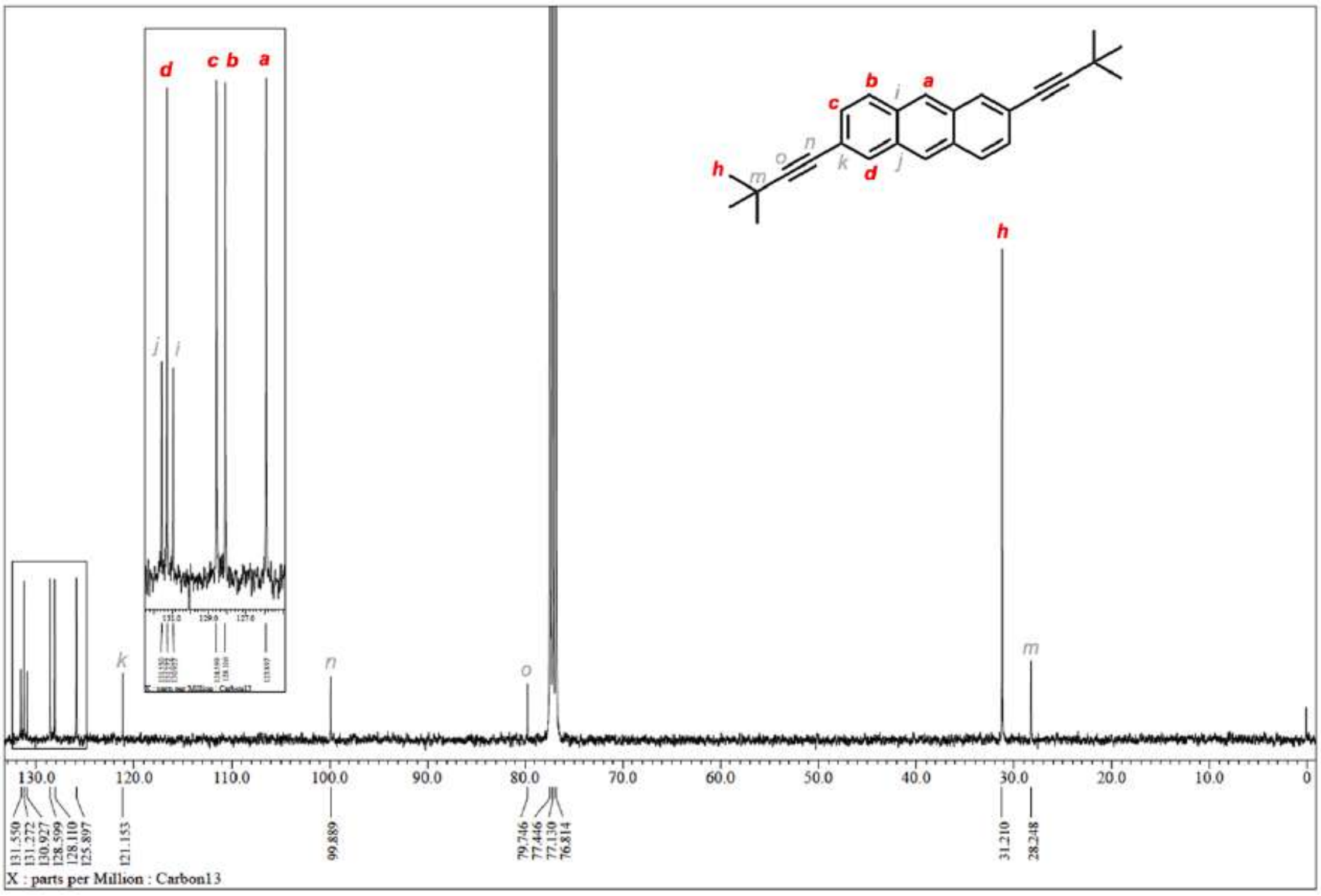

Figure S70. ^{13}C NMR spectra of **BBEA**.

High-resolution mass spectra

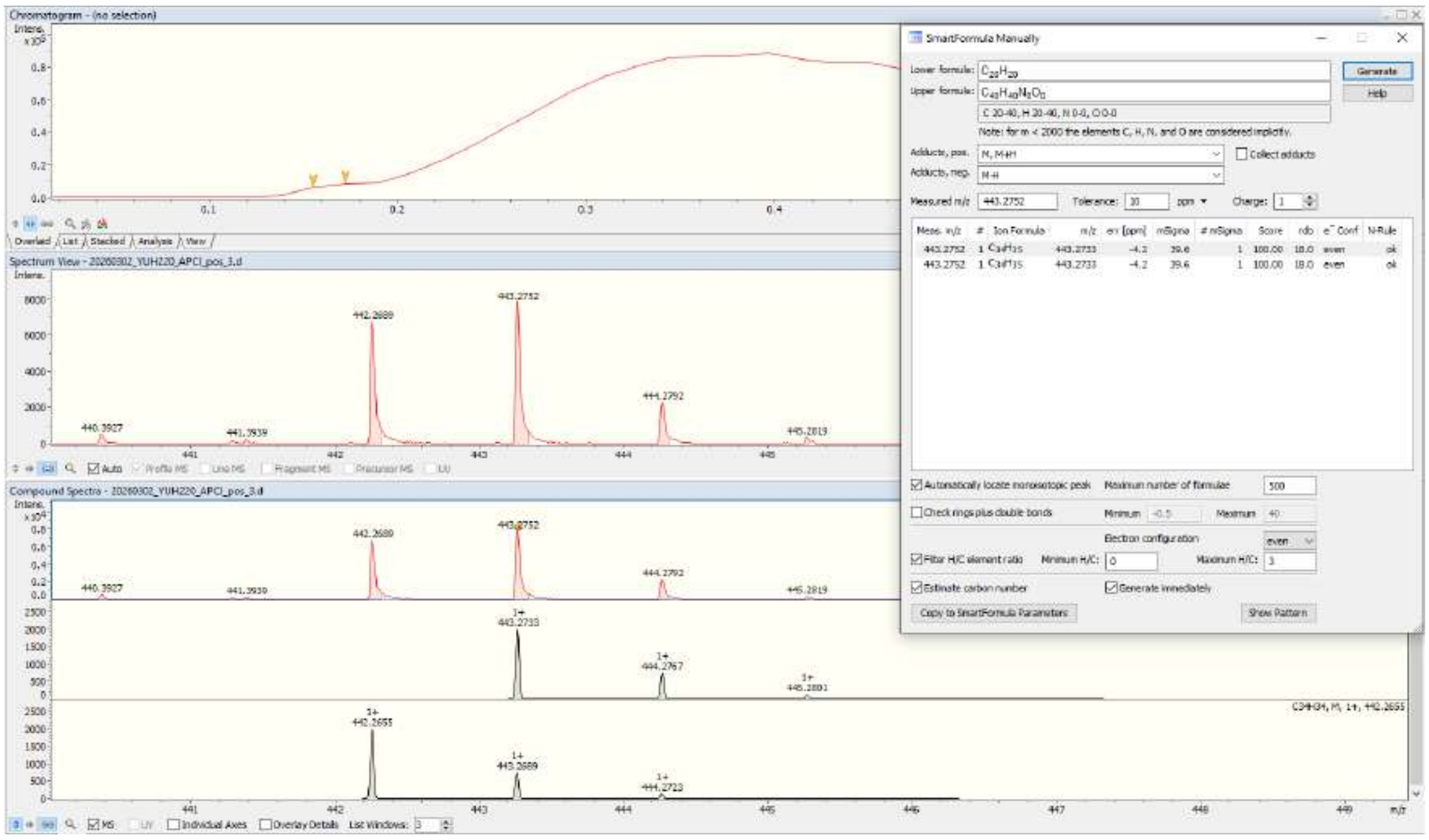

Figure S71. High-resolution mass spectrometric analysis of **BBPA**. Top: chromatogram. Middle: spectrum view. Bottom: compound spectra, showing the experimental spectrum (top), the protonated molecular ion (*M* + nH), and the neutral molecular ion (*M*).

checkCIF/PLATON reports

checkCIF/PLATON report

Structure factors have been supplied for datablock(s) exp_256

THIS REPORT IS FOR GUIDANCE ONLY. IF USED AS PART OF A REVIEW PROCEDURE FOR PUBLICATION, IT SHOULD NOT REPLACE THE EXPERTISE OF AN EXPERIENCED CRYSTALLOGRAPHIC REFEREE.

No syntax errors found. CIF dictionary Interpreting this report

Datablock: exp_256

Bond precision:	C-C = 0.0049 A		Wavelength=0.71073
Cell:	a=12.6713(3)	b=28.9509(5)	c=17.6785(5)
	alpha=90	beta=105.554(3)	gamma=90
Temperature:	103 K		

	Calculated	Reported
Volume	6247.8(3)	6247.8(3)
Space group	P 21	P 21
Hall group	P 2yb	P 2yb
Moiety formula	C34 H34	?
Sum formula	C34 H34	C34 H34
Mr	442.61	442.61
Dx,g cm-3	1.176	1.176
Z	10	10
Mu (mm-1)	0.066	0.066
F000	2380.0	2380.0
F000'	2380.83	
h,k,lmax	15,36,22	15,36,22
Nref	25570[13050]	23997
Tmin,Tmax		
Tmin'		

Correction method= Not given

```
Data completeness= 1.84/0.94        Theta(max)= 26.371

R(reflections)= 0.0495( 19177)                  wR2(reflections)=
                                                0.1302( 23997)
S = 1.023                   Npar= 1623
```

The following ALERTS were generated. Each ALERT has the format
test-name_ALERT_alert-type_alert-level.
Click on the hyperlinks for more details of the test.

Alert level B

```
PLAT919_ALERT_3_B Reflection # Likely Affected by the Beamstop ...          2 Check
               -1 -1  1,  -1  1  1,
```

Author Response: This alert arises because a small number of reflections occur at very

```
PLAT934_ALERT_3_B Number of (Iobs-Icalc)/Sigma(W) > 10 Outliers ..          2 Check
               -1 -1  1,  -1  1  1,
```

Author Response: This alert arises because a very small number of very low-angle reflec

---

Alert level C

```
STRVA01_ALERT_4_C            Flack test results are ambiguous.
           From the CIF: _refine_ls_abs_structure_Flack    0.600
           From the CIF: _refine_ls_abs_structure_Flack_su    1.000
PLAT052_ALERT_1_C Info on Absorption Correction Method   Not Given     Please Do !
PLAT053_ALERT_1_C Minimum Crystal Dimension Missing (or Error) ...     Please Check
PLAT054_ALERT_1_C Medium  Crystal Dimension Missing (or Error) ...     Please Check
PLAT055_ALERT_1_C Maximum Crystal Dimension Missing (or Error) ...     Please Check
PLAT220_ALERT_2_C NonSolvent   Resd 4  C    Ueq(max)/Ueq(min) Range        3.4 Ratio
PLAT241_ALERT_2_C High   'MainMol' Ueq as Compared to Neighbors of       C04H Check
PLAT241_ALERT_2_C High   'MainMol' Ueq as Compared to Neighbors of       C04I Check
PLAT242_ALERT_2_C Low    'MainMol' Ueq as Compared to Neighbors of       C026 Check
PLAT242_ALERT_2_C Low    'MainMol' Ueq as Compared to Neighbors of       C02J Check
PLAT334_ALERT_2_C Small <C-C> Benzene Dist.   C026     -C04H     .       1.37 Ang.
PLAT340_ALERT_3_C Low Bond Precision on  C-C Bonds ...............    0.00493 Ang.
PLAT410_ALERT_2_C Short Intra H...H Contact  H9       ..H01Z     .       1.99 Ang.
                                                      x,y,z  =      1_555 Check
PLAT790_ALERT_4_C Centre of Gravity not Within Unit-Cell: Resd.  #          1 Note
              C34 H34
PLAT907_ALERT_2_C Flack x > 0.5, Structure Needs to be Inverted? .       0.60 Check
PLAT911_ALERT_3_C Missing FCF Refl Between Thmin & STh/L=    0.600          2 Report
                0  2  1,  13  2  4,
PLAT918_ALERT_3_C Reflection(s) with I(obs) much Smaller I(calc) .          4 Check
                1 -1  0,   1  1  0,  -1 -1  1,  -1  1  1,
```

Alert level G

```
PLAT002_ALERT_2_G Number of Distance or Angle Restraints on AtSite         14 Note
PLAT032_ALERT_4_G Std. Uncertainty on Flack Parameter Value High .      1.000 Report
PLAT176_ALERT_4_G The CIF-Embedded .res File Contains SADI Records          6 Report
PLAT301_ALERT_3_G Main Residue  Disorder .............(Resd    1)          9% Note
PLAT301_ALERT_3_G Main Residue  Disorder .............(Resd    2)          9% Note
PLAT412_ALERT_2_G Short Intra XH3 .. XHn     H03P     ..H3C      .       2.01 Ang.
                                                        x,y,z  =      1_555 Check
PLAT432_ALERT_2_G Short Inter X...Y Contact  C00S     ..C017     .       3.16 Ang.
                                                        x,y,z  =      1_555 Check
PLAT720_ALERT_4_G Number of Unusual/Non-Standard Labels ..........        314 Note
              C001    H001    C002    C003    C004    H004    C005    C006
              H006    C007    H007    C008    H008    C009    C00A    C00B
              C00C    C00D    H00D    C00E    C00F    H00F    C00G    C00H
              H00H    C00I    H00I    C00J    H00J    C00K    H00K    C00L
              H00L    C00M    C00N    H00N    C00O    C00P    C00Q    H00Q
              C00R    H00R    C00S    H00S    C00T    H00T    C00U    C00V
              C00W    C00X    H00X    C00Y    H00Y    C00Z    H00Z    C010
              C011    C012    H012    C013    H013    C014    C015    H015
              C016    C017    H017    C018    C019    H019    C01A    H01A
              C01B    C01C    C01D    C01E    H01E    C01F    C01G    C01H
              C01I    H01I    C01J    H01J    C01K    H01K    C01L    C01M
              C01N    C01O    C01P    C01Q    C01R    H01R    C01S    C01T
              H01T    C01U    H01U    C01V    H01V    C01W    C01X    H01X
              C01Y    H01Y    C01Z    H01Z    C020    C021    C022    H022
              C023    C024    C025    H025    C026    C027    C028    H028
              C029    C02A    C02B    H02B    C02C    C02D    C02E    H02E
              C02F    H02F    C02G    H02G    C02H    H02H    C02I    H02I
              C02J    C02K    H02K    C02L    C02M    C02N    H02N    C02O
              C02P    C02Q    H02Q    C02R    H02R    C02S    C02T    H02T
              C02U    H02U    C02V    H02V    C02W    C02X    H02X    C02Y
              H02Y    C02Z    H02Z    C030    C031    H031    C032    C033
              H033    C034    H034    C035    C036    C037    H03D    H03F
              H03G    C038    H038    C039    C03A    H03L    C03B    C03C
              H03M    H03N    H03O    C03D    C03E    H03E    C03F    H03H
              H03I    H03J    C03G    H03Q    C03H    H03K    C03I    Ha
              C03J    H03Z    C03K    Hb      Hc      C03L    C03M    H03R
              C03N    Hd      He      C03O    C03P    H03P    C03Q    C03R
              H03A    H03B    H03C    C03S    H03S    C03T    H03T    C03U
              H03U    C03V    H03V    H03X    H03Y    C03W    H03W    C03X
              C03Y    H03     Hf      Hg      C03Z    C040    H040    C041
              H041    C042    H04P    H04Q    H04R    C043    H04     Hh
              Hi      C044    Hj      Hk      C045    Hl      Hm      C046
              Hn      Ho      C047    H04A    H04B    H04C    C048    Hp
              Hq      C049    H049    C04A    H04D    H04E    H04F    C04B
              Hr      Hs      C04C    C04D    Ht      Hu      C04E    Hv
              Hw      C04F    Hx      Hy      C04G    Hz      C04H    C04I
              C04J    C04K    C04L    H04G    H04H    H04I    C04M    H04J
              H04K    H04L    C04N    H04M    H04N    H04O    C04O    H04S
              H04T    H04U    C04P    H04V    H04W    H04X    C04Q    H04Y
              C04R    H04Z
PLAT790_ALERT_4_G Centre of Gravity not Within Unit-Cell: Resd.  #          2 Note
              C34 H34
PLAT790_ALERT_4_G Centre of Gravity not Within Unit-Cell: Resd.  #          3 Note
              C34 H34
PLAT790_ALERT_4_G Centre of Gravity not Within Unit-Cell: Resd.  #          4 Note
              C34 H34
PLAT790_ALERT_4_G Centre of Gravity not Within Unit-Cell: Resd.  #          5 Note
```

```
              C34 H34
PLAT802_ALERT_4_G CIF Input Record(s) with more than 80 Characters          2 Info
PLAT860_ALERT_3_G Number of Least-Squares Restraints ............           7 Note
PLAT883_ALERT_1_G Absent Datum for _atom_sites_solution_primary ..     Please Do !
PLAT899_ALERT_4_G SHELXL2018 is Outdated   and Succeeded by SHELXL     2019/3 Note
PLAT910_ALERT_3_G Missing FCF Reflection(s) Below Theta(Min)[Deg]=       1.77 Note
                1  0  0,   0  2  0,   0  0  1,   0  1  1,
PLAT912_ALERT_4_G Missing # of FCF Reflections Above STh/L=  0.600          9 Note
PLAT916_ALERT_2_G Hooft y and Flack x Parameter Values Differ by .       0.50 Check
PLAT941_ALERT_3_G Average HKL Measurement Multiplicity ..........         3.6 Low
PLAT969_ALERT_5_G The 'Henn et al.' R-Factor-gap value ..........       2.889 Note
              Predicted wR2: Based on SigI**2  4.51 or SHELX Weight 12.72
PLAT978_ALERT_2_G Number C-C Bonds with Positive Residual Density.         21 Info
PLAT992_ALERT_5_G Repd & Actual _reflns_number_gt Values Differ by          8 Check
```

```
   0 ALERT level A = Most likely a serious problem - resolve or explain
   2 ALERT level B = A potentially serious problem, consider carefully
  17 ALERT level C = Check. Ensure it is not caused by an omission or oversight
  23 ALERT level G = General information/check it is not something unexpected

   5 ALERT type 1 CIF construction/syntax error, inconsistent or missing data
  13 ALERT type 2 Indicator that the structure model may be wrong or deficient
  10 ALERT type 3 Indicator that the structure quality may be low
  12 ALERT type 4 Improvement, methodology, query or suggestion
   2 ALERT type 5 Informative message, check
```

It is advisable to attempt to resolve as many as possible of the alerts in all categories. Often the minor alerts point to easily fixed oversights, errors and omissions in your CIF or refinement strategy, so attention to these fine details can be worthwhile. It is up to the individual to critically assess their own results and, if necessary, seek expert advice.

PLATON version of 26/09/2025; check.def file version of 20/09/2025

Datablock exp_256 - ellipsoid plot

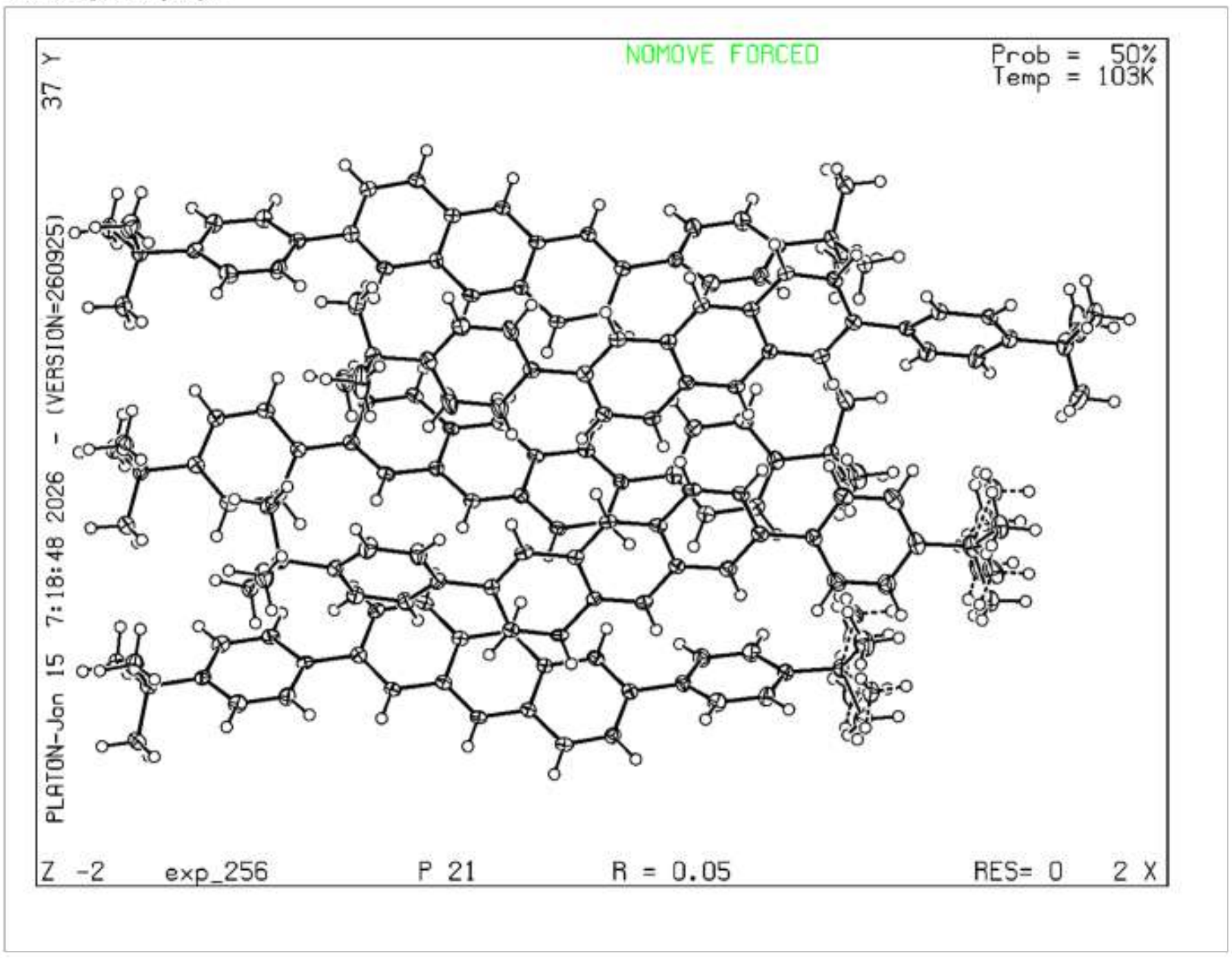

checkCIF/PLATON report

Structure factors have been supplied for datablock(s) exp_1015

THIS REPORT IS FOR GUIDANCE ONLY. IF USED AS PART OF A REVIEW PROCEDURE FOR PUBLICATION, IT SHOULD NOT REPLACE THE EXPERTISE OF AN EXPERIENCED CRYSTALLOGRAPHIC REFEREE.

No syntax errors found. CIF dictionary Interpreting this report

Datablock: exp_1015

Bond precision:	C-C = 0.0019 A		Wavelength=0.71073
Cell:	a=17.9884(12)	b=7.3638(3)	c=6.2625(4)
	alpha=90	beta=90.590(7)	gamma=90
Temperature:	103 K		

	Calculated	Reported
Volume	829.51(8)	829.51(8)
Space group	P 21/c	P 21/c
Hall group	-P 2ybc	-P 2ybc
Moiety formula	C26 H18	?
Sum formula	C26 H18	C26 H18
Mr	330.40	330.40
Dx,g cm-3	1.323	1.323
Z	2	2
Mu (mm-1)	0.075	0.075
F000	348.0	348.0
F000'	348.13	
h,k,lmax	25,10,8	22,10,8
Nref	2445	1581
Tmin,Tmax		
Tmin'		

Correction method= Not given

```
Data completeness= 0.647           Theta(max)= 30.039

                                                    wR2(reflections)=
R(reflections)= 0.0409( 1222)                       0.1116( 1581)
S = 1.062                  Npar= 118
```

The following ALERTS were generated. Each ALERT has the format
       test-name_ALERT_alert-type_alert-level.
Click on the hyperlinks for more details of the test.

Alert level A

```
PLAT029_ALERT_3_A _diffrn_measured_fraction_theta_full value Low .      0.841 Why?
```

Author Response: This alert arises because a fraction of high-angle reflections could n

---

Alert level B

```
PLAT911_ALERT_3_B Missing FCF Refl Between Thmin & STh/L=    0.600        239 Report
               -2  1  1,  -1  1  1,   5  8  1,   6  8  1,   7  8  1,   8  8  1,
               12  4  1,  12  5  1,  13  4  1,  13  5  1,  14  4  1,  14  5  1,
               15  4  1,  15  5  1,  16  4  1,  16  5  1,  17  4  1,  17  5  1,
               18  4  1,  19  4  1,  -4  0  2,  -4  1  2,  -3  0  2,  -3  1  2,
               -2  1  2,   4  8  2,   5  7  2,   5  8  2,   6  7  2,   6  8  2,
                   (  209 More Missing: see the .ckf listing file)
```

Author Response: This alert arises because some reflections between _min and STh/L = 0.

Alert level C

```
PLAT052_ALERT_1_C Info on Absorption Correction Method   Not Given      Please Do !
PLAT053_ALERT_1_C Minimum Crystal Dimension Missing (or Error) ...      Please Check
PLAT054_ALERT_1_C Medium  Crystal Dimension Missing (or Error) ...      Please Check
PLAT055_ALERT_1_C Maximum Crystal Dimension Missing (or Error) ...      Please Check
```

Alert level G

```
PLAT802_ALERT_4_G CIF Input Record(s) with more than 80 Characters          2 Info
PLAT883_ALERT_1_G Absent Datum for _atom_sites_solution_primary ..      Please Do !
PLAT899_ALERT_4_G SHELXL2018 is Outdated   and Succeeded by SHELXL      2019/3 Note
PLAT910_ALERT_3_G Missing FCF Reflection(s) Below Theta(Min)[Deg]=        2.26 Note
                1  0  0,
PLAT912_ALERT_4_G Missing # of FCF Reflections Above STh/L=  0.600         579 Note
PLAT941_ALERT_3_G Average HKL Measurement Multiplicity ...........         2.2 Low
PLAT950_ALERT_5_G Calculated (ThMax) and CIF-Reported Hmax Differ            3 Units
PLAT956_ALERT_1_G Calculated (ThMax) and Actual (FCF) Hmax Differ            3 Units
PLAT969_ALERT_5_G The 'Henn et al.' R-Factor-gap value ...........       2.855 Note
              Predicted wR2: Based on SigI**2  3.91 or SHELX Weight 10.51
PLAT978_ALERT_2_G Number C-C Bonds with Positive Residual Density.          13 Info
```

```
  1 ALERT level A = Most likely a serious problem - resolve or explain
  1 ALERT level B = A potentially serious problem, consider carefully
  4 ALERT level C = Check. Ensure it is not caused by an omission or oversight
 10 ALERT level G = General information/check it is not something unexpected

  6 ALERT type 1 CIF construction/syntax error, inconsistent or missing data
  1 ALERT type 2 Indicator that the structure model may be wrong or deficient
  4 ALERT type 3 Indicator that the structure quality may be low
  3 ALERT type 4 Improvement, methodology, query or suggestion
  2 ALERT type 5 Informative message, check
```

It is advisable to attempt to resolve as many as possible of the alerts in all categories. Often the minor alerts point to easily fixed oversights, errors and omissions in your CIF or refinement strategy, so attention to these fine details can be worthwhile. It is up to the individual to critically assess their own results and, if necessary, seek expert advice.

PLATON version of 26/09/2025; check.def file version of 20/09/2025

Datablock exp_1015 - ellipsoid plot

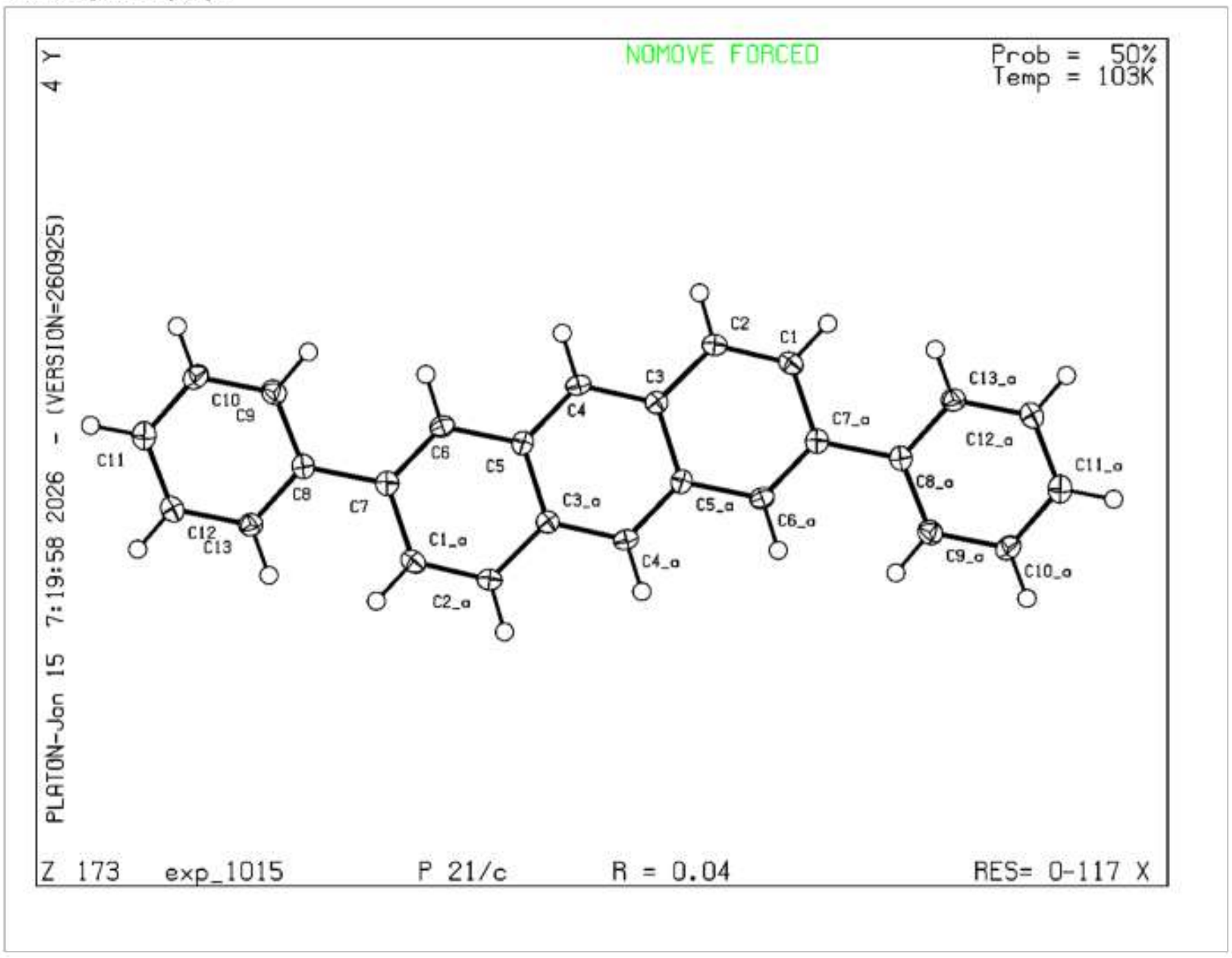

checkCIF/PLATON report

Structure factors have been supplied for datablock(s) exp_100

THIS REPORT IS FOR GUIDANCE ONLY. IF USED AS PART OF A REVIEW PROCEDURE FOR PUBLICATION, IT SHOULD NOT REPLACE THE EXPERTISE OF AN EXPERIENCED CRYSTALLOGRAPHIC REFEREE.

No syntax errors found. CIF dictionary Interpreting this report

Datablock: exp_100

```
Bond precision:   C-C = 0.0014 A          Wavelength=0.41330

Cell:             a=12.7202(1)    b=29.0778(2)       c=17.7050(1)
                  alpha=90        beta=105.587(1)    gamma=90
Temperature:      100 K

                 Calculated                 Reported
Volume           6307.81(8)                 6307.81(8)
Space group      P 21                       P 1 21 1
Hall group       P 2yb                      P 2yb
Moiety formula   C34 H34                    C34 H34
Sum formula      C34 H34                    C34 H34
Mr               442.61                     442.61
Dx,g cm-3        1.165                      1.165
Z                10                         10
Mu (mm-1)        0.031                      0.031
F000             2380.0                     2380.0
F000'            2379.68
h,k,lmax         22,52,31                   22,51,31
Nref             76373[ 38733]              64810
Tmin,Tmax
Tmin'

Correction method= Not given
```

Data completeness= 1.67/0.85 Theta(max)= 21.773

R(reflections)= 0.0404(59634) wR2(reflections)= 0.1157(64810)

S = 1.039 Npar= 1623

The following ALERTS were generated. Each ALERT has the format
test-name_ALERT_alert-type_alert-level.
Click on the hyperlinks for more details of the test.

Alert level B

```
DIFMN02_ALERT_2_B  The minimum difference density is < -0.1*ZMAX*1.00
            _refine_diff_density_min given =     -0.629
            Test value =     -0.600
```

Author Response: This alert arises because the minimum residual electron density slight

```
PLAT098_ALERT_2_B Large Reported Min.  (Negative) Residual Density      -0.63 eA-3
```

Author Response: This alert arises because the minimum negative residual electron densi

```
PLAT910_ALERT_3_B Missing FCF Reflection(s) Below Theta(Min)[Deg]=       1.46 Note
                1  0  0,    1  1  0,    0  2  0,    1  2  0,   -1  0  1,    0  0  1,
                1  0  1,   -1  1  1,    0  1  1,    1  1  1,   -1  2  1,    0  2  1,
                0  3  1,    0  0  2,    0  1  2,
```

Author Response: This alert arises because some very low-angle reflections below theta

---

Alert level C

```
DIFMN03_ALERT_1_C  The minimum difference density is < -0.1*ZMAX*0.75
            The relevant atom site should be identified.
STRVA01_ALERT_2_C           Chirality of atom sites is inverted?
           From the CIF: _refine_ls_abs_structure_Flack    0.800
           From the CIF: _refine_ls_abs_structure_Flack_su    1.000
PLAT052_ALERT_1_C Info on Absorption Correction Method   Not Given     Please Do !
PLAT053_ALERT_1_C Minimum Crystal Dimension Missing (or Error) ...     Please Check
PLAT054_ALERT_1_C Medium  Crystal Dimension Missing (or Error) ...     Please Check
PLAT055_ALERT_1_C Maximum Crystal Dimension Missing (or Error) ...     Please Check
PLAT241_ALERT_2_C High  MainResAtom  Ueq as Compared to Neighbours       C04F Check
PLAT241_ALERT_2_C High  MainResAtom  Ueq as Compared to Neighbours       C04G Check
PLAT242_ALERT_2_C Low   MainResAtom  Ueq as Compared to Neighbours       C029 Check
PLAT242_ALERT_2_C Low   MainResAtom  Ueq as Compared to Neighbours       C02T Check
PLAT250_ALERT_2_C Large U3/U1 Ratio for <U(i,j)> Tensor(Resd    4)        2.3 Note
PLAT410_ALERT_2_C Short Intra H...H Contact  H00N     ..H04      .       1.99 Ang.
                                                      x,y,z  =      1_555 Check
PLAT790_ALERT_4_C Centre of Gravity not Within Unit-Cell: Resd.  #           1 Note
              C34 H34
```

```
PLAT907_ALERT_2_C Flack x > 0.5, Structure Needs to be Inverted? .       0.80 Check
```

Alert level G

```
ABSMU01_ALERT_1_G  Calculation of _exptl_absorpt_correction_mu
                not performed for this radiation type.
PLAT002_ALERT_2_G Number of Distance or Angle Restraints on AtSite         14 Note
PLAT032_ALERT_4_G Std. Uncertainty on Flack Parameter Value High .      1.000 Report
PLAT142_ALERT_4_G s.u. on b - Axis Small or Missing .............    0.00020 Ang.
PLAT143_ALERT_4_G s.u. on c - Axis Small or Missing .............    0.00010 Ang.
PLAT176_ALERT_4_G The CIF-Embedded .res File Contains SADI Records          6 Report
PLAT230_ALERT_2_G Hirshfeld Test Diff for    C04B     --C04N     .       12.3 s.u.
PLAT301_ALERT_3_G Main Residue  Disorder ..............(Resd    1)         9% Note
PLAT302_ALERT_4_G Anion/Solvent/Minor-Residue Disorder (Resd    2)         9% Note
PLAT412_ALERT_2_G Short Intra XH3 .. XHn     H02X     ..H1A      .       2.00 Ang.
                                             x,y,z  =      1_555 Check
PLAT432_ALERT_2_G Short Inter X...Y Contact  C007     ..C00E     .       3.17 Ang.
                                             x,y,z  =      1_555 Check
PLAT720_ALERT_4_G Number of Unusual/Non-Standard Labels ..........        322 Note
              C001     H001     C002     H002     C003     H003     C004     H004
              C005     H005     C006     H006     C007     H007     C008     H008
              C009     H009     C00A     H00D     C00B     C00C     H00F     C00D
              H00I     C00E     H00E     C00F     C00G     H00G     C00H     H00H
              C00I     C00J     H00J     C00K     H00K     C00L     C00M     H00M
              C00N     H00N     C00O     H00O     C00P     H00P     C00Q     C00R
              H00R     C00S     H00S     C00T     C00U     H00U     C00V     H00V
              C00W     H00W     C00X     H00A     H00B     H00C     C00Y     C00Z
              C010     H010     C011     C012     C013     C014     C015     H015
              C016     H016     C017     H017     C018     H018     C019     C01A
              H01A     C01B     C01C     C01D     C01E     H01E     C01F     H01F
              C01G     H01G     C01H     C01I     C01J     H01J     C01K     C01L
              H01L     C01M     H01M     C01N     H01N     C01O     H01O     C01P
              C01Q     C01R     H01R     C01S     H01S     C01T     H01T     C01U
              C01V     C01W     C01X     H01X     C01Y     H01Y     C01Z     C020
              H020     C021     H021     C022     C023     H02D     H02F     H02G
              C024     C025     C026     C027     C028     C029     C02A     C02B
              H02J     C02C     C02D     C02E     H02E     C02F     C02G     H02K
              H02L     H02M     C02H     H02H     C02I     H02I     C02J     H02N
              C02K     C02L     C02M     C02N     H02O     C02O     C02P     C02Q
              H02Q     C02R     C02S     H02S     C02T     C02U     H02U     C02V
              C02W     C02X     H02X     C02Y     H02A     H02B     H02C     C02Z
              C030     C031     C032     H032     C033     H03E     H03F     H03G
              C034     H034     C035     C036     H036     C037     C038     H038
              C039     C03A     C03B     H03H     C03C     H03C     C03D     C03E
              Ha       C03F     H03I     H03J     H03M     C03G     H03      Hb
              Hc       C03H     Hd       He       C03I     C03J     H03A     H03B
              H03D     C03K     H03K     C03L     H03L     C03M     C03N     H03N
              C03O     H03O     H03P     H03Q     C03P     Hf       Hg       C03Q
              H03R     C03R     H03S     C03S     H03T     H03U     H03V     C03T
              H03W     C03U     Hh       Hi       C03V     C03W     C03X     H03X
              H03Z     Hj       C03Y     H03Y     C03Z     C040     C041     H041
              C042     H042     C043     C044     H04A     H04B     H04C     C045
              Hk       Hl       C046     H046     C047     H047     C048     C049
              H04V     H04W     H04X     C04A     Hm       Hn       C04B     C04C
              H04J     H04K     H04L     C04D     H04Y     Ho       Hp       C04E
              H04Z     Hq       Hr       C04F     H04      C04G     C04H     C04I
              C04J     Hs       Ht       C04K     Hu       Hv       C04L     H04M
```

```
              H04N    H04O    C04M    H04P    H04Q    H04R    C04N    H04S
              H04T    H04U    C04O    H04D    H04E    H04F    C04P    H04G
              H04H    H04I
PLAT802_ALERT_4_G CIF Input Record(s) with more than 80 Characters          3 Info
PLAT860_ALERT_3_G Number of Least-Squares Restraints ............           7 Note
PLAT883_ALERT_1_G Absent Datum for _atom_sites_solution_primary ..     Please Do !
PLAT912_ALERT_4_G Missing # of FCF Reflections Above STh/L=  0.600       4729 Note
PLAT969_ALERT_5_G The 'Henn et al.' R-Factor-gap value ...........      2.915 Note
              Predicted wR2: Based on SigI**2  3.97 or SHELX Weight 11.14
PLAT978_ALERT_2_G Number C-C Bonds with Positive Residual Density.         21 Info
PLAT992_ALERT_5_G Repd & Actual _reflns_number_gt Values Differ by         10 Check
PLAT994_ALERT_1_G SHELXL .ins Contains no or MERG 0 Instruction ..          ! Note
```

```
   0 ALERT level A = Most likely a serious problem - resolve or explain
   3 ALERT level B = A potentially serious problem, consider carefully
  14 ALERT level C = Check. Ensure it is not caused by an omission or oversight
  20 ALERT level G = General information/check it is not something unexpected

   8 ALERT type 1 CIF construction/syntax error, inconsistent or missing data
  15 ALERT type 2 Indicator that the structure model may be wrong or deficient
   3 ALERT type 3 Indicator that the structure quality may be low
   9 ALERT type 4 Improvement, methodology, query or suggestion
   2 ALERT type 5 Informative message, check
```

It is advisable to attempt to resolve as many as possible of the alerts in all categories. Often the minor alerts point to easily fixed oversights, errors and omissions in your CIF or refinement strategy, so attention to these fine details can be worthwhile. It is up to the individual to critically assess their own results and, if necessary, seek expert advice.

PLATON version of 15/01/2026; check.def file version of 02/01/2026

---

duplicate check

No duplication found

Datablock exp_100 - ellipsoid plot

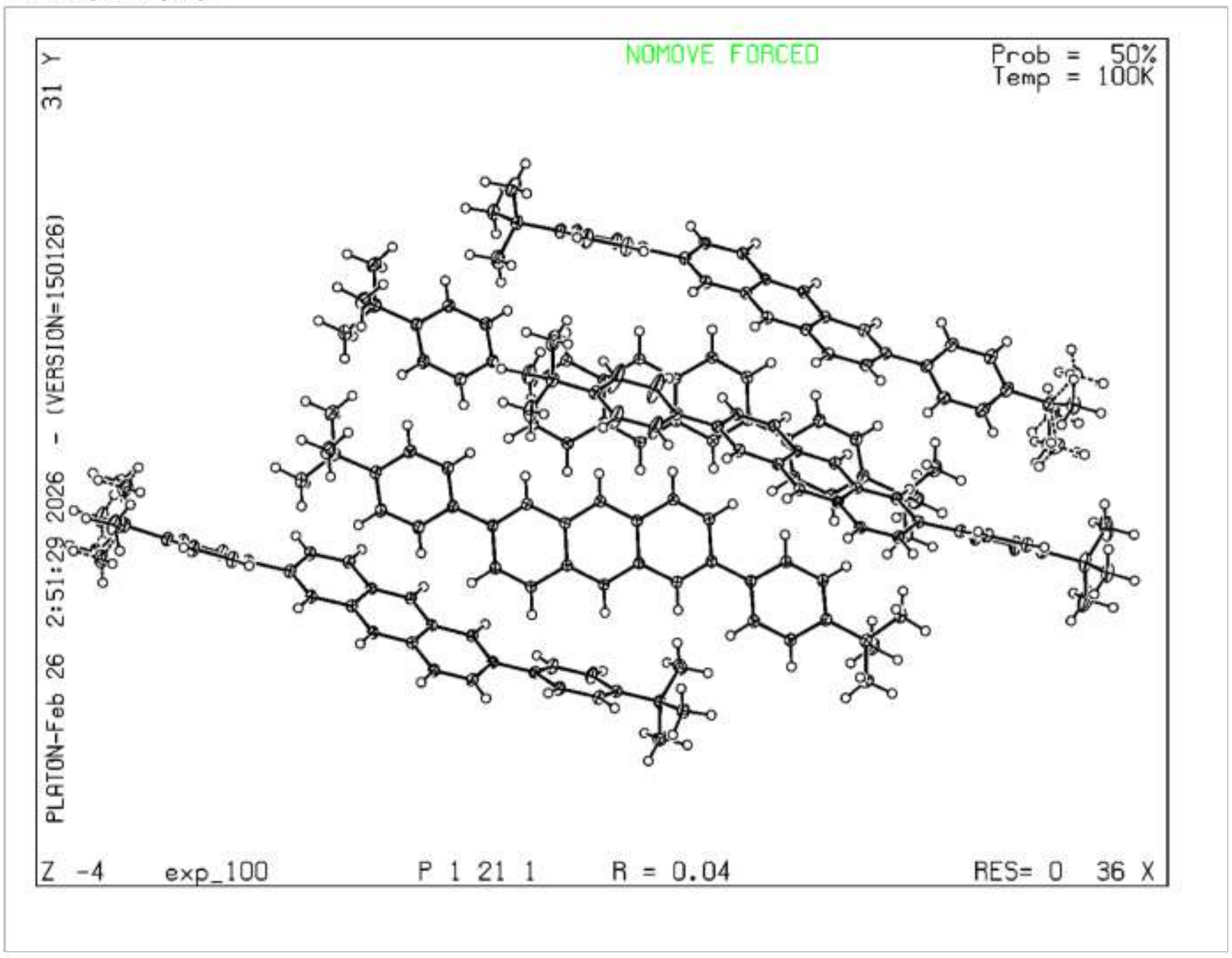

checkCIF/PLATON report

Structure factors have been supplied for datablock(s) exp_200

THIS REPORT IS FOR GUIDANCE ONLY. IF USED AS PART OF A REVIEW PROCEDURE FOR PUBLICATION, IT SHOULD NOT REPLACE THE EXPERTISE OF AN EXPERIENCED CRYSTALLOGRAPHIC REFEREE.

No syntax errors found. CIF dictionary Interpreting this report

Datablock: exp_200

```
Bond precision:   C-C = 0.0019 A           Wavelength=0.41330

Cell:             a=12.8679(1)     b=29.0348(2)        c=17.7691(1)
                  alpha=90         beta=104.967(1)     gamma=90
Temperature:      200 K

                 Calculated                  Reported
Volume           6413.61(8)                  6413.61(8)
Space group      P 21                        P 21
Hall group       P 2yb                       P 2yb
Moiety formula   C34 H34                     ?
Sum formula      C34 H34                     C34 H34
Mr               442.61                      442.61
Dx,g cm-3        1.146                       1.146
Z                10                          10
Mu (mm-1)        0.031                       0.030
F000             2380.0                      2380.0
F000'            2379.68
h,k,lmax         23,52,31                    22,51,31
Nref             77463[ 39289]               66005
Tmin,Tmax
Tmin'

Correction method= Not given
```

```
Data completeness= 1.68/0.85        Theta(max)= 21.755

R(reflections)= 0.0560( 51901)                      wR2(reflections)=
                                                    0.1670( 66005)
S = 1.024                    Npar= 1747
```

The following ALERTS were generated. Each ALERT has the format
test-name_ALERT_alert-type_alert-level.
Click on the hyperlinks for more details of the test.

Alert level B

```
PLAT910_ALERT_3_B Missing FCF Reflection(s) Below Theta(Min)[Deg]=      1.46 Note
                1  0  0,   1  1  0,   0  2  0,   1  2  0,  -1  0  1,   0  0  1,
                1  0  1,  -1  1  1,   0  1  1,   1  1  1,  -1  2  1,   0  2  1,
                0  3  1,   0  0  2,   0  1  2,
```

Author Response: This alert arises because some very low-angle reflections below theta

```
PLAT934_ALERT_3_B Number of (Iobs-Icalc)/Sigma(W) > 10 Outliers ..         4 Check
               -4 10  5,  -7  0 13,  -7 -3 14,  -7  3 14,
```

Author Response: This alert arises because a small number of intensity outliers are pre

---

Alert level C

```
DIFMX02_ALERT_1_C  The maximum difference density is > 0.1*ZMAX*0.75
            The relevant atom site should be identified.
STRVA01_ALERT_2_C           Chirality of atom sites is inverted?
           From the CIF: _refine_ls_abs_structure_Flack    1.300
           From the CIF: _refine_ls_abs_structure_Flack_su    1.000
PLAT052_ALERT_1_C Info on Absorption Correction Method   Not Given     Please Do !
PLAT053_ALERT_1_C Minimum Crystal Dimension Missing (or Error) ...     Please Check
PLAT054_ALERT_1_C Medium  Crystal Dimension Missing (or Error) ...     Please Check
PLAT055_ALERT_1_C Maximum Crystal Dimension Missing (or Error) ...     Please Check
PLAT097_ALERT_2_C Large Reported Max.  (Positive) Residual Density       0.51 eA-3
PLAT220_ALERT_2_C NonSolvent    Resd 2  C   Ueq(max)/Ueq(min) Range       3.2 Ratio
PLAT220_ALERT_2_C NonSolvent    Resd 3  C   Ueq(max)/Ueq(min) Range       3.2 Ratio
PLAT220_ALERT_2_C NonSolvent    Resd 4  C   Ueq(max)/Ueq(min) Range       3.1 Ratio
PLAT230_ALERT_2_C Hirshfeld Test Diff for    C03H     --C049     .        5.4 s.u.
PLAT234_ALERT_4_C Large Hirshfeld Difference C035     --C10      .       0.18 Ang.
PLAT242_ALERT_2_C Low    'MainMol' Ueq as Compared to Neighbors of       C03M Check
PLAT242_ALERT_2_C Low    'MainMol' Ueq as Compared to Neighbors of       C03W Check
PLAT242_ALERT_2_C Low    'MainMol' Ueq as Compared to Neighbors of       C024 Check
PLAT242_ALERT_2_C Low    'MainMol' Ueq as Compared to Neighbors of       C02C Check
PLAT242_ALERT_2_C Low    'MainMol' Ueq as Compared to Neighbors of       C03H Check
PLAT242_ALERT_2_C Low    'MainMol' Ueq as Compared to Neighbors of       C03V Check
PLAT242_ALERT_2_C Low    'MainMol' Ueq as Compared to Neighbors of       C03Z Check
PLAT242_ALERT_2_C Low    'MainMol' Ueq as Compared to Neighbors of       C03K Check
PLAT242_ALERT_2_C Low    'MainMol' Ueq as Compared to Neighbors of       C035 Check
```

```
PLAT907_ALERT_2_C Flack x > 0.5, Structure Needs to be Inverted? .       1.30 Check
```

Alert level G

```
ABSMU01_ALERT_1_G  Calculation of _exptl_absorpt_correction_mu
                not performed for this radiation type.
PLAT002_ALERT_2_G Number of Distance or Angle Restraints on AtSite        42 Note
PLAT032_ALERT_4_G Std. Uncertainty on Flack Parameter Value High .     1.000 Report
PLAT142_ALERT_4_G s.u. on b - Axis Small or Missing .............    0.00020 Ang.
PLAT143_ALERT_4_G s.u. on c - Axis Small or Missing .............    0.00010 Ang.
PLAT176_ALERT_4_G The CIF-Embedded .res File Contains SADI Records        18 Report
PLAT230_ALERT_2_G Hirshfeld Test Diff for    C03M     --C04H     .       7.9 s.u.
PLAT230_ALERT_2_G Hirshfeld Test Diff for    C03W     --C04L     .       5.0 s.u.
PLAT230_ALERT_2_G Hirshfeld Test Diff for    C035     --C04I     .       5.9 s.u.
PLAT301_ALERT_3_G Main Residue  Disorder ..............(Resd    1)       18% Note
PLAT301_ALERT_3_G Main Residue  Disorder ..............(Resd    2)        9% Note
PLAT301_ALERT_3_G Main Residue  Disorder ..............(Resd    3)        9% Note
PLAT301_ALERT_3_G Main Residue  Disorder ..............(Resd    4)        9% Note
PLAT301_ALERT_3_G Main Residue  Disorder ..............(Resd    5)        9% Note
PLAT412_ALERT_2_G Short Intra XH3 .. XHn     H03A     ..H3B      .      1.84 Ang.
                                                      x,y,z  =      1_555 Check
PLAT412_ALERT_2_G Short Intra XH3 .. XHn     H50F     ..H046     .      2.11 Ang.
                                                      x,y,z  =      1_555 Check
PLAT412_ALERT_2_G Short Intra XH3 .. XHn     H03U     ..H04      .      2.00 Ang.
                                                      x,y,z  =      1_555 Check
PLAT412_ALERT_2_G Short Intra XH3 .. XHn     H046     ..H04Y     .      2.08 Ang.
                                                      x,y,z  =      1_555 Check
PLAT412_ALERT_2_G Short Intra XH3 .. XHn     H038     ..H6C      .      1.72 Ang.
                                                      x,y,z  =      1_555 Check
PLAT432_ALERT_2_G Short Inter X...Y Contact  C00P     ..C00U     .      3.18 Ang.
                                                      x,y,z  =      1_555 Check
PLAT720_ALERT_4_G Number of Unusual/Non-Standard Labels ..........       325 Note
              C001    H001    C002    H002    C003    H003    C004    H004
              C005    C006    H006    C007    H007    C008    H008    C009
              H009    C00A    H00A    C00B    C00C    C00D    C00E    H00E
              C00F    C00G    H00G    C00H    H00H    C00I    H00I    C00J
              H00J    C00K    H00K    C00L    H00L    C00M    C00N    C00O
              H00O    C00P    H00P    C00Q    C00R    C00S    H00S    C00T
              H00T    C00U    H00U    C00V    C00W    H00W    C00X    C00Y
              H00Y    C00Z    H00Z    C010    C011    H011    C012    H012
              C013    H013    C014    C015    H015    C016    C017    C018
              C019    H019    C01A    C01B    C01C    H01C    C01D    C01E
              C01F    C01G    H01G    C01H    C01I    C01J    C01K    C01L
              C01M    C01N    C01O    H01O    C01P    C01Q    H01Q    C01R
              H01R    C01S    H01S    C01T    C01U    C01V    H01V    C01W
              H01W    C01X    H01X    C01Y    H01Y    C01Z    H01Z    C020
              H020    C021    C022    C023    H023    C024    C025    H025
              C026    C027    H027    C028    C029    C02A    C02B    H02E
              C02C    C02D    H02D    C02E    C02F    H02F    C02G    C02H
              H02H    C02I    H02I    C02J    H02J    C02K    C02L    H02L
              C02M    H02M    C02N    H02N    C02O    C02P    H02P    C02Q
              H02Q    C02R    H02R    C02S    H02A    H02B    H02C    C02T
              H02T    C02U    C02V    C02W    H02W    C02X    H02X    C02Y
              H02Y    C02Z    C030    C031    H03W    H03X    H03Y    C032
              C033    H033    C034    C035    C036    H036    C037    H037
              C038    H038    C039    C03A    H03A    C03B    C03C    H03C
              C03D    H03N    C03E    H03Z    C03F    C03G    C03H    C03I
```

```
              H03F     H03G     H03H     C03J     H03J     C03K     C03L     H03L
              C03M     C03N     H03      C03O     H03O     H03Q     H03T     C03P
              H03P     C03Q     H03V     C03R     H03R     C03S     H03S     C03T
              Ha       C03U     H03U     C03V     C03W     C03X     H03I     H03K
              H03M     C03Y     H03B     H03D     H03E     C03Z     C040     Hb
              Hc       C041     H041     C042     Hd       He       C043     Hf
              Hg       C044     H044     C045     Hh       Hi       C046     H046
              C047     Hj       Hk       C048     H048     C049     H04S     H04T
              H04U     C04A     Hl       Hm       C04B     H04A     H04B     H04C
              C04C     Hn       Ho       C04D     Hp       Hq       C04E     H04D
              H04E     H04F     C04F     H04V     H04W     H04X     C04G     H04G
              H04H     H04I     C04H     H04J     H04K     H04L     C04I     Hr
              Hs       C04J     H04M     H04N     H04O     C04K     Ht       Hu
              C04L     H04P     H04Q     H04R     C04M     Hv       Hw       C04N
              Hx       Hy       C04O     H04Y     Hz       C04P     H04Z     C04Q
              H04      C0       H0A      H0B      H0C
PLAT773_ALERT_2_G Check long C-C Bond in CIF: C03K     --C6              1.71 Ang.
PLAT773_ALERT_2_G Check long C-C Bond in CIF: C03M     --C14             1.71 Ang.
PLAT802_ALERT_4_G CIF Input Record(s) with more than 80 Characters          2 Info
PLAT860_ALERT_3_G Number of Least-Squares Restraints .............         19 Note
PLAT883_ALERT_1_G Absent Datum for _atom_sites_solution_primary ..     Please Do !
PLAT899_ALERT_4_G SHELXL2018 is Outdated   and Succeeded by SHELXL     2019/3 Note
PLAT912_ALERT_4_G Missing # of FCF Reflections Above STh/L=  0.600       4691 Note
PLAT916_ALERT_2_G Hooft y and Flack x Parameter Values Differ by .       0.60 Check
PLAT969_ALERT_5_G The 'Henn et al.' R-Factor-gap value ..........       2.389 Note
              Predicted wR2: Based on SigI**2  6.99 or SHELX Weight 16.31
PLAT978_ALERT_2_G Number C-C Bonds with Positive Residual Density.         19 Info
PLAT992_ALERT_5_G Repd & Actual _reflns_number_gt Values Differ by          7 Check
```

```
   0 ALERT level A = Most likely a serious problem - resolve or explain
   2 ALERT level B = A potentially serious problem, consider carefully
  22 ALERT level C = Check. Ensure it is not caused by an omission or oversight
  32 ALERT level G = General information/check it is not something unexpected

   7 ALERT type 1 CIF construction/syntax error, inconsistent or missing data
  30 ALERT type 2 Indicator that the structure model may be wrong or deficient
   8 ALERT type 3 Indicator that the structure quality may be low
   9 ALERT type 4 Improvement, methodology, query or suggestion
   2 ALERT type 5 Informative message, check
```

It is advisable to attempt to resolve as many as possible of the alerts in all categories. Often the minor alerts point to easily fixed oversights, errors and omissions in your CIF or refinement strategy, so attention to these fine details can be worthwhile. It is up to the individual to critically assess their own results and, if necessary, seek expert advice.

PLATON version of 26/09/2025; check.def file version of 20/09/2025

Datablock exp_200 - ellipsoid plot

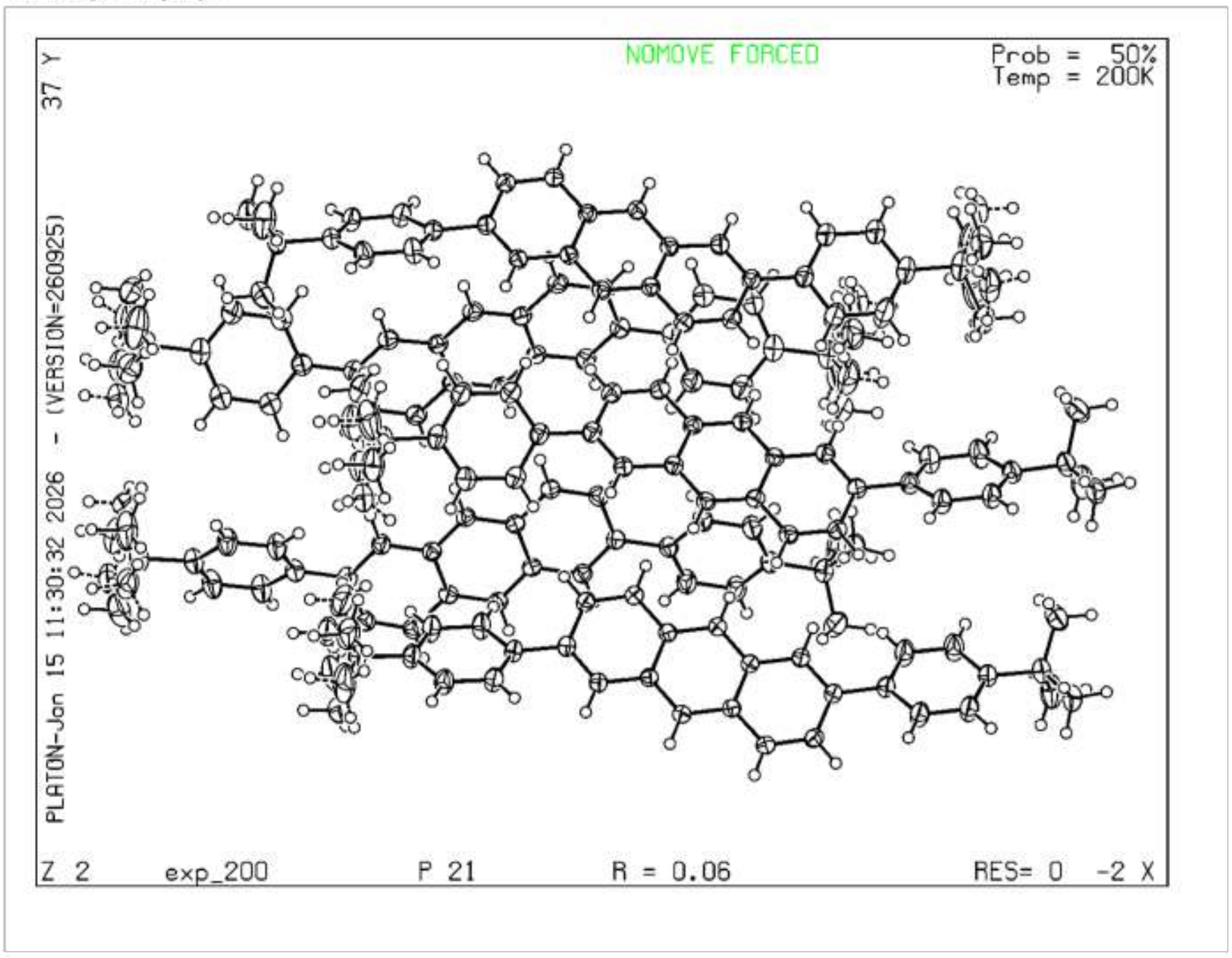

checkCIF/PLATON report

Structure factors have been supplied for datablock(s) exp_300

THIS REPORT IS FOR GUIDANCE ONLY. IF USED AS PART OF A REVIEW PROCEDURE FOR PUBLICATION, IT SHOULD NOT REPLACE THE EXPERTISE OF AN EXPERIENCED CRYSTALLOGRAPHIC REFEREE.

No syntax errors found.   CIF dictionary   Interpreting this report

Datablock: exp_300

```
Bond precision:    C-C = 0.0028 A            Wavelength=0.41330

Cell:              a=12.9889(2)     b=29.2286(3)       c=17.7849(2)
                   alpha=90         beta=104.489(1)    gamma=90
Temperature:       300 K

                  Calculated                  Reported
Volume            6537.25(14)                 6537.25(14)
Space group       P 21                        P 21
Hall group        P 2yb                       P 2yb
Moiety formula    C34 H34                     ?
Sum formula       C34 H34                     C34 H34
Mr                442.61                      442.61
Dx,g cm-3         1.124                       1.124
Z                 10                          10
Mu (mm-1)         0.030                       0.030
F000              2380.0                      2380.0
F000'             2379.68
h,k,lmax          23,52,31                    23,51,31
Nref              79338[ 40236]               67239
Tmin,Tmax
Tmin'

Correction method= Not given
```

```
Data completeness= 1.67/0.85        Theta(max)= 21.787

R(reflections)= 0.0649( 37881)                          wR2(reflections)=
                                                        0.2170( 67239)
S = 0.987                  Npar= 1858
```

The following ALERTS were generated. Each ALERT has the format
test-name_ALERT_alert-type_alert-level.
Click on the hyperlinks for more details of the test.

Alert level B

```
PLAT213_ALERT_2_B Atom C20             has ADP max/min Ratio .....        4.1 prolat
```

Author Response: This alert arises because atom C20 exhibits a strongly anisotropic dis

```
PLAT234_ALERT_4_B Large Hirshfeld Difference C02T     --C9       .       0.28 Ang.
```

Author Response: This alert arises because the Hirshfeld rigid-bond test indicates a sl

```
PLAT242_ALERT_2_B Low   MainResAtom  Ueq as Compared to Neighbours       C02T Check
```

Author Response: This alert arises because atom C02T exhibits a slightly lower Ueq than

```
PLAT910_ALERT_3_B Missing FCF Reflection(s) Below Theta(Min)[Deg]=       1.51 Note
                1  0  0,   1  1  0,   0  2  0,   1  2  0,  -1  0  1,   0  0  1,
                1  0  1,  -1  1  1,   0  1  1,   1  1  1,  -1  2  1,   0  2  1,
                0  3  1,  -1  0  2,   0  0  2,   0  1  2,
```

Author Response: This alert arises because some very low-angle reflections below theta

---

Alert level C

```
STRVA01_ALERT_2_C           Chirality of atom sites is inverted?
           From the CIF: _refine_ls_abs_structure_Flack    6.800
           From the CIF: _refine_ls_abs_structure_Flack_su    1.000
PLAT052_ALERT_1_C Info on Absorption Correction Method   Not Given     Please Do !
PLAT053_ALERT_1_C Minimum Crystal Dimension Missing (or Error) ...     Please Check
PLAT054_ALERT_1_C Medium  Crystal Dimension Missing (or Error) ...     Please Check
PLAT055_ALERT_1_C Maximum Crystal Dimension Missing (or Error) ...     Please Check
PLAT213_ALERT_2_C Atom C16             has ADP max/min Ratio .....        3.4 prolat
```

Author Response: This alert arises because atom C20 exhibits a strongly anisotropic dis

```
PLAT213_ALERT_2_C Atom C17             has ADP max/min Ratio .....        3.1 prolat
```

Author Response: This alert arises because atom C20 exhibits a strongly anisotropic dis

```
PLAT213_ALERT_2_C Atom C18             has ADP max/min Ratio .....        3.6 oblate
```

Author Response: This alert arises because atom C20 exhibits a strongly anisotropic dis

```
PLAT213_ALERT_2_C Atom C19             has ADP max/min Ratio .....        3.2 prolat
```

Author Response: This alert arises because atom C20 exhibits a strongly anisotropic dis

```
PLAT220_ALERT_2_C NonSolvent   Resd 1  C   Ueq(max)/Ueq(min) Range        4.6 Ratio
PLAT221_ALERT_2_C Solv./Anion  Resd 2  C   Ueq(max)/Ueq(min) Range        5.5 Ratio
PLAT222_ALERT_3_C NonSolvent Resd 1  H   Uiso(max)/Uiso(min) Range        4.4 Ratio
PLAT223_ALERT_4_C Solv./Anion  Resd 2  H   Ueq(max)/Ueq(min) Range        6.5 Ratio
PLAT234_ALERT_4_C Large Hirshfeld Difference C02T     --C11      .       0.19 Ang.
```

Author Response: This alert arises because the Hirshfeld rigid-bond test indicates a sl

```
PLAT234_ALERT_4_C Large Hirshfeld Difference C03O     --C5       .       0.19 Ang.
```

Author Response: This alert arises because the Hirshfeld rigid-bond test indicates a sl

```
PLAT234_ALERT_4_C Large Hirshfeld Difference C03O     --C0       .       0.20 Ang.
```

Author Response: This alert arises because the Hirshfeld rigid-bond test indicates a sl

```
PLAT241_ALERT_2_C High  MainResAtom  Ueq as Compared to Neighbours       C03Q Check
PLAT241_ALERT_2_C High  MainResAtom  Ueq as Compared to Neighbours       C040 Check
PLAT241_ALERT_2_C High  MainResAtom  Ueq as Compared to Neighbours       C03H Check
PLAT241_ALERT_2_C High  MainResAtom  Ueq as Compared to Neighbours       C03M Check
PLAT242_ALERT_2_C Low   MainResAtom  Ueq as Compared to Neighbours       C021 Check
```

Author Response: This alert arises because atom C02T exhibits a slightly lower Ueq than

```
PLAT242_ALERT_2_C Low   MainResAtom  Ueq as Compared to Neighbours       C02I Check
```

Author Response: This alert arises because atom C02T exhibits a slightly lower Ueq than

```
PLAT242_ALERT_2_C Low   MainResAtom  Ueq as Compared to Neighbours       C02P Check
```

Author Response: This alert arises because atom C02T exhibits a slightly lower Ueq than

```
PLAT242_ALERT_2_C Low   MainResAtom  Ueq as Compared to Neighbours       C033 Check
```

Author Response: This alert arises because atom C02T exhibits a slightly lower Ueq than

```
PLAT242_ALERT_2_C Low   MainResAtom  Ueq as Compared to Neighbours       C03J Check
```

Author Response: This alert arises because atom C02T exhibits a slightly lower Ueq than

```
PLAT242_ALERT_2_C Low   MainResAtom  Ueq as Compared to Neighbours       C042 Check
```

Author Response: This alert arises because atom C02T exhibits a slightly lower Ueq than

```
PLAT242_ALERT_2_C Low   MainResAtom  Ueq as Compared to Neighbours       C039 Check
```

Author Response: This alert arises because atom C02T exhibits a slightly lower Ueq than

```
PLAT242_ALERT_2_C Low   MainResAtom  Ueq as Compared to Neighbours       C00K Check
```

Author Response: This alert arises because atom C02T exhibits a slightly lower Ueq than

```
PLAT242_ALERT_2_C Low   MainResAtom  Ueq as Compared to Neighbours       C024 Check
```

Author Response: This alert arises because atom C02T exhibits a slightly lower Ueq than

```
PLAT242_ALERT_2_C Low   MainResAtom  Ueq as Compared to Neighbours       C02H Check
```

Author Response: This alert arises because atom C02T exhibits a slightly lower Ueq than

```
PLAT242_ALERT_2_C Low   MainResAtom  Ueq as Compared to Neighbours       C03G Check
```

Author Response: This alert arises because atom C02T exhibits a slightly lower Ueq than

```
PLAT242_ALERT_2_C Low   MainResAtom  Ueq as Compared to Neighbours       C02A Check
```

Author Response: This alert arises because atom C02T exhibits a slightly lower Ueq than

```
PLAT242_ALERT_2_C Low   MainResAtom  Ueq as Compared to Neighbours       C02Q Check
```

Author Response: This alert arises because atom C02T exhibits a slightly lower Ueq than

```
PLAT242_ALERT_2_C Low   MainResAtom  Ueq as Compared to Neighbours       C03O Check
```

Author Response: This alert arises because atom C02T exhibits a slightly lower Ueq than

```
PLAT242_ALERT_2_C Low   MainResAtom  Ueq as Compared to Neighbours       C03V Check
```

Author Response: This alert arises because atom C02T exhibits a slightly lower Ueq than

```
PLAT242_ALERT_2_C Low   MainResAtom  Ueq as Compared to Neighbours       C02W Check
```

Author Response: This alert arises because atom C02T exhibits a slightly lower Ueq than

```
PLAT242_ALERT_2_C Low   MainResAtom  Ueq as Compared to Neighbours       C032 Check
```

Author Response: This alert arises because atom C02T exhibits a slightly lower Ueq than

```
PLAT906_ALERT_3_C Large K Value in the Analysis of Variance ......      5.832 Check
PLAT906_ALERT_3_C Large K Value in the Analysis of Variance ......      2.007 Check
PLAT907_ALERT_2_C Flack x > 0.5, Structure Needs to be Inverted? .       6.80 Check
PLAT911_ALERT_3_C Missing FCF Refl Between Thmin & STh/L=    0.600          4 Report
                1  5  1,   2  0  2,   4  0  5,  -5  0  7,
```

Alert level G

```
ABSMU01_ALERT_1_G  Calculation of _exptl_absorpt_correction_mu
                not performed for this radiation type.
PLAT002_ALERT_2_G Number of Distance or Angle Restraints on AtSite         72 Note
PLAT032_ALERT_4_G Std. Uncertainty on Flack Parameter Value High .      1.000 Report
PLAT033_ALERT_4_G Flack x Value Deviates > 3.0 * Sigma from Zero .      6.800 Note
PLAT176_ALERT_4_G The CIF-Embedded .res File Contains SADI Records         48 Report
PLAT191_ALERT_3_G A Non-default SADI Restraint Value has been used     0.0400 Report
PLAT191_ALERT_3_G A Non-default SADI Restraint Value has been used     0.0400 Report
PLAT191_ALERT_3_G A Non-default SADI Restraint Value has been used     0.0400 Report
PLAT191_ALERT_3_G A Non-default SADI Restraint Value has been used     0.0400 Report
PLAT191_ALERT_3_G A Non-default SADI Restraint Value has been used     0.0400 Report
PLAT191_ALERT_3_G A Non-default SADI Restraint Value has been used     0.0400 Report
PLAT191_ALERT_3_G A Non-default SADI Restraint Value has been used     0.0400 Report
PLAT191_ALERT_3_G A Non-default SADI Restraint Value has been used     0.0400 Report
PLAT191_ALERT_3_G A Non-default SADI Restraint Value has been used     0.0400 Report
PLAT191_ALERT_3_G A Non-default SADI Restraint Value has been used     0.0400 Report
PLAT191_ALERT_3_G A Non-default SADI Restraint Value has been used     0.0400 Report
PLAT191_ALERT_3_G A Non-default SADI Restraint Value has been used     0.0400 Report
PLAT230_ALERT_2_G Hirshfeld Test Diff for    C02P     --C16      .        7.7 s.u.
PLAT301_ALERT_3_G Main Residue  Disorder .............(Resd    1)        41% Note
PLAT302_ALERT_4_G Anion/Solvent/Minor-Residue Disorder (Resd    2)        29% Note
PLAT302_ALERT_4_G Anion/Solvent/Minor-Residue Disorder (Resd    3)         9% Note
PLAT302_ALERT_4_G Anion/Solvent/Minor-Residue Disorder (Resd    4)         9% Note
PLAT333_ALERT_2_G Large Aver C6-Ring C-C Dist C00R     -C010     .       1.42 Ang.
PLAT333_ALERT_2_G Large Aver C6-Ring C-C Dist C00B     -C01S     .       1.42 Ang.
PLAT333_ALERT_2_G Large Aver C6-Ring C-C Dist C01C     -C01F     .       1.42 Ang.
PLAT335_ALERT_2_G Check Large C6 Ring C-C Range C021     -C20            0.18 Ang.
```

```
PLAT410_ALERT_2_G Short Intra H...H Contact  H016      ..H16      .       1.96 Ang.
                                                       x,y,z  =     1_555 Check
PLAT410_ALERT_2_G Short Intra H...H Contact  H01A      ..H1BA     .       1.97 Ang.
                                                       x,y,z  =     1_555 Check
PLAT410_ALERT_2_G Short Intra H...H Contact  H02D      ..H20      .       2.14 Ang.
                                                       x,y,z  =     1_555 Check
PLAT410_ALERT_2_G Short Intra H...H Contact  H00R      ..H02K     .       2.14 Ang.
                                                       x,y,z  =     1_555 Check
PLAT410_ALERT_2_G Short Intra H...H Contact  H00R      ..H13      .       2.04 Ang.
                                                       x,y,z  =     1_555 Check
PLAT410_ALERT_2_G Short Intra H...H Contact  H01P      ..H14      .       2.04 Ang.
                                                       x,y,z  =     1_555 Check
PLAT412_ALERT_2_G Short Intra XH3 .. XHn     H03A      ..H52D     .       2.13 Ang.
                                                       x,y,z  =     1_555 Check
PLAT412_ALERT_2_G Short Intra XH3 .. XHn     H02X      ..H5C      .       2.05 Ang.
                                                       x,y,z  =     1_555 Check
PLAT720_ALERT_4_G Number of Unusual/Non-Standard Labels ..........         321 Note
              C001    H001    C002    C003    H003    C004    C005    C006
              H006    C007    C008    H008    C009    C00A    H00A    C00B
              C00C    H00C    C00D    H00D    C00E    H00E    C00F    H00F
              C00G    H00G    C00H    H00H    C00I    H00I    C00J    C00K
              C00L    C00M    H00M    C00N    H00N    C00O    H00O    C00P
              C00Q    C00R    H00R    C00S    H00S    C00T    C00U    C00V
              H00V    C00W    C00X    C00Y    H00Y    C00Z    C010    H010
              C011    C012    C013    H013    C014    C015    C016    H016
              C017    C018    H018    C019    H019    C01A    H01A    C01B
              H01B    C01C    C01D    C01E    C01F    H01F    C01G    C01H
              C01I    H01I    C01J    H01J    C01K    H01K    C01L    C01M
              C01N    H01N    C01O    C01P    H01P    C01Q    H01Q    C01R
              H01R    C01S    H01S    C01T    H01T    C01U    C01V    C01W
              H01W    C01X    C01Y    H01Y    C01Z    C020    H020    C021
              C022    C023    C024    C025    C026    H026    C027    H027
              C028    C029    H029    C02A    C02B    H02B    C02C    H02C
              C02D    H02D    C02E    H02E    C02F    H02F    C02G    H02G
              C02H    C02I    C02J    H02J    C02K    H02K    C02L    C02M
              H02M    C02N    H02N    C02O    H02O    C02P    C02Q    C02R
              H02A    H02H    H02I    C02S    C02T    C02U    H02U    C02V
              C02W    C02X    H02X    C02Y    H02Y    C02Z    C030    H030
              C031    H031    C032    C033    C034    H034    C035    C036
              H036    C037    H037    C038    C039    C03A    H03A    C03B
              H03P    C03C    H03C    C03D    H03F    C03E    H03T    C03F
              H03U    C03G    C03H    H03H    C03I    H03I    C03J    C03K
              H03K    C03L    H03L    C03M    H03M    C03N    H03N    C03O
              C03P    H03V    H03W    H03Y    C03Q    H03Q    C03R    H03R
              C03S    H03S    C03T    H03     Ha      C03U    H03B    H03D
              H03E    C03V    C03W    Hb      Hc      C03X    H03X    C03Y
              H03G    H03J    H03O    C03Z    H03Z    C040    H040    C041
              Hd      He      C042    C043    Hf      Hg      C044    Hh
              Hi      C045    H045    C046    Hj      Hk      C047    Hl
              Hm      C048    Hn      Ho      C049    H04V    H04W    H04X
              C04A    Hp      Hq      C04B    H04Y    Hr      Hs      C04C
              Ht      Hu      C04D    H04Z    Hv      Hw      C04E    Hx
              Hy      C04F    H04     Hz      C04G    H04A    H04B    H04C
              C04H    H04D    H04E    H04F    C04I    H04G    H04H    H04I
              C04K    C04L    H04J    H04K    H04L    C04M    C04N    H04M
              H04N    H04O    C04O    H04P    H04Q    H04R    C04J    H04S
              H04T    H04U    C0      H0A     H0B     H0C     H1AA    H1BA
```

```
              H1CA
PLAT721_ALERT_1_G Bond    Calc     0.97000, Rep     0.96000 Dev...       0.01 Ang.
              C0       -H0B             1_555   1_555 ........     #  404 Check
PLAT802_ALERT_4_G CIF Input Record(s) with more than 80 Characters          4 Info
PLAT860_ALERT_3_G Number of Least-Squares Restraints ............          49 Note
PLAT883_ALERT_1_G Absent Datum for _atom_sites_solution_primary ..     Please Do !
PLAT899_ALERT_4_G SHELXL2018 is Outdated   and Succeeded by SHELXL     2019/3 Note
PLAT912_ALERT_4_G Missing # of FCF Reflections Above STh/L=  0.600       4960 Note
PLAT913_ALERT_3_G Missing # of Very Strong Reflections in FCF ....          2 Note
                1  5  1,   2  0  2,
PLAT916_ALERT_2_G Hooft y and Flack x Parameter Values Differ by .       5.60 Check
PLAT933_ALERT_2_G Number of HKL-OMIT Records in Embedded .res File          6 Note
               -5  0  7,  -4 10  5,   1 -5  1,   1  5  1,   2  0  2,   4  0  5,
PLAT969_ALERT_5_G The 'Henn et al.' R-Factor-gap value ..........      3.093 Note
              Predicted wR2: Based on SigI**2  7.02 or SHELX Weight 21.98 Note
PLAT978_ALERT_2_G Number C-C Bonds with Positive Residual Density.         20 Info
PLAT992_ALERT_5_G Repd & Actual _reflns_number_gt Values Differ by          3 Check
```

```
   0 ALERT level A = Most likely a serious problem - resolve or explain
   4 ALERT level B = A potentially serious problem, consider carefully
  41 ALERT level C = Check. Ensure it is not caused by an omission or oversight
  47 ALERT level G = General information/check it is not something unexpected

   7 ALERT type 1 CIF construction/syntax error, inconsistent or missing data
  48 ALERT type 2 Indicator that the structure model may be wrong or deficient
  20 ALERT type 3 Indicator that the structure quality may be low
  15 ALERT type 4 Improvement, methodology, query or suggestion
   2 ALERT type 5 Informative message, check
```

It is advisable to attempt to resolve as many as possible of the alerts in all categories. Often the minor alerts point to easily fixed oversights, errors and omissions in your CIF or refinement strategy, so attention to these fine details can be worthwhile. It is up to the individual to critically assess their own results and, if necessary, seek expert advice.

PLATON version of 23/04/2026; check.def file version of 30/03/2026

---

duplicate check

No duplication found

Datablock exp_300 - ellipsoid plot

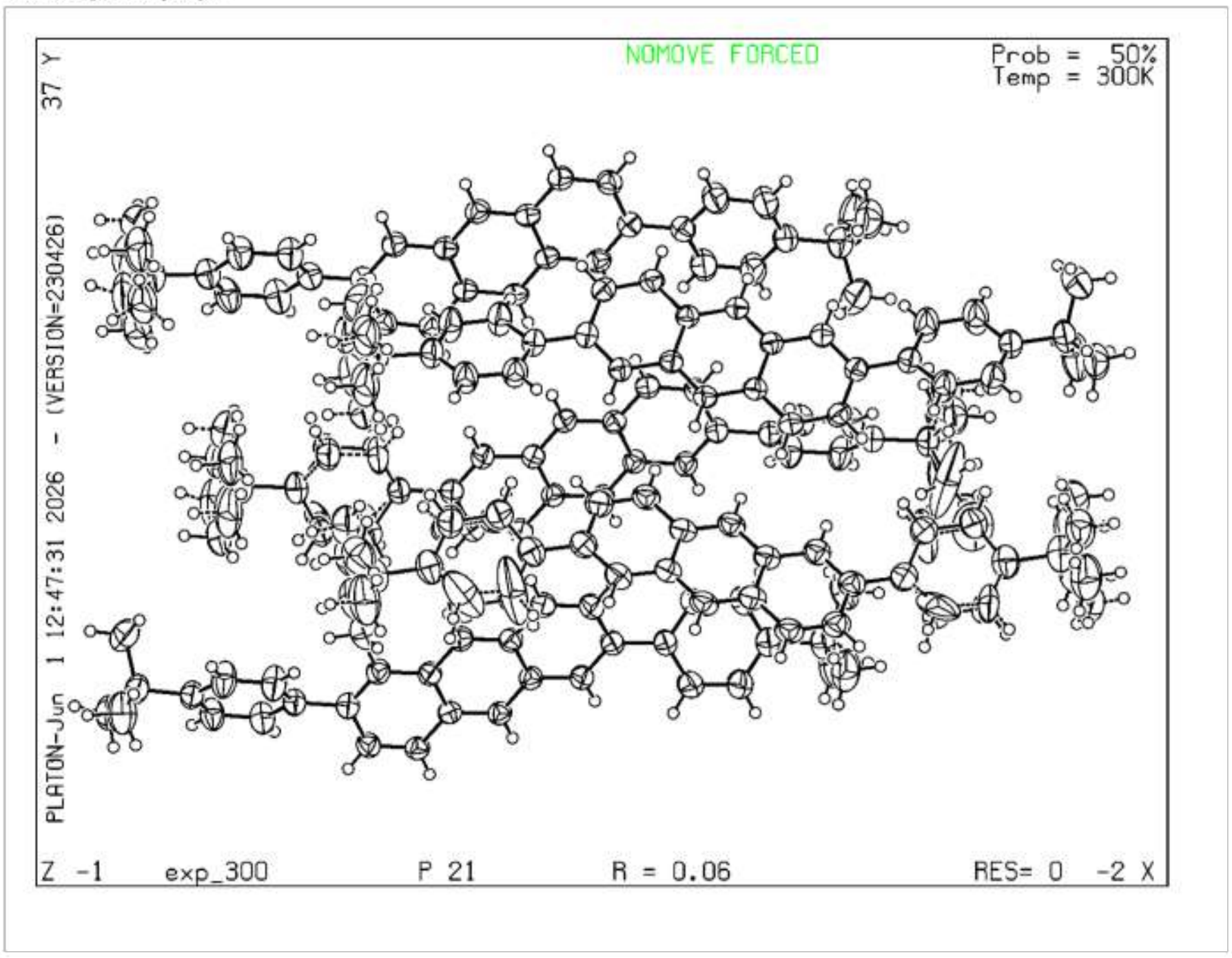

checkCIF/PLATON report

Structure factors have been supplied for datablock(s) exp_400

THIS REPORT IS FOR GUIDANCE ONLY. IF USED AS PART OF A REVIEW PROCEDURE FOR PUBLICATION, IT SHOULD NOT REPLACE THE EXPERTISE OF AN EXPERIENCED CRYSTALLOGRAPHIC REFEREE.

No syntax errors found. CIF dictionary Interpreting this report

Datablock: exp_400

```
Bond precision:   C-C = 0.0048 A            Wavelength=0.41330

Cell:             a=13.1496(3)     b=29.4925(5)        c=17.7763(4)
                  alpha=90         beta=103.947(2)     gamma=90
Temperature:      400 K

                 Calculated                  Reported
Volume           6690.7(3)                   6690.7(2)
Space group      P 21                        P 21
Hall group       P 2yb                       P 2yb
Moiety formula   C34 H34                     ?
Sum formula      C34 H34                     C34 H34
Mr               442.61                      442.61
Dx,g cm-3        1.099                       1.099
Z                10                          10
Mu (mm-1)        0.030                       0.029
F000             2380.0                      2380.0
F000'            2379.68
h,k,lmax         23,52,31                    23,52,31
Nref             81141[ 41145]               68620
Tmin,Tmax
Tmin'

Correction method= Not given
```

Data completeness= 1.67/0.85   Theta(max)= 21.788

R(reflections)= 0.0747(22235)   wR2(reflections)= 0.2783(68620)

S = 0.920   Npar= 1908

The following ALERTS were generated. Each ALERT has the format
test-name_ALERT_alert-type_alert-level.
Click on the hyperlinks for more details of the test.

Alert level A

```
SHFSU01_ALERT_2_A  The absolute value of parameter shift to su ratio > 0.20
            Absolute value of the parameter shift to su ratio given   1.783
            Additional refinement cycles may be required.
```

Author Response: This alert arises because the final refinement cycle shows a relativel

```
PLAT080_ALERT_2_A Maximum Shift/Error ............................       1.78 Why ?
```

Author Response: This alert arises because the final refinement cycle shows a maximum s

```
PLAT234_ALERT_4_A Large Hirshfeld Difference C037     --C20      .       0.50 Ang.
```

Author Response: This alert arises because the Hirshfeld rigid-bond test shows relative

```
PLAT234_ALERT_4_A Large Hirshfeld Difference C03Q     --C6A      .       0.37 Ang.
```

Author Response: This alert arises because the Hirshfeld rigid-bond test shows relative

---

Alert level B

```
PLAT026_ALERT_3_B Ratio Observed / Unique Reflections (too) Low ..        32% Check
```

Author Response: This alert arises because many reflections are weak and fall below the

```
PLAT213_ALERT_2_B Atom C20             has ADP max/min Ratio .....        4.5 oblate
```

Author Response: This alert arises because atom C20 exhibits a strongly anisotropic dis

```
PLAT234_ALERT_4_B Large Hirshfeld Difference C034     --C2A      .       0.26 Ang.
```

Author Response: This alert arises because the Hirshfeld rigid-bond test shows relative

```
PLAT234_ALERT_4_B Large Hirshfeld Difference C03L     --C45A     .       0.28 Ang.
```

Author Response: This alert arises because the Hirshfeld rigid-bond test shows relative

```
PLAT242_ALERT_2_B Low    'MainMol' Ueq as Compared to Neighbors of       C02V Check
```

Author Response: This alert arises because some atoms in the main molecule show slightl

```
PLAT242_ALERT_2_B Low    'MainMol' Ueq as Compared to Neighbors of       C034 Check
```

Author Response: This alert arises because some atoms in the main molecule show slightl

```
PLAT242_ALERT_2_B Low    'MainMol' Ueq as Compared to Neighbors of       C037 Check
```

Author Response: This alert arises because some atoms in the main molecule show slightl

```
PLAT242_ALERT_2_B Low    'MainMol' Ueq as Compared to Neighbors of       C03Q Check
```

Author Response: This alert arises because some atoms in the main molecule show slightl

```
PLAT242_ALERT_2_B Low    'MainMol' Ueq as Compared to Neighbors of       C03L Check
```

Author Response: This alert arises because some atoms in the main molecule show slightl

```
PLAT242_ALERT_2_B Low    'MainMol' Ueq as Compared to Neighbors of       C03Y Check
```

Author Response: This alert arises because some atoms in the main molecule show slightl

```
PLAT910_ALERT_3_B Missing FCF Reflection(s) Below Theta(Min)[Deg]=       1.51 Note
                1  0  0,   1  1  0,   0  2  0,   1  2  0,  -1  0  1,   0  0  1,
                1  0  1,  -1  1  1,   0  1  1,   1  1  1,  -1  2  1,   0  2  1,
                0  3  1,  -1  0  2,   0  0  2,   0  1  2,
```

Author Response: This alert arises because some very low-angle reflections below theta

---

Alert level C

```
STRVA01_ALERT_4_C           Flack parameter is too small
           From the CIF: _refine_ls_abs_structure_Flack   -0.600
           From the CIF: _refine_ls_abs_structure_Flack_su    1.000
PLAT052_ALERT_1_C Info on Absorption Correction Method   Not Given     Please Do !
PLAT053_ALERT_1_C Minimum Crystal Dimension Missing (or Error) ...     Please Check
```

```
PLAT054_ALERT_1_C Medium  Crystal Dimension Missing (or Error) ...     Please Check
PLAT055_ALERT_1_C Maximum Crystal Dimension Missing (or Error) ...     Please Check
PLAT084_ALERT_3_C High wR2 Value (i.e. > 0.25) ..................       0.28 Report
PLAT213_ALERT_2_C Atom C1C             has ADP max/min Ratio .....        3.9 prolat
```

Author Response: This alert arises because atom C20 exhibits a strongly anisotropic dis

```
PLAT220_ALERT_2_C NonSolvent   Resd 2  C   Ueq(max)/Ueq(min) Range        4.9 Ratio
PLAT220_ALERT_2_C NonSolvent   Resd 3  C   Ueq(max)/Ueq(min) Range        4.1 Ratio
PLAT220_ALERT_2_C NonSolvent   Resd 4  C   Ueq(max)/Ueq(min) Range        5.5 Ratio
PLAT220_ALERT_2_C NonSolvent   Resd 5  C   Ueq(max)/Ueq(min) Range        4.3 Ratio
PLAT222_ALERT_3_C NonSolvent Resd 2  H   Uiso(max)/Uiso(min) Range        5.9 Ratio
PLAT222_ALERT_3_C NonSolvent Resd 3  H   Uiso(max)/Uiso(min) Range        4.9 Ratio
PLAT222_ALERT_3_C NonSolvent Resd 4  H   Uiso(max)/Uiso(min) Range        6.4 Ratio
PLAT222_ALERT_3_C NonSolvent Resd 5  H   Uiso(max)/Uiso(min) Range        4.8 Ratio
PLAT230_ALERT_2_C Hirshfeld Test Diff for    C004     --C00U     .        6.0 s.u.
PLAT230_ALERT_2_C Hirshfeld Test Diff for    C011     --C027     .        6.5 s.u.
PLAT234_ALERT_4_C Large Hirshfeld Difference C26A     --C031     .       0.16 Ang.
```

Author Response: This alert arises because the Hirshfeld rigid-bond test shows relative

```
PLAT234_ALERT_4_C Large Hirshfeld Difference C01Y     --C03H     .       0.17 Ang.
```

Author Response: This alert arises because the Hirshfeld rigid-bond test shows relative

```
PLAT234_ALERT_4_C Large Hirshfeld Difference C031     --C29      .       0.21 Ang.
```

Author Response: This alert arises because the Hirshfeld rigid-bond test shows relative

```
PLAT234_ALERT_4_C Large Hirshfeld Difference C03O     --C15A     .       0.22 Ang.
```

Author Response: This alert arises because the Hirshfeld rigid-bond test shows relative

```
PLAT241_ALERT_2_C High   'MainMol' Ueq as Compared to Neighbors of       C03H Check
PLAT241_ALERT_2_C High   'MainMol' Ueq as Compared to Neighbors of       C03W Check
PLAT241_ALERT_2_C High   'MainMol' Ueq as Compared to Neighbors of       C02R Check
PLAT241_ALERT_2_C High   'MainMol' Ueq as Compared to Neighbors of       C035 Check
PLAT241_ALERT_2_C High   'MainMol' Ueq as Compared to Neighbors of       C03B Check
PLAT241_ALERT_2_C High   'MainMol' Ueq as Compared to Neighbors of       C03M Check
PLAT241_ALERT_2_C High   'MainMol' Ueq as Compared to Neighbors of       C02S Check
PLAT241_ALERT_2_C High   'MainMol' Ueq as Compared to Neighbors of       C032 Check
PLAT241_ALERT_2_C High   'MainMol' Ueq as Compared to Neighbors of       C03K Check
PLAT241_ALERT_2_C High   'MainMol' Ueq as Compared to Neighbors of       C02W Check
PLAT241_ALERT_2_C High   'MainMol' Ueq as Compared to Neighbors of       C02Z Check
PLAT241_ALERT_2_C High   'MainMol' Ueq as Compared to Neighbors of       C03N Check
PLAT241_ALERT_2_C High   'MainMol' Ueq as Compared to Neighbors of       C03X Check
PLAT241_ALERT_2_C High   'MainMol' Ueq as Compared to Neighbors of       C038 Check
```

```
PLAT241_ALERT_2_C High   'MainMol' Ueq as Compared to Neighbors of       C03C Check
PLAT241_ALERT_2_C High   'MainMol' Ueq as Compared to Neighbors of       C03D Check
PLAT241_ALERT_2_C High   'MainMol' Ueq as Compared to Neighbors of       C03S Check
PLAT242_ALERT_2_C Low    'MainMol' Ueq as Compared to Neighbors of        C44 Check
```

Author Response: This alert arises because some atoms in the main molecule show slightl

```
PLAT242_ALERT_2_C Low    'MainMol' Ueq as Compared to Neighbors of       C01Y Check
```

Author Response: This alert arises because some atoms in the main molecule show slightl

```
PLAT242_ALERT_2_C Low    'MainMol' Ueq as Compared to Neighbors of       C02D Check
```

Author Response: This alert arises because some atoms in the main molecule show slightl

```
PLAT242_ALERT_2_C Low    'MainMol' Ueq as Compared to Neighbors of       C031 Check
```

Author Response: This alert arises because some atoms in the main molecule show slightl

```
PLAT242_ALERT_2_C Low    'MainMol' Ueq as Compared to Neighbors of       C00F Check
```

Author Response: This alert arises because some atoms in the main molecule show slightl

```
PLAT242_ALERT_2_C Low    'MainMol' Ueq as Compared to Neighbors of       C013 Check
```

Author Response: This alert arises because some atoms in the main molecule show slightl

```
PLAT242_ALERT_2_C Low    'MainMol' Ueq as Compared to Neighbors of       C018 Check
```

Author Response: This alert arises because some atoms in the main molecule show slightl

```
PLAT242_ALERT_2_C Low    'MainMol' Ueq as Compared to Neighbors of       C014 Check
```

Author Response: This alert arises because some atoms in the main molecule show slightl

```
PLAT242_ALERT_2_C Low    'MainMol' Ueq as Compared to Neighbors of       C01M Check
```

Author Response: This alert arises because some atoms in the main molecule show slightl

```
PLAT242_ALERT_2_C Low    'MainMol' Ueq as Compared to Neighbors of       C01U Check
```

Author Response: This alert arises because some atoms in the main molecule show slightl

PLAT242_ALERT_2_C Low 'MainMol' Ueq as Compared to Neighbors of C02U Check

Author Response: This alert arises because some atoms in the main molecule show slightl

PLAT242_ALERT_2_C Low 'MainMol' Ueq as Compared to Neighbors of C03J Check

Author Response: This alert arises because some atoms in the main molecule show slightl

PLAT242_ALERT_2_C Low 'MainMol' Ueq as Compared to Neighbors of C021 Check

Author Response: This alert arises because some atoms in the main molecule show slightl

PLAT242_ALERT_2_C Low 'MainMol' Ueq as Compared to Neighbors of C022 Check

Author Response: This alert arises because some atoms in the main molecule show slightl

PLAT242_ALERT_2_C Low 'MainMol' Ueq as Compared to Neighbors of C029 Check

Author Response: This alert arises because some atoms in the main molecule show slightl

PLAT242_ALERT_2_C Low 'MainMol' Ueq as Compared to Neighbors of C02E Check

Author Response: This alert arises because some atoms in the main molecule show slightl

PLAT242_ALERT_2_C Low 'MainMol' Ueq as Compared to Neighbors of C03O Check

Author Response: This alert arises because some atoms in the main molecule show slightl

PLAT242_ALERT_2_C Low 'MainMol' Ueq as Compared to Neighbors of C01R Check

Author Response: This alert arises because some atoms in the main molecule show slightl

PLAT242_ALERT_2_C Low 'MainMol' Ueq as Compared to Neighbors of C02G Check

Author Response: This alert arises because some atoms in the main molecule show slightl

PLAT242_ALERT_2_C Low 'MainMol' Ueq as Compared to Neighbors of C02P Check

Author Response: This alert arises because some atoms in the main molecule show slightl

```
PLAT242_ALERT_2_C Low    'MainMol' Ueq as Compared to Neighbors of       C03E Check
```

Author Response: This alert arises because some atoms in the main molecule show slightl

```
PLAT260_ALERT_2_C Large Average Ueq of Residue Including      C006      0.129 Check
PLAT260_ALERT_2_C Large Average Ueq of Residue Including        C1      0.121 Check
PLAT260_ALERT_2_C Large Average Ueq of Residue Including      C001      0.114 Check
PLAT260_ALERT_2_C Large Average Ueq of Residue Including        C4      0.123 Check
PLAT260_ALERT_2_C Large Average Ueq of Residue Including      C004      0.136 Check
PLAT334_ALERT_2_C Small <C-C> Benzene Dist.   C00F     -C035     .       1.37 Ang.
PLAT334_ALERT_2_C Small <C-C> Benzene Dist.   C022     -C03X     .       1.37 Ang.
PLAT334_ALERT_2_C Small <C-C> Benzene Dist.   C02G     -C03D     .       1.37 Ang.
PLAT340_ALERT_3_C Low Bond Precision on  C-C Bonds ...............    0.00481 Ang.
PLAT906_ALERT_3_C Large K Value in the Analysis of Variance ......    153.853 Check
PLAT906_ALERT_3_C Large K Value in the Analysis of Variance ......     16.261 Check
PLAT906_ALERT_3_C Large K Value in the Analysis of Variance ......     27.722 Check
PLAT906_ALERT_3_C Large K Value in the Analysis of Variance ......      6.240 Check
PLAT906_ALERT_3_C Large K Value in the Analysis of Variance ......      9.965 Check
PLAT906_ALERT_3_C Large K Value in the Analysis of Variance ......      3.859 Check
PLAT906_ALERT_3_C Large K Value in the Analysis of Variance ......      5.223 Check
PLAT906_ALERT_3_C Large K Value in the Analysis of Variance ......      2.288 Check
PLAT906_ALERT_3_C Large K Value in the Analysis of Variance ......      2.520 Check
PLAT911_ALERT_3_C Missing FCF Refl Between Thmin & STh/L=    0.600          2 Report
                1  5  1,   2  0  2,
PLAT992_ALERT_5_C Repd & Actual _reflns_number_gt Values Differ by         14 Check
```

Alert level G

```
ABSMU01_ALERT_1_G  Calculation of _exptl_absorpt_correction_mu
                not performed for this radiation type.
PLAT002_ALERT_2_G Number of Distance or Angle Restraints on AtSite         80 Note
PLAT032_ALERT_4_G Std. Uncertainty on Flack Parameter Value High .      1.000 Report
PLAT176_ALERT_4_G The CIF-Embedded .res File Contains SADI Records         41 Report
PLAT191_ALERT_3_G A Non-default SADI Restraint Value has been used     0.0400 Report
PLAT191_ALERT_3_G A Non-default SADI Restraint Value has been used     0.0400 Report
PLAT191_ALERT_3_G A Non-default SADI Restraint Value has been used     0.0400 Report
PLAT191_ALERT_3_G A Non-default SADI Restraint Value has been used     0.0400 Report
PLAT191_ALERT_3_G A Non-default SADI Restraint Value has been used     0.0400 Report
PLAT191_ALERT_3_G A Non-default SADI Restraint Value has been used     0.0400 Report
PLAT230_ALERT_2_G Hirshfeld Test Diff for    C02Q     --C03V     .        5.8 s.u.
PLAT301_ALERT_3_G Main Residue  Disorder ..............(Resd    1)        29% Note
PLAT301_ALERT_3_G Main Residue  Disorder ..............(Resd    2)        18% Note
PLAT301_ALERT_3_G Main Residue  Disorder ..............(Resd    3)        18% Note
PLAT301_ALERT_3_G Main Residue  Disorder ..............(Resd    4)        18% Note
PLAT301_ALERT_3_G Main Residue  Disorder ..............(Resd    5)        18% Note
PLAT333_ALERT_2_G Large Aver C6-Ring C-C Dist C002     -C00A     .       1.42 Ang.
PLAT335_ALERT_2_G Check Large C6 Ring C-C Range C8       -C042           0.17 Ang.
PLAT335_ALERT_2_G Check Large C6 Ring C-C Range C02Q     -C21            0.22 Ang.
PLAT410_ALERT_2_G Short Intra H...H Contact  H01H     ..H1E      .       2.13 Ang.
                                                      x,y,z  =     1_555 Check
PLAT412_ALERT_2_G Short Intra XH3 .. XHn     H01N     ..H49E     .       2.06 Ang.
                                                      x,y,z  =     1_555 Check
PLAT412_ALERT_2_G Short Intra XH3 .. XHn     H1A      ..H03X     .       2.13 Ang.
                                                      x,y,z  =     1_555 Check
PLAT412_ALERT_2_G Short Intra XH3 .. XHn     H023     ..H04C     .       2.11 Ang.
```

```
                                                        x,y,z  =      1_555 Check
PLAT412_ALERT_2_G Short Intra XH3 .. XHn     H028     ..H2AB     .       1.93 Ang.
                                                        x,y,z  =      1_555 Check
PLAT412_ALERT_2_G Short Intra XH3 .. XHn     H02S     ..H20B     .       1.81 Ang.
                                                        x,y,z  =      1_555 Check
PLAT412_ALERT_2_G Short Intra XH3 .. XHn     H03G     ..H03K     .       2.12 Ang.
                                                        x,y,z  =      1_555 Check
PLAT412_ALERT_2_G Short Intra XH3 .. XHn     H03K     ..H18A     .       2.05 Ang.
                                                        x,y,z  =      1_555 Check
PLAT412_ALERT_2_G Short Intra XH3 .. XHn     H3       ..H13C     .       2.05 Ang.
                                                        x,y,z  =      1_555 Check
PLAT412_ALERT_2_G Short Intra XH3 .. XHn     H4C      ..H02Z     .       2.12 Ang.
                                                        x,y,z  =      1_555 Check
PLAT412_ALERT_2_G Short Intra XH3 .. XHn     H02W     ..H9AC     .       2.08 Ang.
                                                        x,y,z  =      1_555 Check
PLAT412_ALERT_2_G Short Intra XH3 .. XHn     H1       ..H46C     .       2.09 Ang.
                                                        x,y,z  =      1_555 Check
PLAT412_ALERT_2_G Short Intra XH3 .. XHn     H039     ..H22B     .       1.89 Ang.
                                                        x,y,z  =      1_555 Check
PLAT720_ALERT_4_G Number of Unusual/Non-Standard Labels ..........        283 Note
              C001    C002    C003    H003    C004    H004    C005    C006
              C007    C008    C009    C00A    C00B    H00B    C00C    C00D
              H00D    C00E    H00E    C00F    C00G    H00G    C00H    H00H
              C00I    H00I    C00J    H00J    C00K    H00K    C00L    H00L
              C00M    C00N    C00O    H00O    C00P    H00P    C00Q    C00R
              H00R    C00S    C00T    C00U    C00V    C00W    H00W    C00X
              H00X    C00Y    H00Y    C00Z    H00Z    C010    H010    C011
              C012    H012    C013    C014    C015    H015    C016    C017
              H017    C018    C019    H019    C01A    C01B    C01C    H01C
              C01D    C01E    C01F    H01F    C01G    H01G    C01H    H01H
              C01I    H01I    C01J    C01K    H01K    C01L    H01L    C01M
              C01N    H01N    C01O    H01O    C01P    C01Q    H01Q    C01R
              C01S    C01T    H01T    C01U    C01V    H01V    C01W    H01W
              C01X    C01Y    C01Z    C020    H020    C021    C022    C023
              H023    C024    C025    H025    C026    H026    C027    H027
              C028    H028    C029    C02A    H02A    C02B    C02C    H02C
              C02D    C02E    C02F    H02F    C02G    C02H    H02H    C02I
              H02I    C02J    H02J    C02K    H02K    C02L    C02M    H02M
              C02N    H02N    C02O    H02O    C02P    C02Q    C02R    H02R
              C02S    H02S    C02T    H02T    C02U    C02V    C02W    H02W
              C02X    H02X    C02Y    H02Y    C02Z    H02Z    C030    C031
              C032    H032    C033    H033    C034    C035    H035    C036
              H036    C037    C038    H038    C039    H039    C03A    C03B
              H03U    C03C    H03Y    C03D    H03     C03E    C03F    H03A
              H03B    H03C    C03G    H03D    H03E    H03F    C03H    H03H
              C03I    H03G    H03I    H03J    C03J    C03K    H03K    C03L
              C03M    H03X    C03N    C03O    C03P    C03Q    C03R    H03L
              H03M    H03N    C03S    C03T    H03O    H03P    H03Q    C03U
              H03R    H03S    H03T    C03V    H03V    C03W    H03W    C03X
              C03Y    C03Z    H03Z    C040    H04D    H04E    H04F    C041
              H04A    H04B    H04C    C042    H042    C043    H04G    H04H
              H04I    H9AA    H9AB    H9AC    H4AA    H4AB    H4AC    H6AA
              H6AB    H6AC    H7AA    H7AB    H7AC    H1AA    H1AB    H1AC
              H3AA    H3AB    H3AC    H2AA    H2AB    H2AC    C0      H0A
              H0B     H0C     H5AA    H5AB    H5AC    H1BA    H1BB    H1BC
              H1CA    H1CB    H1CC
PLAT721_ALERT_1_G Bond    Calc     0.96000, Rep     0.97210 Dev...       0.01 Ang.
```

```
              C6A      -H6AA          1_555   1_555 ........     #  410 Check
PLAT721_ALERT_1_G Bond    Calc     0.96000, Rep     0.97430 Dev...       0.01 Ang.
              C6A      -H6AB          1_555   1_555 ........     #  411 Check
PLAT721_ALERT_1_G Bond    Calc     0.96000, Rep     0.97450 Dev...       0.01 Ang.
              C6A      -H6AC          1_555   1_555 ........     #  412 Check
PLAT721_ALERT_1_G Bond    Calc     0.95000, Rep     0.96120 Dev...       0.01 Ang.
              C7A      -H7AC          1_555   1_555 ........     #  415 Check
PLAT721_ALERT_1_G Bond    Calc     0.96000, Rep     0.97240 Dev...       0.01 Ang.
              C17      -H17A          1_555   1_555 ........     #  452 Check
PLAT721_ALERT_1_G Bond    Calc     0.96000, Rep     0.97240 Dev...       0.01 Ang.
              C17      -H17B          1_555   1_555 ........     #  453 Check
PLAT721_ALERT_1_G Bond    Calc     0.96000, Rep     0.97200 Dev...       0.01 Ang.
              C17      -H17C          1_555   1_555 ........     #  454 Check
PLAT721_ALERT_1_G Bond    Calc     0.96000, Rep     0.99520 Dev...       0.04 Ang.
              C20      -H20A          1_555   1_555 ........     #  467 Check
PLAT721_ALERT_1_G Bond    Calc     0.95000, Rep     0.99330 Dev...       0.04 Ang.
              C20      -H20B          1_555   1_555 ........     #  468 Check
PLAT721_ALERT_1_G Bond    Calc     0.96000, Rep     0.99540 Dev...       0.04 Ang.
              C20      -H20C          1_555   1_555 ........     #  469 Check
PLAT721_ALERT_1_G Bond    Calc     0.95000, Rep     0.96550 Dev...       0.02 Ang.
              C1C      -H1CC          1_555   1_555 ........     #  475 Check
PLAT722_ALERT_1_G Angle   Calc      110.00, Rep      111.30 Dev...       1.30 Degree
              C03Q    -C6A     -H6AB       1_555  1_555  1_555     #  728 Check
PLAT722_ALERT_1_G Angle   Calc      110.00, Rep      111.50 Dev...       1.50 Degree
              C03Q    -C6A     -H6AC       1_555  1_555  1_555     #  730 Check
PLAT722_ALERT_1_G Angle   Calc      109.00, Rep      107.90 Dev...       1.10 Degree
              H6AA    -C6A     -H6AC       1_555  1_555  1_555     #  731 Check
PLAT722_ALERT_1_G Angle   Calc      109.00, Rep      110.20 Dev...       1.20 Degree
              C02V    -C1A     -H1AC       1_555  1_555  1_555     #  742 Check
PLAT722_ALERT_1_G Angle   Calc      109.00, Rep      110.50 Dev...       1.50 Degree
              C01M    -C14     -H14A       1_555  1_555  1_555     #  805 Check
PLAT722_ALERT_1_G Angle   Calc      110.00, Rep      108.60 Dev...       1.40 Degree
              H14B    -C14     -H14C       1_555  1_555  1_555     #  810 Check
PLAT722_ALERT_1_G Angle   Calc      109.00, Rep      110.80 Dev...       1.80 Degree
              C01M    -C17     -H17A       1_555  1_555  1_555     #  811 Check
PLAT722_ALERT_1_G Angle   Calc      109.00, Rep      110.80 Dev...       1.80 Degree
              C01M    -C17     -H17B       1_555  1_555  1_555     #  812 Check
PLAT722_ALERT_1_G Angle   Calc      110.00, Rep      108.30 Dev...       1.70 Degree
              H17B    -C17     -H17C       1_555  1_555  1_555     #  816 Check
PLAT722_ALERT_1_G Angle   Calc      109.00, Rep      110.40 Dev...       1.40 Degree
              C03Y    -C16A    -H16E       1_555  1_555  1_555     #  824 Check
PLAT722_ALERT_1_G Angle   Calc      109.00, Rep      113.00 Dev...       4.00 Degree
              C037    -C20     -H20A       1_555  1_555  1_555     #  841 Check
PLAT722_ALERT_1_G Angle   Calc      110.00, Rep      112.40 Dev...       2.40 Degree
              C037    -C20     -H20B       1_555  1_555  1_555     #  842 Check
PLAT722_ALERT_1_G Angle   Calc      110.00, Rep      106.10 Dev...       3.90 Degree
              H20A    -C20     -H20B       1_555  1_555  1_555     #  843 Check
PLAT722_ALERT_1_G Angle   Calc      109.00, Rep      113.00 Dev...       4.00 Degree
              C037    -C20     -H20C       1_555  1_555  1_555     #  844 Check
PLAT722_ALERT_1_G Angle   Calc      109.00, Rep      105.90 Dev...       3.10 Degree
              H20A    -C20     -H20C       1_555  1_555  1_555     #  845 Check
PLAT722_ALERT_1_G Angle   Calc      110.00, Rep      105.90 Dev...       4.10 Degree
              H20B    -C20     -H20C       1_555  1_555  1_555     #  846 Check
PLAT722_ALERT_1_G Angle   Calc      109.00, Rep      110.30 Dev...       1.30 Degree
              C037    -C1C     -H1CB       1_555  1_555  1_555     #  854 Check
PLAT722_ALERT_1_G Angle   Calc      110.00, Rep      108.90 Dev...       1.10 Degree
              H1CA    -C1C     -H1CC       1_555  1_555  1_555     #  857 Check
```

```
PLAT722_ALERT_1_G Angle   Calc      110.00, Rep      108.70 Dev...       1.30 Degree
              H45E    -C45A    -H45F       1_555  1_555  1_555     #  876 Check
PLAT790_ALERT_4_G Centre of Gravity not Within Unit-Cell: Resd.  #          4 Note
              C34 H34
PLAT802_ALERT_4_G CIF Input Record(s) with more than 80 Characters          7 Info
PLAT860_ALERT_3_G Number of Least-Squares Restraints ............          42 Note
PLAT883_ALERT_1_G Absent Datum for _atom_sites_solution_primary ..     Please Do !
PLAT899_ALERT_4_G SHELXL2018 is Outdated   and Succeeded by SHELXL     2019/3 Note
PLAT912_ALERT_4_G Missing # of FCF Reflections Above STh/L=  0.600       5171 Note
PLAT913_ALERT_3_G Missing # of Very Strong Reflections in FCF ....          2 Note
                1  5  1,   2  0  2,
PLAT916_ALERT_2_G Hooft y and Flack x Parameter Values Differ by .       0.80 Check
PLAT933_ALERT_2_G Number of HKL-OMIT Records in Embedded .res File          3 Note
                1 -5  1,   1  5  1,   2  0  2,
PLAT969_ALERT_5_G The 'Henn et al.' R-Factor-gap value ..........      2.509 Note
              Predicted wR2: Based on SigI**2 11.11 or SHELX Weight 30.26
PLAT978_ALERT_2_G Number C-C Bonds with Positive Residual Density.          7 Info
```

```
   4 ALERT level A = Most likely a serious problem - resolve or explain
  11 ALERT level B = A potentially serious problem, consider carefully
  79 ALERT level C = Check. Ensure it is not caused by an omission or oversight
  74 ALERT level G = General information/check it is not something unexpected

  36 ALERT type 1 CIF construction/syntax error, inconsistent or missing data
  83 ALERT type 2 Indicator that the structure model may be wrong or deficient
  31 ALERT type 3 Indicator that the structure quality may be low
  16 ALERT type 4 Improvement, methodology, query or suggestion
   2 ALERT type 5 Informative message, check
```

It is advisable to attempt to resolve as many as possible of the alerts in all categories. Often the minor alerts point to easily fixed oversights, errors and omissions in your CIF or refinement strategy, so attention to these fine details can be worthwhile. It is up to the individual to critically assess their own results and, if necessary, seek expert advice.

PLATON version of 26/09/2025; check.def file version of 20/09/2025

Datablock exp_400 - ellipsoid plot

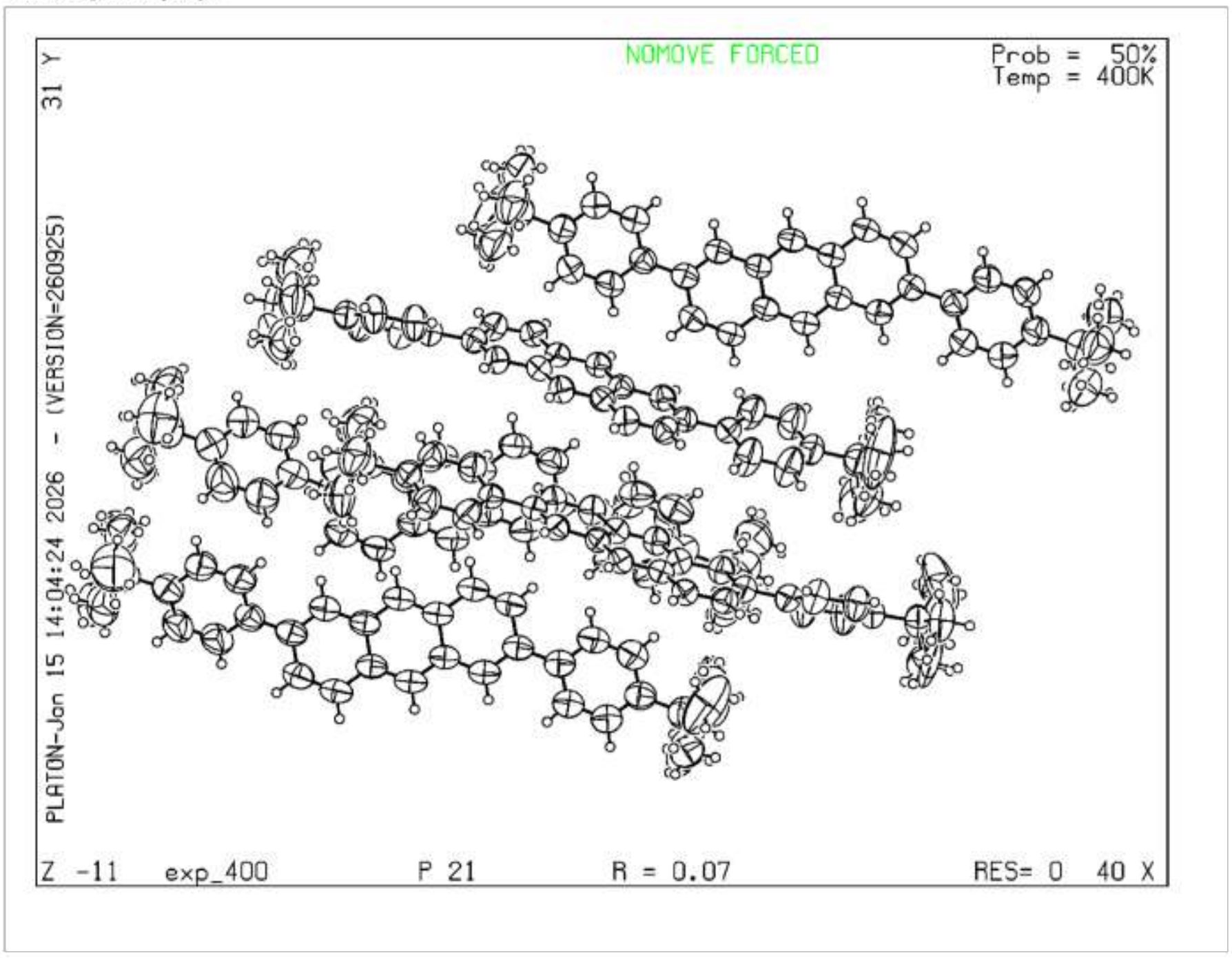

checkCIF/PLATON report

Structure factors have been supplied for datablock(s) exp_423

THIS REPORT IS FOR GUIDANCE ONLY. IF USED AS PART OF A REVIEW PROCEDURE FOR PUBLICATION, IT SHOULD NOT REPLACE THE EXPERTISE OF AN EXPERIENCED CRYSTALLOGRAPHIC REFEREE.

No syntax errors found. CIF dictionary Interpreting this report

Datablock: exp_423

```
Bond precision:   C-C = 0.0044 A          Wavelength=0.41330

Cell:             a=17.7442(7)     b=28.9193(7)       c=13.4932(5)
                  alpha=90         beta=102.709(4)    gamma=90
Temperature:      423 K

                 Calculated                  Reported
Volume           6754.4(4)                   6754.4(4)
Space group      P 21/c                      P 21/c
Hall group       -P 2ybc                     -P 2ybc
Moiety formula   C34 H34                     ?
Sum formula      C34 H34                     C34 H34
Mr               442.61                      442.61
Dx,g cm-3        1.088                       1.088
Z                10                          10
Mu (mm-1)        0.029                       0.029
F000             2380.0                      2380.0
F000'            2379.68
h,k,lmax         31,51,24                    31,51,24
Nref             41077                       35529
Tmin,Tmax
Tmin'

Correction method= Not given
```

Data completeness= 0.865 Theta(max)= 21.808

R(reflections)= 0.1141(7895) wR2(reflections)= 0.4754(35529)

S = 0.931 Npar= 936

The following ALERTS were generated. Each ALERT has the format
test-name_ALERT_alert-type_alert-level.
Click on the hyperlinks for more details of the test.

Alert level A

SHFSU01_ALERT_2_A The absolute value of parameter shift to su ratio > 0.20
Absolute value of the parameter shift to su ratio given 0.396
Additional refinement cycles may be required.

Author Response: This alert arises because the final refinement cycle shows a relativel

PLAT026_ALERT_3_A Ratio Observed / Unique Reflections (too) Low .. 22% Check

Author Response: This alert arises because the ratio of observed to unique reflections

PLAT080_ALERT_2_A Maximum Shift/Error .... 0.40 Why ?

Author Response: This alert arises because the final refinement cycle shows a maximum s

PLAT084_ALERT_3_A High wR2 Value (i.e. > 0.25) .... 0.48 Report

Author Response: This alert arises because the wR2 is relatively high due to the presen

---

Alert level B

PLAT230_ALERT_2_B Hirshfeld Test Diff for C005 --C00D . 8.3 s.u.

Author Response: This alert arises because the Hirshfeld rigid bond test indicates elev

PLAT230_ALERT_2_B Hirshfeld Test Diff for C00J --C00V . 10.0 s.u.

Author Response: This alert arises because the Hirshfeld rigid bond test indicates elev

PLAT230_ALERT_2_B Hirshfeld Test Diff for C00M --C014 . 7.7 s.u.

Author Response: This alert arises because the Hirshfeld rigid bond test indicates elev

PLAT230_ALERT_2_B Hirshfeld Test Diff for   C00Q   --C00S   .   16.3 s.u.

Author Response: This alert arises because the Hirshfeld rigid bond test indicates elev

PLAT230_ALERT_2_B Hirshfeld Test Diff for   C012   --C01D   .   11.3 s.u.

Author Response: This alert arises because the Hirshfeld rigid bond test indicates elev

PLAT230_ALERT_2_B Hirshfeld Test Diff for   C01F   --C01S   .   7.7 s.u.

Author Response: This alert arises because the Hirshfeld rigid bond test indicates elev

PLAT230_ALERT_2_B Hirshfeld Test Diff for   C01M   --C01Y   .   9.2 s.u.

Author Response: This alert arises because the Hirshfeld rigid bond test indicates elev

PLAT230_ALERT_2_B Hirshfeld Test Diff for   C01O   --C01X   .   9.5 s.u.

Author Response: This alert arises because the Hirshfeld rigid bond test indicates elev

PLAT242_ALERT_2_B Low   MainResAtom Ueq as Compared to Neighbours   C01U Check

Author Response: This alert arises because several atoms in the main molecule (C01U, C0

PLAT242_ALERT_2_B Low   MainResAtom Ueq as Compared to Neighbours   C01V Check

Author Response: This alert arises because several atoms in the main molecule (C01U, C0

PLAT242_ALERT_2_B Low   MainResAtom Ueq as Compared to Neighbours   C01P Check

Author Response: This alert arises because several atoms in the main molecule (C01U, C0

PLAT242_ALERT_2_B Low   MainResAtom Ueq as Compared to Neighbours   C01X Check

Author Response: This alert arises because several atoms in the main molecule (C01U, C0

PLAT242_ALERT_2_B Low   MainResAtom Ueq as Compared to Neighbours   C01Y Check

Author Response: This alert arises because several atoms in the main molecule (C01U, C0

```
PLAT910_ALERT_3_B Missing FCF Reflection(s) Below Theta(Min)[Deg]=       1.49 Note
                1  0  0,   2  0  0,   1  1  0,   2  1  0,   0  2  0,   1  2  0,
                1  3  0,  -1  1  1,   0  1  1,   1  1  1,  -1  2  1,   0  2  1,
```

Author Response: This alert arises because some very low-angle reflections below theta

---

Alert level C

```
RINTA01_ALERT_3_C  The value of Rint is greater than 0.12
            Rint given   0.140
PLAT020_ALERT_3_C The Value of Rint is Greater Than 0.12 .........      0.140 Report
PLAT052_ALERT_1_C Info on Absorption Correction Method   Not Given     Please Do !
PLAT053_ALERT_1_C Minimum Crystal Dimension Missing (or Error) ...     Please Check
PLAT054_ALERT_1_C Medium  Crystal Dimension Missing (or Error) ...     Please Check
PLAT055_ALERT_1_C Maximum Crystal Dimension Missing (or Error) ...     Please Check
PLAT082_ALERT_2_C High R1 Value ..................................       0.11 Report
PLAT220_ALERT_2_C NonSolvent   Resd 1  C   Ueq(max)/Ueq(min) Range        3.7 Ratio
PLAT222_ALERT_3_C NonSolvent Resd 1  H   Uiso(max)/Uiso(min) Range        4.3 Ratio
PLAT230_ALERT_2_C Hirshfeld Test Diff for    C01C     --C01T     .        5.2 s.u.
```

Author Response: This alert arises because the Hirshfeld rigid bond test indicates elev

```
PLAT230_ALERT_2_C Hirshfeld Test Diff for    C00J     --C00Z     .        5.3 s.u.
```

Author Response: This alert arises because the Hirshfeld rigid bond test indicates elev

```
PLAT230_ALERT_2_C Hirshfeld Test Diff for    C00V     --C012     .        6.7 s.u.
```

Author Response: This alert arises because the Hirshfeld rigid bond test indicates elev

```
PLAT230_ALERT_2_C Hirshfeld Test Diff for    C013     --C016     .        7.0 s.u.
```

Author Response: This alert arises because the Hirshfeld rigid bond test indicates elev

```
PLAT230_ALERT_2_C Hirshfeld Test Diff for    C013     --C01N     .        6.2 s.u.
```

Author Response: This alert arises because the Hirshfeld rigid bond test indicates elev

```
PLAT230_ALERT_2_C Hirshfeld Test Diff for    C01K     --C01N     .        5.2 s.u.
```

Author Response: This alert arises because the Hirshfeld rigid bond test indicates elev

```
PLAT230_ALERT_2_C Hirshfeld Test Diff for    C01O     --C01W     .        5.6 s.u.
```

Author Response: This alert arises because the Hirshfeld rigid bond test indicates elev

```
PLAT234_ALERT_4_C Large Hirshfeld Difference C39      --C01V     .       0.19 Ang.
PLAT234_ALERT_4_C Large Hirshfeld Difference C01U     --C020     .       0.16 Ang.
PLAT234_ALERT_4_C Large Hirshfeld Difference C01P     --C38A     .       0.22 Ang.
PLAT234_ALERT_4_C Large Hirshfeld Difference C01P     --C43A     .       0.17 Ang.
PLAT241_ALERT_2_C High  MainResAtom  Ueq as Compared to Neighbours       C01E Check
PLAT241_ALERT_2_C High  MainResAtom  Ueq as Compared to Neighbours       C01I Check
PLAT241_ALERT_2_C High  MainResAtom  Ueq as Compared to Neighbours       C01L Check
PLAT241_ALERT_2_C High  MainResAtom  Ueq as Compared to Neighbours       C01T Check
PLAT241_ALERT_2_C High  MainResAtom  Ueq as Compared to Neighbours       C015 Check
PLAT241_ALERT_2_C High  MainResAtom  Ueq as Compared to Neighbours       C01A Check
PLAT241_ALERT_2_C High  MainResAtom  Ueq as Compared to Neighbours       C01G Check
PLAT241_ALERT_2_C High  MainResAtom  Ueq as Compared to Neighbours       C01J Check
PLAT241_ALERT_2_C High  MainResAtom  Ueq as Compared to Neighbours       C019 Check
PLAT241_ALERT_2_C High  MainResAtom  Ueq as Compared to Neighbours       C01Q Check
PLAT241_ALERT_2_C High  MainResAtom  Ueq as Compared to Neighbours       C01W Check
PLAT242_ALERT_2_C Low   MainResAtom  Ueq as Compared to Neighbours       C00R Check
```

Author Response: This alert arises because several atoms in the main molecule (C01U, C0

```
PLAT242_ALERT_2_C Low   MainResAtom  Ueq as Compared to Neighbours       C00W Check
```

Author Response: This alert arises because several atoms in the main molecule (C01U, C0

```
PLAT242_ALERT_2_C Low   MainResAtom  Ueq as Compared to Neighbours       C00X Check
```

Author Response: This alert arises because several atoms in the main molecule (C01U, C0

```
PLAT242_ALERT_2_C Low   MainResAtom  Ueq as Compared to Neighbours       C01C Check
```

Author Response: This alert arises because several atoms in the main molecule (C01U, C0

```
PLAT242_ALERT_2_C Low   MainResAtom  Ueq as Compared to Neighbours       C00N Check
```

Author Response: This alert arises because several atoms in the main molecule (C01U, C0

```
PLAT242_ALERT_2_C Low   MainResAtom  Ueq as Compared to Neighbours       C011 Check
```

Author Response: This alert arises because several atoms in the main molecule (C01U, C0

```
PLAT242_ALERT_2_C Low   MainResAtom  Ueq as Compared to Neighbours       C013 Check
```

Author Response: This alert arises because several atoms in the main molecule (C01U, C0

```
PLAT242_ALERT_2_C Low   MainResAtom  Ueq as Compared to Neighbours       C01D Check
```

Author Response: This alert arises because several atoms in the main molecule (C01U, C0

```
PLAT242_ALERT_2_C Low   MainResAtom  Ueq as Compared to Neighbours       C01M Check
```

Author Response: This alert arises because several atoms in the main molecule (C01U, C0

```
PLAT242_ALERT_2_C Low   MainResAtom  Ueq as Compared to Neighbours       C01O Check
```

Author Response: This alert arises because several atoms in the main molecule (C01U, C0

```
PLAT260_ALERT_2_C Large Average Ueq of Residue Including      C001      0.160 Check
PLAT260_ALERT_2_C Large Average Ueq of Residue Including      C002      0.145 Check
PLAT260_ALERT_2_C Large Average Ueq of Residue Including      C00B      0.199 Check
PLAT334_ALERT_2_C Small <C-C> Benzene Dist.   C00R     -C01L     .       1.37 Ang.
PLAT340_ALERT_3_C Low Bond Precision on  C-C Bonds ...............    0.00442 Ang.
PLAT906_ALERT_3_C Large K Value in the Analysis of Variance ......    456.706 Check
PLAT906_ALERT_3_C Large K Value in the Analysis of Variance ......    106.576 Check
PLAT906_ALERT_3_C Large K Value in the Analysis of Variance ......     41.362 Check
PLAT906_ALERT_3_C Large K Value in the Analysis of Variance ......     72.127 Check
PLAT906_ALERT_3_C Large K Value in the Analysis of Variance ......     16.529 Check
PLAT906_ALERT_3_C Large K Value in the Analysis of Variance ......     30.273 Check
PLAT906_ALERT_3_C Large K Value in the Analysis of Variance ......      6.015 Check
PLAT906_ALERT_3_C Large K Value in the Analysis of Variance ......     11.039 Check
PLAT906_ALERT_3_C Large K Value in the Analysis of Variance ......      2.786 Check
PLAT906_ALERT_3_C Large K Value in the Analysis of Variance ......      3.651 Check
PLAT911_ALERT_3_C Missing FCF Refl Between Thmin & STh/L=    0.600          2 Report
                1  5  1,   2  0  2,
```

Alert level G

```
ABSMU01_ALERT_1_G  Calculation of _exptl_absorpt_correction_mu
                not performed for this radiation type.
PLAT002_ALERT_2_G Number of Distance or Angle Restraints on AtSite        35 Note
PLAT176_ALERT_4_G The CIF-Embedded .res File Contains SADI Records        15 Report
PLAT301_ALERT_3_G Main Residue  Disorder ..............(Resd    1)       18% Note
PLAT301_ALERT_3_G Main Residue  Disorder ..............(Resd    2)       18% Note
PLAT301_ALERT_3_G Main Residue  Disorder ..............(Resd    3)       18% Note
PLAT412_ALERT_2_G Short Intra XH3 .. XHn     H19A     ..H01T     .       2.03 Ang.
                                                      x,y,z  =      1_555 Check
PLAT412_ALERT_2_G Short Intra XH3 .. XHn     H018     ..H68B     .       2.07 Ang.
                                                      x,y,z  =      1_555 Check
PLAT412_ALERT_2_G Short Intra XH3 .. XHn     H01B     ..H50D     .       2.14 Ang.
                                                      x,y,z  =      1_555 Check
PLAT412_ALERT_2_G Short Intra XH3 .. XHn     H01I     ..H02C     .       1.91 Ang.
                                                      x,y,z  =      1_555 Check
PLAT412_ALERT_2_G Short Intra XH3 .. XHn     H01J     ..H38F     .       2.11 Ang.
                                                      x,y,z  =      1_555 Check
PLAT412_ALERT_2_G Short Intra XH3 .. XHn     H01H     ..H53F     .       2.14 Ang.
                                                      x,y,z  =      1_555 Check
PLAT412_ALERT_2_G Short Intra XH3 .. XHn     H01K     ..H67C     .       1.90 Ang.
                                                      x,y,z  =      1_555 Check
PLAT412_ALERT_2_G Short Intra XH3 .. XHn     H01S     ..H40A     .       2.08 Ang.
                                                      x,y,z  =      1_555 Check
PLAT412_ALERT_2_G Short Intra XH3 .. XHn     H01W     ..H22F     .       1.95 Ang.
                                                      x,y,z  =      1_555 Check
PLAT720_ALERT_4_G Number of Unusual/Non-Standard Labels ..........       122 Note
```

```
              C001    C002    C003    C004    C005    C006    C007    H007
              C008    H008    C009    H009    C00A    H00A    C00B    C00C
              H00C    C00D    H00D    C00E    H00E    C00F    C00G    H00G
              C00H    H00H    C00I    C00J    C00K    H00K    C00L    H00L
              C00M    H00M    C00N    C00O    C00P    H00P    C00Q    C00R
              C00S    H00S    C00T    C00U    H00U    C00V    H00V    C00W
              C00X    C00Y    H00Y    C00Z    H00Z    C010    H010    C011
              C012    C013    C014    H014    C015    H015    C016    C017
              H017    C018    H018    C019    H019    C01A    H01G    C01B
              H01B    C01C    C01D    C01E    H01E    C01F    H01F    C01G
              H01J    C01H    H01H    C01I    H01I    C01J    H01M    C01K
              H01K    C01L    H01L    C01M    C01N    H01N    C01O    C01P
              C01Q    H01Q    C01R    H01R    C01S    H01S    C01T    H01T
              C01U    C01V    C01W    H01W    C01X    C01Y    C01Z    H01A
              H01C    H01D    C020    H02A    H02B    H02C    C0      H0A
              H0B     H0C
PLAT721_ALERT_1_G Bond    Calc     0.95000, Rep     0.96010 Dev...       0.01 Ang.
              C0      -H0B           1_555   1_555 ........     #  206 Check
PLAT802_ALERT_4_G CIF Input Record(s) with more than 80 Characters         7 Info
PLAT860_ALERT_3_G Number of Least-Squares Restraints ............         15 Note
PLAT883_ALERT_1_G Absent Datum for _atom_sites_solution_primary ..     Please Do !
PLAT899_ALERT_4_G SHELXL2018 is Outdated   and Succeeded by SHELXL     2019/3 Note
PLAT912_ALERT_4_G Missing # of FCF Reflections Above STh/L=  0.600      5534 Note
PLAT913_ALERT_3_G Missing # of Very Strong Reflections in FCF ....         2 Note
                1  5  1,   2  0  2,
PLAT933_ALERT_2_G Number of HKL-OMIT Records in Embedded .res File         2 Note
                1  5  1,   2  0  2,
PLAT969_ALERT_5_G The 'Henn et al.' R-Factor-gap value ..........      2.601 Note
              Predicted wR2: Based on SigI**2 18.28 or SHELX Weight 51.07
PLAT978_ALERT_2_G Number C-C Bonds with Positive Residual Density.         0 Info
PLAT992_ALERT_5_G Repd & Actual _reflns_number_gt Values Differ by         5 Check
PLAT994_ALERT_1_G SHELXL .ins Contains no or MERG 0 Instruction ..         ! Note
```

```
   4 ALERT level A = Most likely a serious problem - resolve or explain
  14 ALERT level B = A potentially serious problem, consider carefully
  57 ALERT level C = Check. Ensure it is not caused by an omission or oversight
  28 ALERT level G = General information/check it is not something unexpected

   8 ALERT type 1 CIF construction/syntax error, inconsistent or missing data
  61 ALERT type 2 Indicator that the structure model may be wrong or deficient
  23 ALERT type 3 Indicator that the structure quality may be low
   9 ALERT type 4 Improvement, methodology, query or suggestion
   2 ALERT type 5 Informative message, check
```

It is advisable to attempt to resolve as many as possible of the alerts in all categories. Often the minor alerts point to easily fixed oversights, errors and omissions in your CIF or refinement strategy, so attention to these fine details can be worthwhile. It is up to the individual to critically assess their own results and, if necessary, seek expert advice.

PLATON version of 15/01/2026; check.def file version of 02/01/2026

Datablock exp_423 - ellipsoid plot

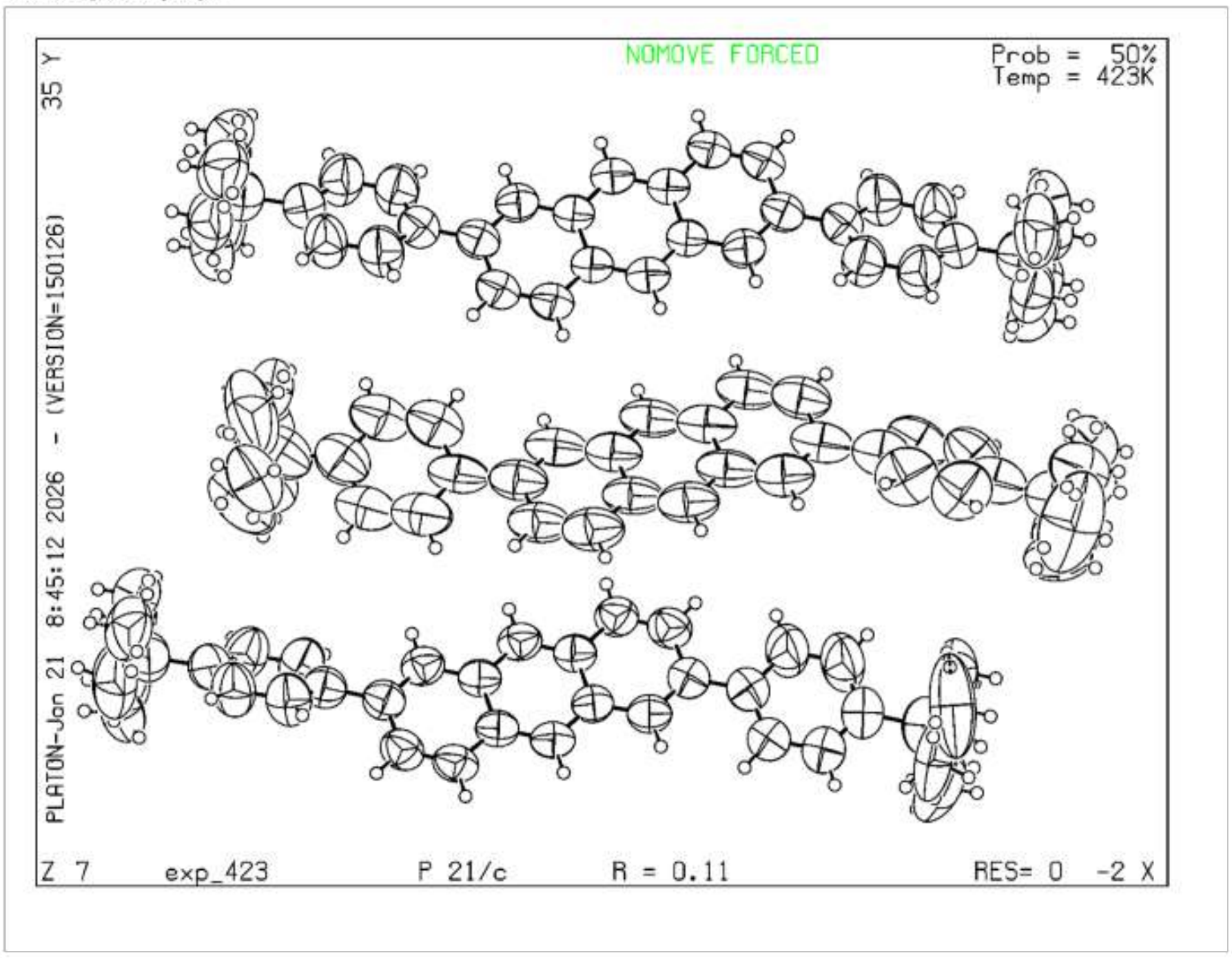

checkCIF/PLATON report

Structure factors have been supplied for datablock(s) exp_799

THIS REPORT IS FOR GUIDANCE ONLY. IF USED AS PART OF A REVIEW PROCEDURE FOR PUBLICATION, IT SHOULD NOT REPLACE THE EXPERTISE OF AN EXPERIENCED CRYSTALLOGRAPHIC REFEREE.

No syntax errors found.   CIF dictionary   Interpreting this report

Datablock: exp_799

```
Bond precision:   C-C = 0.0023 A           Wavelength=0.71073

Cell:             a=11.8442(2)      b=11.9402(2)      c=33.2880(5)
                  alpha=91.155(1)   beta=95.095(1)    gamma=119.725(2)
Temperature:      103 K

                   Calculated                  Reported
Volume             4060.82(14)                 4060.82(13)
Space group        P -1                        P -1
Hall group         -P 1                        -P 1
Moiety formula     C26 H26                     ?
Sum formula        C26 H26                     C26 H26
Mr                 338.47                      338.47
Dx,g cm-3          1.107                       1.107
Z                  8                           8
Mu (mm-1)          0.062                       0.062
F000               1456.0                      1456.0
F000'              1456.51
h,k,lmax           16,16,47                    16,16,45
Nref               24030                       19849
Tmin,Tmax
Tmin'

Correction method= Not given
```

Data completeness= 0.826 Theta(max)= 30.158

R(reflections)= 0.0554(11116) wR2(reflections)= 0.1839(19849)

S = 1.050 Npar= 961

The following ALERTS were generated. Each ALERT has the format
test-name_ALERT_alert-type_alert-level.
Click on the hyperlinks for more details of the test.

Alert level B

```
PLAT097_ALERT_2_B Large Reported Max.  (Positive) Residual Density       0.68 eA-3
```

Author Response: This is due to residual electron density located in the vicinity of ca

---

Alert level C

```
DIFMX02_ALERT_1_C  The maximum difference density is > 0.1*ZMAX*0.75
            The relevant atom site should be identified.
PLAT052_ALERT_1_C Info on Absorption Correction Method   Not Given     Please Do !
PLAT053_ALERT_1_C Minimum Crystal Dimension Missing (or Error) ...     Please Check
PLAT054_ALERT_1_C Medium  Crystal Dimension Missing (or Error) ...     Please Check
PLAT055_ALERT_1_C Maximum Crystal Dimension Missing (or Error) ...     Please Check
PLAT094_ALERT_2_C Ratio of Maximum / Minimum Residual Density ....       2.93 Report
PLAT790_ALERT_4_C Centre of Gravity not Within Unit-Cell: Resd.  #          1 Note
              C26 H26
PLAT905_ALERT_3_C Negative K value in the Analysis of Variance ...     -3.085 Report
```

Alert level G

```
PLAT371_ALERT_2_G Long   C(sp2)-C(sp1) Bond  C00E     - C00X     .       1.44 Ang.
PLAT371_ALERT_2_G Long   C(sp2)-C(sp1) Bond  C011     - C01Q     .       1.43 Ang.
PLAT371_ALERT_2_G Long   C(sp2)-C(sp1) Bond  C00D     - C01V     .       1.44 Ang.
PLAT371_ALERT_2_G Long   C(sp2)-C(sp1) Bond  C00Z     - C01E     .       1.43 Ang.
PLAT371_ALERT_2_G Long   C(sp2)-C(sp1) Bond  C017     - C018     .       1.44 Ang.
PLAT371_ALERT_2_G Long   C(sp2)-C(sp1) Bond  C00U     - C01P     .       1.43 Ang.
PLAT371_ALERT_2_G Long   C(sp2)-C(sp1) Bond  C01B     - C01O     .       1.44 Ang.
PLAT371_ALERT_2_G Long   C(sp2)-C(sp1) Bond  C00W     - C013     .       1.43 Ang.
PLAT720_ALERT_4_G Number of Unusual/Non-Standard Labels .........        189 Note
              C001    C002    C003    C004    C005    H005    C006    C007
              C008    C009    H009    C00A    C00B    H00B    C00C    C00D
              C00E    C00F    C00G    H00G    C00H    H00H    C00I    C00J
              C00K    H00K    C00L    C00M    C00N    C00O    H00O    C00P
              H00P    C00Q    H00Q    C00R    H00R    C00S    H00S    C00T
              H00T    C00U    C00V    H00V    C00W    C00X    C00Y    C00Z
              C010    H010    C011    C012    H012    C013    C014    H014
              C015    H015    C016    H016    C017    C018    C019    H019
              C01A    H01A    C01B    C01C    H01C    C01D    C01E    C01F
              C01G    H01G    C01H    H01H    C01I    H01I    C01J    C01K
              H01K    C01L    C01M    H01M    C01N    H01N    C01O    C01P
              C01Q    C01R    C01S    C01T    H01T    C01U    H01U    C01V
```

```
               C01W    H01W    C01X    H01X    C01Y    H01Y    C01Z    C020
               C021    C022    C023    C024    C025    C026    C027    C028
               C029    H02S    H02T    H02U    C02A    Ha      C02B    H02A
               H02B    H02C    C02C    H02V    H02W    H02X    C02D    Hb
               Hc      C02E    Hd      He      C02F    Hf      Hg      C02G
               Hh      Hi      C02H    H02D    H02E    H02F    C02I    H02G
               H02H    H02I    C02J    H02J    H02K    H02L    C02K    Hj
               Hk      C02L    H02Y    Hl      Hm      C02M    Hn      Ho
               C02N    H02Z    Hp      Hq      C02O    Hr      Hs      C02P
               H02     Ht      Hu      C02Q    H02M    H02N    H02O    C02R
               Hv      Hw      C02S    H02P    H02Q    H02R    C02T    Hx
               Hy      C02U    Hz      C02V    C02W
PLAT790_ALERT_4_G Centre of Gravity not Within Unit-Cell: Resd.  #          2 Note
               C26 H26
PLAT790_ALERT_4_G Centre of Gravity not Within Unit-Cell: Resd.  #          3 Note
               C26 H26
PLAT802_ALERT_4_G CIF Input Record(s) with more than 80 Characters          1 Info
PLAT883_ALERT_1_G Absent Datum for _atom_sites_solution_primary ..     Please Do !
PLAT899_ALERT_4_G SHELXL2018 is Outdated   and Succeeded by SHELXL     2019/3 Note
PLAT908_ALERT_2_G Max. Perc. Data with I > 2*s(I) per Res.Shell ..     65.47% Note
PLAT910_ALERT_3_G Missing FCF Reflection(s) Below Theta(Min)[Deg]=       1.85 Note
                 0  0  1,   0  0  2,
PLAT912_ALERT_4_G Missing # of FCF Reflections Above STh/L=  0.600       4142 Note
PLAT933_ALERT_2_G Number of HKL-OMIT Records in Embedded .res File          1 Note
                 0  0 27,
PLAT941_ALERT_3_G Average HKL Measurement Multiplicity ...........        2.9 Low
PLAT952_ALERT_5_G Calculated (ThMax) and CIF-Reported Lmax  Differ          2 Units
PLAT958_ALERT_1_G Calculated (ThMax) and Actual (FCF) Lmax  Differ          2 Units
PLAT969_ALERT_5_G The 'Henn et al.' R-Factor-gap value ...........      4.272 Note
               Predicted wR2: Based on SigI**2  4.31 or SHELX Weight 17.50 Note
PLAT978_ALERT_2_G Number C-C Bonds with Positive Residual Density.          6 Info
```

```
   0 ALERT level A = Most likely a serious problem - resolve or explain
   1 ALERT level B = A potentially serious problem, consider carefully
   8 ALERT level C = Check. Ensure it is not caused by an omission or oversight
  23 ALERT level G = General information/check it is not something unexpected

   7 ALERT type 1 CIF construction/syntax error, inconsistent or missing data
  13 ALERT type 2 Indicator that the structure model may be wrong or deficient
   3 ALERT type 3 Indicator that the structure quality may be low
   7 ALERT type 4 Improvement, methodology, query or suggestion
   2 ALERT type 5 Informative message, check
```

It is advisable to attempt to resolve as many as possible of the alerts in all categories. Often the minor alerts point to easily fixed oversights, errors and omissions in your CIF or refinement strategy, so attention to these fine details can be worthwhile. It is up to the individual to critically assess their own results and, if necessary, seek expert advice.

PLATON version of 23/04/2026; check.def file version of 30/03/2026

---

duplicate check

No duplication found

Datablock exp_799 - ellipsoid plot

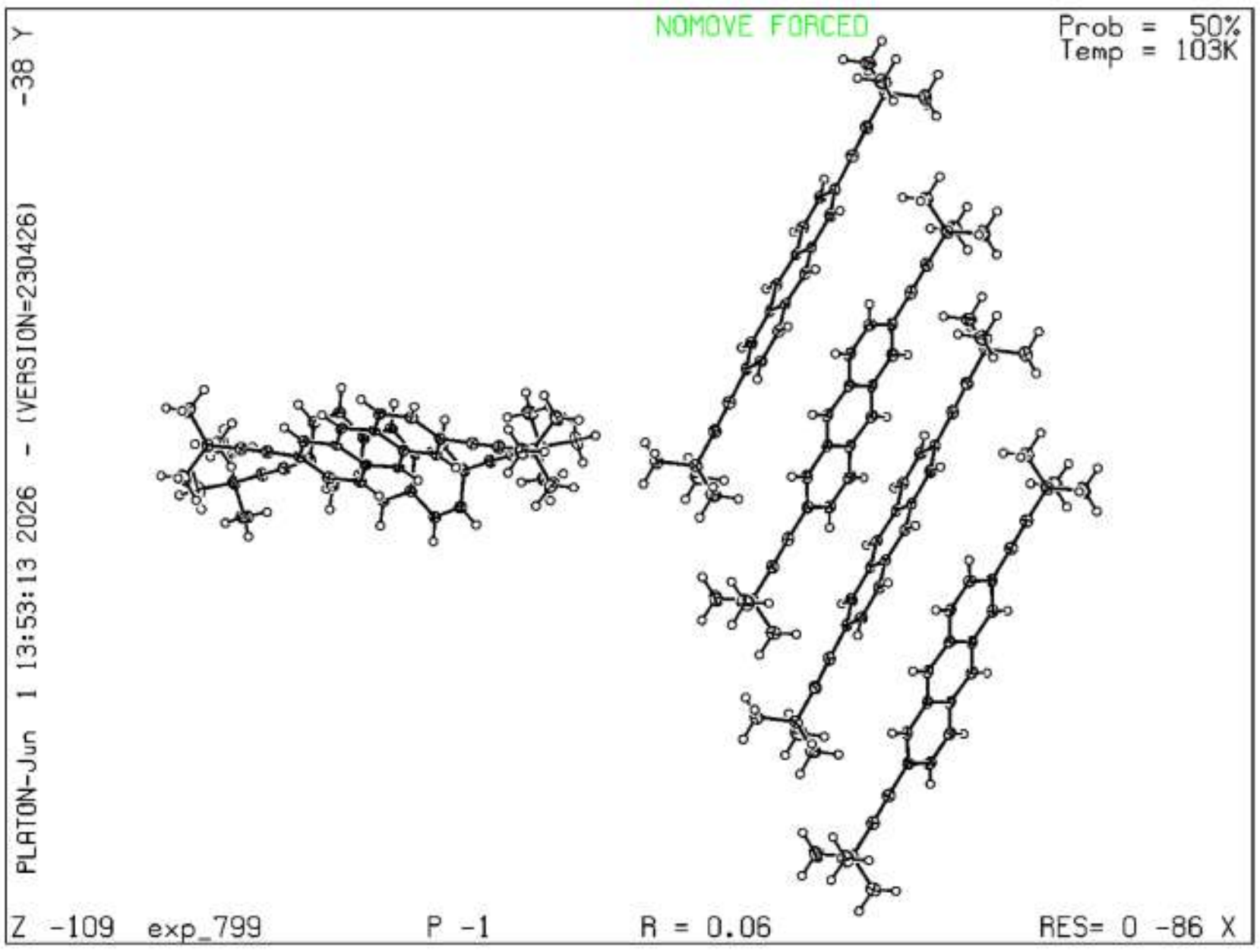

checkCIF/PLATON report

Structure factors have been supplied for datablock(s) exp_804

THIS REPORT IS FOR GUIDANCE ONLY. IF USED AS PART OF A REVIEW PROCEDURE FOR PUBLICATION, IT SHOULD NOT REPLACE THE EXPERTISE OF AN EXPERIENCED CRYSTALLOGRAPHIC REFEREE.

No syntax errors found. CIF dictionary Interpreting this report

Datablock: exp_804

```
Bond precision:   C-C = 0.0028 A           Wavelength=0.71073

Cell:            a=11.9558(3)      b=12.0778(3)      c=33.7088(5)
                 alpha=83.349(2)   beta=84.934(2)    gamma=60.376(2)
Temperature:     303 K

                  Calculated                   Reported
Volume            4200.10(18)                  4200.10(18)
Space group       P -1                         P -1
Hall group        -P 1                         -P 1
Moiety formula    C26 H26                      ?
Sum formula       C26 H26                      C26 H26
Mr                338.47                       338.47
Dx,g cm-3         1.071                        1.071
Z                 8                            8
Mu (mm-1)         0.060                        0.060
F000              1456.0                       1456.0
F000'             1456.51
h,k,lmax          16,17,47                     16,17,46
Nref              24910                        20459
Tmin,Tmax
Tmin'

Correction method= Not given
```

Data completeness= 0.821 Theta(max)= 30.204

R(reflections)= 0.0627(9741) wR2(reflections)= 0.2093(20459)

S = 1.036 Npar= 961

The following ALERTS were generated. Each ALERT has the format
test-name_ALERT_alert-type_alert-level.
Click on the hyperlinks for more details of the test.

Alert level C

```
PLAT026_ALERT_3_C Ratio Observed / Unique Reflections (too) Low ..       48% Check
PLAT052_ALERT_1_C Info on Absorption Correction Method   Not Given     Please Do !
PLAT053_ALERT_1_C Minimum Crystal Dimension Missing (or Error) ...     Please Check
PLAT054_ALERT_1_C Medium  Crystal Dimension Missing (or Error) ...     Please Check
PLAT055_ALERT_1_C Maximum Crystal Dimension Missing (or Error) ...     Please Check
PLAT094_ALERT_2_C Ratio of Maximum / Minimum Residual Density ....       2.44 Report
PLAT242_ALERT_2_C Low   MainResAtom  Ueq as Compared to Neighbours        C2T Check
PLAT242_ALERT_2_C Low   MainResAtom  Ueq as Compared to Neighbours        C2U Check
PLAT242_ALERT_2_C Low   MainResAtom  Ueq as Compared to Neighbours        C2V Check
PLAT242_ALERT_2_C Low   MainResAtom  Ueq as Compared to Neighbours        C2W Check
PLAT242_ALERT_2_C Low   MainResAtom  Ueq as Compared to Neighbours        C2Y Check
PLAT242_ALERT_2_C Low   MainResAtom  Ueq as Compared to Neighbours        C2R Check
PLAT242_ALERT_2_C Low   MainResAtom  Ueq as Compared to Neighbours        C2S Check
PLAT242_ALERT_2_C Low   MainResAtom  Ueq as Compared to Neighbours        C2X Check
PLAT906_ALERT_3_C Large K Value in the Analysis of Variance ......     67.957 Check
PLAT906_ALERT_3_C Large K Value in the Analysis of Variance ......     11.454 Check
PLAT906_ALERT_3_C Large K Value in the Analysis of Variance ......      4.428 Check
```

Alert level G

```
PLAT154_ALERT_1_G The s.u.'s on the Cell Angles are Equal ..(Note)      0.002 Degree
PLAT230_ALERT_2_G Hirshfeld Test Diff for    C1R      --C2J      .        5.8 s.u.
PLAT230_ALERT_2_G Hirshfeld Test Diff for    C1Y      --C2P      .        5.2 s.u.
PLAT371_ALERT_2_G Long   C(sp2)-C(sp1) Bond  C1M      - C25      .       1.43 Ang.
PLAT371_ALERT_2_G Long   C(sp2)-C(sp1) Bond  C1R      - C2J      .       1.44 Ang.
PLAT371_ALERT_2_G Long   C(sp2)-C(sp1) Bond  C2M      - C23      .       1.43 Ang.
PLAT371_ALERT_2_G Long   C(sp2)-C(sp1) Bond  C2C      - C20      .       1.44 Ang.
PLAT371_ALERT_2_G Long   C(sp2)-C(sp1) Bond  C2E      - C22      .       1.44 Ang.
PLAT371_ALERT_2_G Long   C(sp2)-C(sp1) Bond  C1W      - C2A      .       1.43 Ang.
PLAT371_ALERT_2_G Long   C(sp2)-C(sp1) Bond  C1Y      - C2P      .       1.43 Ang.
PLAT371_ALERT_2_G Long   C(sp2)-C(sp1) Bond  C2D      - C26      .       1.44 Ang.
PLAT720_ALERT_4_G Number of Unusual/Non-Standard Labels ..........         42 Note
              H2ZA    H2ZB    H2ZC    H3AA    H3AB    H3AC    H3BA    H3BB
              H3BC    H3CA    H3CB    H3CC    H3DA    H3DB    H3DC    H3EA
              H3EB    H3EC    H3FA    H3FB    H3FC    H3GA    H3GB    H3GC
              H3HA    H3HB    H3HC    H3IA    H3IB    H3IC    H3JA    H3JB
              H3JC    H3KA    H3KB    H3KC    H3LA    H3LB    H3LC    H3MA
              H3MB    H3MC
PLAT790_ALERT_4_G Centre of Gravity not Within Unit-Cell: Resd.  #          5 Note
              C26 H26
PLAT883_ALERT_1_G Absent Datum for _atom_sites_solution_primary ..     Please Do !
PLAT899_ALERT_4_G SHELXL2018 is Outdated   and Succeeded by SHELXL     2019/3 Note
```

```
PLAT908_ALERT_2_G Max. Perc. Data with I > 2*s(I) per Res.Shell ..     64.71% Note
PLAT910_ALERT_3_G Missing FCF Reflection(s) Below Theta(Min)[Deg]=       1.83 Note
                0  0  1,   0  0  2,
PLAT912_ALERT_4_G Missing # of FCF Reflections Above STh/L=  0.600       4424 Note
PLAT941_ALERT_3_G Average HKL Measurement Multiplicity ..........         2.8 Low
PLAT969_ALERT_5_G The 'Henn et al.' R-Factor-gap value ...........      4.635 Note
              Predicted wR2: Based on SigI**2  4.52 or SHELX Weight 20.21 Note
PLAT978_ALERT_2_G Number C-C Bonds with Positive Residual Density.          8 Info
PLAT992_ALERT_5_G Repd & Actual _reflns_number_gt Values Differ by          2 Check
```

```
   0 ALERT level A = Most likely a serious problem - resolve or explain
   0 ALERT level B = A potentially serious problem, consider carefully
  17 ALERT level C = Check. Ensure it is not caused by an omission or oversight
  22 ALERT level G = General information/check it is not something unexpected

   6 ALERT type 1 CIF construction/syntax error, inconsistent or missing data
  21 ALERT type 2 Indicator that the structure model may be wrong or deficient
   6 ALERT type 3 Indicator that the structure quality may be low
   4 ALERT type 4 Improvement, methodology, query or suggestion
   2 ALERT type 5 Informative message, check
```

It is advisable to attempt to resolve as many as possible of the alerts in all categories. Often the minor alerts point to easily fixed oversights, errors and omissions in your CIF or refinement strategy, so attention to these fine details can be worthwhile. It is up to the individual to critically assess their own results and, if necessary, seek expert advice.

PLATON version of 23/04/2026; check.def file version of 30/03/2026

---

duplicate check

No duplication found

Datablock exp_804 - ellipsoid plot

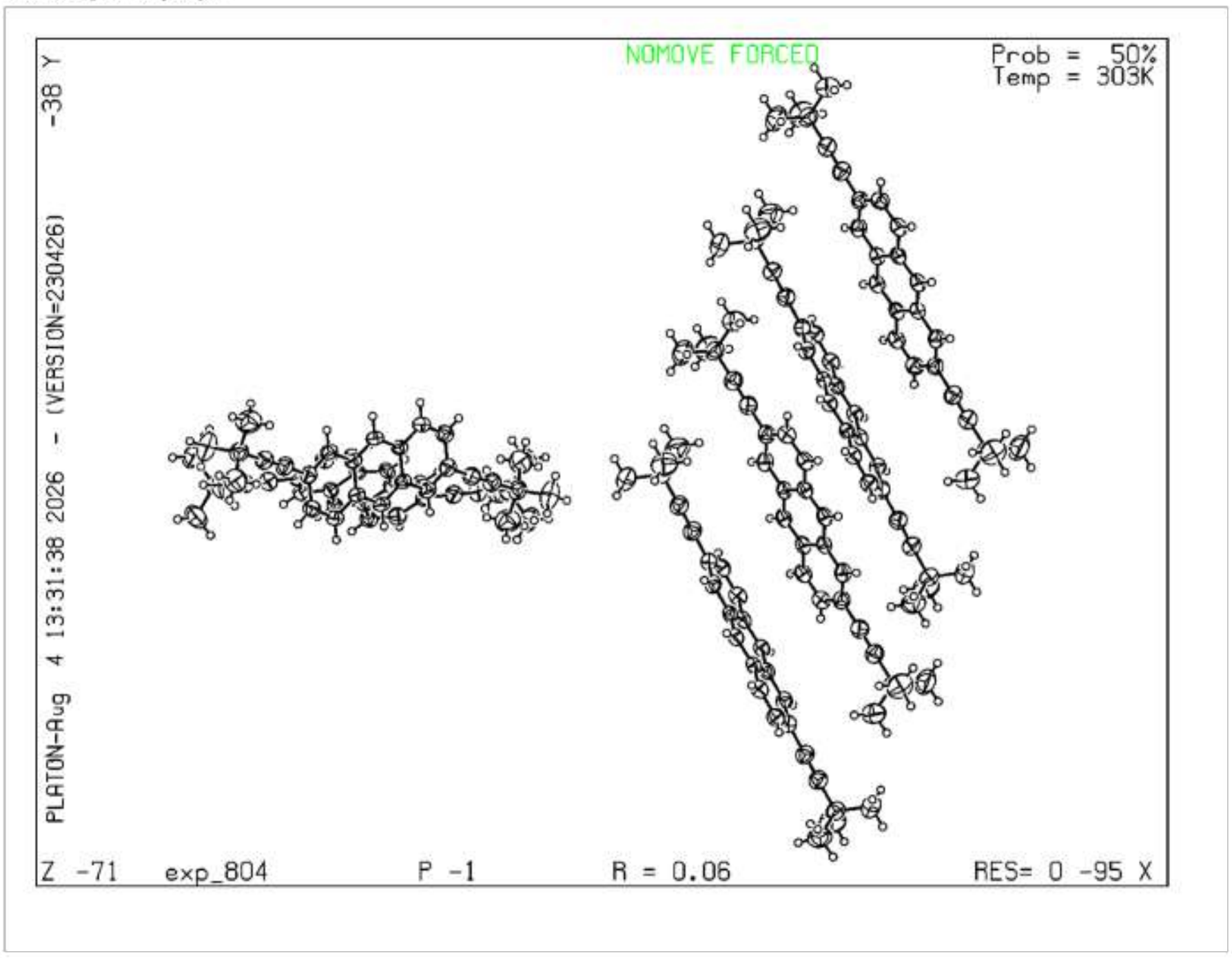

PLATON/ADDSYM

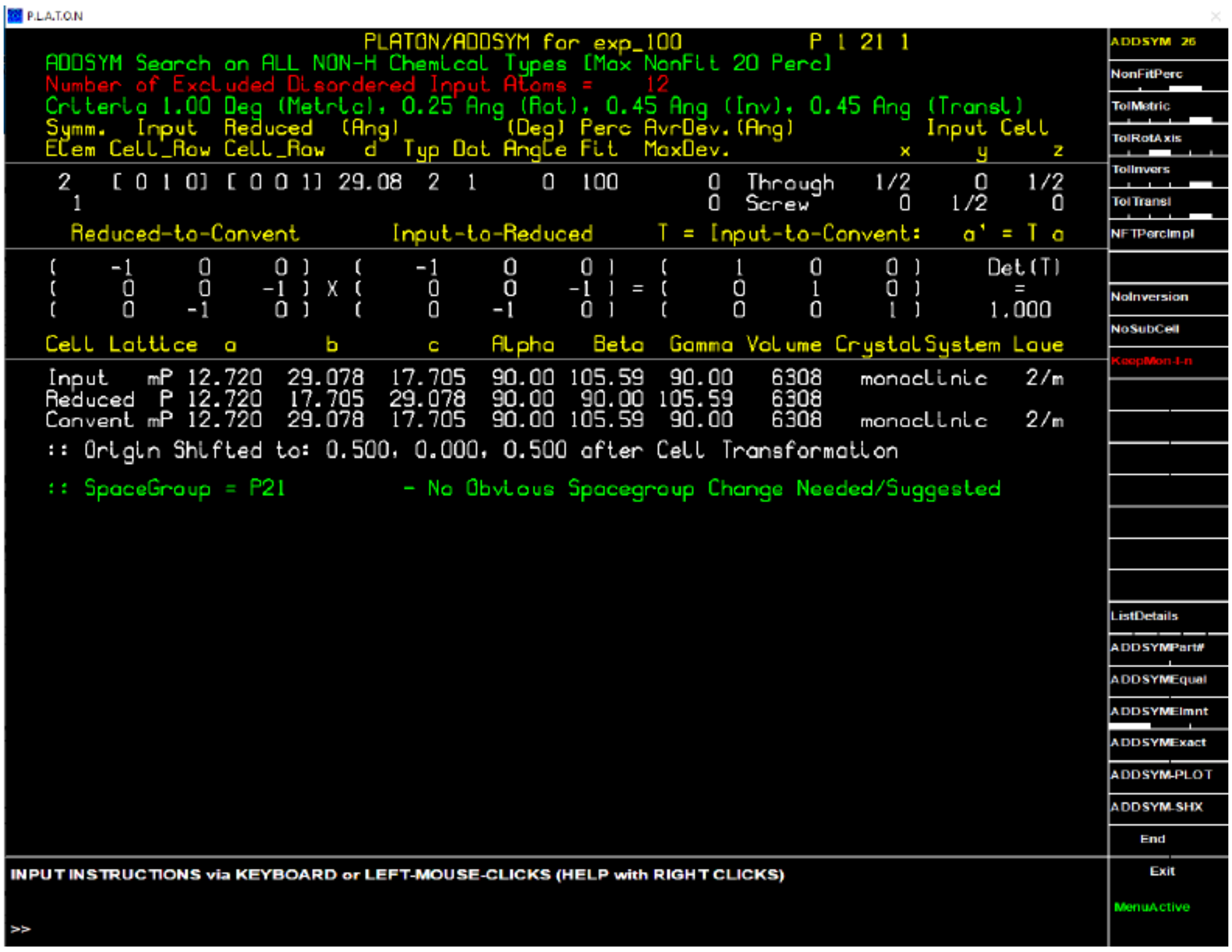

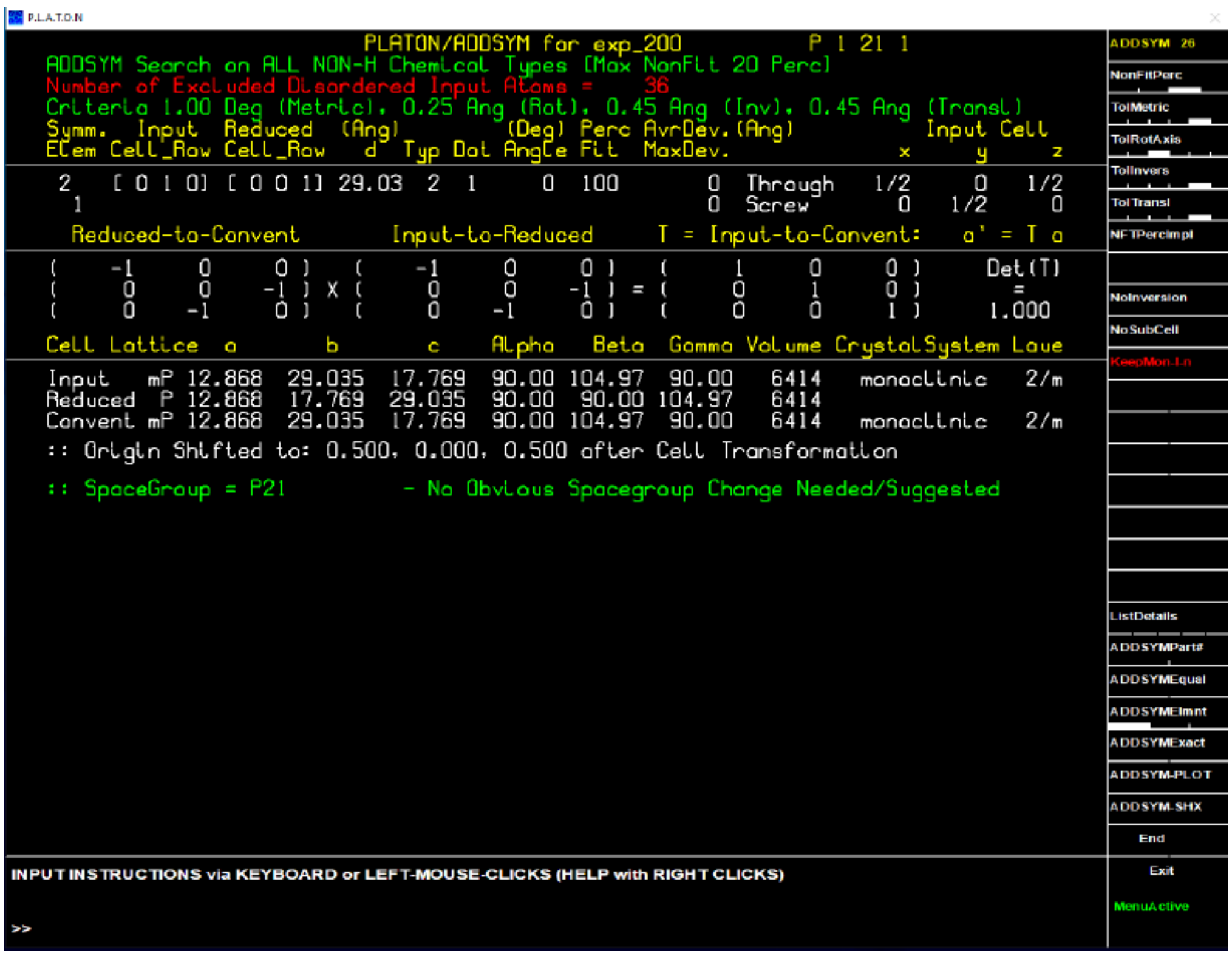

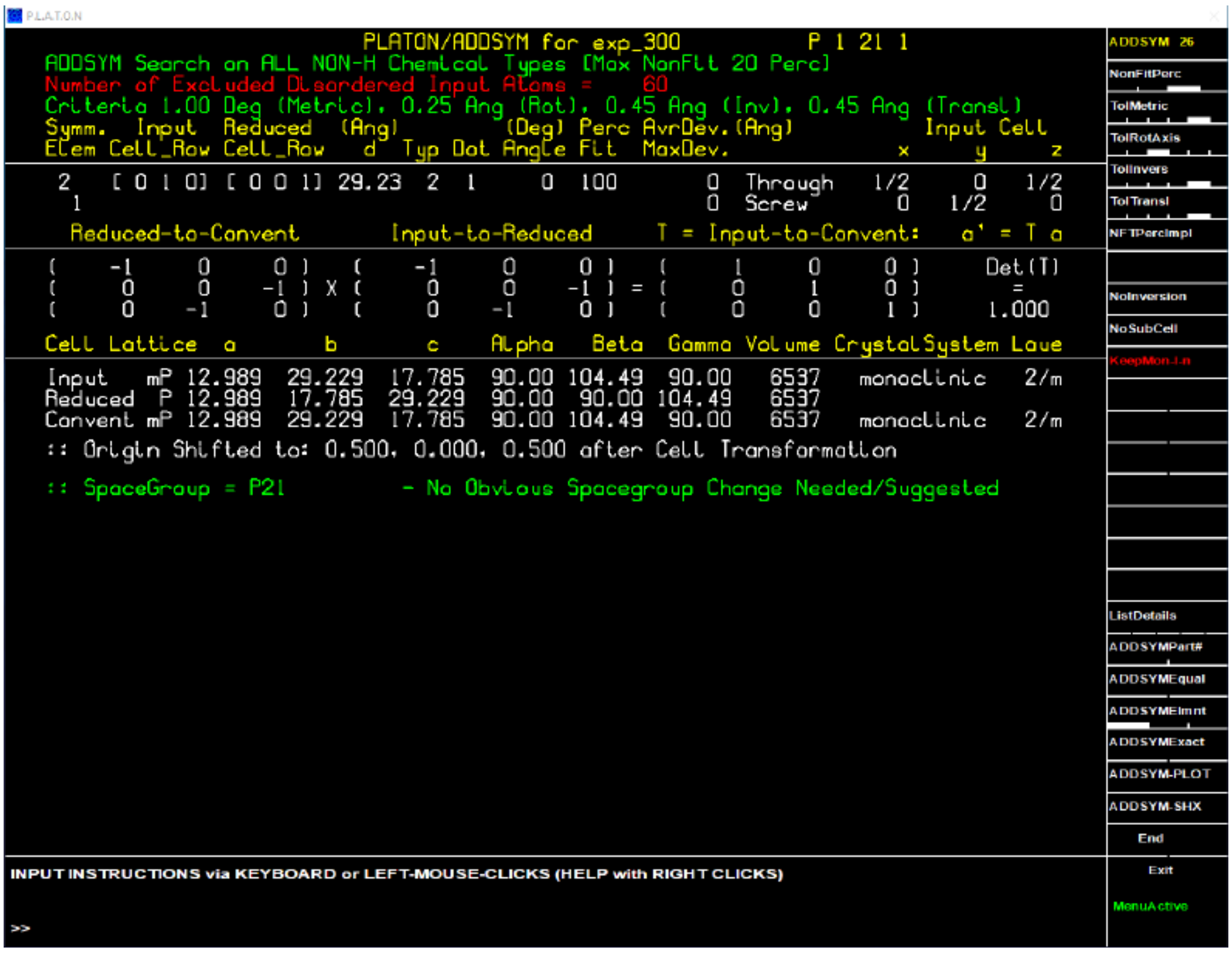
PLATON/ADDSYM for exp_300 P 1 21 1
ADDSYM Search on ALL NON-H Chemical Types [Max NonFit 20 Perc]
Number of Excluded Disordered Input Atoms = 60
Criteria 1.00 Deg (Metric), 0.25 Ang (Rot), 0.45 Ang (Inv), 0.45 Ang (Transl)
Symm. Input Reduced (Ang) (Deg) Perc AvrDev.(Ang) Input Cell
Elem Cell_Row Cell_Row d Typ Dot Angle Fit MaxDev. x y z
2 [0 1 0] [0 0 1] 29.23 2 1 0 100 0 Through 1/2 0 1/2
1 0 Screw 0 1/2 0
Reduced-to-Convent Input-to-Reduced T = Input-to-Convent: a' = T a
(-1 0 0) (-1 0 0) (1 0 0) Det(T)
(0 0 -1) X (0 0 -1) = (0 1 0) =
(0 -1 0) (0 -1 0) (0 0 1) 1.000
Cell Lattice a b c Alpha Beta Gamma Volume CrystalSystem Laue
Input mP 12.989 29.229 17.785 90.00 104.49 90.00 6537 monoclinic 2/m
Reduced P 12.989 17.785 29.229 90.00 90.00 104.49 6537
Convent mP 12.989 29.229 17.785 90.00 104.49 90.00 6537 monoclinic 2/m
:: Origin Shifted to: 0.500, 0.000, 0.500 after Cell Transformation
:: SpaceGroup = P21 - No Obvious Spacegroup Change Needed/Suggested
INPUT INSTRUCTIONS via KEYBOARD or LEFT-MOUSE-CLICKS (HELP with RIGHT CLICKS)
>>
ADDSYM 26
NonFitPerc
TolMetric
TolRotAxis
TolInvers
TolTransl
NFTPercImpl
NoInversion
NoSubCell
KeepMon.I.n
ListDetails
ADDSYMPart#
ADDSYMEqual
ADDSYMElmnt
ADDSYMExact
ADDSYM-PLOT
ADDSYM-SHX
End
Exit
MenuActive

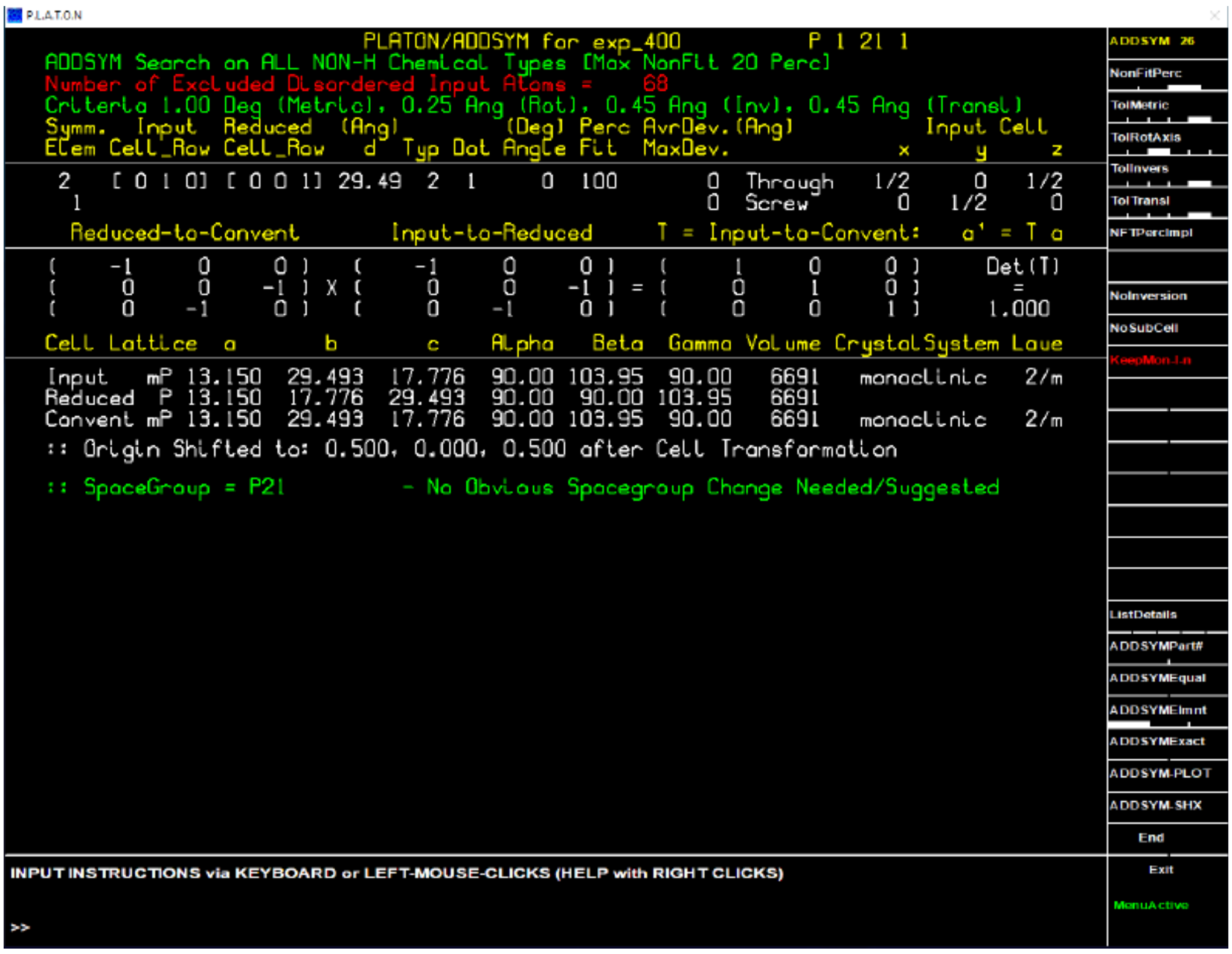
PLATON/ADDSYM for exp_400 P 1 21 1
ADDSYM Search on ALL NON-H Chemical Types [Max NonFit 20 Perc]
Number of Excluded Disordered Input Atoms = 68
Criteria 1.00 Deg (Metric), 0.25 Ang (Rot), 0.45 Ang (Inv), 0.45 Ang (Transl)
Symm. Input Reduced (Ang) (Deg) Perc AvrDev.(Ang) Input Cell
Elem Cell_Row Cell_Row d Typ Dot Angle Fit MaxDev. x y z
2 [0 1 0] [0 0 1] 29.49 2 1 0 100 0 Through 1/2 0 1/2
1 0 Screw 0 1/2 0
Reduced-to-Convent Input-to-Reduced T = Input-to-Convent: a' = T a
(-1 0 0) (-1 0 0) (1 0 0) Det(T)
(0 0 -1) X (0 0 -1) = (0 1 0) =
(0 -1 0) (0 -1 0) (0 0 1) 1.000
Cell Lattice a b c Alpha Beta Gamma Volume CrystalSystem Laue
Input mP 13.150 29.493 17.776 90.00 103.95 90.00 6691 monoclinic 2/m
Reduced P 13.150 17.776 29.493 90.00 90.00 103.95 6691
Convent mP 13.150 29.493 17.776 90.00 103.95 90.00 6691 monoclinic 2/m
:: Origin Shifted to: 0.500, 0.000, 0.500 after Cell Transformation
:: SpaceGroup = P21 - No Obvious Spacegroup Change Needed/Suggested
INPUT INSTRUCTIONS via KEYBOARD or LEFT-MOUSE-CLICKS (HELP with RIGHT CLICKS)
>>
ADDSYM 26
NonFitPerc
TolMetric
TolRotAxis
TolInvers
TolTransl
NFTPercImpl
NoInversion
NoSubCell
KeepMon.I.n
ListDetails
ADDSYMPart#
ADDSYMEqual
ADDSYMElmnt
ADDSYMExact
ADDSYM-PLOT
ADDSYM-SHX
End
Exit
MenuActive

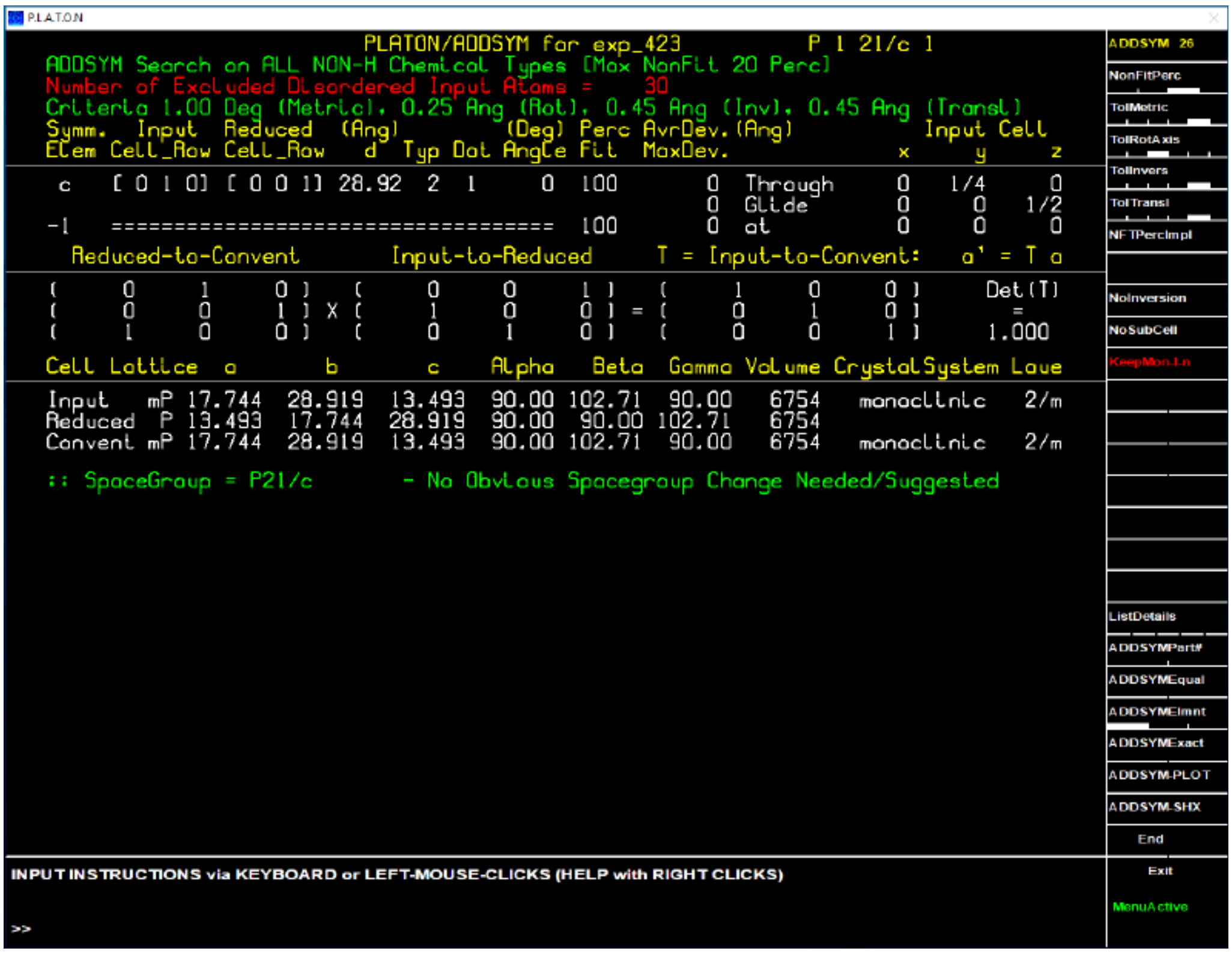
PLATON
PLATON/ADDSYM for exp_423 P 1 21/c 1
ADDSYM Search on ALL NON-H Chemical Types [Max NonFit 20 Perc]
Number of Excluded Disordered Input Atoms = 30
Criteria 1.00 Deg (Metric), 0.25 Ang (Rot), 0.45 Ang (Inv), 0.45 Ang (Transl)
Symm. Input Reduced (Ang) (Deg) Perc AvrDev.(Ang) Input Cell
Elem Cell_Row Cell_Row d Typ Dot Angle Fit MaxDev. x y z
c [0 1 0] [0 0 1] 28.92 2 1 0 100 0 Through 0 1/4 0
0 Glide 0 0 1/2
-1 ==================================== 100 0 at 0 0 0
Reduced-to-Convent Input-to-Reduced T = Input-to-Convent: a' = T a
(0 1 0) (0 0 1) (1 0 0) Det(T)
(0 0 1) X (1 0 0) = (0 1 0) =
(1 0 0) (0 1 0) (0 0 1) 1.000
Cell Lattice a b c Alpha Beta Gamma Volume CrystalSystem Laue
Input mP 17.744 28.919 13.493 90.00 102.71 90.00 6754 monoclinic 2/m
Reduced P 13.493 17.744 28.919 90.00 90.00 102.71 6754
Convent mP 17.744 28.919 13.493 90.00 102.71 90.00 6754 monoclinic 2/m
:: SpaceGroup = P21/c - No Obvious Spacegroup Change Needed/Suggested
INPUT INSTRUCTIONS via KEYBOARD or LEFT-MOUSE-CLICKS (HELP with RIGHT CLICKS)
>>
ADDSYM 26
NonFitPerc
TolMetric
TolRotAxis
TolInvers
TolTransl
NFTPercImpl
NoInversion
NoSubCell
KeepMon-Ln
ListDetails
ADDSYMPart#
ADDSYMEqual
ADDSYMElmnt
ADDSYMExact
ADDSYM-PLOT
ADDSYM-SHX
End
Exit
MenuActive

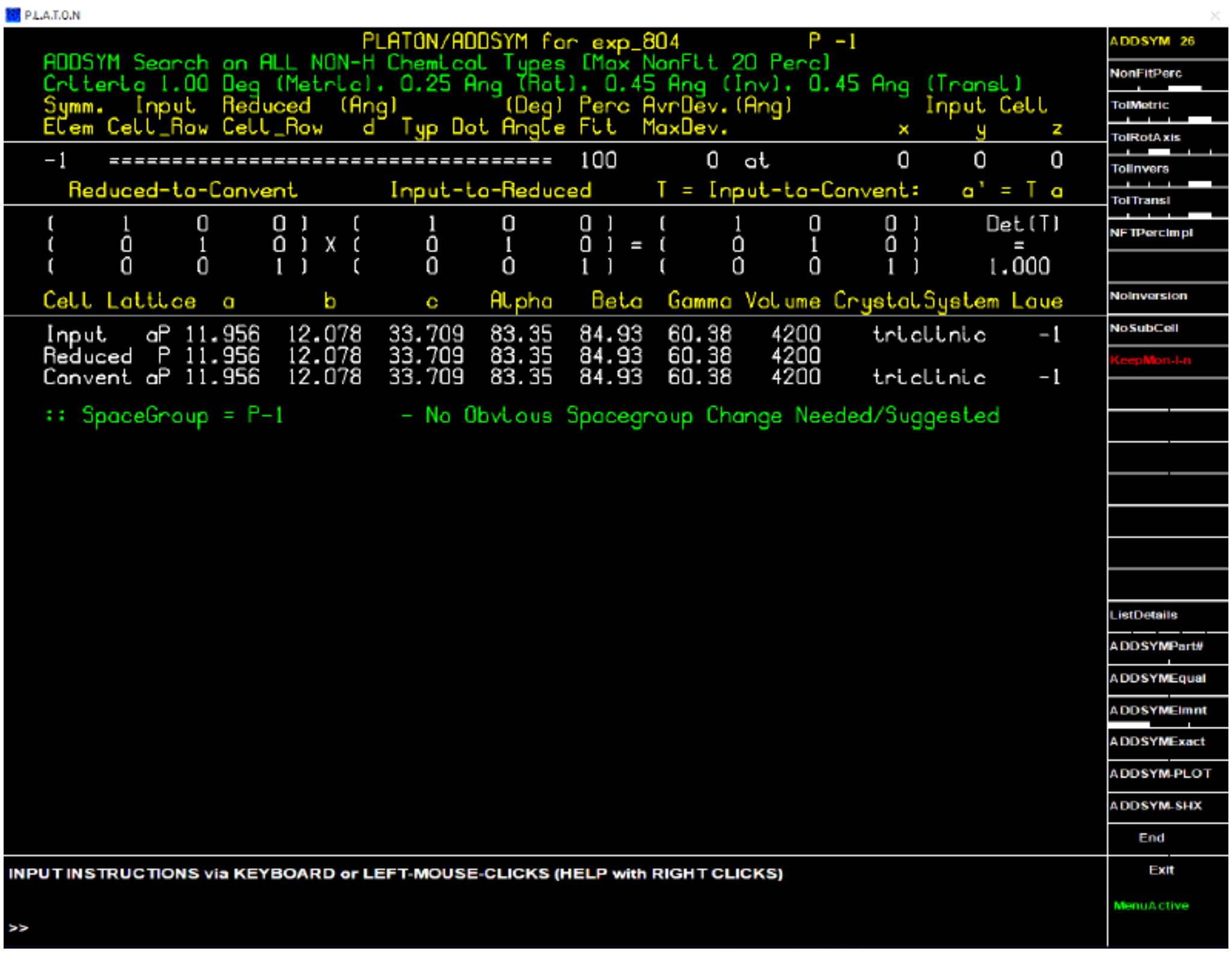
PLATON
PLATON/ADDSYM for exp_804 P -1
ADDSYM Search on ALL NON-H Chemical Types [Max NonFit 20 Perc]
Criteria 1.00 Deg (Metric), 0.25 Ang (Rot), 0.45 Ang (Inv), 0.45 Ang (Transl)
Symm. Input Reduced (Ang) (Deg) Perc AvrDev.(Ang) Input Cell
Elem Cell_Row Cell_Row d Typ Dot Angle Fit MaxDev. x y z
-1 ==================================== 100 0 at 0 0 0
Reduced-to-Convent Input-to-Reduced T = Input-to-Convent: a' = T a
(1 0 0) (1 0 0) (1 0 0) Det(T)
(0 1 0) X (0 1 0) = (0 1 0) =
(0 0 1) (0 0 1) (0 0 1) 1.000
Cell Lattice a b c Alpha Beta Gamma Volume CrystalSystem Laue
Input aP 11.956 12.078 33.709 83.35 84.93 60.38 4200 triclinic -1
Reduced P 11.956 12.078 33.709 83.35 84.93 60.38 4200
Convent aP 11.956 12.078 33.709 83.35 84.93 60.38 4200 triclinic -1
:: SpaceGroup = P-1 - No Obvious Spacegroup Change Needed/Suggested
INPUT INSTRUCTIONS via KEYBOARD or LEFT-MOUSE-CLICKS (HELP with RIGHT CLICKS)
>>
ADDSYM 26
NonFitPerc
TolMetric
TolRotAxis
TolInvers
TolTransl
NFTPercImpl
NoInversion
NoSubCell
KeepMon-Ln
ListDetails
ADDSYMPart#
ADDSYMEqual
ADDSYMElmnt
ADDSYMExact
ADDSYM-PLOT
ADDSYM-SHX
End
Exit
MenuActive

Appendix

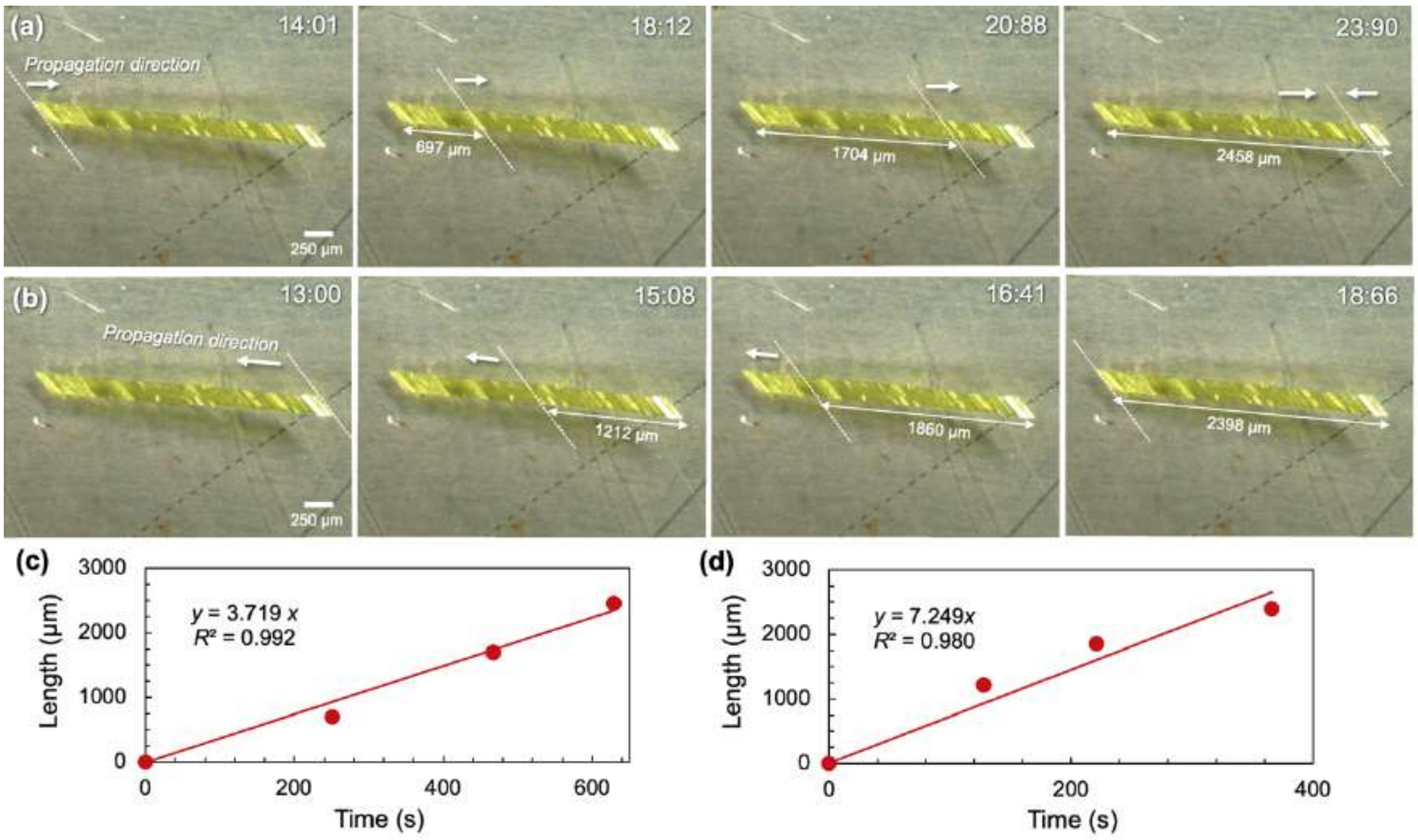

Figure R1. Time-sequential optical microscopy images and phase-boundary propagation in a one-end-fixed **BBPA** crystal during heating and cooling. Images recorded during (a) heating and (b) cooling; dashed lines mark the phase boundaries, and elapsed times are indicated. Phase-boundary position along the crystal long axis, measured from the fixed end, during (c) heating and (d) cooling.
